# The Role of rDNA Clusters in Global Epigenetic Gene Regulation

**DOI:** 10.3389/fgene.2021.730633

**Published:** 2021-08-31

**Authors:** Nickolai A. Tchurikov, Yuri V. Kravatsky

**Affiliations:** Engelhardt Institute of Molecular Biology Russian Academy of Sciences, Moscow, Russia

**Keywords:** rDNA, inter-chromosomal contacts, epigenetics, nucleoli, cancer, H3K27ac mark, super-enhancers, phase separation

## Abstract

The regulation of gene expression has been studied for decades, but the underlying mechanisms are still not fully understood. As well as local and distant regulation, there are specific mechanisms of regulation during development and physiological modulation of gene activity in differentiated cells. Current research strongly supports a role for the 3D chromosomal structure in the regulation of gene expression. However, it is not known whether the genome structure reflects the formation of active or repressed chromosomal domains or if these structures play a primary role in the regulation of gene expression. During early development, heterochromatinization of ribosomal DNA (rDNA) is coupled with silencing or activation of the expression of different sets of genes. Although the mechanisms behind this type of regulation are not known, rDNA clusters shape frequent inter-chromosomal contacts with a large group of genes controlling development. This review aims to shed light on the involvement of clusters of ribosomal genes in the global regulation of gene expression. We also discuss the possible role of RNA-mediated and phase-separation mechanisms in the global regulation of gene expression by nucleoli.

## Introduction

Nucleoli are the largest organelles in nuclei. They are not separated from chromosomes by any kind of membrane and potentially could shape contacts with chromosomal regions in interphase cells either without any particular order, or in some order to attain structural or functional features. If ordered, these contacts should be re-established in the course of cell division and epigenetic mechanisms may be involved. In interphase chromosomes, chromatin forms loops of different sizes that are required for both the compaction of chromosomes and for establishing a regulatory network. The close contacts between nucleoli and the chromosomal DNA were demonstrated by the co-isolation of chromosomal regions that are rather strongly attached to nucleoli during the isolation of nucleoli preparations (Németh et al., 2010; van Koningsbruggen et al., 2010). However, the size of the attached chromosomal DNA fragments (up to 1 Mb) did not allow a precise estimation of the contact sites of nucleoli in chromosomes or to determine their roles.

The Hi-C approach (Lieberman-Aiden et al., 2009) allows more precise mapping of the genome-wide chromatin contacts including those of the rDNA units. Using Hi-C or its derivative, the 4C (circular chromatin conformation capture) approach, it was possible to determine the rDNA contacts in human and *Drosophila* genomes. Of particular interest, the novel data suggested a role for nucleoli in differentiation. Localized heterochromatization of rDNA genes initiates the appearance of condensed chromatin structures in different genomic regions coupled with transcriptional activation of differentiation genes and the loss of pluripotency of embryonic stem cells (Feinberg, 2014; Savić et al., 2014). Modulating the rDNA expression fosters changes in the cell fate, growth, and proliferation of female *Drosophila*

ovarian germline stem cells and their daughters (Zhang et al., 2014). The mechanisms of the regulation of rDNA units and the factors involved are described in more detail in the recent review by Kresoja-Rakic and Santoro (2019).

There are two possible ways that rDNA units could modulate differentiation. The first is rDNA-mediated regulation by a remote mechanism that works at the level of unknown protein or RNA factors from active or silent rDNA units that initiate activation or silencing of different target genes. The second possible mechanism is the formation of dynamic direct contacts between rDNA units and different chromosomal regions that contain development-regulating genes. At present, both mechanisms should be considered. In this review, we discuss the recent data supporting the view that nucleoli are involved in the formation of 3D inter-chromosomal structures and that they shape contacts with different chromosomal genes, as well as the data on the role of phase-separation mechanisms in this type of regulation. We do not attempt to exhaustively review the literature and only refer to the main papers describing the most important ideas and findings in this area.

### Genetic and Molecular Evidence of the Regulatory Role of Nucleoli

**FIGURE 1:**
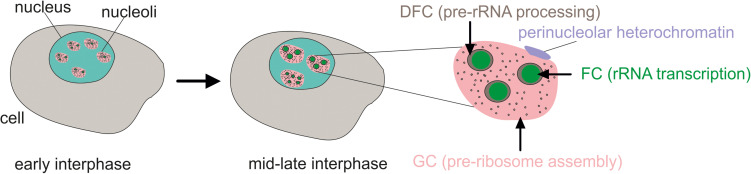
The tripartite structure of nucleoli as seen by microscopy. FC, fibrillar center; DFC, dense fibrillar component; GC, granular component. Transcribed rDNA units are at the border between the FC and DFC. In the early interphase, rDNA clusters form functional nucleoli. Then, during the interphase, the small nucleoli fuse to larger but fewer mature nucleoli or even one nucleolus.

Nucleoli are the largest membrane-less organelles in the nucleus. By light and electron microscopy, the tripartite structure of nucleoli can be observed including the fibrillar center (FC), dense fibrillar component (DFC), and the granular component (GC) (Figure 1). The clusters of rDNA genes reside around the FC while at the border of the FC and DFC, the chromatin loops that contain rDNA units are transcribed (Tiku and Antebi, 2018). The processing of 47S pre-rRNA and ribosomal protein assembly occurs in the DFC, and then the assembly of pre-ribosomal subunits is performed in the GC (Granneman and Baserga, 2005). Pre-ribosomal particles are formed in the GC using 5S rRNA, which is synthesized by RNA polymerase III from independent genes outside of the nucleolus, and the ribosomal proteins, which are transported from the cytoplasm to the nucleolus (Baßler and Hurt, 2019). There are several dozen FC–DFC modules in each nucleolus in human cells (Lafontaine et al., 2021). The number of FC–DFC modules is relatively constant for a particular cell type but differs widely between cell types, making it a powerful biomarker for cell classification (Lafontaine et al., 2021).

Some rDNA clusters are silent and form constitutive heterochromatin and are not associated with nucleoli (Akhmanova et al., 2000). Active rDNA units, which represent about half of the rDNA copies, are bound with upstream binding factor (UBF) and form nucleoli. Some inactive rDNA copies in the nucleolus are attached to the periphery of the nucleolus and shape co-called perinucleolar heterochromatin (Lindström et al., 2018). During the cell cycle, nucleoli disassemble at the prophase stage and begin to reassemble during the telophase (Pederson, 2011).

One way to determine the global role of rDNA expression on cell function is using a genetic approach to change the level of transcription by damaging some components of the Pol I machinery, pre-rRNA processing, or ribosome assembly. The *Drosophila* Pol I regulatory complex includes Under-developed (Udd) and TAF1B factors. Damaging *udd* or *TAF1B* leads to a reduced number of germ stem cell clones that produce differentiating cysts over time (Zhang et al., 2014). Similarly, active rDNA expression delays the differentiation of ovarian germline stem cells, whereas reduced rRNA production induces morphological changes that accompany early germline differentiation. These findings demonstrate that modulating rRNA synthesis promotes changes in the cell fate, growth, and proliferation of female *Drosophila* germline stem cells. The underlying mechanisms are not known; however, it is speculated that changes in ribosome biogenesis lead to changes in the expression of specific proteins that direct cell fate decisions, growth, and proliferation within an *in vivo* stem cell lineage more rapidly or to a greater extent (Zhang et al., 2014).

Important evidence in favor of the regulatory role of nucleoli in differentiation was obtained during studies of mouse embryonic stem cells (ESCs). Topological-associated domains (TADs) in ESCs are similar in different cell types and the chromatin is generally less condensed (Dixon et al., 2012; Nora et al., 2012). During differentiation of ESCs in mammals and *Drosophila*, large-scale silencing occurs and highly condensed heterochromatin appears in different chromosomes, including the regions of heterochromatic centric and pericentric repeats (Bhattacharya et al., 2009). In the course of differentiation, condensed heterochromatin regions are formed inside particular subsets of rDNA clusters. The nucleolar repressor TIP5, in association with long non-coding pRNA (promoter RNA), transcribed from the intergenic spacer (IGS) of rDNA, and some other factors are required for heterochromatin formation in some rDNA units (Santoro et al., 2010; Guetg et al., 2012). The analysis of the levels of pre-rRNA, rDNA methylation, and histone repressive marks in rDNA and satellites revealed that the formation of silenced rDNA units takes place during the transition from ESCs to neural progenitor cells and coincides with the switch to a more condensed heterochromatic form of centric and pericentric repeats (Savić et al., 2014). Interestingly, the silencing of particular rDNA units promotes the transcriptional activation or downregulation of hundreds of differentiation genes. These data suggest that nucleoli are involved in the regulation of chromatin states and the expression of genes associated with differentiation. The underlying mechanisms by which nucleoli control the expression of developmental genes in this model are unknown. How specific lncRNAs selectively locate the corresponding interaction sites in the genome is not understood and the nature of lncRNA-chromatin interactions, as well as their possible functional roles, is not yet clear (Rinn and Chang, 2012). It is possible that rDNA-derived lncRNAs are involved in targeting and regulating a specific set of developmental genes.

### Role of Nucleoli in Genome Stability, Aging, and Cancer

There is much evidence suggesting an important role for rDNA clusters in the regulation of cellular processes that are unrelated to ribosome biogenesis. For example, rDNA plays an important role in the DNA-damage response and in maintaining genome stability. The expression of rDNA is inhibited by DNA double-strand breaks (DSBs) induced by exogenous agents, e.g., ionizing radiation (Moore et al., 2011). DSBs occur under normal physiological conditions throughout the human genome, but the most fragile sites in the human genome coincide with actively transcribed rDNA genes, which possess hot spots of DSBs (Tchurikov et al., 2015). High transcriptional activity that leads to the formation of R-loops and to conflicts between transcription and replication within rDNA gene clusters are responsible for the DNA breakage of rDNA genes (Takeuchi et al., 2003; Lindström et al., 2018). There are nine hot spots of DSBs in the IGS of the rDNA repeats, denoted Pleiades (Tchurikov et al., 2016). The sites of these hot spots coincide with γ-H2AX marks, which suggests that the *in vivo* origin of DSBs is associated with transcription. However, Pleiades are only characteristic of active rDNA clusters that possess the UBF mark. It follows that a high level of DNA breakage inside the nucleoli should be accompanied by a high level of DNA repair (Korsholm et al., 2019).

The presence of hot spots of DSBs in rDNA explains the fact that there are 166 DNA-damage response (DDR) proteins found in the nucleolus (Hutten et al., 2011; Ogawa and Baserga, 2017). Among the proteins that are phosphorylated by kinases in response to DNA damage by ataxia-telangiectasia-mutated (ATM) and ataxia-telangiectasia and Rad3-related (ATR) kinases are 98 nucleolar proteins involved in ribosome biogenesis, ribosome function, and epigenetic regulation of rDNA genes. These facts led to the conclusion that the nucleolus is an important hub of the DDR (Matsuoka et al., 2007; Larsen and Stucki, 2016). The data suggesting a general role of nucleoli in chromosomal DNA repair were confirmed by the finding that many DNA repair proteins can freely relocalize from nucleoli to the nucleoplasm and contribute to DNA repair at different chromosomal loci (Antoniali et al., 2014). Nucleolar proteins constantly move between the nucleolus and the nucleoplasm (Hernandez-Verdun, 2006; Sirri et al., 2008). This constant movement is associated with other novel nucleoli functions beyond the formation of ribosomes, including ribonucleoprotein biogenesis and the regulation of mitosis and the cell cycle, as well as the response to several types of stress (Boisvert et al., 2007; Boulon et al., 2010; Lindström and Latonen, 2013; Latonen, 2019). Thus, nucleoli are dynamic functional hubs that coordinate genome integrity, DNA repair mechanisms, stress response, and other cellular functions.

Upon proteotoxic insults, such as proteasome inhibition or heat shock treatment, nucleolar aggresomes are formed within the nucleolus in nucleolar cavities and intranucleolar bodies (Latonen, 2019). Similar structures are formed in certain neurodegenerative disorders in which proteins and RNA accumulate and aggregate. Interestingly, several non-coding RNAs that are transcribed from the IGS can recruit proteins to the aggresomes (Audas et al., 2012).

Nucleoli are shaped by the most conserved DNA sequences and thus, could potentially serve as markers of cellular longevity and aging mechanisms (Tiku et al., 2017; Wang and Lemos, 2019). The nucleolus is considered to be a convergent point of regulation of major longevity pathways, which strikingly reduce nucleolar size and diminish the expression of the nucleolar protein FIB-1, ribosomal RNA, and ribosomal proteins across species; furthermore, the development of small nucleoli correlates with longevity in higher organisms (Tiku et al., 2017). The underlying mechanisms of this correlation are unknown. However, nucleolar size positively correlates with rRNA synthesis and the TOR signaling pathway regulates nucleolar size (Tiku and Antebi, 2018). The reduced TOR signaling leads to diminished nucleolar size and function, as well as increased longevity, in different organisms. On the other hand, active TOR signaling promotes growth and proliferation and is often hyperactivated in tumors, leading to increased nucleolar size (Derenzini et al., 1998). Genome instability can accelerate cellular senescence, which restricts the lifespan of a cell, and the stability of rDNA affects the lifespan (Kobayashi, 2014). It has also been proposed that rDNA clusters play a key role in maintaining the stability of the whole genome and the control of the cellular lifespan.

It has been suggested that changes in cytosine-5 methylation within CpG dinucleotides sites across the genome can be used to predict human chronological age, as well as aspects of biological age (Horvath, 2013). However, the mechanisms linking DNA methylation changes with age are also unclear. The methylation status inside rDNA units may explain both observed correlations (longevity with small nucleoli size and aging with DNA methylation). Human rDNA genes possess a high density of CpGs and potentially could be regulated by DNA methylation mechanisms during aging. The putative association of rDNA methylation with age was tested during aging in humans, mice, and dogs (Wang and Lemos, 2019). A significant age-associated hypermethylation of the rDNA relative to other regions of the genome was detected. However, the underlying mechanisms of this association are yet to be elucidated. Whatever the mechanism may be, it is not universal. In *Drosophila* and yeast, there is only low rDNA methylation and so other mechanisms of aging must exist beyond rDNA methylation.

rDNA genes are hot spots of DNA damage, and they often make intra- and inter-chromosomal contacts with different genomic regions that also possess hot spots of DSBs. These rDNA features lead to a high potential for translocations with different chromosomal regions, as well as with other rDNA clusters (Tchurikov et al., 2015). The latter could explain the origin of Robertsonian translocations that involve one or two rDNA-containing human acrocentric chromosomes (13, 14, 15, 21, and 22). The rDNA-mediated genome rearrangements could change the regulation of critical target genes and give rise to a cancer cell. As rDNA clusters consist of tandemly repeated genes, damage inside rDNA could be repaired by recombination with another rDNA copy and, as a result, the cluster could lose copies. It was observed that in 54% of solid tumors, there are rDNA cluster rearrangements before the start of clonal tumor expansion (Stults et al., 2009). The link between nucleoli and cancer was established more than 100 years ago by the observation of large and abnormal nucleoli in cancer cells (Pianese, 1896), which is thought to be due to hyper-activated transcription of rDNA (Hein et al., 2013). Cancer cells boost rDNA expression mainly via the genes involved in Pol-I-mediated transcription and through stimulation of their activity via different signaling pathways (for a detailed review see Gaviraghi et al., 2019). Although the link between nucleoli and cancer is well proved, the mechanisms of rDNA-mediated cancer genesis are not yet clear, possibly because, although we are aware that rDNA has many roles beyond ribosome biogenesis, the full list of cellular functions is unknown.

### Nucleoli Shape Frequent Contacts With Genes Controlling Differentiation

Evidence of the role of nucleoli in differentiation, aging, and cancer raises questions on the nature of the underlying mechanisms by which rDNA clusters regulate different cellular processes. From a general point of view, there are two possible ways for such regulation to occur. One is the regulation by factors that act at a distance, e.g., non-coding RNAs or regulatory proteins that are dependent on the expression of rDNA genes. The second possible way is through direct contacts of rDNA clusters with particular sets of genes by the formation of a net of nucleoli-mediated 3D chromosomal structures. The first microscopic evidence in favor of the formation of reproducible contacts of nucleoli with specific bands in *Drosophila* polytene chromosomes was found many years ago (Ananiev et al., 1981). More examples from *Deptera* were described later (Zhimulev, 1998). Then, molecular indications for the interactions between nucleoli and different chromosomal regions were gained from experiments on the co-purification of large stretches of chromosomal DNA (up to 1 Mb) with nucleoli preparations (Németh et al., 2010; van Koningsbruggen et al., 2010). However, this approach cannot accurately localize the contact sites of rDNA clusters within particular chromosomal regions or genes and so cannot reveal the regulatory targets. Therefore, high-resolution analyses are required because the regulatory influence of rDNA contacts could only spread to the nearest gene(s).

**FIGURE 2:**
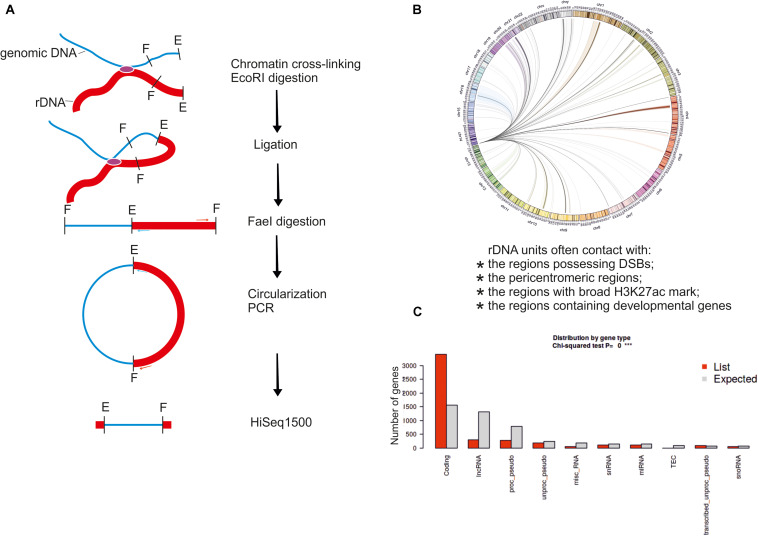
The 4C-rDNA approach for mapping of rDNA contacts in HEK293T cells. **(A)** Schematic presentation of the 4C-Seq approach. **(B)** Circos presentation of rDNA contacts representing at least 50 mapped 4C reads per million reads, which were filtered to remove the reads that entirely correspond to genomic repeats. Only one rDNA unit was included at the tip of chr14. **(C)** Distribution of rDNA contacts by gene type was determined using “ShinyGO v0.66: Gene Ontology Enrichment Analysis + more” software (http://bioinformatics.sdstate.edu/go/).

The high-resolution Hi-C and 4C approaches were used to more precisely localize the patterns of rDNA contacts in human cells (Yu and Lemos, 2018; Diesch et al., 2019). About 15 billion Hi-C reads from several experiments were used to map the rDNA-genome interactions with 1-Mb resolution. It was found that rDNA contacts are enriched in segments of closed, repressed, and late replicating chromatin, as well as CTCF binding sites (Yu and Lemos, 2018). Only a small portion of Hi-C reads represents the rDNA contacts. In contrast, the 4C-rDNA approach (Figure 2A) is more productive and allows amplification of only the DNA regions at the contact sites of rDNA. This approach was used to map rDNA contacts at better resolution (5 kb or less) using a MYC-driven lymphoma model or HEK293T cells (Diesch et al., 2019; Tchurikov et al., 2019).

The increased resolution confirmed the role of direct contacts of nucleoli with particular genes. First, the rDNA contacts are dynamic and their pattern changes during differentiation or in response to physiological stimuli.

Secondly, these changes in the contacts correlate with the changes in the expression of rDNA-contacting genes. Interestingly, in the lymphoma cells, gene expression changes at the rDNA-contacting loci include genes controlling B-cell differentiation, cell growth, and metabolism (Diesch et al., 2019). In HEK293T cells of neuronal origin, the nucleoli regulate the contacts with hundreds of genes controlling nervous system and neuron development (Tchurikov et al., 2019). In these cells, the contacts are detected in all chromosomes and often correspond to protein-coding genes (Figures 2B,C). In the MYC-driven lymphoma model, during the cellular transition from premalignancy to malignancy, there is a correlation between interactions of associated genes with the rDNA and transcriptional repression. These results suggest that the interactions with nucleoli contribute to Pol II gene regulation during the development of malignancy (Diesch et al., 2019).

**FIGURE 3:**
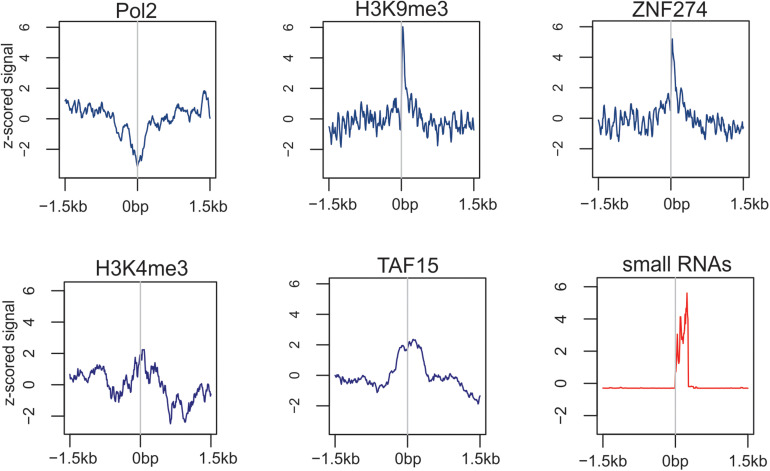
Profiles of binding sites of Pol II, modified histones, transcription factors, and small RNAs around the rDNA-contacting sites in HEK293T cells.

In mice, the nucleolus may act as a hub for the location and regulation of repressive genomic domains, whereas nuclear speckles are hubs of the location and regulation of active genomic domains (Quinodoz et al., 2018; Kresoja-Rakic and Santoro, 2019). These findings were supported by the observation of repressive histone modifications at rDNA-containing sites. Nevertheless, the detailed analysis of profiles ± 1.5 kb around rDNA-contacting sites in HEK293T cells revealed both active and repressive states around the rDNA contacts (examples shown in Figure 3), while in *Drosophila*, the contacting sites are enriched with repressive chromatin marks (Tchurikov et al., 2019, 2020).

The depletion of Pol II and enrichment with repressive H3K9me3 marks and the binding sites of the transcriptional repressor ZNF274 suggest the presence of silent chromatin at rDNA-contacting sites (Figure 3). At the same time, rDNA also makes contact with active chromatin regions, where TAF15 and active chromatin H3K4me3 marks are present. Interestingly, small RNAs often occur at rDNA-contacting sites, which suggests that RNA-mediated mechanisms may be involved in the contacts.

**FIGURE 4:**
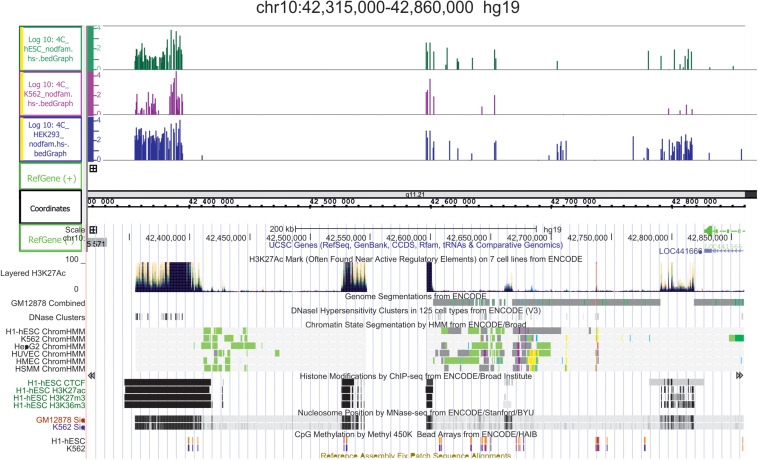
Conserved hot spots of rDNA contacts in three human cell lines coincide with the broad H3K27ac marks in chr10. The leftmost H3K27ac region at coordinate 42,400 kb is about 40 kb in length. Before the mapping, the 4C-rDNA reads were filtered to remove the reads that entirely corresponding corresponded to repetitive sequences (Dfam filtration, nodfam). 4C-rDNA data are shown for HEK293T, K562, and hESC cells (hESM01 line, Lagarkova et al., 2010). The Y-axis shows the log10 of the number of mapped 4C reads in three human cell lines that were not treated by heat shock (HS-).

In light of the above, we conclude that both active and repressed chromosomal regions shape the contacts with nucleoli. We speculate that both active and silent rDNA clusters could spread the corresponding chromatin state to the chromosomal regions that make contact and, thus, by these mechanisms, nucleoli participate in the organization of both active and repressed hubs in nuclei during differentiation. This conclusion is supported by the detection of conspicuous rDNA contacts in different human cell lines in 5–50-kb regions marked with active H3K27ac marks that may correspond to super-enhancers (Hnisz et al., 2013; Tchurikov et al., 2013); Figure 4 shows one example in chr10. The functional role of these regions is unknown.

**FIGURE 5:**
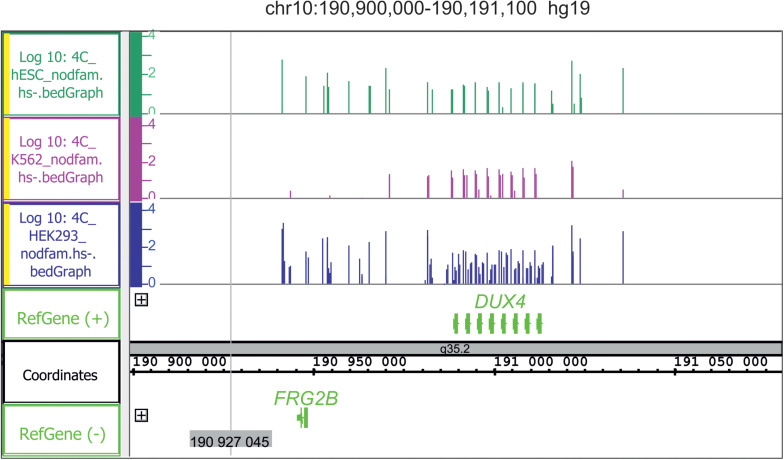
Conserved hot spots of rDNA contacts in three human cell lines with the *DUX4* gene cluster in chr10. Before mapping, the 4C-rDNA reads were filtered to remove the reads that entirely corresponded to repetitive sequences (Dfam filtration, nodfam). 4C-rDNA data are shown for HEK293T, K562, and hESC cells (hESM01 line, Lagarkova et al., 2010). The Y-axis shows the log10 of the number of mapped 4C reads in three human cell lines that were not treated by heat shock (HS-).

There are regions of frequent rDNA contacts that span about 100 kb in length and cover the silenced genes. One example is the *DUX4* gene cluster in the sub-telomeric region of chr4 (Figure 5). Heat shock treatment removes the rDNA contacts in this region (Tchurikov et al., 2020). *DUX4*, which is required at the two-cell embryo stage, specifies a transcription factor that activates hundreds of endogenous human genes (Hendrickson et al., 2017). At later stages, the genes are repressed and their abnormal activation leads to facioscapulohumeral muscular dystrophy (De Iaco et al., 2017). The data strongly suggest both the association of rDNA contacts with the silencing of human developmental genes and the dynamic character of the contacts. The regions of rDNA contacts may correspond to the repressed chromatin. The large rDNA-contacting region precisely coincides with the repressed chromatin state in the *FANK1* gene (Kretova et al., 2020b).

**FIGURE 6:**
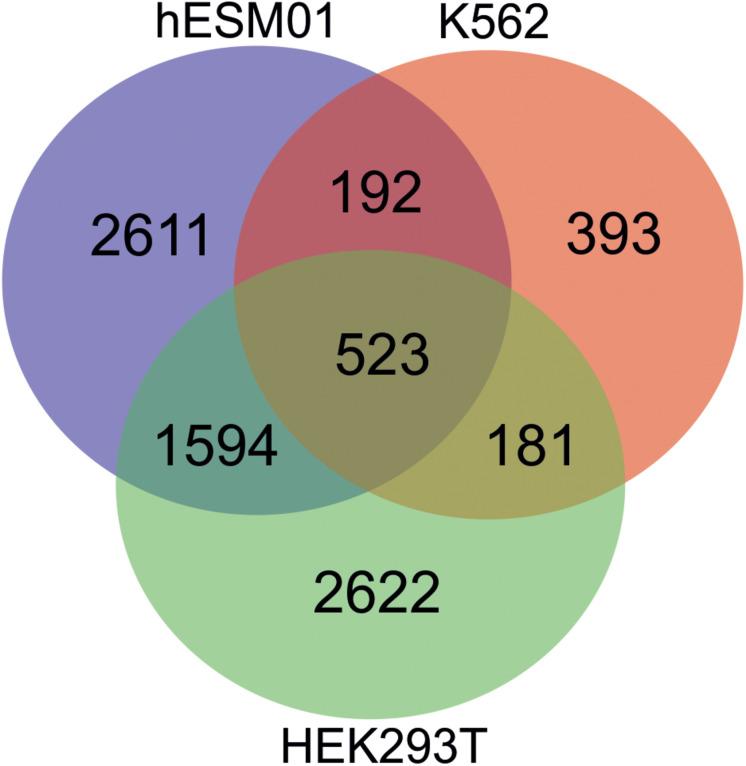
There are the conserved rDNA contacts in human cell lines. Venn diagram showing the numbers of overlapping rDNA-contacting genes between HEK293T, K562, and hESC cells (hESM01 line). The complete list of corresponding genes is shown in Supplementary Table 1.

Different human cell lines possess overlapping sets of rDNA-contacting genes that exhibit conserved rDNA contacts. For example, in HEK293T, K652, and hESM01 cells, the same set of about 500 genes frequently shape the contacts with rDNA (Figure 6).

Gene ontology searches suggest that the overlapping genes are involved in development and morphogenesis. About 100 of these genes (Supplementary Table 2) are highly associated with silencing by the H3K27me3 mark in several normal cell types, including bronchial epithelial cells, keratinocytes, myoblasts, monocytes, endothelial cells, and kidney epithelial cells (Tchurikov et al., 2021). Thus, a concerted silencing of a specific group of rDNA-contacting genes controlling development occurs during differentiation.

The association of nucleoli contacts with silenced or activated genes suggests the involvement of rDNA clusters in the global regulation of gene expression. Nevertheless, although the nucleoli may play a major role in the regulation of gene expression regulation, they cannot work alone. Therefore, nucleoli contacts are necessary but not sufficient for such regulation. There are likely many other players in the global regulation of gene expression, e.g., for the silencing of *DUX4* genes, LINE1 transcripts are required (Percharde et al., 2018). It is conceivable that active or silent DNA units harbor hundreds of RNA and protein factors and their complexes. Furthermore, dynamic rDNA contacts may be shared with different genes and DNA regions, thus leading to an active or repressed state, or to treat the DNA breakage, and so on, delivering tools for multiple processes. The data on the presence of small RNAs at rDNA-contacting sites (Figure 3) confirm this supposition. However, the rDNA-mediated epigenetic players remain to be elucidated.

There are three major classes of rRNA genes in mammalian cells: silent, inactive, and active (Bersaglieri and Santoro, 2019). Silent rDNA units are characterized by DNA methylation in their promoter regions and by the presence of repressive histone marks, such as H3K9me2, H3K9me3, and deacetylated histones (Zhou et al., 2002). The active and inactive rDNA clusters do not possess DNA methylation in their promoter regions but may carry significant DNA methylation levels in, for example, the non-coding IGS (Moss et al., 2019). The active clusters are epigenetically marked by UBF and are nucleosome-free in the rDNA coding region, while inactive genes do not possess UBF marks and are packed with nucleosomes (Mars et al., 2018; Bersaglieri and Santoro, 2019). Therefore, the 4C experiments should reflect the inter-chromosomal interactions of all rDNA classes. We assume that ChIP-Seq experiments that aim to reveal the genome-wide localization of nucleolar proteins (UBF, fibrillarin, or nucleolin), which are present at active rDNA units, could help to discriminate the inter-chromosomal contacts of nucleoli from the contacts of the silent or inactive rDNA clusters.

### Nucleoli and Phase-Separation Mechanisms

Active rDNA clusters organize nucleoli and rebuild them after each cell cycle (Hernandez-Verdun, 2011). UBF, which is required for the activation of rDNA units, is epigenetically inherited and marks the clusters that were active in the previous cell cycle and are destined to be active in the next cell cycle (O’Sullivan et al., 2002; McStay and Grummt, 2008; Dimitrova, 2011). The same is true for γ-H2AX that marks the active rDNA clusters (Tchurikov et al., 2016). Inter-chromosomal rDNA contacts are also re-established in every cell cycle. These data suggest that epigenetic mechanisms are involved in the lifecycle of nucleoli and their 3D network.

Nucleoli in their genomic contacts prefer some epigenetic marks, e.g., active H3K27ac marks (Tchurikov et al., 2015). At present, we do not know whether these marks appeared before or after the contacts were made with rDNA clusters. The H3K27ac mark is associated with super-enhancers and with phase-separation mechanisms (Hnisz et al., 2013; Sabari et al., 2018). The link between rDNA contacts, broad H3K27ac marks, and super-enhancers suggests the involvement of nucleoli in phase-separation mechanisms (Tchurikov et al., 2020). The H3K27ac mark is a characteristic of super-enhancers and was used to create a catalog of super-enhancers in different human cell and tissue samples (Hnisz et al., 2013; Tang et al., 2020). Super-enhancers have a high density of different transcription factors, which makes them a source of the nucleation event during phase separation (Mansour et al., 2014). MED1, a subunit of Mediator, and BRD4, a chromatin reader protein that recognizes and binds acetylated histones, occupy discrete nuclear bodies that occur at super-enhancers (Sabari et al., 2018). These bodies are disrupted by 1, 6-hexanediol, a drug that disrupts liquid-like condensates, possibly by disruption of hydrophobic interactions (Kroschwald et al., 2017). These results show that transcriptional coactivators form phase-separated condensates at super-enhancers. Together with the data that nucleoli frequently form contacts with the regions decorated with broad H3K27ac marks (Tchurikov et al., 2015), these data suggest that nucleoli are associated with phase-separated condensates. The idea is supported by data showing that transcriptional condensate formation contributes to long-range genomic interactions (Shrinivas et al., 2019).

In general, all genomic repeats could generate phase separations (Hall et al., 2019). The tripartite structure of the nucleolus itself, which separates the FC, DFC, and GC, also depends on the phase separation of different protein components. The large number of different factors controlling transcription and DNA repair and non-coding RNAs that accumulate at rDNA units could be the source of the nucleation event of phase separation and the formation of nucleoli in each cell cycle. Recently, novel chaperone-like properties of the nucleolus as a phase-separated organelle associated with the refolding of misfolded proteins were described (Frottin et al., 2019). Metastable nuclear proteins that misfold after heat shock treatment could enter the nucleoli where they avoid irreversible aggregation and remain competent for HSP70-dependent refolding upon recovery from stress.

The cognate phase-separated structures on chromosomes could promote the interaction between condensates of the same nature, including nucleoli interactions. The regions of inter-chromosomal rDNA contacts may compete with the local intra-chromosomal contacts and displace them. In *Drosophila* genes, the multiple nucleoli contacts are located in the center of a bubble around which the main chromatin loops are formed (Kretova et al., 2020a; Tchurikov et al., 2020). The forces of phase-separation mechanisms of nucleoli are probably stronger than those between intra-chromosomal loops in this region.

Recently, it was demonstrated that fibrillarin, the dense fibrillar component constituent, and nucleophosmin, the scaffold protein of the granular component, are implicated in nucleation, including the tripartite organization of nucleoli (Yao et al., 2019; Lafontaine et al., 2021). In direct experiments, the 5′ end of nascent 47S pre-rRNA binds co-transcriptionally to the RNA-binding domain of fibrillarin, which diffuses to the DFC (Yao et al., 2019). In the DFC, the local self-association between glycine- and arginine-rich domains of fibrillarin shapes the phase-separated clusters that immobilize fibrillarin-interacting pre-rRNAs. In this way, the directional traffic of nascent pre-rRNAs occurs, thus facilitating pre-rRNA processing and DFC formation. *In vitro* droplet reconstitution with purified fibrillarin and nucleophosmin showed that the proteins readily form condensed liquid droplets that exhibit biophysical features similar to those of intact nucleoli (Lafontaine et al., 2021).

The nature of the nucleolus, which is made up of phase-separated compartments itself, suggests a potential role in the long-range dynamic interactions in the nucleus because liquid-liquid phase separation physically allows the rapid movement of components into and within the dense phase (Brangwynne et al., 2009). These interactions are dynamic and dependent on the differentiation state, phase of the cell cycle, and external physiological conditions. The dynamics of nucleoli correspond to the dynamic organization of chromosomes revealed by live-cell imaging data that suggest an organized motion of highly viscous droplet-like domains that can be likened to chromatin “breathing” (Latonen, 2019; Misteli, 2020; Shaban and Seeber, 2020; Feric and Misteli, 2021). The understanding of nucleoli structure and function has come a long way from the 1830s (Pederson, 2011). Novel approaches could elucidate the phase separation mechanisms underlying the structure and functions of nucleoli as the most remarkable component of nuclei.

## Conclusion and Future Perspectives

Nucleoli play many important roles beyond the biogenesis of ribosomes, including shaping of the nuclear architecture and regulation of DNA repair, differentiation, chaperone-like functions, RNP formation, diverse stress responses and others. The abnormal function of nucleoli leads to cancer genesis and diseases. Recently, it was demonstrated that inter-chromosomal contacts of nucleoli are involved in the regulation of global gene expression. The nature of these contacts and their role in development remains to be elucidated. It is not clear how rDNA inter-chromosomal contacts affect the local intra-chromosomal 3D domains. The contacts may be important for other functions of the nucleoli, including DNA repair and stress responses. The key areas for study in the future include determining the underlying molecular mechanisms of nucleoli function as a driver of nucleoli’s role in cellular development and the response to environmental stimuli, the RNA-mediated mechanisms involved in recognizing target genes, and the phase-separation mechanisms in the formation of nucleoli and their dynamic 3D structures.

## Author Contributions

NT wrote the manuscript. YK developed software for image and statistical analysis. Both authors contributed to the article and approved the submitted version.

## Conflict of Interest

The authors declare that the research was conducted in the absence of any commercial or financial relationships that could be construed as a potential conflict of interest.

## Publisher’s Note

All claims expressed in this article are solely those of the authors and do not necessarily represent those of their affiliated organizations, or those of the publisher, the editors and the reviewers. Any product that may be evaluated in this article, or claim that may be made by its manufacturer, is not guaranteed or endorsed by the publisher.
