# Supplementary material for: The Role of rDNA Clusters in Global Epigenetic Gene Regulation
Source: Front Genet. 2021 Aug 31;12:730633. doi: 10.3389/fgene.2021.730633 (PMC8438155; doi:10.3389/fgene.2021.730633)
Supplement: Supplementary file 1 [file Data_Sheet_1.PDF]

Table S1. The overlap between 4C-contacting genes in three cell lines. Related to the Venn diagram in Figure 6.

Names	Total	Elements
HEK293T-hg19-4020 K562-hg19-1289 hESC-hg19-4020	523	*PKNOX2 MIR4461 RTN1 ERG PDE1C WSCD1 NFIA SYN3 RPS6KA5 SUGCT EIF4G3 SLC8A1 PCDHA13 ANKRD20A3 ABCD1P3 SAMSN1 CDC42EP3 IGSF11 DPYD ARHGAP15 LINC00486 LCA5L SKAP2 IGLV3-7 DUX4L3 MIR3118-2 UPK3B ASTN2 CSMD3 DLGAP1 DUX4L15 BICD1 LRP1B ABCG8 OTUD7A SUMF1 NF1 FPGT-TNNI3K PCDHA12 SNX18P25 ARID1B WDPCP ADAMTS17 MTND6P3 SPATA16 ST6GALNAC3 ADAMTS6 GRID1 PLXNA4 HFM1 ACSS3 CNBD1 CACNA1C FOXP2 FSD2 CDC27P2 NCOR1P1 SH3TC2 PCMTD1P2 RN7SL92P ANKRD20A14P NDST3 NBEA TIAM2 IQCJ-SCHIP1 LINC00273 DUX4L12 PLEKHB2 GLIS3 ANKRD36 NRG3 PTPRG NCOR1 ELMO1 DIO2 ABCD1P4 WDR27 SLC9B1P4 FBXL17 ZNF585A CACNB2 BRE BACH1-IT2 SOX6 TBC1D22A TRPM3 TACR3 DNAH8 SHANK2 UNC5D GABRA5 DMD ADCY2 ZRANB2-AS2 ANKRD20A4 MIR3118-3 FNDC3B MIR1324 GNG12-AS1 ATRNL1 TMEM135 DUX4L2 CPE SH3GL2 ARHGAP24 TPTE2P6 ZNF804A WRB XKR4 GPC5-IT1 CCDC178 KAZN ADAMTSL1 ZNF675 OR4F16 DTNA PHKB KMT2C VWA3B ZNF280D HECW1 AFF3 GPHN SLC9B1P3 ELP4 LAMA2 ZNF385D REXO1L10P ZSCAN5A MYO3B DIP2C RGS3 SPAG16 DSCAML1 DUX4L8 RERG MAGI2-IT1 TBC1D5 CNTN4 MUC16 MRPL33 KIFAP3 PON1 VWA8 SNAP25-AS1 HMCN1 N4BP2 POTEE EPHA5 VN1R7P MERTK GRIK4 NPSR1-AS1 RUNX1 TSHZ2 MTND4P12 IGHV1OR21-1 PCDHA2 PCDHA11 MIR663A NOTCH2NL LINC00894 LINC00669 SATB2 KIAA1217 ANKS1B SPOCK3 INTS4L2 ANK2 REXO1L8P ROCK1P1 INVS ANO2 PCDHA9 SLIT3 BAGE2 ROBO1 ANKRD30BL IFNGR2 DGKB DUX4L13 KLF12 ANKRD36BP2 SGMS1 CCDC30 MIR1299 KHDRBS2 MGMT CPQ TM4SF2 EHBP1 HNRNPA1P7 FGF14 CSRNP3 FRG2C ITGAM DUX4L7 MGAM DUX4L10 PRDM11 CACNA1D PACRG PCDHA10 LANCL3 SNX29P2 CUL5 DSCAM DGKI MTND1P23 ANKFN1 TRAPPC8 SDK1 SLC9B1P2 GRM5 EPHA6 CA10 ARAP2 SNRPN CORIN TEX41 PDZRN4 CNTNAP2 SHFM1 AQP4-AS1 MIR96 BRINP2 EFTUD1 MSRA PCDH9 NKAIN2 CST13P REXO1L1P COBL MDN1 ATP6V0D2 ARHGAP6 MCM3AP PCDHA1 Y_RNA PCDHA8 GPM6B ANK3 ZMAT4 LHFPL3 THSD4 GPC6 RELN LINC00284 HS3ST4 ADAMTSL3 SNHG14 CDH4 GABRA3 DPP10 OCA2 ARHGAP22 SUSD1 HAPLN1 SPOCK1 MIR3118-1 APBA1 PAK1 FRAS1 CACNA2D1 PON3 CHRM5 LINC00693 PCDHAC1 CTBP2P8 UTRN GPC5 ENOX2 AGBL4 TENM4 DUX4L18 CASC16 PPP3CA DUX4 MIR3118-6 PTK2 RNA5-8SP2 LRRC4C INTS4L1 AVEN LINC01090 DUX4L6 LRRK2 DYNC1H1 CLSTN2 MTND2P28 TTLL11 DUX4L11 PRRG1 TCF4 FAM126B LIPI FMN1 ANKRD20A1 ZFPM2 PIEZO2 SLC35F1 NTM LINC00960 PRMT8 IFT43 XRCC4 LRBA FHIT NSG2 MIR3687 LINC00842 AGGF1P2 SPIDR NAV2 MIR183 RUNX1T1 PCDH15 SGCZ SEL1L2 GRM1 PDE4D TRPC4 CNTN5 RNA5SP519 PCBD2 SLC25A15P4 PCDHA7 HUNK NMD3P1 REXO1L11P EPHB1 LINC00478 FHOD3 DOCK1 DIAPH3 REXO1L9P ANKUB1 LINC00536 PCDHA5 TLN2 ZNF493 ZNF717 TRPC5 PLCB4 CASC15 UNC5C ANKRD36C CSGALNACT1 CSMD2 SORCS3 ATP1A4 OPCML DUX4L19 MGAT4C COL6A5 CCDC91 MMP26 CALN1 LRP2 SEMA6D TSPY5P IGLV2-8 FBXL7 MAPRE2 SGCD CA3 GABRB3 TPTE SEZ6L GRM7 MIPOL1 PTPRM KIRREL3 NRXN3 DLC1 GABBR2 ATP8A2 BMS1P15 GPR139 MACROD2 TMTC1 AUTS2 MTND6P4 ERC1 COX10-AS1 PCDHA6 DUX4L16 WLS RYR3 ZPLD1 RNA5-8SP6 CTNND2 DNAH14 MAST2 COL22A1 CDH13 SAMD12 DACH1 TRDN DAB1 TEKT4P2 PCDHA4 SNTG1 ALK EXOC6B DNAH6 REXO1L3P MYO5B DUX4L5 STIM1 ANKRD30BP1 FAM135B LRRC69 AKR1C2 MIR3118-4 HDAC9 ELOVL7 CDK6 LARGE PCMTD1P1 KYNU DIO2-AS1 TBXAS1 TTLL11-IT1 CNTNAP4 SCAI PGM5P2 NCOR1P3 MIR3648 SPECC1 FAM155A PTPRT KY CSMD1 SLC24A2 COL25A1 MTND5P11 SLC35F3 ANKRD20A12P CTNNA3 VPS13D FREM2 hsa-mir-6723 B3GALTL MLLT10P2 THSD7B FAM227B ZNF618 U3 AGBL4-IT1 MIR3118-5 DNM3 SYNDIG1 ASXL3 DHX32 LINC00856 DUX4L17 DLG2 NCKAP5 PTPRD STXBP5L ANKRD20A2 PLCB1 AGGF1P1 PRKG1 ZNF283 CTBP2P1 CHST9 SPTLC2 TTC28 MAGI2 SNX18P15 PLCL1 EDA CCDC144CP ASIC2 PRKD1 ADK TNNI3K RANBP17 TRIM51CP RYR2 SEMA3D DUX4L4 FAT3 PCDHA3 SYCP2 TMEM163 CLVS1 EYS BACH1 ROBO2 CREBRF CTBP2 SRGAP3 PGM5P1 AKAP13 MYRIP DUX4L14 PCP4 CCDC171 SCN8A FRG1B KSR2 HIVEP3 SEMA5A GAREM PRDM15 EIF3FP1 LRRC16A DCC IGHV1OR15-2 PARD3B SLC2A9 snoU13 FAM110B NRG1 SLC22A10 NPAS3 MLTK FMN2 DGKZP1 KIAA0825*
K562-hg19-1289 hESC-hg19-4020	192	*RIT2 MMP2 TMEM132E MRPL1 RAPGEF6 MTND5P12 ROR2 CCDC41 ERBB2IP MYBPC1 RSU1 TFEC CNTNAP3B USH2A FGF12 MSTO2P GNGT1 FAM13A RPL36P1 ALDH1L1 WDR70 BRCA2 SLC30A8 TACC1 ITGBL1 PTPRQ SPATA3 ZDHHC20 ENPEP RPRD1A CD247 HADHB NAB1 ACOXL MIR4500HG RAB28 ST3GAL5 JPH1 ACTN1 DOCK7 NOXRED1 BNC2 KRBA1 CLEC9A LINC00883 SENP5 TAF4B UGT3A1 ARPP21 SCOC GALNT13 MIR204 DOCK8 RNU6-67P LINC00511 PARVA CCSER1 USP10 MED8 STAT4 ZNF609 CCDC83 LPXN NPIPA7 MTND5P10 LINC00535 GPR12 MYPN ANP32AP1 ZEB1-AS1 KIF21A OXR1 C8orf34 MLLT3 PRKAR2B MUC19 ZNF542 RANBP3L SEPSECS DIP2B ALDH1A1 TMCC1 UBE4B FRK CD48 MLLT10 ZSWIM5 RAB11A ULK2 ANKIB1 SHROOM2 LINC00578 HIST2H2BF UBE2V2 TTC7B OR10AG1 ZNF32-AS3 MEIS2 TDRD5 PKD1P5 DLGAP4 DPH6-AS1 RNU1-59P OSBPL10 CLTCL1 ASXL2 PDGFD DNAJC16 TEX2 ANO10 NCKAP5L SYT7 DLEU2 DMGDH TPD52L1 L3MBTL4 PPM1E ASAH2B MSTO1 NNMT PDE7B NDUFAF4P2 CCDC102B HERC1 EXT1 ABCA10 IQCE MIR1273C ENTPD1-AS1 NPIPA8 DIRC3 ZNF423 PCMTD1 TRPC6 ADAM18 NLRP4 ADAMTS3 FFAR2 RAPGEF2 ENTPD1 AGPAT5 RABGAP1L XYLT1 EYA2 TMTC2 PDE3B ACTR3C PDE6A RBFOX3 CMIP JADE3 SYT2 ENPP7P4 TFCP2L1 USP24 TUFMP1 PPARG SLC25A24P1 PLXNC1 TOM1L2 PBRM1 SCGB1D4 MYO5A KANSL1 MEI4 SRP9 LHFPL3-AS1 FAM169B C2CD5 CEP57L1 ROCK1 LIFR CLCN5 CACNA1A CNTRL PTH2R JARID2 GTPBP10 GLYAT PRLR KLHL29 ZNF100 RAVER2 MST4 NEDD9 CLRN1-AS1 SYT14 PDE8B RNF13 HNRNPA1P33 XIRP2 PEPD*
HEK293T-hg19-4020 hESC-hg19-	1594	*DPY30 KCNMA1 C10orf90 RALYL BBOX1 KLHL13 REXO1L12P GLT1D1 FRG2 GGT3P MTHFD2L ALDOAP2 TMC4 SCAF8 CPXM2 C4orf22 CDK14 PARN XK SEMA4D BANK1 NINL*

4020		RNF185 TNS3 IGSF3 MED13L CXCL13 KIAA1199 CNTN4-AS2 POTED ZNF510 GADL1 STX8 PRKCB BAZ1B PHF20 CPNE4 GRK5 FGGY RNU6-749P OR8J3 ANKRD6 GPR55 STAU2 RSU1P2 SIAH3 LINC01121 GML LINC00159 IGHV3-25 GSTA2 OR7K1P ARHGAP42P4 FBXO31 C12orf40 FRS2 HDAC7 PIWIL3 FPGT OPHN1 TRAPPC9 FAM182B KIAA0100 FNDC1 LCMT1 MMP16 CAMTA1 KIAA1324 OR4M2 PLCL2 C4orf29 GXYLT1P1 ADAM32 RSRC1 CHST11 FGF7P2 SLC9A9 ZSCAN5C IPP ADAM23 B3GALT5 RAG1 TSPAN7 HERC2P8 FSIP1 GRIK2 RNA5SP284 CDK17 MAPK10 OTX2-AS1 LRRTM3 OR4A42P COX10 GPATCH2 ZNF227 CDH8 KCNQ5 ZBTB20 HEPHL1 LTBR HBG2 PRR5 PCSK6 SPRR2B PALM2-AKAP2 AHI1 BDH2P1 UBE2W ACTR5 HK3 LPIN3 LRRC53 ATP10A DLEC1 C7orf60 ACAD11 NEGR1-IT1 SCHIP1 SYTL5 PIGL KIF19 DYRK4 ZNF667 PPARGC1A KCNJ6 ZNF709 ETFA FSTL5 AHRR BRD4 GPX1P2 GPR158 ITSN1 NDRG2 TMEM241 GRIP1 APBA2 TTC3 P2RX6P ZNF962P CMA1 TLK1 FAM9B TANC2 WDR16 ZNF863P DEFA1B RCAN1 FAM193A TMEM189 EDDM3B LGR5 JAK2 TM9SF4 SIRPG AKAP2 PHGDH SNX18P9 TPTE2 EMCN-IT2 ANKRD20A5P KALRN CCDC60 MKLN1 DOPEY2 SDHD PPFIBP1 USP24P1 NEGR1 MAP4K3 CACNG2 IGLL5 SNORA75 BTBD9 CD93 TAF1D IGHVIII-47-1 STRN3 MEGF11 FBXL5 NR2F2-AS1 ZNF536 HS3ST5 KAT5 MAP6 PARVB CDH11 CDK2AP2P1 ZBTB7C ITGA2 GRIN2A NRXN1 SNORD116-27 FAM230C DSCAM-IT1 MBL1P SPTLC3 AGBL1 PARK2 LINC00226 NELL2 DEFT1P2 IL1RAPL1 MAGI1 LAMA3 SLC14A2 RNU7-87P KIR2DL1 FOXO1 GBP4 WDR82 NPHP3 RERGL TNR-IT1 SETD3 DPP6 CD109 SLC22A3 C2orf48 ARHGAP8 PTGER4P2 LINC00504 ANAPC1P1 ZDHHC17 KCNH1 HLCS FEM1AP4 PDCD6 CCSER2 AMPH BRI3BP LMO7 RNU6-768P MAN1A1 PCED1B RNU6-1021P NLGN4X EXOC4 SPRR2C HEATR5A BPIFB1 HTR4 PPP1R26P3 EMB FAM19A5 ANO4 GIPC2 BBS9 C5orf64 FAM83B OR4K15 ST7L LINC00595 COMMD1 MYO9A CCDC3 NTRK2 FOXN3 ENTHD1 RNU6-917P OR5K4 PAK7 WDFY3 ADHFE1 SH3PXD2B IGKC AK8 POTEC FAM19A2 ANKRD30BP2 OR5L2 ZNF285 AFAP1 NCAM2 OC90 CYSLTR1 SLC20A1P3 ZNF10 TRAK2 C14orf37 CEACAM5 MYT1L SRGAP2B STAG1 CLDN14 SCARNA15 KIR2DP1 LRRC49 SMARCA4 HERC2P3 CNTN1 TTC39B SNORA1 MTATP8P1 HBE1 ABCA11P PLAC1 MC2R BTBD11 SYNE1 TUBA3FP PKHD1L1 PDZD2 IFT88 ARHGAP5 ANKRD20A9P KIAA1731 DNAH10 GAS2 PDE10A GRXCR1 NUMB STPG2 STXBP4 TOX2 PSG10P ZNF720 LIN9 RHOJ PHEX CIRH1A FAM182A POTEM SYBU FILIP1L PDE4DIP COL4A6 CATSPERB ZMYM4 STK32B LINC01020 ACTBP8 BRMS1L POTEB2 NHS CNTNAP5 IGLC2 MIR670HG RAPGEF5 CDCA2 DTD2 ZMYM2 TAB3 SDIM1 CHCHD6 RNU6-631P CD8B TRIM48 KIRREL PDGFRA DOK5 PTPRJ C22orf39 RNU4-56P STAG3L2 AGMO OTC MRPS6 MYH4 ASTN1 ADAMTS9-AS2 SH3BGR NF1P8 KIR3DL3 DTX2P1 CHRM3 WARS2 LINC00587 UBE2V1 RFFL CALD1 IGLJ2 EIF3E TRDV1 RPL21P41 KIAA1462 ABCC13 COL24A1 SNX18P8 GAK C10orf113 ALMS1 TMEM132D COL11A1 SLC13A3 LINC00907 IGHV1OR15-9 PITPNC1 MYLK3 C2orf88 CEP128 MCM3AP-AS1 SNORA67 NARS2 OR4N3P LPPR5 PTGER4P3 POTEB SLC26A7 ROR1 MYO1D GLP2R MAP2K3 SLC4A4 RN7SL83P CSNK2A1 PMS2P9 AKT3 CRB1 GULP1 TRIM5 KCNS3 SLC5A8 FLVCR2 STEAP2-AS1 MEDAG FAM230B TTC26 PSD3 ABCA13 CHRNA7 HOMER2P2 MEF2C PSPH IGHVII-31-1 EFTUD1P1 SDAD1P4 RPTOR LPP VWFP1 SNORD115-48 LINC00839 MNAT1 RAP1A IQCK C8A TEX36 KCNE1 RAD51B PTPRE ZNF248 PTK2B TSPAN3 FBXW8 DUSP22 FAM95B1 SH3RF3 TPP2 STRADB MYOM1 PSG8 IL21-AS1 DIAPH2 RNU6-58P PSMA1 BCO2 CLDN11 BMPR1A STAG2 ABHD4 DUXAP8 FBXO27 SLC16A7 HTR2C CACNA1E GLRA2 DDAH1 RNF17 TMEM161BP1 PRAMEF26 FAM102B PIBF1 SNORD115-20 MIR3667 TMPRSS11E GABRR2 PIK3C3 BNIP3P3 SNORA40 CHD6 FGD4 MACROD2-IT1 ZNF721 SGIP1 B4GALT4-AS1 HIRA RN7SL801P AEBP2 CORO2B OR5BL1P SLC7A14 C7orf76 TMEM74 RBM19 ENAH IL1RL1 ZNF765 GPR64 TAC4 MYO16 ZNF366 ESRRG ISPD PTGFR SNX31 GRHL2 PSMC6 FAR2 FAM171A1 TRAT1 ARHGAP42P5 ZNF595 CNN2P12 NLRP13 HECW2 FAM230A ABCA8 PEAK1 SLCO1B7 KCNQ1OT1 EYA1 MAPK14 TMEM132C MIR4477A CDH18 SNORD115-21 P2RX6 FRMD6 UNC79 SPON1 STXBP5-AS1 LNP1 SNORD115 SUPT3H KIAA1244 MAML3 GABRB2 MAN1C1 GRIN2B NLRP7 DMBT1P1 PRR5-ARHGAP8 GPR176 EGFLAM PLD5 RNU6-538P PWRN1 WDR4 UACA TSNAX TAS2R1 IGHV1OR15-6 PCDHAC2 LNX1-AS2 LINC00473 CDH6 GABRA2 MDM1 DUX4L9 USP53 ACSL5 LATS2-AS1 DOK6 RN7SL449P FCHSD2 hsa-mir-4528 ADCY8 LINC01122 HERC2P4 TTC7A RNU6-978P PPIAP1 ITPR2 SUN2 SNRPGP9 LINC00158 FRMPD2 OMA1 BCL2L13 RPL30 IGSF5 SULT4A1 SIRPD LEMD3 CHODL FOXD4L4 KCTD9 MSANTD3-TMEFF1 ACTN2 NEB KLHL32 CEP164P1 BCL7C RNU6-278P CACNA2D3 ZNF429 DRD5P1 CLDN12 PRKCE DTD1 CCDC149 GLIS1 ELOVL2-AS1 DPT RGS5 ACTR3BP2 SPAG17 EPHA3 CHCHD3 LATS2 TMEM51 CC2D2A ST13P15 LRRTM4 PTPRN2 CACNB4 USP6 INPP4B KLF7 MYO3A DKKL1P1 AKR1B1P1 PYGL FAM65B GRIK1 MTA3 FOXD4L2 NUBPL SOX5 KIF6 OR52H2P PABPC1P5 MIR4519 DENND1B EFHB NBPF10 FGF14-IT1 ACSM2A LINC00544 CYCSP41 C5orf66 DHX57 TDRD12 SNORA8 SLC12A1 ENTPD4 GTF2IRD2 KRTAP8-3P TMCO4 BMS1P12 PAMR1 DIAPH1 NGLY1 U6 IGLC1 ENTPD3-AS1 LDB2 MYRFL IGF1R CYP4Z2P TATDN2P3 WDR72 PPP1R26P5 C21orf62 NLGN1 DNAH9 TMEM260 ARHGAP42P3 SHISA9 OR9Q1 SHROOM3 CYCSP32 MSI2 LRRC7 LINC00458 LINC00221 CPHL1P ANKRD29 DEFB127 SPECC1L-ADORA2A LCE6A KIF18A CBLN4 LIPE-AS1 C21orf91 KCNIP4 CFTR CAMK1D FLRT2 NALCN-AS1 CCDC144NL RAD51L3-RFFL EFCAB6 ZNF402P RNU6-56P C2CD2 TEX26-AS1 RPS20P5 NIPA1 ZFP64 EMCN-IT3 SYNPR KIF13B MKRN3 ITFG1 IMPG1 C22orf34 DSCR4 GLB1L3 CYP2C8 CD96 RBMS3 SNORD56 FAR2P4 OFCC1 CSNK1G3 TMOD1 NHSL1 SMG6 ITGA11 GAS7 CIT CLEC16A ZNF607 HERC4 HTR4-IT1 KCTD7 ZNF98 ST3GAL3 MIR4307 TCF7L2 ITGA9 RN7SKP96 GBA3 RPGR CES5A PABPC1 TMEFF1 STARD9 HSD17B12 PCSK5 CTPS2 LINC00537 NDUFA9 EGLN3 HS6ST3 CUX1 RN7SL456P CDH12 TMEM132B SLC25A15P5 ENPP7P10 FLG-AS1 SV2C ZNF490 GMDS RGPD1 WDR52 CNIH3 DOCK3 ANKRD62P1-PARP4P3 LINC00702 RPL39P33 FOXO3 LARS2 TRIM59 HIP1 ACTR3BP3 TRPS1 MFSD9 NXPH1 PHF2P2 LINC00871 MYO18B TNR PPP4R4 CDKAL1 PRKCQ-AS1 VAV3 INPP5A KCTD9P1 VRK1 CNTN6 CLIC6 ACSM2B ZNF420 SCN9A CDK2AP2P2 APP MS4A14 BNIP3P2 LINC00470 MAMLD1 RANGAP1 CCDC88A RAB7A C10orf11 MIR663B C5orf17 RNLS NF1P6 ANKRD20A7P RAB2A HPSE2 PLCE1 TACC2 WASF1 ATP5BP1 ANKRD36B ADAM12 ITPK1 FAM214A NT5DC1 CUBNP3 ATP9B MITF PEBP4 RNU6-10P RAB3IP CEACAM6 SPECC1L

		ZNF208 GCNT1P1 KIAA1009 FRY CXADR POLR3K PTPN20B DKK2 GGT2 LRFN5 CDC14A PALLD TRAV24 CECR2 hsa-mir-490 IPO7P2 RHOC GHR SPTSSA BCLAF1 KIAA1715 WDR59 C10orf120 RPS15AP3 RASGRF1 FRMD5 MAGI1-IT1 ULK4 RNF217 UBR3 RNU6-1225P DLX6-AS1 RBFOX1 KIR2DL4 STARD13 SCP2 ASUN ALCAM DENND1A PDZRN3 TMEM117 GUCY2C ADAMTS16 TASP1 NIPBL POTEG HNRNPA3P14 ZNF114P1 DEPDC5 RGL1 ATG10 NKAIN3 INO80D ZNF728 SLC25A21 PALM2 EPHA1-AS1 RNU6-316P PLD1 CD300A PKHD1 NEBL RARB CCDC18 COG3 CBFA2T2 LINC00993 MYH13 PKIB MYH1 MGST1 NAALADL2 FRYL AP3B1 AGGF1P3 MMP20 MIR4533 PHACTR1 MLIP ZNF583 LINC01058 EML5 SORCS2 C5orf47 PRIM2 OR4C9P OR4H6P DISC1 DZANK1 RN7SL141P MAP3K7CL RALGPS1 PMS2P11 ANKRD62P1 SEC63 GC SNTG2 C21orf49 LINC00113 PCBP3 BFSP2 LINC00971 FRMPD4 ATP8B4 LMCD1-AS1 COL23A1 NSD1 LINC00639 FAM3B KCND3 ITGB1 PSMC4 RIMBP2 RABGEF1 MEMO1 GAB4 RNU6-617P DISC1FP1 OR4A9P MAP7 TEX9 BARD1 GRIA1 FAM19A1 TRABD2B UBA6-AS1 ANKHD1-EIF4EBP3 SNORD115-23 STK3 CHN2 ZNF781 VN1R53P NBEAP3 POTEKP LCE2B MSR1 USP18 MED15P7 S100B NRG1-IT1 ESR1 GCNT2 GABRG3 ZNF767 SLCO1B1 ZNF790 EDA2R TNFRSF19 FAM107B LUZP2 SIK2 ERC2 PRKACB RIMS1 CPB1 MIR4798 ENPP2 DOCK4 KCNMB3P1 VPS53 SLC5A3 TRIM51 OR4K4P FRMD4A ZNF965P MCTP2 CERS3 WWOX EGFEM1P ABCA12 PCSK2 KCNIP1 TMEM178A MARK2P8 KRTAP19-10P CNKSR2 HMGB3P20 SNX29 SNURF SAMD5 BEND5 SSBP2 BMS1P18 COLEC12 LSAMP RPH3A KCNQ1 GREB1L PSG7 TEAD4 TNRC6B IGSF21 SLC38A6 GLCCI1 USP40 LNX1-AS1 MIPEP UCHL3 IPO8P1 GSG1L NR3C2 SNORD115-47 MTUS2 EMCN TMEM178B TTC40 ARHGAP32 ADGB LINC00276 EFNA5 NOL4 GTF2IP3 NXN PM20D1 CMSS1 PHC1 CACNA1C-IT3 BPESC1 FAAH2 KLHL1 TEP1 NF1P4 ATP9A RNF216 DMXL2 STIM2 POTEF NF1P1 SNORD54 C8orf46 C10ORF68 SEC24B PPFIA2 IGSF11-AS1 ZNF268 ENOX1 PRIMA1 AKAP6 NKAIN1P1 ANKRD26P4 PTPN20A LST3 ANKHD1 LINC00189 TTC8 POU6F2 SNORD6 TENM3 LINGO2 DIAPH2-AS1 OR52N5 ATF2 CEP85L ARFIP1 PLSCR1 RNU6-156P PHACTR2 ZNF72P RBBP8 RNA5SP283 KCNJ12 OR51AB1P STARD4 MTAP NSUN6 PDE7A GRID2 CYBB SPTA1 ZNF483 ZNF573 RALGPS2 FAM27E4 SNRK SUZ12 MACC1 ZNF415 LHFP CAMK4 SERPINA9 GALNT14 TMEM189-UBE2V1 RNU6-721P VWC2 ANKRD20A17P GUCY2F CELF2 ATP8A2P1 HHLA1 RBFOX2 PDE4B MIR5095 DDX10 OR4C2P ZNF26 SMTN TMEM108 CKS1BP5 LRRC20 MIR3182 LPPR1 SHROOM4 NAV3 IGHV3-29 MX1 ANKRD13A ZCCHC11 KIAA1211 IMMP2L DNAH3 ATXN1 ACTR3BP5 MIR548W AK5 ELAVL1 IGKV1OR-1 BNIP3L PRKCH GALNT8 HAPLN3 OR6N1 RHPN2 ANKRD30A ASXL1 LCE2C SNORA70C TSNAX-DISC1 FOXD4L5 C14orf64 NSG1 PLEKHG1 ANKRD20A8P KCND2 NSUN7 NHSL2 SLC24A3 SOBP KLF13 MPRIP GRIA4 MARK2P9 TMPRSS12 ZNF91 PATE4 PMP22 CNNM2 LOXHD1 FAM108A8P INADL PLEKHA5 OR5AK4P IL18R1 TTLL5 BNIP3P7 TSHR CCDC7 EPHB2 LPHN2 PLAUR BMS1P17 RNU6-1293P TEX11 ESR2 SYT16 RALBP1 ZCWPW2 ADARB2 SATB1 IGHV4-31 C14orf177 ST20-MTHFS COMT ROS1 EFCAB11 NRG1-IT2 AGO3 RNA5SP405 ZBED5 MCTP1 RNU1-51P RPL10P3 PRTG ADAMTS18 SEPT14 SOS1 CCDC59 RNU6-1320P TC2N KCNQ5-IT1 LGR6 SYNE2 DOCK2 BAI3 SDCCAG8 EXOC5 SLCO1B3 DSCR8 PTPDC1 PPM1J NRP1 TRIM22 MDGA2 SAMD3 RFC3 CROCC ASCC3 BASP1 ACYP2 GRM8 ARHGEF7 PHACTR3 7SK PTPN11 ZNF141 CHODL-AS1 COL15A1 PPP1R26P2 EVC2 LINC01019 FAM27E3 BDNF LDLRAD4 ACBD6 CCDC26 DSCR4-IT1 MGAT5 TSPAN8 CADM2 RNU6-469P BDKRB2 PACRGL CDKN2B-AS1 TUBA3C KIF16B CASP8 SWT1 IGLJ1 CDH2 CECR7 DPYD-IT1 FLNC CCNG2 TENM2 DSG2 CEA TANC1 JAG1 PAPPA EVI5 VPS41 SYCP1 IFT80 LINC00879 ZNF527 DYM GOLGA1 ANKRD20A18P GPR133 FSTL4 FAM108A10P PFN2 ACTR3BP6 WT1 OR4C5 CYP3A54P STK38L KSR1 RALGAPA2 CBLB FAF1 GABRB1 SGSM1 TPTEP1 ST8SIA1 SH3KBP1 RTFDC1 SIRPB1 CADPS RNA5SP222 PRB2 ZFP30 MTND5P5 BEND7 FAM212B STXBP5 RN7SKP139 SLC44A1 FAM196B SULT1C2P1 SPRED1 BMS1P9 NPR3 SIPA1L3 MROH2B GALC LIPA RNU6-1269P MRPS22 GNG4 TMC1 LINC00393 CDH26 AGPAT4 NOX4 PDS5B WDFY4 RNU6-157P SNORA32 STK33 DIRAS2 ABCB5 FCF1P9 OR4Q2 DPY19L2 FTLP15 NCOR1P2 CYYR1 LINC00559 KIR3DL1 NEK2P2 SETD5-AS1 SNORD115-24 GLI3 PPIAP27 LRRC9 GRIA3 RNA5SP488 GOPC NTRK3 RXFP1 LINC00457 POLR3C SGCG FBN1 NANP LONP2 HYDIN AGGF1P4 LINC00418 CPPED1 GAB2 DIS3L2 C21orf91-OT1 LINC00922 ANKRD31 EFHC2 PDE1A TMPRSS2 OR10R2 MS4A6E LINC01036 OR4H12P DUXAP10 HMGA2 HHAT TMPRSS15 MTATP6P1 CREB5 RPA3-AS1 PSPC1P2 CLOCK CPA6 MIR105-1 CPNE8 DACH2 DEFA3 ZC3H13 KIR2DS4 KCNK2 SORCS1 TBC1D4 SYT1 OR4K1 TMEM45B SCAPER CDK2AP2P3 OR4K13 NPHP4 IKZF2 IGHV3-32 PPP1R12B SACS AFF2 MIR650 PLXNA2 ZDHHC15 MGLL THAP7-AS1 SCFD2 RNA5-8SP5 RORA CYP4X1 REXO1L2P SHISA6 NFASC OR52U1P IGKV2OR22-4 METTL9 ATP5O LINC00923 snoMe28S-Am2634 MAP2K5 RPS20 LOXL2 LINC01146 IGHV1OR15-3 BPTF ZNF229 NPEPPS SLC2A12 GRIK1-AS2 PLXDC2 RASGRP1 KIR2DL3 KRTAP9-8 NDUFAF2 KREMEN1 SNORD116-26 SNAP23 STXBP6 ANXA8L1 CYP4Z1 LINC00664 DMBT1 NLRP2 FLT3 GALK2 NELL1 ANKRD30B PCDH19 STX12 FAM46A RNU6-368P MCM9 GALNTL6 PXDNL FIP1L1 DOCK10 PRB1 CTXN2 OR11K2P TMEM173 PPP4R1L FNDC3A KHDRBS3 TPK1 MICAL2 ATXN8OS MTHFS LGALS9 GBP6 DRD5P2 OR5J2 MICAL3 TPO ETS1 GTDC1 TMEM131 BICC1 DISC1-IT1 HSH2D KCNK13 DDC YME1L1P1 RNU6-614P WFDC11 NPSR1 SNORA25 POTEH-AS1 WSCD2 SNORA70 MTMR2 KCNH5 RRM1 TSPAN9 PCCA MS4A7 ATF6 PRKAR1B FAM189A1 VCL IQCJ PPFIBP2 POTEH DTX2P1-UPK3BP1-PMS2P11 ZMYND11 RGS6 ZNF525 NCALD EFCAB2 CACNA1C-IT2 OXSR1 SARDH PSG6 CCDC146 MIR4273 FAM221A SLC39A11 RNU4-45P LINC00343 KCNE2 UNC13C RIMS2 TMEM55A IGHVII-28-1 SYT17 FOXP1 SLC5A1 C16orf95 STAC IGHVIII-25-1 MIR4300 KCTD16 EYA4 DEFB122 PARP4P3 NOVA1-AS1 IGKV2OR22-3 ARL15 SPATA13 CEP152 RNA5SP518 RARRES2P2 CST2 OR4N2 NUMA1 RFX4 AGAP1 SNORD115-19 ZNF112 CTNNA2 MYO5BP2 OR8L1P CYB561A3 CHN1 PRAMEF12 ETV6 HGSNAT RAG2 LCE2A FMNL2 RNU6-458P CEP112 KCNJ15 PARD3 CAST LGR4 SYT9 FANK1 LINC00534 VSIG10 DLEU1 GPR39 CASK PXK FAM160A1 SUCLA2 SLC35F4 ZNF562 SPRR2E DNAJA1P4 SEMA3C GRAMD3 ARMCX2 DPY19L1 LMBR1 ZPBP BCKDHB OR4K11P VPS13B ADRBK2 C3orf67 SNORD115-25
HEK293T-hg19-4020 K562-hg19-	181	ZHX3 APBB2 EPB41L4B ACSM3 SLCO2B1 DNAJC15 C2CD3 ISM1 VAT1L FAM19A4 A2ML1-AS1 C8orf44-SGK3 THRB CD53 HS1BP3 ZDHHC11B OAS1 NCK2 C18orf64 CR1 GRIN3A

1289		*ABCD3 DCDC1 BACH1-AS1 CARM1P1 ABL1 SLC24A4 DDX21 PAN3 EVA1A DNM1P32 SLIT2 RAPGEF4-AS1 CYP4B1 RAP1GDS1 BAIAP2L1 PTN TRIO SLC15A5 TFDP2 EML1 TBC1D12 SND1 DPYD-AS1 MTND1P12 CDC27P1 TCF12 SLC47A1 TP73 TMPRSS6 ANTXR2 ALDH1A2 WFDC10A DPH6 OVOL2 PRUNE2 ELP3 IGHV1-3 KIF3B CTBP2P5 RNF213 CNTN3 IL1RAPL2 ZDHHC11 MS4A1 UVRAG TXK KDM4C ACSM1 CTBP2P7 LINC00937 PRSS23 SGMS2 ZNF626 TTC6 QSER1 KIAA1328 OFD1P13Y LINC00298 FKSG68 SAMD9 OFD1P12Y CELF4 CXorf30 SMEK2 KCTD1 CHCHD2P4 ADCK1 HCN1 A1CF CCDC57 ABHD2 ZNF331 MICU1 MBD5 C6orf183 TIAM1 GUSBP1 DGCR5 KSR1P1 DENND2A PCDH11X TRANK1 ATP6V1E1 PTPN9 BMS1P16 LPHN3 WFDC9 SLC9B1P1 ARHGAP20 SLC27A6 FLT1 ISM1-AS1 SLC22A23 LINC01049 NAMPT DNM1P50 HS1BP3-IT1 FRG1 DNM1P28 LINC00299 BAZ2B TUSC3 TMEM38B KCNJ3 NPLOC4 LINC00466 PDXDC1 FAM228A RNU6-16P DGCR10 SLC6A17 STT3B PRCP METTL8 PHF3 CACHD1 C2orf27A MAGI3 TMEM261 CACNA1B NEK7 ZNF585B PSMB2 TPTE2P5 HS6ST2 SPATA6 REV1 FLNB POTEA HNRNPA1P58 TRIM9 PLEKHH2 NIPAL2 RAB31 TBX15 BMPER ATF7IP ZNF558 LINC01141 MGA CEP97 OR11H13P KIAA1671 CNTNAP3 ATP8A1 CELSR1 LINC00271 FLNB-AS1 SERTM1 LINC00707 KCNC4 RAB30 MYH8 FREM1 CCDC73 HSPG2 ATP10B MARCH1 TUBB1 C8orf44*
hESC-hg19-4020	2611	*CASC20 IGKV2D-14 FAM153A MTERF SALL1 PTPRR MIR4329 ACOT12 SLC10A7 ENO1P2 EEF1A1P10 OR7A10 SLC6A11 RNU6-322P TOP1MT MYO9B FAM134B ITGB1P1 SIRPB2 ADAMTS7P4 TRIB3 ATP5G2 KBTBD8 COL4A5 OR5L1 AK4P4 ZEB1 MIR582 CYP4F23P CT60 COX15 SLC15A2 HEPH MMS22L ING3 CCT8L2 MAP3K3 OR2V2 TBC1D3H IGKV1D-13 OSBPL8 TCEB1P28 SERPINA12 KNDC1 IPO9 CYP4F59P HERC2P2 SLC25A14 LHFPL2 PTPN22 CMPK2 RUNX3 KRT18P16 ANXA3 RN7SL308P LRGUK TRPM6 CRHR1-IT1 TERF2IP GTPBP1 Metazoa_SRP FAM170B-AS1 PRDM2 ZNF83 PDCD4 ARL14EPL RRN3P2 RNU6-385P ANO5 C16orf45 GFPT2 LINC00261 ABR NCOR2 IGHV1-12 TM4SF4 AP3M2 MAOA NDUFS5P2 BPIFB2 GYPC EPB42 LCOR RASA4B HSPA8P16 HS2ST1 PREP SNORA2A MIR1273G HCRTR2 RNU6-310P RPL15P21 RNA5SP266 FAM198A SEC14L1 METTL25 ZNF284 CHFR NDUFS4 THADA PIK3CB TP53TG3C FAXC ZNF35 XRN2 RPL3P5 CD6 PTGES3P4 ZBTB11 EXOC2 MYLK-AS1 TACR1 CHST6 KDM2A MED27 NCAM1 RN7SL42P AHCYL2 RPS3AP34 ATF7 KDM4B C17orf85 IL6R MORC1-AS1 GCA IGKV1-33 EPHA4 RPS2P24 HIGD1AP14 L2HGDH PIN1P1 LZTFL1 PRR23D2 DNHD1 BST1 HCFC1 NFYA RERG-IT1 MYO16-AS1 SLC3A1 NADSYN1 DEFB125 KRT18P3 MYEOV2 ARMC6 OR4S1 PPP1R37 EMR1 ACO2 TAS2R3 ELFN1 IL23R SH3PXD2A SPINK5 IGHV3-13 GRPEL2-AS1 UNC5CL FXYD2 IGKV1OR2-108 JPX RIC3 PPM1H RN7SKP1 ZMIZ1-AS1 TRAV34 VMP1 UGT1A13P MTMR6 ENTPD7 CENPJ TNRC18 FHDC1 FABP5P11 SVIL CADPS2 C5 RN7SL168P ATP2B2 TMED7 NBPF13P IGLJ3 ABCC12 RELL1 FAM177A1 TAB2 EIF3D GPATCH8 DDI2 KIF4A ZNF586 SNCAIP XKR9 UGT1A8 SLC38A9 HIST1H3E SNORA18 POTEJ TARSL2 C1orf115 LINC01028 RNA5SP352 DCAF11 GTF2H5 SNORA26 SULT1C3 HBP1 SDR16C5 XPO4 FAM131C VN1R42P BDP1 LINC00422 EMR2 TSPAN19 LGALS4 LPPR4 NRAP SEPHS1 TRIM63 C10orf53 BCL7A RPS24P6 GTF2H2B PTCHD3P1 CLK3 NPIPB5 ELF1 PHBP3 PARP4 GALNT9 PRSS37 NOTCH2 OR51N1P STARD13-IT1 ADD1 OR51B3P ZNF136 KDM6B TTC28-AS1 RN7SL831P CLDN10 USP43 SRGAP2C OR11H4 PEF1 ALG9 BTBD10 HERC3 HMBOX1 FAM122B ITK LINC00882 SLC48A1 ARHGAP26 NPNT PTPN4 EPB41L3 LAMA1 MORC1 SHANK3 KANK4 RNA5SP238 CYCSP4 TRIM51GP MUS81 CPO OR1B1 VWA3A RPAP1 MARK2P13 VAV3-AS1 SLC35A1 SCGB1D2 MIR21 SH3GL1 EIF3H LINC01111 KIF5C DUSP10 BLK SNORD114-23 GPM6A FARS2 CDH20 CA12 PPP4R1 TBC1D3 ZC3H14 CNOT2 MAN1A2 SNORD113 MROH5 CTDP1 DDX50 ZNF75D STYXL1 KRT86 GALNT10 GPR110 RPS12P27 KRT18P56 SLC25A24 C20orf112 ACER3 TRPV1 AVL9 FER1L6 LINC01140 TRIM24 MIR5192 WBP2P1 TBC1D16 SLC22A5 MIR3145 ATL1 SEPT7 RNU6-774P SERPINB11 ALG1L6P NDE1 MIR770 FAM87A RNU6-790P FAM20B FIG4 SEPT7P4 CST4 RNU6-116P ZNF84 THAP4 MARK2P14 MIR641 GLYCAM1 RPS3P7 LINC00964 RPS20P10 PCDP1 RN7SL38P LALBA MIR3122 BATF2 CRHR1 SLC35F5 LPAR3 RNU6-862P KRTAP1-3 IFIT1B DNAH7 FMR1 C6orf89 C7orf50 CR1L SLC12A3 UBN1 SLC5A7 FAM153B SLC31A1 PPP5C ZNRF2P1 C9orf153 RNU7-136P ADCYAP1 TMEM182 CEP290 ANXA10 CENPIP1 FAM171B DCAF4 TSKU VN1R21P SMURF2P1 GMDS-AS1 VN2R11P RAET1L DCAF13P3 RFPL1S SAMHD1 ARL17B RPL30P10 RPSAP52 SIK3-IT1 RNU6-811P SH3YL1 PADI4 DYNC1LI1 TRBV3-1 PRAMEF23 OR2C1 THSD7A KLHL15 FAM207BP LHX4 GNAI1 AKT2 EGFR LINC00334 IGKV2D-26 ZMAT1 SLC10A6 CLASP1 MRPL13 PLGLB2 MAPKAPK3 PAPOLG CDK13 DZIP1 MIR552 TAS2R62P TBC1D3G GTF2IRD2B LINC00589 MXRA7 UGT1A7 RPL36P18 APOOL PSD4 HS6ST1 YWHAEP7 PSMD6 OR10H5 SNORA34 AKAP3 D2HGDH MPP6 DNAJC5B EIF3J TEKT4 OR5G5P C5AR2 RNU6-345P BCKDHA F10 TMEM39B THBS1 MOCOS SLC16A4 SRGAP1 C8orf31 SPATA18 CALCR CDC45 MKL2 C16orf72 CSN1S2AP FAM184A LRRCC1 CCT3 ZNF121 OR13A1 MTHFD1L CMTM4 TUBGCP4 DEFB4B RASGRP3 GAS2L2 USP41 KIAA1239 MED21 TGS1 ZBTB10 MCC IST1 KANSL3 SLC24A1 PRAMEF6 ATXN1L SLC9A4 MTO1 BAHD1 OR5BR1P HIST1H2BA CCDC85C KNG1 TBC1D32 METTL24 C1orf105 GNG7 RUNX2 PGR MSMO1 ZFYVE16 KRBOX1 RPUSD2 WEE2-AS1 MECOM C11orf74 PLEKHA2 CXADRP3 GAGE10 LRRC1 TOMM70A SCN7A PEG3 NADK NLRP8 MCF2 SLC35C1 NPIPB4 FBLN7 POLR2J3 HERC5 OR10Q2P ADAP2 TM9SF3 MIR190A GOLGA6L1 SHPK NUDT13 SDR16C6P C10orf131 CDH7 FGF12-AS1 TOMM5 JUP EMR3 OSBP2 POTEI DAK C3orf58 LRRC18 FAM124A PRDM5 RNU6-684P NBEAP1 RAB11FIP5 C2orf15 IGHVII-26-2 FAM168A NPM3 NPIPB6 ZNF660 ITCH ITCH-IT1 MBP IGKV1D-12 C20orf194 SOSTDC1 CCDC71L TBRG1 ARHGAP23P1 GRP FAM108A9P MPEG1 SNORA33 NDUFA12 FAM205A ADAT1 KLHL20 CCT8P1 NFYB KCNQ3 ANKRD33B AKAP8 KCNC2 CDH19 IGKV1-9 CCBE1 PLA2G16 MIR5708 PGM5 ELMOD3 LINC01006 RNMT MTCP1 MASP1 JAKMIP3 RN7SL691P MROH6 TRAJ17 RPAP2 RARRES2P7 TMEM232 MIA3 RNU4ATAC9P MED26 FAM46C PREX1 ASF1A MARCO GNB1 RNU6-662P CXCR6 IGSF6 RGPD3 SEPT11 RNU6-1065P POLD1 SNRPB2 NIPAL1 ERICH1 STPG2-AS1 ST6GAL1 ZNF404 ADSS SLC25A51 ATP2B3 PYGO1 PIGU TLE4 TSTD2 ALAS2 MRPL23 RPN2 ANO3 HIBADH CRTC1 PGAM1P3 PFN1P11 EFR3A SEC61A2 IGHV3-35 MLH1 TREM1 EFCAB8 SNORD114-22 SNORA72 NBPF14 NXF5 NCOA7 CCP110 RNU2-33P LINC00663 ANKDD1B SLC25A31*

		RNA5SP457 ZNF391 ABCA5 BPI TTC33 KCNJ16 STAB2 RNU6-757P BMP2K TBC1D23 SFXN5 HEXA TMEM30C CCDC126 GOLGA6L17P BRAF PAX5 SNORA48 IL1R1 C22orf43 PCNPP1 ITGB5 VWC2L-IT1 ARSJ XPO7 GPR75 HIST2H2BE WDR73 BTAF1 RBMS1 MLLT4 HECW1-IT1 SNORD114-13 RNU6-279P KCTD14 ST8SIA5 B3GNT5 CASS4 TBC1D19 ERGIC1 MAP4 ACTR3 SNORA56 RNA5SP325 ZNF554 HIVEP1 RAE1 RMDN2-AS1 SOX7 MACF1 SEC24D EPB41L4A SH2D6 ADD3 MBNL2 NDUFA5P4 MALT1 RTDR1 STARD4-AS1 KANSL2 ATXN7L1 FBXO16 FCRL6 WEE2 ZNF473 SNORD116-25 NLRC4 AP1G1 SMARCC1 AMBRA1 STX18-AS1 MYCNUN KLF8P1 OR6C70 RANBP20P IRF2BP2 SOX4 C5orf24 RN7SL375P MSN MFSD6 LINC00355 MUSK HIST1H2AA L3MBTL2 ALKBH3-AS1 PHBP4 GPNMB TRPM8 SLC39A14 RTL1 PTPRZ1 VN1R65P MSH5-SAPCD1 SCARNA10 MSL3 ACACA MAML2 CCR9 SEPT10 SLC35F6 SSX2 RPL9P30 RALY SHISA3 THAP10 DDX27 GJB6 CCDC39 C1orf101 MTND3P6 CCDC141 ZRANB3 RBM33 ZBTB38 OTOR MEIS3P1 FYCO1 RAP1GAP2 OR2AO1P DPPA5P2 PLEKHB1 PELO MIR127 CSTF3 OSMR GAMTP2 REP15 ZC3H3 MIR4293 SKP2 INSC CYP4F10P PTHLH CEACAMP1 UBLCP1 TMCO5A TTLL1 PRKG2 SLC12A6 XRCC6 PPAP2C HUS1B SLC20A1P2 C12orf76 PAG1 SOS2 LETM1 ZIM2 RPL7AP66 ALG14 CNGB3 EMBP1 ESYT3 FAT2 ZNF81 GATAD2A HSPB11 HCG17 ZNF318 FAM135A ZNF382 GOLGA6D ITGA1 FAM194B TSGA10 HAL C3orf20 RNU4ATAC14P MARCH5 CXorf67 MCHR2-AS1 MIR619 KRTAP17-1 TRPC7 AGAP1-IT1 MID1 NBPF5P IGHV1-14 ATP2C1 SHCBP1L AHCY THSD1 PPTC7 KIF4B CNGA1 GLP1R DNAH1 BPIFC ZNF566 ABCC4 STRADA RNU6-967P PLAGL1 PCLO PCNX USP54 ABCD2 SNORD115-31 HUWE1 ARNTL2 SMIM8 POLA1 MROH3P AQPEP MAP4K1 EARS2 NBPF6 PITPNA FABP7P1 BTBD16 ZNF20 RASA4 RNU6-820P CDADC1 ADAMTS19 DPM3 SMPD1 TRAJ16 AKAP10 FA2H EEF1B2P4 ZBED5-AS1 GSN EGR2 WWC1 CPSF3 MYH3 YY1AP1 RNA5SP151 MYCNOS RIF1 RNY4P26 LARP1 CGGBP1 ZBTB16 SAMD9L CARS2 IFLTD1 IGHV3-36 RNU4-79P IGHV3-30 TTC5 CD226 PPP2R5C CAPG OOEP ARSB SNORD114-21 IL34 CRISPLD1 RUVBL1 MAD1L1 TRIM53CP AMPD1 DPYSL5 ANKRD11 RN7SL754P ITPR1 FAR2P3 CPM CDC42EP2 TMEM214 OR2R1P FBXO5 PAK3 LINC00700 CDC42 RNA5SP175 GALNT12 EBF1 TMEM169 RRS1 SNORD5 MINOS1P3 LAMTOR5-AS1 MYO18A CATSPERD PASD1 MIPEPP1 TINAG OR1H1P ZNF32-AS1 CENPA AQR SNORD81 DCAF8L2 ZSWIM6 GSTA7P TTC3-AS1 UGT1A4 OR51T1 EDEM2 LACE1 ZNF630 UGT1A6 POM121L2 CBWD5 FTSJ3 ATG13 OR4A8P DFNB31 ACPP SLC4A7 DKC1 ZNRF2 PRPSAP1 SCARB1 RNA5SP27 F10-AS1 GREM1 NFIC RPS20P20 PRKAG2 IGLV2-34 RFX8 PPT1 NPIPB3 ABLIM2 GOSR1 JAZF1 IGFL2 DYNC2H1 WDR7-OT1 SOX9-AS1 MR1 LIX1 MYO7B HSPA8P5 PPP1R3A AMIGO1 PRR11 ATAD3A PET112 ADCY10 MMP24-AS1 RNA5SP134 CPED1 TRAPPC3L PTAR1 RPS27P22 RNA5SP298 ZNF335 MCCC2 VPS8 KRTAP8-2P ZMYM1 RN7SL399P OR51M1 MVB12B NUP210 BCAS4 FTH1P7 FAM120C ASB15 LINC00519 FAM200B NNT-AS1 CUL1 NCKAP1 RN7SL443P XPR1 C16orf74 ZNF333 IRF4 snR65 GLYATL1 TVP23CP1 TBC1D3F NEK11 RN7SL791P HIGD1AP16 DUXAP7 KIAA1324L LIPM GTPBP8 RPL35P2 LINC01060 PIK3R4 SGPP2 ARMC3 FBXO21 LINC01128 SLC8A1-AS1 TBL1X TMEM150C LINC00624 ZNF155 SERGEF B3GNT3 GPR179 VWDE TPRG1 ZNF32-AS2 PGAM4P1 CAPZA1 CDYL2 SNORA22 ARHGAP18 KIAA0247 ATP5L FCGR1C IGHV1-67 STX17 MSL3P1 BMPR2 MSH5 USP31 NUCB2 SNX27 FRMD1 SNRPA1 OSBPL10-AS1 AKAP1 HIBCH IQCA1 SIRPB3P PIK3CG LPA RREB1 RNU4-73P AXIN2 MIR3675 HCCS MIR1298 MIR488 TAF1 NCAPD2P1 PIK3CA RNU6-853P COLEC10 IRGM SLC9A6 HTT SLC6A5 RN7SL812P CYP2C18 SYK FUT9 IQGAP1 PLAG1 TGM6 PAFAH2 PUS7 CCDC82 NAV2-AS5 FOXL2 CAMK2A PDZD8 SLC25A39P2 SNTN HNF4A-AS1 DDX6 OR10W1 RNU6-848P FEM1AP2 ULBP2 SNORD115-30 COMMD10 SIL1 RNU6-760P FAR1 PURG RN7SL432P EFEMP2 MAGEA6 NMNAT3 FMO5 FANCI FAM69A LINC00342 C20orf26 ACAA1 NCAPD2 WNT2B DCP2 GLYR1 CCT6P1 GIN1 APCS KRR1 SETDB2 HNF4A IGHEP1 TMED7-TICAM2 ACAD10 SSX2B TXNDC9 OTUD5 MAP2 TNFRSF11A FER1L6-AS2 IBTK RPL7P42 CDC73 TRAJ11 SERAC1 NFATC2 RNU6-1032P FGD5 KRTAP20-4 C12orf79 RAB13 FBXW11 S100A10 TDGF1 COPS8P2 EIF2AK4 NOS1AP MTND2P16 DGCR6 BAZ1A TMC3 OTOGL ARID3B CXorf36 ZFAND3 RHBDL2 KIF24 FAM83F RNA5SP26 HMGB1P36 RNU6-831P SNORD114-20 IGFL3 DOCK5 ACVR1B PSMD1 SLC41A3 C7orf31 RNU4ATAC3P MBIP FAR2P2 FAM162A TGFBR2 LINC01099 CDC123 ACMSD GLIPR1 GLRB RNU6-962P HIST2H3PS2 MARK4 METTL4 IGHVIII-13-1 TTC23L AMOTL1 RASAL2 SMG1P1 RGP1 SYNJ1 MMD MIR4527 LINC00927 POLR2J2 PPP2R2B ARHGEF17 IGKV1D-43 JAK1 GPD2 ZFR ACSL6 PARP4P2 HNRNPLL NRCAM ZMIZ1 DAP ZNF462 MCM6 TRIM51BP IGBP1-AS1 COL6A3 FABP12 OR1L4 CNN2P10 GPRIN3 CCDC85A CYP2B6 SLC25A12 OR52E8 ERCC6L TBCK CDC42SE2 PLB1 CKAP5 HPS5 PGAM1P10 ST13 SPDYE2 ZBTB44 QRSL1 RGPD8 LINC00880 GTF2IRD1 SCARNA24 RNU6-273P ZNF613 RSC1A1 COL6A1 EXOC3L1 ANKRD44-IT1 SGCE DTWD1 UQCRHP4 HSD11B1 GKAP1 OR1S1 RNF19A DDR2 CBX5 CSAG2 MIR4687 TRAV12-1 BOLA1 DERL1 ZNF662 ZNF266 NXPE1 NPM1P37 PEG10 PRAME MIR431 IGHV3-37 SRSF5 POLR2KP2 APLF OR11H3P RBMS2P1 MCF2L2 GSDMD LPAR1 OLA1 MAGED2 SSBP1 TFPI2 ERN1 EOGT ATAD5 C1orf116 ZKSCAN7 AOAH FAM231B ANTXRLP1 AR CSAG3 PLXDC1 AP4E1 KIF3C EPB41L5 GAD1 PHTF2 KLHL3 SYTL3 OR10G2 PTPN13 SSX5 TMF1 DAPK1 MAP3K19 DAPL1 PINX1 TNFRSF10D UGT1A3 ATG14 CES1 RNU6-829P UGT1A5 ADD2 PITPNB MTND4P4 IL7 BHLHE40-AS1 SNX18P6 FAM21C SOCS2P1 LINC00310 ZNF788 NADK2 IGHV3-66 TIMM9P3 OR52K2 RNGTT ZNF638 CHORDC2P GSKIP PIGX PYROXD2 ARHGAP5-AS1 IFT52 PSTPIP1 CNBP RNF38 NAALAD2 HSD17B10 NBPF12 DCAF5 GNAL B9D1 LPO MESDC2 KIAA0895L PAIP1 C15orf48 MYLKP1 EGFLAM-AS2 ZNF131 FBXO42 MSH3 SLC16A14 KRT8P9 NSMCE2 TTC37 GPR143 FMO10P LINC00670 HMGB3P4 RNA5SP442 EPS8 ARHGAP21 PTPRG-AS1 HOOK3 ITM2B PAPSS2 CYP2C19 ACSL3 BVES-AS1 GLT8D2 TLR1 PDIA3 USP32P2 DCP1A KPNA3 ANLN SPATA1 GOLGA3 OR1D4 KIAA2018 GTF2H2 NPAP1P6 LAIR1 HIST2H2BB BHMT SNORA15 HECTD2 ALDH2 RIBC1 SLCO2A1 BTN3A1 GPR75-ASB3 ATAD1 UGT2B10 UGT1A10 ZNF25 ATOH8 IGLV3-2 HMGN2P46 OTUD3 FAM163A ENOSF1 TICAM2 TNFRSF10A SLC36A3 SH2B2 SLC6A6 ZNF605 ATP1B4 HMGN2P35 PPP1R9A LINC01081 SLC22A6 SAA3P NPM1 ADAM19 PCK2 DNAH12 SCP2D1 GOLGA6L3 OR51A10P EFHC1 ZZEF1 RN7SL144P FNDC8 RN7SL624P FOXB1 GPR19 TRAV41 HHLA2 CRIPT LTBP1 UQCC1 SLAMF1 KCNE3 PLD6 PPIAP29 ZNF395 RNU1-54P IKZF3 SEC11C HINT3 C20orf78

		RPL7P18 OPRK1 C12orf4 VTCN1 MIS18BP1 HPS4 NOMO1 SNORD114-17 PLA2R1 LYSMD2 RNU7-96P IMMT PICALM C1orf141 SPANXA2-OT1 GALNT18 PBX1 KRT17 RSPO2 MIR5006 TTC21B DGKD PRRX1 LILRP2 C11orf53 RPS6KA6 IL1RL2 IGKV1OR-2 IFI16 BMP6 SCN3A LANCL2 ARHGAP42 CCDC6 MTHFD1 C6orf58 VSTM4 C17orf66 RNU2-47P NAV2-IT1 COG4 IGLC3 FANCA GRM6 AMMECR1 VSIG1 NCAPD3 IGKV1OR2-11 BRCC3 OR7E89P HIATL2 CCNYL3 LRRC42 TRAJ10 ZNF649 LEF1-AS1 RAB9B LINC00352 RPL21P112 MAP1LC3B TBC1D8 SLC50A1 DSE RNU6-902P SPAG9 C6ORF165 ANTXR1 ROPN1 ATG7 RNU6-462P FBN3 SFTPD ZNF442 EPS15 CYP4F35P OSTCP5 WNT5A SPDL1 TRPA1 GNS SLC35E1P1 SLC25A34 WBP11 RNU6-55P RNU4ATAC12P DNM1P34 IL21R REPS2 CCNK EPHX4 CYP2C9 FCAR TRAV26-2 COL19A1 GAD2 MIR5682 HNF4G SMCO4 LRCH4 EXOSC3 FCF1P4 FAM122A AP2B1 ZFP90 CCNT2-AS1 RRAS BMP2 TOX UBE2Q2 EMCN-IT1 IGKV2D-18 SAR1P3 CNFN KCNAB1 PP13439 MAGEA3 NFATC3 EPSTI1 FASTKD2 C7orf72 KCNMB3 SAYSD1 PDE3A RNU6-326P RCN1 TEX14 OR4X7P CLLU1 LIMS1 MIR4435-1HG KRT18P36 TMPRSS11D PIK3CD SLC7A11 KRT18P33 ACAN ZNF780B ACKR2 MRPL11 LCE1C MPDU1 GAST PGLYRP4 SAR1A C3orf70 ZNF223 BCL11B NLGN3 ACAA2 DAG1 GP2 RN7SL258P TSPAN2 ALMS1P FAM91A1 BTBD8 PIP5K1C INTS12 TRAJ19 CHD1L FUT8 TREML2 ZC2HC1A NEK10 WWP1 FILIP1 LINC00621 SNORD111B HIST2H2AC TMEM62 ZNF90 POLR2M BFSP2-AS1 KIAA0556 MYOCD FBXO15 PTGER4 CD68 METAP1 RN7SL324P HSD17B7P2 RNVU1-6 RPS27P29 PRDM1 CCDC47 KCNH1-IT1 AGPAT6 FN3K ARMC2 RERE BIN1 LINC01077 ZNF33B PTDSS1 KIAA1109 MMP11 VWC2L OR10AE1P C4orf45 CNTROB LINC01098 ZNF577 NPAS2 HDDC2 FAM111A IQGAP3 PRSS3P2 BLNK IL19 IRAK1BP1 RNY4P19 LECT1 IGHV3OR16-7 COL5A3 ZC4H2 SLC1A6 LRRC63 ZNF563 BRD7P5 RN7SL53P FAM126A SLC25A2 ZNF528 ZNF44 IGF2BP2 EIF5B OR1D5 ACTR3B SASH1 IL17RD CDC14B PPP3CB IGKV6-21 EMC2 FLCN MIR3663 TBCEL ZNF582 RPS6 HS3ST3B1 C6orf163 TBCE HSD17B3 OR4K16P SNORA59A OARD1 OR4C4P AASS TAS2R42 RNU6-473P CALCB RBM45 CEACAMP5 LINC00290 UGGT2 GNAZ APOPT1 PLAT UBE3B CD99L2 RPGRIP1L PTPRF NRL MYLK CYP4F2 RN7SL401P B4GALNT3 FAM133DP TRAJ13 EEF1A1P33 TRIM66 UBAC2 SNX9 CBWD6 AQP3 RNU1-2 ARHGEF28 UBQLN3 ISPD-AS1 SAPCD1 SLC22A15 EEF1DP3 RN7SKP93 MUCL1 GCOM1 DEFA1 WDR7 SNORD114-16 XIST LRRC37B CEP170P1 LINC00877 RNA5SP79 FEZ1 CTPS1 EHD4 CCDC38 ELOVL2 HDAC8 CBX7 LINC00115 FAM179B ESCO1 PID1 RFC1 ZFYVE28 PIK3R2 GAS6-AS2 KIF27 GZMB ANKRD44 CAPS2 PATL2 OR9I2P XPNPEP3 MAPK13 RSBN1 NOP14 ERO1LB RNU7-150P EIF4EBP1 NTF3 HMGN1P4 OR56B1 VN1R69P PI4K2B KCMF1 C10orf118 ST5 MPP5 BTN2A3P SUSD4 RNU6-1147P FGF5 PHLDA2 RPL7P6 FKBP15 RN7SL482P ERLIN1 OR11H7 KARSP2 RNU6-924P VWA8P1 USP27X-AS1 OSBPL9 SLC25A36 ZNF804B ZNF507 DNM1P17 SUB1P4 CSNK1G1 KRT7 KIF13A MICU2 GLIPR1L2 GPR158-AS1 RN7SL11P CCDC180 TWIST2 SCG3 FAM21FP RIC8B CC2D2B COL6A4P2 OR5B17 TAF3 C20orf203 GAB3 MYZAP PCYOX1L RN7SKP12 STS KANK2 ALDH1A3 TGIF1 ICA1 MIR1912 SPAG1 PLEKHD1 ZSCAN23 PLGLB1 CD24P2 CT64 PRPSAP2 TLDC2 PSMC5 SETD1A NLRP1 RELB UNC80 ZNF512B SNORA31 SPSB4 PALD1 CENPP ZNF32 PRKCQ MARCH4 LINC00968 MEI1 NUDCD3 VDAC1P4 RNU6-443P ATP10D IGBP1 SNORA36A KIAA0226 ACACB RASSF8 CUTC OOEP-AS1 PREPL GHRHR RNU6-272P FCGR1A IGHV3-11 RNU6-1303P IFIH1 XRCC5 POU5F1 RNU1-34P ELTD1 LINC00424 RGS17 EIF4HP2 VRK3 MANBA GSTA5 GFRA1 SPHKAP IGHV1OR16-1 RNA5SP164 GDF1 ECT2L TDRD7 CDKL5 FKBP9L PIP5K1B INPP4A TRAJ18 SBF2-AS1 SIAE AGBL5 SNRNP200 PCNAP4 RIMS4 RSPO3 U1 DNAL1 C16orf97 NLE1 TAF15 RNU6-870P TECRP2 WASF5P RAB18 TXLNB HIST2H2AB RNU6-457P AK3P2 MTND4P16 KRT84 GNA12 ARCN1 CDC42EP1 SEC24A PIGC LRAT SPTBN4 MCMBP DTX3 AMOT PROSER2-AS1 OR2AL1P ETV1 IGHVIII-38-1 NRK CYBRD1 BACH2 ME1 RNU4-68P LEPREL1 HAP1 MRPL30 PPARGC1B CYFIP2 RPLP0P2 RNU6-956P DNAI2 RPS9 LPCAT1 CCDC148 RBM28 FCRL1 ENPP7P6 PGLYRP3 SNORD115-32 AMIGO2 TMEM26 CTGLF12P BCAS3 ZC3H12B KCNJ8 OTOG IGHV1-24 SF3B3 RNU6-143P SLC17A5 LNX1 NUDT21 IGHV3OR16-6 TP53BP2 LINC00592 KCP PDLIM5 ZFAT IGLV3-9 B3GNT2 TMEM63A RN7SL164P ZNF780A RNU6-115P ZNF471 MAGEA4 UCK2 DEFB130 CDYL OR52H1 GPAA1 C11orf16 DONSON MIR137HG C3orf72 LINC01171 MYO1B OR2T7 DCHS2 TAF5L RNU6-804P CALM2P1 RNA5SP338 ARSF AGFG1 ERI1 C2orf73 RNF216-IT1 XRCC6P5 RNA5SP432 TRAJ15 ZFP28 ATP6V0CP2 C9orf85 NETO2 SERPINA6 RNU6-110P RPL36P19 CDK5RAP2 SYNPO2 SSH2 RNVU1-8 NUP210L PRAM1 GK ALPK3 CHID1 ALOX5 IGKV1OR2-9 FBXO8 AMBP HNRNPA1P69 SLC25A26 MTCO2P3 OR2H4P CLDN4 HMGB1P14 SLC22A18 SLC23A2 PKD2 RMDN2 ZNF704 CYP2U1 TRIM43B MAN2A2 ZNF197 SNX18P4 MIR561 C1QL3 RPL23P10 MPP7 FBN2 DAP3 KIAA1958 KRT28 RFTN1 RSU1P1 IKZF1 HIF3A SNORD114-15 KCNA2 LPGAT1 NDUFC2-KCTD14 SEC16B TRAJ20 MFAP3 ZC2HC1B HDAC5 RNU6-376P FRRS1L WDR1 SGPL1 GON4L YWHAE SWAP70 MOSPD2 MARK1 ZNF596 OR52N3P LMBRD1 SEMA3A BTRC USP49 IQCH SLC25A16 EIF2B3 SEMA3E TRAJ12 JTB SMARCB1 MIR433 C15orf43 SLC8A3 FNIP1 MBOAT1 ARID3A MALRD1 UHRF2 MUC7 CSDE1 FNDC1-IT1 RPL21P65 DEFT1P TLR6 TMCO2 AOAH-IT1 CAMK2D ZFP69B MARCH8 IFNG-AS1 CELF6 NTNG2 RAI14 SFSWAP NPAT ZNF716 APOB LINC01155 CMC4 LINC00649 PTPRB EEFSEC CASC3 KRAS ATP7A ITSN2 TMEM72-AS1 SNORA73 NCOA7-AS1 CAPN9 MPV17L ANKS6 IL31RA C11orf58 RNU7-104P ASB3 HLA-DPB2 TRIM55 TRIM43 SCRG1 NRAS KIAA1755 OGG1 WIPF3 MIR374B C1orf21 OR7A5 RAB21 CRY1 RPS19BP1 IGKV1D-17 KIAA1549L FAM133CP ZNF343 TADA3 HECTD4 PPP3CC TGM5 PIK3R3 SNORD114-19 CDPF1 PARP11 CLCP1 ZBED3-AS1 PTPN20CP CCDC109B AASDHPPT OR2T27 LRRC66 COX8C PDXDC2P LDHAP1 OR7E102P IQUB C4orf19 SLC9A7 OAS3 LRIG3 CTSS MUC12 RP1 SUB1 PRPF6 AKTIP LYST AKIP1 RNU7-196P ZNF587B ANKRD46 GAS2L3 IGHV1-46 MYO1H PDE8A EDN3 BLM NALCN TIMM10B CEACAM4 OR11H6 FCER1A GOLGA8A LPCAT2 BCRP9 SNORD116-30 FGFR2 ITGAV IQCH-AS1 ZNF221 CERS1 DDX39AP1 GH2 RBM20 B4GALT6 EPRS SGTB SLC38A8 CHUK IGHV7-27 RAB6B CHAT TNPO1P2 SOS1-IT1 MPP1 CD2AP AK9 EIF2AP4 MYLK4 THOC1 LGSN RN7SL865P ZNF124 KLHL35 TBC1D3C PLEKHA7 SLPI PARP6 SPEN C1orf159 RPS24P17 PSG5 PPP2R2C ZNF217 KRT18P5 SLC6A13 VWF PCDHA14 ZNF644 SMPDL3A TULP4 PAPPA2 MIR4263 WDR78 SUGP1 PFKFB3 WDR49 SIN3A

		TRIM51DP RNF115 KIAA2026 HRASLS5 OR51A9P RND3 CDHR3 TJP1 RN7SL138P IGKV2-18 GABPB1 TYSND1 LINC00635 RPSAP58 KRT16P4 SEC22B SNRPGP14 PTCH1 NEDD1 TINF2 EPC1 OCIAD1 DDX46 THRSP MIR3714 KAT6A BCOR BHLHB9 SLC25A5P8 GNG5P2 C16orf52 FANCF CUX2 RPL3P8 MIR421 TMEM187 DDX4 CDRT15L2 RNU6-95P IQCB1 RNU1-146P ARNTL RNMTL1P1 DROSHA HEG1 RNA5SP237 ALS2CR11 KPNA1 RNU6-959P C4orf40 FAF2 CCND3 ATP5EP2 PIN4 RN7SKP110 EIF2AK3 IGHV1OR16-2 SIGLEC6 PRAMEF11 EPHX2 LMTK2 MTPAP UBE4A TRAV16 RNA5SP361 IGHV1OR15-1 NDUFC2 C7orf13 SKP1P1 PHLPP1 SLC4A10 BPIFA4P SIAH1 SAP30L-AS1 WARS2-IT1 GOLGA7 MTND5P31 GLYATL1P1 KATNBL1 ZNF45 CYP4F9P FAM183A UHRF1BP1L SNORD114-14 APIP MMP28 CCDC84 ELMO2P1 PDGFRL MYLIP PHIP HMGN5 ISCUP1 RNU6-1259P ANKRD16 ECM2 ARHGEF37 OR51A2 OR8B9P BRAP ADAM6 MIR3194 PPARA CD244 MIR3943 LINC00609 HMBOX1-IT1 MYH15 DENND2C EXOSC3P2 RPL31P63 DEFA7P SLC5A12 UGT1A9 TG DGKG ATP8A2P3 CAMTA1-IT1 TRBV4-1 GPR137C BTC TRIM51JP PPEF1 LINC00899 DYNAP KLF8 DCAF13P2 SMARCD2 GPR27 TRIM21 SBF2 NFIB T SORBS1 GUCY1A2 IL37 PACSIN1 TBCA LRCH2 RFPL4B TRAJ14 RN7SL86P GSTK1 ZDHHC9 TSEN2 MAP3K2 MIR3198-2 ANKRD13C TRAV9-2 C2orf54 CYP4F62P FGF9 RPL23AP53 SLC1A4 PLA2G15 LIMCH1 SSH1 C4orf21 ENSA HOOK2 INTS9 CCDC144B LINC00491 LINC00461 OR4A17P VN2R10P ESF1 ANAPC16 ELOVL6 TMEM71 ZNF14 AP4S1 KLHL22 COL14A1 MICALCL RECK POT1-AS1 RAPGEF3 MIR136 USP34 OR7E161P LTF TGOLN2 EDC4 MRGPRX3 SLC25A33 SLC30A6 KCNU1 CEACAMP3 TMEM231 ENPP7P3 TRRAP MIR4274 LDLRAD3 NIPAL3 SNORD114-18 CATSPER3 PTPRA CEACAM7 DST MSH2 LNX2 MS4A18 UBE3D C6orf165 IGLV2-11 LINC00359 MGAT4A KIAA1468 ZNF180 USP48 NR1H4 NAA30 CADM1 RAD17 TMEM17 TEX12 IGHVIII-11-1 RGS9 LINC00301 SPATS1 XKR8 OR4C6 YLPM1 KRTAP8-1 GUSBP10 RNASEH2A PAWRP1 NMD3P2 RNU7-99P PRAMEF4 LINC00558 DTL GNA14 SMIM20 PRR23D1 RSAD2 OR2D3 CRTC3 CDH23 KLC1 TTN PLLP NAF1 FLG WDR41 MEG3 BCCIP KRT18P6 ACN9 RBBP5 HBS1L SETP21 DENND4C CD46P1 KIAA0319 C6orf106 MORC4 ETV5 LINC00401 GCG MRPL45P2 MTND6P13 NCMAP HNRNPA3 ZNF667-AS1 IDUA RNU6-496P CAB39L OR5T3 PDZK1 NDUFAF4P1 PTPN3 NOP14-AS1 SEPHS1P1 LINC00437 RPS3AP39 MLNR RNA5SP355 PAPLN SLC9A8 RNU7-31P RN7SKP67 CHSY3 EFCAB13 RALGAPA1 EDN2 NNT HDAC1P1 PRKRIRP7 SIK3 ST8SIA2 DLGAP2 TAPT1 IRF8 ZNF654 ZNF253 RN7SL606P ITGB3 SEP15 BMPR1B ABLIM3 CABLES1 LRRC28 ADAMTS10 ALKBH8 ZNF222 IGHV1-45 LINC00264 MIR432 ATP6V1G1P6 PI16 OR5B19P FKBP9 FBXO18 C2ORF15 TMEM185A SLC25A24P2 SNORD116-29 NDUFA10 NUDT15 CEPT1 MRPS16P3 PRAMEF22 TRAV23DV6 CLIC2 ZNFX1 DNAJC19P2 DCTN5 MIR4715 B3GALNT2 CYP4F30P MGEA5 PVT1 NMD3 BCAT1 RNU6-602P IGKV1OR2-3 C18orf54 DNER TARDBPP1 VPS13C SLC14A1 ALKBH3 SEC14L1P1 UAP1 IRAK4 SERPINI2 TTC9 ATP2A2 APBB1IP CKS1BP7 MIR3126 TUBB3P1 PGM3 MARVELD2 TAOK1 RNA5SP370 C20orf197 PVRL3-AS1 AMMECR1LP1 OR51G1 IGHV1-17 GALNT16 CNPY1 KRT8P13 RNU6-79P PRKCA GLIPR1L1 RNF6 MTND5P6 VAV1 SLC16A6P1 FBXO10 ECSCR PDGFC TP63 IFI44 RPS6KA3 RPL5P14 NPHP3-AS1 SPRR1B FRMD4B FTX CRISP1 MIR1304 OR51A3P SLC2A11 GOLGA8B RAB8A IGLV4-3 IGKV2-24 RNF169
K562-hg19-1289	393	GPR98 EDN1 RPS2P35 STOX1 LRRC6 DDB1 CENPQ RNU7-188P CEP350 SMYD3 ST8SIA4 KIAA1211L SYT13 CPEB3 SLC24A5 C12orf75 RNA5SP347 FAM110C LIMD1 PEX16 NOP58 TMEM161B-AS1 CAMSAP1 PSMF1 CYP4A22 MIR4509-3 MTG1 TTC13 ADAMTS9-AS1 ATP5LP7 PSKH2 CDC42P5 ZMYM6NB METTL21EP NPIPP1 SPATS2L YPEL1 METTL16 NRSN1 SLC37A1 LINC00494 SLC30A2 PCYT1B OR4F7P SMURF2 CCDC129 INTS7 ANKRD18B AXL ZNF317P1 ANKMY1 ZDHHC7 TNK1 PKD1P1 PCDH20 SESN1 SEC22C PRC1 RASA2 IGLV2-5 MUC17 BRDT IER5 RGS22 MOBP AKAP17BP RNF111 RNU6-15P MAP3K4 C21orf33 VSTM2A CAMTA2 ZDHHC21 SLC9C2 ACAP2 LURAP1L PAK4 SLC46A3 CAV2 CD200R1 DEK TBC1D3P1-DHX40P1 RPL30P16 LINC00616 STMN2 C9orf92 UGT8 SETD4 MAPK8IP1 RFX2 ZAP70 TNIK ITIH5 SLC37A3 GINS3 PKD1 CD55 RN7SKP117 NEDD4 KRT18P9 IPO8 SLC26A3 ZNF175 ANKRD28 NDUFAF6 MYO10 TMEM230 OR2AI1P LEPR OR8I2 ZNF502 OR1L1 RPAP3 HMGB3P27 PRC1-AS1 ABCC3 SYNJ2 SLAMF8 SLC45A2 NPIPA1 SLC35G1 ATM LTN1 RUNDC3B ADAMTS20 IPMK AACSP1 RNU2-46P GPR149 TMEM40 AGXT2 MUC4 MMEL1 EXOSC8 WDFY1 CASP3 KCTD20 SMC2 TMBIM7P PRPF4 OCIAD2 NUS1 LINC01151 LINC00383 RPS15A MBNL1 CYP27A1 TP53INP1 XPO1 LINC01105 CXCL12 ST6GAL2-IT1 GLTSCR1L RPL19P13 PIGN PLBD1 RNA5SP249 FAM172A LOXL3 ZNF280B GDI2 RHBDF2 PTPRO AQP1 ANO7P1 C18orf25 XKR6 C11orf24 KLK10 ZNF800 CYP4A22-AS1 FTLP16 HIPK2 TTC23 RNF125 CASC1 PPM1F GRM4 RRAGD SHC4 COL5A1 RABL2A F3 WNK1 ATP5E AGMAT STK38 B3GALT1 ADAMTS5 RUNDC3A ITPRIP ALDH1L1-AS2 NEK3 STK17B C1orf146 IGLV3-4 WDR63 DMP1 CSTF1 ITLN2 PCSK1 SYNGAP1 BET1L PCYT2 CCDC124 EXOSC2 RBPJ PRDM16 PLIN2 TBC1D3P5 OR2B8P ARIH1 CDC6 ASAH2 ARHGEF38 C10orf40 UBL7 MTR ANKRD52 PFKL LCN1 MRPS28 PFKFB1 DIXDC1 MEIS1 GLDN NEDD4L CYP1D1P BEX2 ZNF254 ABCC2 CYB5R4 TMPRSS13 LINC00229 HPGDS INCA1 RNA5SP474 RNU6-620P HMGB1P25 EEF1A1P16 UBE2L3 RPS4Y1 TMEFF2 CKS1BP6 GNA13 FAM217A ZBTB24 ALS2 SMPD4P1 PWRN4 COPB2 GGH COL4A2 PPP1R13L RCOR1 PUM2 ZNF365 CCDC125 C5orf63 GTF2H1 TNRC6A LINC00476 DCAF6 PIP4K2A FCHO1 WNT2 AGAP6 MUT C2orf43 AKAP12 SLC7A11-AS1 FER ROBO3 REG1A RPA3 VPS54 GLS HNRNPA1P35 GLI2 C7orf73 FAM53B ALG5 GUSBP9 OR8I4P KMO UBE2U MED28P3 MIR422A ELP2 POLR3GL POU2F1 GALNT1 RNU6-14P EPHA10 ERCC6L2 RANBP9 ACCSL ZNF440 AP1AR ASAH2C OR6C64P PACSIN2 MIR4509-2 GAPDHP36 NR2F1 KIAA1430 ZNF274 RTN4R CASP10 GAN YEATS2 MYEF2 TAF2 ADAMTS9 DHX40P1 NRP2 DHX40 GNB1L RNU6-53P MGAT5B DEFB107B LINC00529 FBXO40 ARHGAP28 RAB27B LMBRD2 HSPA8P1 GPATCH4 REV3L SARM1 MLK7-AS1 CWC27 ARMC4 IRAK3 VTN LINGO1 TECPR2 DEF6 AK2 TES PKD1P6 CLASP2 PDCD10 PBX3 CMAHP TRAPPC13 BTF3L4 TPD52 PTPRK PHACTR4 SMIM4 OR4F2P TCP11 SS18 LRP6 OR5BM1P CFI UMODL1 C11orf94 RTTN SRPK2 ALDH8A1 GNPNAT1 TRPM1 EPG5 SPIRE1 MYL7 RBM47 GBAS RNA5SP402 MOB1A ST6GAL2 TRAF1 PTCHD4 TGFA TTLL3 ARID4A ACER2 C6orf201 NT5C2 HTRA2 CUL4A C1orf204 SF3B4 XYLB SIGLEC11 TXN TMEM199 ZNF770 MLPH IL2RB VSTM1 DPY19L2P3

HEK293T-hg19-4020	2622	*DDC8 USP17L28 CD44 SAMD4A TRAF3IP2-AS1 RNU7-66P LRRC37A5P MIR4677 PAX7 CMTR2 C15orf27 C10orf25 VPS4A ZNF799 NCF4 CST2P1 RNU6-498P LCORL LDB3 BSN-AS2 FGFR1OP2P1 DLEU7 EP300 NIFK-AS1 RDH10 GPSM2 OR51A8P KLHL14 LRRC71 KCNA6 MIR3156-3 BCRP6 CHD9 UBE2CP4 EPN2 MSRB3 MREG AK7 INIP SLC12A8 NR4A2 PIR RRP7A ZFYVE1 SORL1 TEAD1 CTAGE5 SNORA46 SLC22A2 LINC00698 ATXN10 RNA5SP359 DHRS11 CCDC34 BMS1P11 KIAA1210 TAOK3 A2M ROCK2 RNU1-77P TEK OR5D3P FOXK2 DGUOK SRRM4 FAM73A ARHGEF26-AS1 PYY KCNC1 STARD5 GBP7 SLFN12 OR9L1P CD38 CCNE1 ZNF257 RPL26P9 GPR78 RNU6-132P CORO2A ANGPT4 MIR4499 AMY1B AP4B1-AS1 CDK8 CHST15 CDK1 NLGN1-AS1 N4BP2L2 ZNF232 RPS15AP6 BANF2 FAM90A6P MAP3K13 CLUHP4 LINC00924 SSXP1 SAA2-SAA4 BCL2 MIR654 ZNF239 LINC01047 SPANXN4 OR7E25P BDKRB1 WDFY2 FAM90A3P COL18A1 PXDN RNU6-436P TOMM34 MIR1200 RAC1P5 TOX3 TF PWP2 DNAL4 ANHX CDC42BPA F7 CNOT1 SLC13A4 SAMM50 OR10J6P MIR1185-1 RN7SL52P RHOH ATR IGLV3-25 ADAM20P1 PAQR8 MIR4768 AKR1B15 SNX25 GNRHR SUZ12P SDC2 SARS QKI GAGE2B LAMB4 MAN2B1 PRDX4 AGT DAPP1 CHRM1 HMGB3P24 FUT4 DBH FAM46D OR5G1P CENPU LINC00162 TMEM11 MCOLN2 RPL7P55 PLCXD2 LINC00851 KRT222 UBE2L6 IPO13 SUV420H1 GMFB CCR3 GNB4 IGLV2-23 SALL4P7 RNY4P7 SCN11A ACTG1P1 FAM225B RTCB SORBS2 PCMTD1P3 RNU7-177P MOB1B GDI2P2 C1orf112 HUS1 GOT2 CYCSP34 GRIK1-AS1 TRAV8-7 MIR4789 PLSCR4 STRIP1 MSNP1 KCNK10 PCAT1 CASP5 RNF182 UPP2 NDUFA13 MIA2 LINC00517 MLIP-AS1 PCDHGB4 S100Z ANKRD26P3 VRK2 DNAJC9 ZNF568 LIN28B SIDT1-AS1 ELMO1-AS1 RN7SL16P C3orf22 PLK1S1 PAWR TBC1D3B PREX2 LDLR URGCP-MRPS24 ZNF384 SNORD11 ELAVL2 RPN1 PGM1 ZMYM5 DLGAP1-AS4 OR11L1 DNAJB4 MIR519D PECR PCDHGA10 C15orf60 GTF2IP1 SVOPL EIF2S1 POLR1A TMPRSS4 TSHZ1 LRRC16B RAB5A ARMC10P1 LRRC37A12P ZBTB8OS MIR17HG JRK H2AFZP1 ICA1L RNA5SP470 MIR491 OR8K3 RAB11FIP4 RPL39P36 RPS17P15 FAM49A KIAA0753 SNTA1 PCDHGA5 IL20RA MIR4439 OR9I3P SAFB2 FMO9P DNAJC25 GEMIN8P3 CCDC144A OR52T1P FKBP5 ERCC8 OR11H12 WIF1 CMKLR1 TMOD2 PGBD4P7 LINC00575 SCNN1B SLC44A5 PLCB1-IT1 SDK2 DICER1-AS1 CCT6B KRT74 SPINT4 CLIC5 RNU6-1241P RNF43 TRHDE ZNF677 MAP2K4 CSRP2BP CD72 SDR39U1 MAS1 OR2L13 PPP6R2 SETBP1 ZBTB41 MME FAM213A MAST4 RNU1-142P WDR26 RNU6-723P YJEFN3 DTWD2 ULBP1 MALL RNU4ATAC8P MAPK8IP2 FLJ00273 DPY19L2P2 IGHVIII-2-1 C21orf2 NIPA2 RNU6-554P BZRAP1-AS1 GSK3B PDXP KIF9-AS1 VPS37B PCDHGA4 PCDH9-AS2 C9orf135 MAPK1IP1L PKP2 AMPD3 ADCYAP1R1 SFXN3 SNORA71 PDE6G LRRFIP1 GCLC TUBAP SERPINA4 MTCO3P2 TBPL2 IL18RAP RNU6-164P TRIM16 CDC40 MIR539 PQLC3 MIR4713 PRELID2 MKKS STK39 ACOX3 GOLGA8J RPS6KA2 LINC00161 SNORD116-17 ZNF571-AS1 SLCO1C1 RN7SL678P DEPDC1 GRAMD1C SMOC2 TNRC18P3 IGHVIII-26-1 HORMAD2 MCF2L STK24 LINC00604 RHOQP1 VPS26B PARVG NUTF2 CDRT4 KRTAP10-5 TPT1P2 U8 MIR524 SNORD109A IGHV4-28 EIF4E LINC01162 ATP5A1 URGCP TRAF6 AMY1C CKMT1B PNPLA3 C14orf183 LINC00320 C12orf55 RNU6-1003P KCNQ4 PIK3C2B PA2G4P3 FAM184B ERCC4 EXD1 CTNNA1 C15orf26 RNASEH2C SCAMP4 PFKFB2 HNRNPA1P61 WDR33 RN7SKP233 CHD7 RNF126 RXFP4 C5orf51 ANKH C19orf18 MOV10L1 BIRC6 PTBP3 NLK PCAT2 MDM2 BCRP7 TDRD3 NIPA2P2 MIR544A IPPK OVCH1-AS1 CDHR4 GBE1 SNX5 RASGEF1B DPY19L2P1 PAPD7 MIR154 SNORD115-35 GPR161 OR51B2 NR4A1 TIGD4 SLX1B-SULT1A4 TMPRSS3 ZNF878 UPB1 GIMAP4 KCNA3 ZHX2 C1orf177 SOHLH2 RNF152 TAGLN3 FAHD2A ZNRF3 NFE2L3 TTLL9 PALMD MEOX2 EML4 STK31 ENPP7 KCNG3 TMC5 KIAA1257 LINC00365 HIGD1AP13 OR4K6P TMEM50B KRTAP13-5P ARHGAP29 OR4S2 GOLGA8K RTCA TFP1 APOOP1 TMEM52B CAMSAP2 LINC00970 RNU6-141P ZNF433 RPL7L1P12 PWAR6 LINC01094 DUSP27 TBX3 OR8K4P NANOGNBP2 CHRFAM7A MIR620 RPL13AP2 PAAF1 SLC5A10 CABIN1 TRIOBP A2ML1-AS2 HRH1 MRPS31P5 CHEK2 FBXO47 CYP4F33P OIT3 ZBBX HTRA1 SIGLEC30P GUSBP11 RAB22A HSPE1P19 ZNF160 ZNF688 PWP1 ILDR1 GRIK3 TNIP1 FSIP2 MED15 ZBED4 NABP1 MT1HL1 MTPN ZER1 CLUHP5 MRPS31P4 FAM9A ZNF474 SEC14L2 OR52X1P OGFOD1 ZBTB8B CCZ1 POLE TSPEAR LINC01035 TTC4P1 ZNF559 ZNF559-ZNF177 DET1 SLC7A7 VCAN SLC1A3 MIR3173 RPS3P6 PCDHGA11 RGS12 C14orf119 DNAJA1P1 RNA5SP438 ASNSP5 LRCH1 CHP1 SAA4 MIR3666 TCL1B RN7SKP218 RPA2P1 UBE2E2 SLC39A10 IGHVIV-44-1 PDIA6 PTGFRN RNU7-119P SNORA76 NCK1 KCTD8 FTCDNL1 SPIN3 SH2D4B CPB2-AS1 HS3ST2 MRPS21 ZNF830 MAGEA11 ZNF567 SH3BP1 ERMN MIR605 SIM2 TANGO6 CHCHD2P9 CCNB1IP1P2 GPC3 TLK2P1 PIGUP1 NANOGP4 STAMBPL1 ZNF737 CRYL1 NOVA1 HADHA IGHV3-64 DLG5 PER1 RNU6-46P WFDC10B AIFM3 PCDHGB3 CENPK RN7SL714P SUN3 SLC27A2 PRDX2P3 RPL23AP60 C16orf3 RNA5SP385 TUBGCP5 MBOAT7 POMC NLRP12 MIR4681 TLK2 ZNF2 MIR759 ATP5F1P6 HSF5 PSPC1 TERF1 SNORA16B VN1R91P SPRED2 N4BP2L1 C11orf65 ITGAE ANKRD18A CACNG6 LRIT3 ZSWIM3 IGHVII-67-1 WDR83 COMMD6 MIR1254-2 TTC4 ZNF734P C15orf54 OR10AK1P PCOLCE2 CDC20B IGLVI-68 LINC01143 LINC00347 C9orf3 KIAA1432 SYNM MAPK4 OMG ZNF486 KRR1P1 EZH2P1 CWF19L2 NRG3-AS1 VN2R17P EXOSC3P1 RNA5SP353 MRPS18B AOX1 IGKV1-5 USP17L27 SERPINA5 ANKRD34C ADAM2 NDEL1 RBAK LAPTM4B LINC00472 MT2P1 DIO3OS IFNWP4 ARHGAP11B RPL23AP82 PCDHGA3 THEMIS SDHA COL12A1 MIR4529 RNU6-810P MTND2P25 ABLIM1 GAGE2C RHBDD1 OR8K2P ZNF22 IGHV3-76 HMGB1 FAM174B MAST4-AS1 COG7 IGHVII-22-1 KLF3 RN7SL130P IGKJ5 LINC01013 KANK1 KIF26B UGCG SLC6A2 C1orf106 ORC3 ATRX DMRT1 TVP23C CHST8 RYK BID IER2 TGFB2 CHRDL1 TRAF3IP2 HSPB8 PXT1 ATP6V0D1 RLBP1 CD300C NPM1P41 LZTR1 CAPN3 CTNNBL1 CCDC12 IGLVIV-64 PSMC1P13 ITLN1 TRMT61B DGCR9 RNA5SP487 FAM20A LINC00645 RNU6-321P RALGAPA1P SH2D1A CALB1 LINC00866 SLX1B KRT223P C18orf8 ZNF347 GTF2E1 ST6GALNAC5 KIAA2022 KCNIP3 EXT2 RGS16 URB1 ZSCAN30 TUBAL3 CRYZL1 MECP2 ZNF454 MAP3K5 NOS1 TIMP3 MIR4742 RNA5SP50 RPL23AP12 ABCG1 ZNF299P FAM207CP PLN PRDM9 YOD1 SMAD3 CTDSPL2 C14orf182 PABPC1P12 DGKH CDK12 USP17L6P RNU6-196P NEK4 DCBLD2 PRKAR2A FAM90A13P SYT10 THYN1 KATNAL1 NF1P2 MYO6 EZH2 RBM22P2 AKIRIN2 VCAN-AS1 HIGD1AP2 PHKA2-AS1 BORA PI4KA SOD3 ALDH4A1 TMC2 SAMD13 DHRS7C OR51B8P PCDHGA7 RNU2-5P RNU1-139P TNFRSF11B CD36 MIR3116-2 ARF4 LINC00448*

		SLCO3A1 TNFSF15 RN7SKP162 NCF2 CAPN14 RNA5SP453 FAM90A5P USP16 SLC9C1 KRTAP20-2 GPCPD1 BCL9 UBE2CP2 IGHV4OR15-8 C9orf41 TRAF3 RIPPLY3 PLXNB1 C9orf171 MPPED1 ERICH1-AS1 MIRLET7C PWRN2 LY86 CYCSP51 PDXK LRRC4 SPICE1 ETS2 IFFO2 IGFBP7 NDNL2 FCRL3 CTBP2P4 NMNAT1P1 ERMP1 FAM24B OVGP1 FAM27B ARMC8 RN7SKP253 SNORD115-36 RNF144A USP17L30 C18orf63 RN7SL864P RBMS2 SERPINA1 LINC00376 RN7SKP76 TAPBP KIR3DL2 FEM1AP1 C3orf33 GABRG1 CYP4F31P KRTAP10-1 TRAV22 MAPK9 MIR548U ZNF540 LOH12CR1 CD58 CDR2 IGHVII-30-1 SELE MAGT1 MIR517A ITGB7 CHKB SCUBE1 ZSCAN10 ARHGAP22-IT1 CDH17 GNAT3 OXCT1 RPL5P35 MANSC4 SV2B PDE9A ZNF679 PRR14L DYNLL2 FBXW12 RNF216P1 ABTB2 MORC3 MAGOH2 GPR126 OR4F14P PPHLN1 RNU6-185P CHL1 RAPGEF1 VANGL2 KRTAP19-7 HSF2BP BNIP3P1 PLS1 IGLVVI-22-1 RAB23 FAM8A2P ZNF516 PRLH SLC1A2 KRTAP12-2 CST9L hsa-mir-6080 HPS3 LINS KCNK17 ARHGEF3 RHBDL3 ZNF840 EDNRB CFTRP1 RCVRN LIN7A CYCSP27 CYCSP17 TPCN2 CNN2P4 MPZL1 ZNF578 LRRIQ3 ADAM9 TPH1 SNORA51 MARK2P5 RNU6-1266P MATN3 PKP4 SEPT9 RNASE9 FAM155A-IT1 UOX LUZP4 SIPA1L1 TGFBR1 GBP2 RPL19P12 TTLL2 LRPPRC ACVR1C HEXB RPL34P3 ZNF209P TNKS RPS4XP22 ABI3BP DNAJC25-GNG10 YBX1P7 PRRC2C TUB ARHGEF18 KCNK1 EREG AXDND1 DCAF7 WBSCR17 PCDHGB2 NDFIP2 RBPJP7 RIMKLB MIR4327 KRT126P TRAV8-5 TNS1 SSUH2 RBL2 LENG8 RN7SL516P CBX3P4 IGHV3-30-2 AATF CHEK2P4 RNU6-352P C1orf87 WDR64 GART PCDHGA12 RN7SL99P GRAMD4 PSG1 GJB7 BRINP1 HIAT1 SEPT7P9 RNU6-552P NPM1P31 SHC3 DRG1 SRP19 TRAPPC10 KLHL25 GAGE2D IGLVIVOR22-1 POLQ CNOT10 DYNLRB2 SOAT1 ABHD17AP5 MAP4K5 TCAIM OASL OSGIN2 DARC SKA1 SLC9B2 SHMT1 CRISP3 RPL36AL SRSF4 GABPA SCGB2B2 POU5F1P3 CAMK2G C6orf3 BRD1 PRICKLE2 MIR1290 RNU6-1184P TMEM100 PSTPIP2 LINC00861 SNORD115-12 MIR4490 RNF165 FXR1 DNAH2 ANP32C CASC4P1 ITGB3BP UBASH3A HDHD1 CUBNP1 LYPD6 PDIA5 SUCLG1 TMEM67 OR51I1 WDR47 VDR MIR4452 SLC25A1P2 KRTAP13-6P SPESP1 BTF3L4P1 MIR4695 SH3RF2 SIGLEC26P RPS3AP46 OR4A5 RN7SKP168 CD163 BTBD3 RBM22P1 ACSS1 BBS2 ULK4P1 CLSTN1 IGHVIII-22-2 GAPDHP69 MOV10 EDIL3 RN7SL587P RPL39P40 SLC20A1P1 ZNF337-AS1 MIXL1 RPS6KA2-IT1 IGHV3-16 SKAP1 DSC3 WDR11 MIR487B RNU6-909P CNR2 KLHL33 GLRX3 CCR1 LINC00674 MTND1P2 SDAD1P1 LINC00395 LYN ZNF732 MIR655 AHSA1 MIR941-1 GDAP2 MGAT2 LARGE-IT1 HIVEP2 UBE2R2 RN7SL673P OR9A4 NOL12 CHTF8 SYT6 C7orf66 LINC00446 PRAMENP ADH5P3 GXYLT1P2 IL12RB2 RIN3 RN7SL23P REG4 USP17L29 UBL3 RNASE3 NLN RNU6-49P PABPC1L TBC1D30 NME7 MND1 GTF2A1L ANKRD30BP3 SPNS2 SKOR1 BOLL HERC2P5 RNU6ATAC33P IL1R2 RPS27P27 RUSC1 DCUN1D4 SLC1A1 ST20 RNU6-1063P MIR1267 RNA5SP366 CCL8 PPP6R2P1 THAP7 MAPKAPK5P1 SMC1B RNF144B FAM27C ZNF841 PEX3 ATG4C RBM11 NTN1 ABCB10 LHFPL1 RPL31P40 LINC01107 KRTAP20-3 GAGE12I OS9 SLC4A1AP IGHV7-34-1 NR5A2 SNX30 SNORD112 PGAP3 PKD1L1 TSNARE1 RNU6-803P STRBP HELLS OR11H1 BTF3P14 JAM2 ZNF355P OR4K17 SNHG17 IGHV4-4 RNA5SP125 SRD5A3-AS1 RBPMSLP EPM2A TNFRSF10B S100A7L2 SNORD45 LINC00930 MIR600HG QTRTD1 LARP1B RN7SL743P NPM1P13 SOX2-OT OR4C15 RNU2-27P DYNC1I1 KRT18P2 WRN SDF2 KMT2A RN7SL435P ZNF90P3 XBP1P1 FAM210A TCEB1P32 ARHGAP44 ERCC6 PRMT3 LINC00840 RN7SKP140 DSCR3 IGKV2OR2-2 HSPE1P25 USP17L5 ZDHHC14 DPP8 MIR4764 FAM194A DDX51 LINC00349 SLC30A5 LINC01029 ARHGEF33 RN7SKP99 RANBP10 FAM90A4P CAP2 LINC01053 DGCR6L MATN2 TDRP SNORD115-41 SRGNP1 CIZ1 WWTR1 SNORD17 NFX1 RNVU1-18 PLCD3 APCDD1L-AS1 WSB2 TYW1B SETD7 hsa-mir-3171 CHAF1B RNU6-286P ADIPOR2 RN7SKP238 OVCH1 CATSPER2 GPR125 CLDN8 RN7SL766P EFCAB6-AS1 RNU1-131P NES TBC1D2 RNU3P3 MUC5AC CLEC4A LINC01010 PRR12 KCNMB2 SLFN5 SNORD74 TIMP2 LINC00911 TECRL GTF3C6 SNX24 MORC2 OR11A1 UPP2-IT1 FRMD6-AS2 CPSF1P1 PTPLAD2 HNRNPKP3 AKR1C3 FGFR3P5 TPTE2P3 CACNA2D4 SPARC C14orf144 PLEKHM2 ASB17 RNU1-33P FEZ2 CDC5L SLC9B1 EFCAB1 RPS24P16 KPNB1 LRRC37A7P TAS2R38 NF1P3 TTLL8 MRPS31P2 RASGRF2 ST7 MAMDC2 RPS17P14 RNU6-1193P PCNXL2 MTUS2-AS2 TMEM101 NF2 EVA1C FBXO32 PCDHGA6 DDX3X IGLV4-69 ARHGEF10 RNU6-410P SERPINB12 CASP12 E2F3 FRG2B FGD6 TFF3 MIR603 LINC00687 VDAC1P1 ADCY9 RMI2 MROH1 ULK4P2 NPFFR1 NPC1 PCDHGB1 RPL12L3 C17orf51 SMARCE1 SLC18B1 MIR649 ZNF678 MLLT10P1 TMEM106B TRAP1 SCARNA17 ATP2B1 ATF7IP2 C6 KCTD9P2 GTF2IP2 IGF2BP1 ZNRF1 SERHL ATRN FBLN5 MIR3152 MIR551B CHKB-CPT1B ARNT2 SPEF2 CXorf22 H2AFY SNORD115-11 CEACAMP6 KRTAP4-12 IGHV1OR15-4 GRAP2 MUM1L1 HNRNPA1P71 CUBN RNU6-1280P CYP39A1 DNAJA1P5 RNU4-82P AMZ2 SIDT1 ARL2BPP8 TPRG1L BTN2A1 ASNSP1 IGF2BP3 STK32C DHX35 DEC1 LINC00520 G2E3 RPS15AP34 CCDC88B TMCC3 RN7SL143P TCL6 SAMSN1-AS1 CD2BP2 PAPOLA TOP3A TET3 CASP6 MAGEB3 ZNF582-AS1 NOC4L PIGFP2 MNS1 MSANTD2P1 ZNF718 FAM209A TANGO2 CCDC64 HERC2 NASP PIK3R5 CPNE5 GZMAP1 CLTA PARP16 ASB13 SLC6A10P CHRNA5 RNU6-1291P FAM63B TSPAN13 RNU6-311P SPIRE2 FTH1P27 LINC00374 NKD1 ARHGAP39 AKR1CL1 HTR7 ZNF345 RIPK4 MYL12A TRUB2 LINC00656 CEP170 DNAJC5 LINC00440 RIN2 MIR99A RNU6-1132P OTUD4 LLGL2 SERPING1 TMEM120B PCDH7 ASIP CDH10 KL USP17L25 MZT1 C1orf168 ZNF41 TPTE2P2 DNMT3B OR10T1P PPP2R5A FYB SLC17A3 EBPL IGHVII-62-1 BDH2 CNN2P7 MIR3198-1 BACE2 KIAA0355 MIR548AX CPEB1 PCDHGA1 LSM12 PWRN3 VASH1 SLC35A5 MEG8 MIR1281 DIDO1 DIRC1 MARK3 DHX15 KRTAP29-1 AGGF1 ITPRIPL2 ADAMTS2 NR2C2 RBM44 MMP27 ZNF432 SPG11 VN1R4 RARRES2P4 FUBP1 KIAA0586 STON1 CPAMD8 FOCAD PCA3 OR7E83P PLEKHM1P PRG3 KRT43P GANC DDX24 OR5H8P ATP1A1 MIR487A PGM2L1 RNU4-59P STRC BCL2L15 RN7SL646P YIPF6 ALG6 PPL LRTM1 DNM1P51 MIR485 TMEM212 CHAF1A PHF21B TRAV6 TDRD10 RPL9P28 TOR1AIP1 ADC PXN SVEP1 TNF USP17L24 PHYKPL VTI1A ASAP1 RNU4-40P HPYR1 TMEM179 DSCR10 FYN KIR3DP1 TTC12 CSPP1 CCDC169-SOHLH2 DCLK2 PRELID1P2 EPHA7 LINC00408 PPOX CRCP C7orf49 KANK3 RAD54B MIR1911 LRR1 OR8A2P LINC01070 MSANTD3 ZNF177 MCFD2 IGKV2-36 POM121L9P ZNF627 RNU2-42P KRT8P15 RN7SL204P RN7SL194P C1QTNF7 ARR3 C17orf80 GALNT2 EFCAB5 RNF150 CNOT7 FSHR GAS8 MARCH3 BMP15 SGK2 RNVU1-17 SNORD115-40 LINC00380 PSIP1 NOL11 TRPM2 SPRR4 SLC30A7 SMCHD1

		LINC00507 NET1 ARHGAP12 DPY19L3 CILP2 CCDC176 ICT1 PLCXD3 NAP1L6 MIR889 APTX MXRA5 SCFD1 MTND1P31 ZNF705A GNG2 RN7SL662P UBE2K RRH BCAR3 DRP2 RNU7-176P PDZD7 POR FHL5 TRIM60P13 RPS27P16 RUFY1 LINC00382 TMPRSS4-AS1 IGHV3-41 RPS3AP41 SNORD115-38 LMX1A PIGK PLOD2 TTC27 FIGN NUF2 OR4C14P ABT1 ARID2 CD86 MIR670 RNA5SP300 ZNF33BP1 B4GALT4 FAM228B RNU6-78P LINC01043 DNM3-IT1 GFRA2 FAM104A RPS23P5 COL8A1 ISCA1P3 ATXN3L FAM85B REEP1 FAM90A22P CHMP4C MIR548F1 NR2F2 CTTNBP2 BCL2A1 C14orf164 C2orf27B IGHVIII-67-3 LINC01088 PARP8 ZNF215 RNU6-725P EFEMP1 DTX4 RNA5SP186 PIGB SRRM1 NME9 RN7SL321P AJAP1 ZNF608 RNU6-250P SNX19P2 RASSF8-AS1 DUSP23 HRNR IGKJ4 ABCC9 KLHDC8A BPIFB4 MRPL42P4 RWDD2B INSL6 SERPINA2P INPP5F MIR134 CRADD SHC2 NCOA5 RNA5SP478 VASH2 WWTR1-IT1 OR8U1 TPTE2P1 PTTG1IP COL9A1 GGT1 ADARB1 ZNF664 GPC4 RN7SL568P SOGA1 HAS3 SOD1P3 OR10Z1 PAGE1 SLC7A13 TRPM7 CGNL1 PPP2R4 CDRT1 RN7SKP6 LINC00032 VSTM2B MIR4634 STARP1 ENPP7P5 CNTLN ASL NEK5 C1orf173 AMY1A KLHDC7A BMS1P10 DSCAM-AS1 PLCH1 PCNXL4 PCDHGB7 C14orf39 HDAC4 RNASE13 GLRA1 TRAV33 MIR376A1 STK36 RNU4-24P CTNNBIP1 SRRM1P2 FAM90A20P YAF2 WDR95P KIDINS220 KIAA1644 SMPD4P2 CT49 HNRNPUP1 MRM1 F11-AS1 HNRNPA2B1 GADD45A NHLRC2 ZNF430 RCHY1 ZNF793 FTO MGC4294 USP46 SNORD23 RNU6-1327P FAM201B ADORA2A-AS1 RNA5SP492 EIF3A ADAM28 TMED8 SDHAF2 KRTAP4-11 RN7SKP60 MIR648 ACSBG1 POLR2F KRTAP5-8 IZUMO3 RNU6-1005P KRTAP20-1 SMAD1 ARHGEF15 OR1E3 ZNRF3-IT1 LINC00523 RPL21P6 VPS37A KRTAP12-4 LARS2-AS1 C1QTNF6 BRD7 FCF1P10 RNU6-280P KRTAP27-1 SFI1 GNE RBMX2P1 TSPEAR-AS1 HMG20A ARL6IP5 COPG2 RNU4-60P PAPPA-AS1 CD163L1 ZNF880 NOX5 C1orf167 EEF1A1P1 ATP5J SRP54 DNAAF2 TAS1R2 ADORA3 TTI1 SH2D7 HYAL4 BACE2-IT1 SP2 CYTIP UXS1 PCDHGA8 DPP9 RGL2 PMPCB DHRS4L2 GLDC TTC29 OR4C10P CXorf21 IL17RA YTHDF2 BLZF2P ZNF729 MIR548AS RN7SL177P PJA1 IGLVIV-65 DKFZP761J1410 MZF1 IGHVII-40-1 COL16A1 TTC22 KRTAP6-1 OR5D15P SMIM2-AS1 TP53I11 CACNA1C-IT1 SHOC2 NTNG1 ZNF606 ADTRP SNORD116-18 IL1RN PCDH17 PHF8 TFPI USP17L9P ISX MIR323B RAD51AP1 ABCA9 PQLC1 SEC14L6 CYB5R2 BFSP1 DSCR9 ANKRD20A11P LCP2 RMRPP4 NAP1L4P3 SLC39A8 PET117 IL4R RNU7-144P TM4SF1 SMG7 ZNF564 PAXBP1 PLGRKT COPS8P3 RNU6-288P RNA5SP219 IQSEC3 ZNF782 TGFBR3 PNLIPRP3 RNU6-772P PPA2 MAP3K7 UPRT AKR1C1 ZNF615 GTF2I C16orf80 C15orf32 SLC7A1 SSPN ADAM17 USP36 CHRNA3 OR6C68 TMEM186 SLC35F2 PCDHGB6 RFPL4AP7 OR7D1P RMRPP5 WBP11P1 CYP2AC1P FSBP KRT25 HMGN3P1 C9orf40 KCNH7 NUAK2 GNG5P5 KRTAP15-1 NUDT4 RNU1-104P CLDN1 PCTP ABCC1 WFDC8 IGHV7-56 SPRR2G TMEM211 RBAK-RBAKDN WDR83OS BCRP1 WIPF1 IGF2R C21orf90 PTPRU LY86-AS1 SNTB1 TGFB1I1 TSGA13 SHOX2 NPY4R RNU6-530P MIR381 EGLN3-AS1 ZNF207 RN7SL153P RNASE12 RN7SKP100 NMNAT1P4 IGHVIII-67-2 ANKRD32 CERS6 SCMH1 VPS26AP1 ATP8B5P USP32P1 TPH2 WDYHV1 SNORD115-10 KIAA1598 MORF4L1 APC ELK3 ZNF233 FAM111B OLFM3 RRP15 LINC00508 COX5BP6 ADNP2 SGK3 FAM90A21P INO80 MARCH11 NAA11 TIMD4 LINC00317 OR4A43P RGS7BP TFF1 FSD1L STON1-GTF2A1L PLEKHA6 COL27A1 PHLDB2 TMEM56 SCAF4 PRR9 MTND5P14 IGHV3-47 TMC7 FBXL13 KAT7 HNRNPA1L2 IFI27L2 MIR376C MYBPC2 ZNF235 OR6K4P ACSL4 CAPZB PPM1D OR10N1P ZNF850 NXPH2 MACC1-AS1 SLC5A4 SNX19P1 MAGEB2 CD1C CKAP4 SLFN12L CCDC13 MOB3B SNORA16 PGK1 RNASEH1 MIR656 ANKS1A RNF135 NBAS MTND1P17 RN7SKP86 NBN PANX1 C12orf42 MIR767 EHF LINC00884 RNA5SP20 FRMD3 KLRF2 RPS15AP1 SETD2 RASSF3 CYCSP6 KRTAP9-3 RNU6-494P SNORD116-19 DCK ZBTB34 ZNF826P ENPP7P2 RNU6-954P SQRDL TRIM23 DPYSL2 MAGEC3 FPR3 MIR381HG RN7SL17P OR4Q1P CYLC2 FLVCR1 RAB3C CEP135 UBE2E3 BTNL9 EPN2-AS1 VEPH1 FAM129A OOSP1P2 BCAN AKAP7 RNU6-953P OR4C7P TGM2 STAM MIR4535 MARCH10 GRK6 MIR382 CRLS1 C7orf69 ZNF277 SQSTM1 ANKRD26P1 ZNF879 PHKA2 RNU6-1066P DEFB116 KRTAP4-3 APOL6 KRT39 ZNF397 MIR5704 MIR1276 DPRXP4 KRT8P25 LINC00442 DICER1 ZNF148 SULT1A4 GGT8P C5orf38 THOC2 SLC16A12 UBQLN4 TAF1B LINC00906 LINC00898 IGHV3-63 IGKV1OR2-2 GREB1 NUP62CL ADAT2 ARNT REEP3 TCAM1P MIR320B2 CHMP1B2P EIF4EBP3 OR51F1 CSF1 REST IGHVII-44-2 MRPS11 STOML1 MAEL SDR42E1 LINC01057 RNU6-127P SNORD109B RNA5SP465 TMEM233 RNF180 TAS2R41 H3F3AP4 DCLK1 RNF215 NDST4 MIR543 ADORA2A FAM90A23P RNA5SP495 TSEN15 MIR521-2 CCDC122 SYPL2 COL21A1 GPATCH2L BACH1-IT3 TSC22D3 NRIP1 MROH7 RNU6-26P ARF1 ARID5B SIPA1L2 PLCH2 RCAN2 CASC18 MTMR8 RPL31P3 PPIAP22 CNN3 MIR105-2 SERPINB7 AKNAD1 FAM27A IGHVII-65-1 BMS1P13 PSMG2 MYBL2 RN7SL373P HSPA4L RPL21P11 POU5F1P6 FAM208B METTL15 OR5J7P CUZD1 CPB2 UHMK1 RNF219-AS1 LINC00351 CHST13 VDAC2P1 NR3C1 PPIAP6 MIR300 BRF1 CDCA7L MTOR FAM222B MCM3 KCNB2 SLC19A3 C17orf75 RNU6-690P CCDC170 RN7SKP197 HNRNPA1P40 RORB KIAA1407 VPS39 VAPB CFL1P6 TUFT1 SNAP25 FBLN1 MTFMT LUC7L RAF1 ANO1 ABCD1P5 JAKMIP1 RIMS3 GRPEL2P1 MIR4290 MIR410 RPL4P2 FHL2 CDC42BPG HIGD1A LTB LINC01101 SNRPD1 OR4A41P NEU3 ZFPM1 BZW1 PIP5K1A GDAP1 OR51H2P RPL8P2 ZRANB1 RGS7 DISP1 MYCT1 CPT1B SCGN MS4A5 GPN3 RNU6-21P TFAP2D TNMD RN7SKP101 CLVS2 CYP7B1 RN7SL609P KXD1 OSBPL9P4 USP25 OR2BH1P RN7SL674P BMS1P14 FCRL2 H3F3C KBTBD11 HBD FAM220A TSPAN33 TTC32 IGKV1-37 CES1P2 LRFN2 RNU6-1049P ACO1 UBE2Q2P11 MEGF9 MIR5190 OR4C12 RNU1-11P SNUPN KRT18P31 PCDHGA9 NAPEPLD GCNT7 OBSCN GACAT1 C20orf196 IGKV2OR2-1 IL1RAP UBXN2B MRPL3P1 FAM118A TAF4 IGHV3-48 WRAP73 SNORA38 RPS3AP1 MANEAL CENPBD1P1 LRRTM1 VAV2 FAM90A7P VPS13A SPRR1A SCEL SNX20 NSUN3 ILF3 RNU6-400P RP2 ZFRP1 OR5E1P DPRXP5 MIAT DCDC2 ZNF621 SEMA4B DYNC1I2 LGALS14 DHRS3 TXNDC16 CEP89 YRDCP3 SPATS2 ABHD17C ZNF292 ZNF829 NOS2P3 SNTB2 SLC7A8 TMEM41B MYO5BP3 FAM149B1 S100A11 SPATA7 DOT1L ZNF521 RNU7-35P ZBTB8A SNORD113-2 SMYD2 NFAM1 TMEM194B ONECUT3 ZMAT3 TRAV8-4 IPPKP1 VIPR2 GMEB2 MIR1185-2 LINC00632 MX2 B3GAT2 LAMP5 EFCAB4B UBE2G2 ACSM5 KIAA0040 RN7SL683P HLCS-IT1 KRT2 CA5AP1 RBKS DNAJC6 DSG4 PGM2 LINC00521 M1AP NUS1P2 ZNF610 WBP1L RXRG DNAH10OS H2BFM KDR BRD9 PCDHGA2 OR2M5 OGT MSL2 MOXD1

		FAM13C OR5AO1P DPF3 HNRNPA1P68 KCNT2 GZMH DENND5A CRYBB2P1 RNU5A-5P MED4 RNF220 PTPRS PNPT1P1 TNC DOCK9 PML FAM101A AGBL3 TTLL12 RBCK1 IGHV3-75 RN7SL552P FAM27E2 FAM83G IL2RA TRAPPC12 FAP NSF SCARA5 TPT1P5 ERICH2 HDX SCPEP1 RN7SKP85 RBAKDN LINC00200 PCDHGB8P SEPP1 CPEB2 LINC00885 TMEM56-RWDD3 ATAD2 RPS10P7 LRRK1 RNA5SP221 DNMT1 PIWIL4 MRGPRG ZNF571 UBFD1P1 ELAVL4 SNORD116-16 CADM3 CDH9 PIP5K1P2 FOXRED2 IGLV2-18 IGHV3-65 GLTSCR1 AK4 DGUOK-AS1 FMNL3 WDR25 MXI1 USP17L26 MTURN GRAMD4P5 GYG1P1 SRIP1 ZNF451 ADORA1 MIR514A1 VN1R87P MRPL39 MEG9 SNORD27 HPCAL1 PTGR1 RNU6-405P ST7-OT4 SAP18 RALA SPCS2P4 IGDCC4 RN7SKP126 EDDM3A GNPTAB RPS26P30 ABCC10 CACNG3 RARRES2P1 COL2A1 RPA1 PPIAP14 MIR4760 NAP1L4P1 LINC00617 MBD3L1 SLC30A10 DNAJC2 NECAB1 MYO5BP1 SOX8 TNFAIP8 KIAA0195 CALCRL PTP4A1P1 RNU1-150P ABAT RPS24P12 SLC22A25 EGFL6 BCL2L1 BRSK2 SCARNA21 MIR495 HDAC2 THBS2 IGLV3-19 MRPS27 MLST8 TVP23C-CDRT4 SMOC1 TMEM138 ADAT3 C9orf131 RNU6-230P MIR155HG OR8K5 IGHV3-43 RNASE11 RNA5SP497 MIR4480 CRNN SCAMP1 SLX4IP BANP MTND2P4 CCDC169 MIR548AL ZNF569 LYPD5 RN7SKP147 HNRNPA1P53 COX6A1P3 OR1AA1P CAB39 ENPP7P1 TMEM161A OR11G2 RAD23BLP AAGAB YWHAQP9 FAM189A2 MFSD12 OR52B4 CTDSPL PDE11A RNU6-576P POLR3H THUMPD1 MAT1A CLYBL USP32P3 ACIN1 CEP41 GNB5 IL16 LINC00972 ZSWIM7 DEPTOR CCDC88C B4GALT3 IGHV3-60 NVL RNA5SP490 ATAT1 TMEM220 EDARADD CENPV EWSR1 RNU6-1239P SNORD113-1 RNF144A-AS1 SNX19P3 ZNF337 MIR548X RAD51D MIR369 DYRK1A CUL2 RNASET2 SCNN1A IGKV1OR22-5 LINC00378 ZNF443 OR5V1 KRTAP10-10 RNU6-898P SART3 ACTA2-AS1 MAS1LP1 SNORA80 DNAJC3-AS1 SNRPD3 RBMX2P3 OR4M1 FDXR RAB27A PNLIPRP1 TMTC4 L3MBTL3 HLA-DQB2 MROH7-TTC4 SGOL2 IGHV3OR16-12 ZNF736 CLIP1 RUSC1-AS1 GUSBP6 TUBGCP6 MORN2 5S_rRNA PLAC4 BLOC1S6 MTND4P14 BCL11A RPL18AP14 ZNF330 ADAM20 SNORD115-45 ATP6V1D SAA2 LAMA4 PMEPA1 C1orf94 RN7SL659P IGHV4-55 ZZZ3 RN7SL163P MIR548Q UBE3C PDSS2 ATE1 CCDC92 RPSAP55 CEP44 PEX5L CYP46A1 TERF1P1 FAM90A15P UCHL1-AS1 RNU6-249P GATAD2B RNU6-540P ANXA4 IGKV3OR22-2 MYOM3 ATIC OR8S1 SYNPR-AS1 NPL CES1P1 SNORD115-34 GAB1 SULT1B1 ZNF845 DGCR2 DHRS4-AS1 TRDC RPS20P1 JPH3 SLC38A7 CYP4F29P RNF128 SLC25A48 CP BTF3P10 AMD1 LCE4A DNAH5 KHDC1 PGPEP1 CCNYL2 WDR93 CACNA1I SEC22A RPS12P21 RNA5SP280 NUSAP1 PCNT PAH USP3 LINC00940 DCLRE1C HBG1 LINC00353 ZNF622 SELO OSBPL5 SNORD115-39 TOP1 RXFP2 TRDV3 EXOC6 RN7SKP199 SHISA5 MPST
