# Supplementary material for: The Role of rDNA Clusters in Global Epigenetic Gene Regulation
Source: Front Genet. 2021 Aug 31;12:730633. doi: 10.3389/fgene.2021.730633 (PMC8438155; doi:10.3389/fgene.2021.730633)
Supplement: Supplementary file 2 [file Data_Sheet_2.PDF]

Table S2. The association of 523 overlapping genes (Figure 6) with silencing by the H3K27me3 mark in different cell types (ENCODE Histone Modifications 2015).

Term	Overlap	Adjusted P-value	Genes
H3K27me3 bronchial epithelial cell hg19	119/2082	3.501211322077821E-14	*ATP8A2;CLSTN2;CTNND2;MIR663A;DACH1;FAM110B;EPHB1;SH3GL2;ANKS1B;PCDHAC1;EPHA5;KIRREL3;EPHA6;SEMA6D;KAZN;UNC5C;UNC5D;KIAA1217;MIR3648;CACNB2;INTS4L1;SCN8A;HECW1;ELMO1;ASTN2;FREM2;DGKI;SKAP2;KHDRBS2;RTN1;PDE1C;NTM;CACNA1D;CACNA1C;NPAS3;NKAIN2;SNTG1;SYNDIG1;PLXNA4;LINC00273;WSCD1;CNTN5;AUTS2;LRP1B;PTPRD;SDK1;DAB1;FGF14;NBEA;CNTN4;XKR4;ALK;PTPRT;ROBO2;RYR2;SLC35F1;FMN2;ARHGAP6;RYR3;HAPLN1;GRM1;ROBO1;TTC28;GRM7;ADAMTSL3;DLGAP1;PRKG1;PRMT8;GRID1;TRPC4;COL25A1;DCC;HUNK;TACR3;NRG1;KSR2;MYRIP;SEZ6L;SYN3;SORCS3;ANO2;NRG3;MIR3687;COL6A5;PLCB1;DSCAML1;MAPRE2;PDZRN4;SLC24A2;OCA2;ACSS3;SATB2;PON1;ADCY2;LRP2;TMEM163;HDAC9;RELN;ANKRD36BP2;GPC5;CSMD3;CSMD2;CSMD1;GPC6;RUNX1T1;CA10;GABRA5;ATRNL1;ANKRD30BL;PLCL1;ZNF804A;LHFPL3;VWA3B;SGCZ;HS3ST4;STXBP5L;FAM155A;ASXL3;APBA1*
H3K27me3 SK-N-SH hg19	120/2149	7.287490971519383E-14	*ATP8A2;CLSTN2;CTNND2;MIR663A;CDH4;PIEZO2;HMCN1;SAMD12;MIR4461;EPHB1;SH3GL2;PKNOX2;EPHA5;KIRREL3;EPHA6;DSCAM;SEMA6D;KAZN;UNC5D;FOXP2;KIAA1217;MIR3648;INTS4L1;HECW1;PRKD1;ERG;ZFPM2;FREM2;SKAP2;RTN1;PDE1C;NTM;GLIS3;NPAS3;NKAIN2;SNTG1;ZMAT4;ADAMTS17;SYNDIG1;PLXNA4;TRPM3;GPM6B;LINC00273;WSCD1;CNTN5;PTPRD;FAM135B;CDK6;MYO5B;CNTN4;XKR4;PTPRT;ROBO2;CNTNAP2;RYR2;EDA;SLC35F1;RYR3;RERG;GRM1;PTPRG;ROBO1;GRM5;CA3;ADAMTSL3;DLGAP1;GPR139;CHST9;TRPC5;PRMT8;GRID1;TRPC4;COL25A1;DCC;ARAP2;HUNK;TACR3;COBL;ELOVL7;KSR2;SEZ6L;SYN3;SORCS3;ANO2;DNM3;NRG3;MIR3687;CDH13;COL6A5;DSCAML1;MAPRE2;PDZRN4;PON3;ACSS3;LAMA2;SATB2;PON1;ADCY2;LRP2;TMEM163;HDAC9;RELN;SPOCK3;ANKRD36BP2;SPOCK1;GPC5;CSMD3;CSMD2;CSMD1;OPCML;GABBR2;CA10;GABRA5;ANKRD30BL;ZNF804A;LHFPL3;VWA3B;SGCZ;HS3ST4;ASXL3*
H3K27me3 megakaryocyte mm9	137/2638	8.045575428372598E-14	*FHOD3;ATP8A2;CLSTN2;THSD7B;DGKB;CTNND2;CCDC91;PIEZO2;HMCN1;DIP2C;SAMD12;NCKAP5;SH3GL2;ANKS1B;PCDHAC1;CSGALNACT1;EPHA5;EPHA6;CACNA2D1;MAGI2;FNDC3B;UNC5C;ANK2;UNC5D;PCDHA12;FOXP2;PCDHA10;HECW1;ELMO1;TTLL11;PRKD1;ASTN2;ZFPM2;KHDRBS2;PDE1C;NTM;TSHZ2;GLIS3;FHIT;NKAIN2;SNTG1;ZMAT4;LARGE;ADAMTS17;SYNDIG1;PCDHA2;LRRC4C;ASIC2;TRPM3;AUTS2;GAREM;RANBP17;ARHGAP24;IGSF11;PTPRD;MSRA;SDK1;FAM135B;DAB1;CDK6;FGF14;DLG2;NBEA;MYO5B;CPE;CNTN4;XKR4;FBXL7;GABRB3;DPP10;ALK;ROBO2;CNTNAP2;RYR2;ITGAM;EDA;OTUD7A;PTPRM;FMN1;SLC35F1;HAPLN1;GRM1;PTPRG;ROBO1;TTC28;GRM5;SGCD;GRM7;ADAMTSL3;GPR139;TRPC5;PRMT8;GRID1;TRPC4;COL25A1;TACR3;COBL;NRG1;MYRIP;SEZ6L;SYN3;SORCS3;DNM3;NRG3;DPYD;PLCB1;DSCAML1;PDZRN4;SLC24A2;SEMA3D;ATP1A4;ADCY2;LRP2;TMEM163;THSD4;RELN;SPOCK3;PRRG1;SPOCK1;GPC5;SLIT3;CSMD1;GPC6;CLVS1;PCDH9;GABRA5;ATRNL1;PLCL1;GABRA3;LHFPL3;SGCZ;HS3ST4;STXBP5L;FAM155A;FRAS1;ASXL3;DLC1*
H3K27me3 mammary epithelial cell hg19	128/2586	2.8083918465743247E-11	*ATP8A2;CLSTN2;CTNND2;MIR663A;DACH1;PIEZO2;SH3GL2;ANKS1B;EPHA5;KIRREL3;EPHA6;CACNA2D1;SEMA6D;MAGI2;KAZN;UNC5C;UNC5D;ROCK1P1;FOXP2;KIAA1217;MIR3648;CACNB2;PCP4;HECW1;ELMO1;PRKD1;ERG;ASTN2;ZFPM2;FREM2;DGKI;KHDRBS2;RTN1;PDE1C;NTM;GLIS3;NPAS3;NKAIN2;SNTG1;ZMAT4;ADAMTS17;SYNDIG1;PLXNA4;GPM6B;LINC00273;CNTN5;AUTS2;FRG1B;KY;PTPRD;SDK1;FGF14;NBEA;CNTN4;FBXL7;GABRB3;PTPRT;ROBO2;CNTNAP2;RYR2;SLC35F1;FMN2;RYR3;GRM1;ROBO1;TTC28;IQCJ-SCHIP1;GRM7;ADAMTSL3;DLGAP1;GPR139;PRKG1;GRID1;TRPC4;COL25A1;DCC;HUNK;TACR3;KSR2;MYRIP;SEZ6L;SYN3;SORCS3;ANKFN1;NRG3;MIR3687;COL6A5;PLCB1;MAPRE2;PDZRN4;CNTNAP4;SLC24A2;OCA2;ACSS3;LAMA2;DNAH8;SATB2;PON1;DNAH6;NRXN3;ADCY2;LRP2;TMEM163;HDAC9;PAK1;SPOCK3;ANKRD36BP2;GPC5;CTNNA3;CSMD3;CSMD2;CSMD1;RUNX1T1;CLVS1;CA10;GABRA5;ATRNL1;ANKRD30BL;GABRA3;ZNF804A;LHFPL3;MERTK;SGCZ;HS3ST4;STXBP5L;AGBL4;FAM155A;ASXL3*

H3K27me3 keratinocyte hg19	113/2221	1.502175353257994E-10	FHOD3;CLSTN2;CTNND2;MIR663A;SLC8A1;DACH1;NDST3;FAM110B;PIEZO2;SH3GL2;ANKS1B;PCDHAC1;EPHA5;KIRREL3;EPHA6;HFM1;SEMA6D;MAGI2;KAZN;ROCK1P1;KIAA1217;MIR3648;CACNB2;INTS4L1;ELMO1;PRKD1;ASTN2;ZFPM2;FREM2;DGKI;LINC00284;KHDRBS2;PDE1C;TMTC1;CACNA1D;NKAIN2;ZMAT4;GPM6B;LINC00273;SPECC1;WSCD1;CNTN5;AUTS2;LRP1B;FAM135B;FGF14;NBEA;MYO5B;CPE;CNTN4;XKR4;FBXL7;GABRB3;PTPRT;CNTNAP2;RYR2;SLC35F1;RYR3;GRM1;ROBO1;TTC28;ADAMTSL3;DLGAP1;GPR139;ZNF385D;CHST9;TRPC5;GRID1;TRPC4;DCC;HUNK;TACR3;COBL;NRG1;KSR2;SEZ6L;SYN3;ANO2;PLCB4;NRG3;MIR3687;COL6A5;PLCB1;MAPRE2;PDZRN4;SLC24A2;PON3;ACSS3;LAMA2;NRXN3;ADCY2;LRP2;TMEM163;HDAC9;SPOCK3;ANKRD36BP2;CSMD3;GPC6;RUNX1T1;LINC00478;CA10;PCDH9;GABRA5;ANKRD30BL;PLCL1;GABRA3;ZNF804A;LHFPL3;SGCZ;HS3ST4;FAM155A;ASXL3;APBA1
H3K27me3 liver mm9	100/2000	8.851954293383034E-9	ATP8A2;CLSTN2;THSD7B;DGKB;HMCN1;SAMD12;SOX6;SH3GL2;ANKS1B;PCDHAC1;CSGALNACT1;EPHA5;KIRREL3;EPHA6;HFM1;DSCAM;CACNA2D1;SEMA6D;MAGI2;DIO2;UNC5C;UNC5D;PCDHA11;FOXP2;TIAM2;CACNB2;SCN8A;HECW1;ASTN2;ZFPM2;DGKI;KHDRBS2;PDE1C;GLIS3;NKAIN2;PCDHA1;SNTG1;HIVEP3;PCDHA2;ASIC2;TRPM3;GPM6B;PCDHA6;WSCD1;AUTS2;RANBP17;KY;PTPRD;SDK1;FAM135B;DAB1;DLG2;NBEA;NSG2;CPE;MIR183;XKR4;FBXL7;DPP10;ALK;SPAG16;SYCP2;LRRK2;OTUD7A;PTPRM;MIR96;GRM1;GRM5;GRM7;ADAMTSL3;TRPC4;COL25A1;DCC;NRG1;ELOVL7;KSR2;NAV2;MYRIP;SEZ6L;ANO2;CDC42EP3;LANCL3;COL6A5;SLC24A2;LAMA2;SATB2;LRP2;THSD4;SLIT3;CSMD3;CSMD1;RUNX1T1;OPCML;CLVS1;PCDH9;PLCL1;LHFPL3;HS3ST4;STXBP5L;APBA1
H3K27me3 erythroblast mm9	124/2712	9.186565296317154E-9	FHOD3;CLSTN2;DGKB;CTNND2;CDH4;DACH1;FAM110B;PIEZO2;HMCN1;DIP2C;SH3GL2;ANKS1B;WLS;PCDHAC1;EPHA6;CACNA2D1;SEMA6D;MAGI2;FNDC3B;UNC5C;ANK2;UNC5D;CACNB2;HECW1;ELMO1;PRKD1;ASTN2;ZFPM2;FREM2;DGKI;KHDRBS2;RTN1;PDE1C;NTM;TSHZ2;GLIS3;NKAIN2;SNTG1;ZMAT4;LARGE;ADAMTS17;SYNDIG1;HIVEP3;LRRC4C;ASIC2;TRPM3;GPM6B;WSCD1;AUTS2;EXOC6B;KY;ARHGAP24;IGSF11;PTPRD;FAM135B;FGF14;DLG2;NBEA;NSG2;MYO5B;CPE;XKR4;FBXL7;GABRB3;DPP10;ALK;SPAG16;ROBO2;CNTNAP2;RYR2;EDA;SYCP2;LRRK2;OTUD7A;PTPRM;SLC35F1;BICD1;GPHN;HAPLN1;PTPRG;ROBO1;AKAP13;GRM5;GPR139;TRPC5;PRMT8;TRPC4;COL25A1;IFNGR2;ARAP2;TACR3;COBL;NRG1;MYRIP;PARD3B;DNM3;NRG3;CDC42EP3;DPYD;LANCL3;PLCB1;DSCAML1;MAPRE2;SHANK2;CNTNAP4;SLC24A2;SGMS1;SEMA3D;ADCY2;LRP2;LRRC16A;TMEM163;GPC5;CORIN;OPCML;CLVS1;PCDH9;GABRA5;PLCL1;GABRA3;LHFPL3;HS3ST4;STXBP5L;ASXL3
H3K27me3 skeletal muscle myoblast hg19	99/2000	1.4628488132705944E-8	CLSTN2;CTNND2;MIR663A;DACH1;NDST3;PIEZO2;MACROD2;HMCN1;NCKAP5;EPHB1;SH3GL2;ANKS1B;PCDHAC1;EPHA5;EPHA6;DSCAM;MAGI2;UNC5D;ROCK1P1;KIAA1217;CACNB2;PCP4;ELMO1;FREM2;LINC00284;PDE1C;NTM;TSHZ2;CACNA1D;CACNA1C;NPAS3;NKAIN2;SNTG1;SYNDIG1;DTNA;WSCD1;CNTN5;LRP1B;PTPRD;SLC2A9;SDK1;DAB1;NBEA;MIR1324;MYO5B;CNTN4;XKR4;ALK;INVS;PTPRT;ROBO2;RYR2;EDA;SLC35F3;LRRK2;SLC35F1;FMN2;RERG;GRM1;TTC28;CA3;DLGAP1;PRKG1;CHST9;GRID1;TRPC4;COL25A1;HUNK;TACR3;COBL;ELOVL7;KSR2;MYRIP;DNM3;NRG3;CDC42EP3;COL6A5;PDZRN4;OCA2;ACSS3;HDAC9;SPOCK3;ANKRD36BP2;GPC5;CTNNA3;CSMD2;DYNC1H1;GABBR2;CA10;ATRNL1;PLCL1;ZNF804A;LHFPL3;SGCZ;HS3ST4;STXBP5L;FAM155A;FRAS1;ASXL3
H3K9me3 G1E mm9	134/3067	2.1849482360779355E-8	SEMA5A;CLSTN2;DGKB;CTNND2;DACH1;NDST3;MACROD2;DIP2C;NCKAP5;EPHB1;ANKS1B;PCDHAC1;CALN1;EPHA5;KIRREL3;DSCAM;CACNA2D1;MAGI2;FNDC3B;KAZN;UNC5D;ANK3;PCDHA12;PCDHA11;PCDHA10;SCN8A;ELMO1;PRKD1;ASTN2;FREM2;DGKI;KHDRBS2;RTN1;TSHZ2;PCDH15;TMTC1;FHIT;NPAS3;NKAIN2;ARHGAP22;PCDHA1;ZMAT4;LARGE;ADAMTS17;PCDHA5;SYNDIG1;CCDC30;PCDHA4;PCDHA3;HIVEP3;SRGAP3;PCDHA9;LRRC4C;ASIC2;PCDHA8;PCDHA7;PCDHA6;AUTS2;RANBP17;KY;ARHGAP24;IGSF11;PTPRD;SDK1;FAM135B;DAB1;FGF14;NBEA;TBXAS1;CNTN4;XKR4;FBXL7;GABRB3;DPP10;ALK;PTPRT;ROBO2;CSRNP3;TENM4;LRRK2;GRIK4;PTPRM;SLC35F1;FMN2;RYR3;PTPRG;ROBO1;AKAP13;GRM5;SGCD;GRM7;ADAMTSL3;GPR139;PRMT8;KLF12;GRID1;TRPC4;COL25A1;DCC;ARAP2;HUNK;TACR3;NRG1;KSR2;MYRIP;PARD3B;ANKFN1;PLCB4;NRG3;DPYD;PLCB1;DOCK1;PDZRN4;CNTNAP4;SLC24A2;ACSS3;SATB2;ADCY2;TMEM163;RELN;SPOCK3;SPOCK1;GPC5;GPC6;RUNX1T1;OPCML;PCDH9;GABRA5;MGMT;PLCL1;

			AGBL4;FAM155A;NFIA;ASXL3
H3K27me3 CD14-positive monocyte hg19	145/3445	3.920067644152572E-8	*SEMA5A;CTNND2;MIR663A;CDH4;NDST3;FAM110B;PIEZO2;MACROD2;HMCN1;NCKAP5;SH3GL2;PCDHAC1;EPHA5;KIRREL3;EPHA6;HFM1;SEMA6D;MAGI2;KAZN;UNC5C;ANK2;ANK3;PCDHA12;ROCK1P1;PCDHA11;PCDHA10;KIAA1217;MIR3648;INTS4L2;CACNB2;INTS4L1;PCP4;PRKD1;ERG;ASTN2;ZFPM2;FREM2;DGKI;KHDRBS2;PDE1C;TSHZ2;GLIS3;TMTC1;CACNA1D;SEL1L2;NPAS3;SNTG1;ADAMTS17;PCDHA5;SYNDIG1;PCDHA3;HIVEP3;PCDHA2;PCDHA9;LRRC4C;PCDHA7;PLXNA4;TRPM3;PCDHA6;CNTN5;DNAH14;KY;LRP1B;PTPRD;PGM5P2;FAM135B;DAB1;CNTN4;XKR4;FBXL7;ALK;PTPRT;ROBO2;CSRNP3;SLC35F3;SLC35F1;FRG2C;ARHGAP6;BICD1;RYR3;RERG;HAPLN1;GRM1;ROBO1;ADAMTSL1;IQCJ-SCHIP1;GRM5;CA3;ADAMTSL3;DLGAP1;GPR139;ZNF385D;SLC22A10;TRPC5;PRMT8;KLF12;TRPC4;TACR3;COBL;ELOVL7;MYRIP;ANO2;PARD3B;PLCB4;NRG3;BAGE2;MIR3687;LANCL3;CDH13;COL6A5;DSCAML1;DOCK1;PDZRN4;CNTNAP4;SLC24A2;PON3;ACSS3;LAMA2;SATB2;PON1;LRP2;HDAC9;MIPOL1;RELN;SPOCK3;ANKRD36BP2;GPC5;CTNNA3;CSMD3;CSMD2;CSMD1;GPC6;CORIN;CLVS1;CA10;GABRA5;ANKRD30BL;COL22A1;LHFPL3;SGCZ;HS3ST4;STXBP5L;AGBL4;FAM155A;FRAS1*
H3K27me3 ES-Bruce4 mm9	97/2000	4.9720931138823E-8	*CLSTN2;THSD7B;CTNND2;DACH1;PIEZO2;SOX6;EPHB1;ANKS1B;PCDHAC1;CSGALNACT1;EPHA5;KIRREL3;EPHA6;HFM1;DSCAM;CACNA2D1;UNC5C;UNC5D;PCDHA11;FOXP2;TIAM2;CACNB2;PRKD1;ASTN2;ZFPM2;DGKI;TSHZ2;GLIS3;PCDH15;NPAS3;SNTG1;ZMAT4;SYNDIG1;PCDHA4;PCDHA3;LRRC4C;ASIC2;PLXNA4;GPM6B;WSCD1;AUTS2;KY;LRP1B;IGSF11;SDK1;NBEA;MYO5B;CPE;CNTN4;FBXL7;DPP10;ALK;PTPRT;ROBO2;RYR2;SLC35F3;GRIK4;PTPRM;SGCD;GRM7;ADAMTSL3;PRKG1;TRPC5;PRMT8;GRID1;COL25A1;HUNK;MYRIP;SEZ6L;SORCS3;PARD3B;NRG3;CDH13;PLCB1;DSCAML1;PDZRN4;SATB2;TMEM163;RELN;SPOCK3;SPOCK1;GPC5;SLIT3;CSMD1;CORIN;RUNX1T1;GABBR2;PCDH9;GABRA5;PLCL1;SUSD1;LHFPL3;SGCZ;HS3ST4;FAM155A;ASXL3;DLC1*
H3K27me3 endothelial cell of umbilical vein hg19	142/3393	7.801791636079459E-8	*SEMA5A;ATP8A2;CLSTN2;CTNND2;MIR663A;CDH4;DACH1;FAM110B;SAMD12;SH3GL2;ANKS1B;PCDHAC1;EPHA5;KIRREL3;EPHA6;UNC5C;ANK2;UNC5D;ANK3;PCDHA13;ROCK1P1;FOXP2;CNBD1;KIAA1217;MIR3648;CACNB2;SCN8A;HECW1;ERG;ASTN2;FREM2;DGKI;RTN1;PDE1C;TSHZ2;CACNA1D;CACNA1C;NKAIN2;PCDHA1;ZMAT4;LARGE;ADAMTS17;SYNDIG1;SRGAP3;LRRC4C;TRPM3;GPM6B;LINC00273;SPECC1;CNTN5;KY;PTPRD;SDK1;DAB1;FGF14;MIR1324;MYO5B;CPE;CNTN4;XKR4;GABRB3;ALK;PTPRT;ROBO2;RYR2;ITGAM;CSRNP3;SYCP2;SLC35F3;LRRK2;SLC35F1;FMN2;RYR3;RERG;HAPLN1;ROBO1;IQCJ-SCHIP1;GRM5;CA3;GRM7;ADAMTSL3;DLGAP1;GPR139;ZNF385D;PRKG1;SLC22A10;ANKUB1;CHST9;PRMT8;TRPC4;COL25A1;ARAP2;HUNK;TACR3;COBL;NRG1;ELOVL7;KSR2;SEZ6L;SYN3;SORCS3;NRG3;MIR3687;LANCL3;COL6A5;DSCAML1;MAPRE2;PDZRN4;OCA2;PON3;ACSS3;LAMA2;SATB2;PON1;DNAH6;ADCY2;LRP2;TMEM163;RELN;SPOCK3;ANKRD36BP2;DMD;GPC5;CTNNA3;SLIT3;CSMD3;CSMD2;CSMD1;GPC6;CORIN;GABRA5;ATRNL1;ANKRD30BL;PLCL1;ZNF804A;LHFPL3;VWA3B;SGCZ;HS3ST4;FRAS1;ASXL3;APBA1*
H3K27me3 BJ hg19	98/2090	1.9701016351925295E-7	*ATP8A2;CLSTN2;CTNND2;MIR663A;CDH4;DACH1;NDST3;PIEZO2;SH3GL2;ANKS1B;PCDHAC1;EPHA5;EPHA6;SEMA6D;UNC5C;UNC5D;ANK3;FOXP2;KIAA1217;MIR3648;CACNB2;HECW1;ASTN2;ZFPM2;FREM2;RTN1;PDE1C;NTM;TSHZ2;CACNA1D;NPAS3;NKAIN2;SNTG1;ZMAT4;ADAMTS17;SYNDIG1;SRGAP3;PLXNA4;LINC00273;SPECC1;WSCD1;LRP1B;PTPRD;SDK1;FGF14;MIR1324;MYO5B;ALK;PTPRT;ROBO2;RYR2;EDA;SLC35F1;ARHGAP6;RERG;HAPLN1;ROBO1;PRMT8;GRID1;COL25A1;HUNK;COBL;NRG1;ELOVL7;KSR2;MYRIP;SEZ6L;SYN3;SORCS3;ANO2;DNM3;NRG3;MIR3687;MAPRE2;PDZRN4;OCA2;PON3;DNAH8;SATB2;ADCY2;LRP2;TMEM163;HDAC9;PAK1;RELN;GPC5;CSMD3;CSMD2;ATRNL1;ANKRD30BL;PLCL1;LHFPL3;VWA3B;MERTK;HS3ST4;STXBP5L;FAM155A;ASXL3*
H3K27me3 kidney epithelial cell hg19	102/2217	2.2557701468814125E-7	*ATP8A2;MIR663A;DACH1;PIEZO2;SH3GL2;ANKS1B;PCDHAC1;EPHA6;DSCAM;SEMA6D;KAZN;UNC5C;UNC5D;FOXP2;KIAA1217;MIR3648;CACNB2;INTS4L1;SCN8A;HECW1;ELMO1;ERG;ZFPM2;FREM2;KHDRBS2;RTN1;PDE1C;NTM;TMTC1;CACNA1D;NKAIN2;SNTG1;ZMAT4;ADAMTS17;SYNDIG1;PLXNA4;TRPM3;GPM6B;SPECC1;KY;PTPRD;FAM135B;DAB1;FGF14;CNTN4;GABRB3;ALK;ROBO2;RYR2;SLC35F3;RYR3;RERG;HAPLN1;GRM1;ROBO1;TTC28;GRM5;GRM7;ADA*

			MTSL3;DLGAP1;GPR139;PRKG1;TRPC5;PRMT8;TACR3;COBL;NRG1;KSR2;NAV2;MYRIP;SEZ6L;SYN3;SORCS3;ANO2;MIR3687;COL6A5;MAPRE2;PDZRN4;OCA2;LAMA2;DNAH8;SATB2;PON1;ADCY2;LRP2;HDAC9;RELN;ANKRD36BP2;GPC5;CSMD3;CSMD2;CORIN;RUNX1T1;OPCML;CA10;GABRA5;ANKRD30BL;PLCL1;LHFPL3;VWA3B;STXBP5L;AGBL4
H3K9me3 HepG2 hg19	86/1809	9.535647120575813E-7	NCOR1P1;GABRB3;SEMA5A;CNTNAP2;ZNF493;SLC35F3;DGKB;CTNND2;MIR663A;FMN2;FRG2C;ZNF280D;CDH4;SGCD;GRM7;ADAMTSL3;DLGAP1;MACROD2;ZNF385D;MIR4461;NCKAP5;SH3GL2;PACRG;SNRPN;ZNF283;DSCAM;DCC;SEMA6D;DIO2;UNC5C;UNC5D;TNNI3K;PCDHA13;SEZ6L;SYN3;PCDHA12;ROCK1P1;PARD3B;CNBD1;MIR3648;ANKFN1;PCP4;NRG3;ZNF717;BAGE2;MIR3687;PRKD1;ASTN2;ST6GALNAC3;ZNF675;OCA2;TSHZ2;TPTE;SEL1L2;NPAS3;DHX32;PCDHA1;SNTG1;PCDHA5;PCDHA4;PCDHA3;DMD;PCDHA2;CSMD3;FPGT-TNNI3K;CSMD2;LRRC4C;CSMD1;PCDHA7;ZNF585A;LINC00273;CA10;MUC16;GABRA5;ANKRD30BL;DNAH14;RANBP17;AKR1C2;FRG1B;PTPRD;MGAT4C;SLC2A9;AGBL4;MIR1324;TCF4;CNTN4
H3K27me3 osteoblast hg19	92/2000	1.2872032783888762E-6	GABRB3;PTPRT;ROBO2;CNTNAP2;RYR2;ITGAM;CLSTN2;SLC35F3;CTNND2;MIR663A;SLC35F1;FMN2;CA3;ADAMTSL3;FAM110B;PIEZO2;DLGAP1;MACROD2;PCDHAC1;EPHA6;COL25A1;DCC;SEMA6D;HUNK;COBL;NRG1;UNC5D;ELOVL7;KSR2;SEZ6L;SORCS3;ROCK1P1;FOXP2;KIAA1217;MIR3648;NRG3;MIR3687;SCN8A;ELMO1;LANCL3;TTLL11;COL6A5;ASTN2;PLCB1;ZFPM2;FREM2;PDZRN4;SKAP2;PON3;ACSS3;RTN1;SATB2;NTM;ADCY2;LRP2;NPAS3;ENOX2;NKAIN2;SNTG1;ZMAT4;LARGE;ADAMTS17;SPOCK3;CCDC30;ANKRD36BP2;CTNNA3;CSMD2;LRRC4C;CSMD1;GPM6B;RUNX1T1;LINC00273;CA10;DTNA;CNTN5;AUTS2;GABRA5;LHFPL3;SGCZ;HS3ST4;LRP1B;STXBP5L;PTPRD;DAB1;FRAS1;NBEA;MIR1324;ASXL3;MYO5B;APBA1;XKR4;FBXL7
H3K27me3 mononuclear cell hg19	91/2000	2.4062372974884615E-6	SEMA5A;FHOD3;PTPRM;HAPLN1;TTC28;CA3;LIPI;ADAMTSL3;NDST3;FAM110B;PIEZO2;DLGAP1;MACROD2;MIR4461;NCKAP5;PRKG1;SLC22A10;ANKS1B;ANKUB1;KIRREL3;TRPC5;PACRG;DCC;SEMA6D;MAGI2;TACR3;KAZN;UNC5C;ANK2;UNC5D;KSR2;ANK3;PCDHA13;SORCS3;PCDHA12;ROCK1P1;ANO2;PCDHA10;KIAA1217;MIR3648;INTS4L1;MIR3687;HECW1;CDH13;COL6A5;PON3;ACSS3;LAMA2;NTM;DNAH6;TMTC1;CACNA1C;LRRC16A;MIPOL1;PCDHA1;SNTG1;ZMAT4;LARGE;SPOCK3;PRRG1;SYNDIG1;ANKRD36BP2;SPOCK1;PCDHA3;HIVEP3;CTNNA3;CSMD2;LRRC4C;CSMD1;GPC6;CORIN;PCDHA6;OPCML;CLVS1;CA10;CNTN5;ANKRD30BL;COL22A1;DNAH14;RANBP17;LHFPL3;SGCZ;KY;AGBL4;FAM155A;DAB1;FGF14;NBEA;CPE;CNTN4;XKR4
H3K9me3 G1E-ER4 mm9	116/2782	3.1889241611952305E-6	SEMA5A;CLSTN2;DGKB;CTNND2;SLC8A1;DACH1;NDST3;MACROD2;SH3GL2;PCDHAC1;EPHA5;DSCAM;CACNA2D1;MAGI2;FNDC3B;KAZN;UNC5C;UNC5D;ANK3;PCDHA12;PCDHA10;SCN8A;ELMO1;PRKD1;ASTN2;ZFPM2;KHDRBS2;CTBP2;PCDH15;TMTC1;FHIT;NPAS3;NKAIN2;ARHGAP22;PCDHA1;ZMAT4;LARGE;ADAMTS17;PCDHA5;SYNDIG1;CCDC30;PCDHA4;PCDHA3;PCDHA2;PCDHA9;PCDHA8;PCDHA7;PCDHA6;AUTS2;RANBP17;ARHGAP24;PTPRD;SDK1;FAM135B;FGF14;NBEA;CNTN4;XKR4;FBXL7;GABRB3;DPP10;PTPRT;ROBO2;RYR2;CSRNP3;LRRK2;OTUD7A;GRIK4;PTPRM;FMN1;SLC35F1;FMN2;PTPRG;ROBO1;AKAP13;GRM5;GRM7;ADAMTSL3;DLGAP1;PRMT8;GRID1;TRPC4;COL25A1;DCC;ARAP2;TACR3;NRG1;ELOVL7;KSR2;MYRIP;UPK3B;PARD3B;NRG3;CDC42EP3;DPYD;PLCB1;DSCAML1;PDZRN4;SLC24A2;SATB2;ADCY2;TMEM163;RELN;SPOCK3;GPC5;GPC6;RUNX1T1;GABBR2;PCDH9;MGMT;PLCL1;HS3ST4;AGBL4;FAM155A;FRAS1;ASXL3
H3K27me3 cardiac mesoderm hg19	135/3485	1.535890576081863E-5	ATP8A2;MIR663A;CDH4;DACH1;PIEZO2;HMCN1;SAMD12;EPHB1;SH3GL2;ANKS1B;WLS;PCDHAC1;EPHA5;KIRREL3;EPHA6;DSCAM;SEMA6D;MAGI2;ANK3;FOXP2;KIAA1217;MIR3648;INTS4L1;PCP4;HECW1;ELMO1;TTLL11;ERG;ZFPM2;DGKI;KHDRBS2;RTN1;PDE1C;GLIS3;CACNA1D;CACNA1C;NKAIN2;SNTG1;ADAMTS17;SYNDIG1;HIVEP3;PLXNA4;TRPM3;GPM6B;DTNA;WSCD1;CNTN5;KY;LRP1B;PTPRD;PGM5P2;FAM135B;CDK6;FGF14;MYO5B;CPE;MIR183;CNTN4;GABRB3;PTPRT;ROBO2;SLC35F3;LRRK2;FMN2;FRG2C;MIR96;RYR3;RERG;HAPLN1;GRM1;ROBO1;GRM5;CA3;ADAMTSL3;GPR139;ZNF385D;PRKG1;CHST9;TRPC5;PRMT8;TRPC4;COL25A1;DCC;TACR3;NRG1;ELOVL7;KSR2;MYRIP;SEZ6L;SORCS3;ANO2;PARD3B;DNM3;NRG3;MIR3687;LANCL3;CDH13;COL6A5;PLCB1;DSCAML1;MAPRE2;PDZRN4;CNTNAP4;SLC24A2;OCA2;LAMA2;DNAH8;SATB2;PON1;LRP2;TMEM163;HDAC9;RELN;SPOCK3;ANKRD36BP2;SPOCK1;GPC5;CSMD3;CSMD2;CSMD1;CORIN;OPCML;GABBR2;CA10

			;GABRA5;ANKRD30BL;PLCL1;GABRA3;SUSD1;ZNF804A;LHFPL3;VWA3B;SGCZ;HS3ST4;FAM155A
H3K27me3 fibroblast of lung hg19	121/3052	2.1170037133281055E-5	ATP8A2;CLSTN2;CTNND2;MIR663A;DACH1;NDST3;PIEZO2;HMCN1;EPHB1;SH3GL2;PCDHAC1;CSGALNACT1;EPHA5;EPHA6;SEMA6D;KAZN;UNC5C;UNC5D;ANK3;ROCK1P1;FOXP2;KIAA1217;MIR3648;CACNB2;INTS4L1;HECW1;ELMO1;ASTN2;FREM2;DGKI;RTN1;PDE1C;NTM;TMTC1;CACNA1D;FHIT;NKAIN2;SNTG1;ZMAT4;ADAMTS17;SYNDIG1;PLXNA4;GPM6B;LINC00273;DTNA;WSCD1;CNTN5;FRG1B;LRP1B;PTPRD;SDK1;DAB1;FGF14;NBEA;MIR1324;CPE;CNTN4;XKR4;ALK;ROBO2;RYR2;ITGAM;EDA;LRRK2;SLC35F1;FMN2;RYR3;ROBO1;CA3;ADAMTSL3;DLGAP1;TRPC5;PRMT8;GRID1;TRPC4;COL25A1;DCC;HUNK;COBL;NRG1;ELOVL7;KSR2;MYRIP;SEZ6L;SORCS3;ANO2;DNM3;NRG3;MIR3687;COL6A5;DSCAML1;MAPRE2;PDZRN4;PON3;SATB2;PON1;DNAH6;ADCY2;LRP2;LRRC16A;TMEM163;HDAC9;RELN;SPOCK3;ANKRD36BP2;GPC5;CTNNA3;SLIT3;CSMD3;CSMD1;GABRA5;ANKRD30BL;PLCL1;LHFPL3;VWA3B;MERTK;SGCZ;STXBP5L;FAM155A;FRAS1;ASXL3
H3K27me3 astrocyte hg19	87/2000	2.649674308564323E-5	GABRB3;ALK;CNTNAP2;RYR2;ATP8A2;SLC35F3;MIR663A;AFF3;TTC28;IQCJ-SCHIP1;DACH1;GRM7;ADAMTSL3;NDST3;PIEZO2;GPR139;MACROD2;ERC1;SH3GL2;ANKS1B;PCDHAC1;CHST9;TRPC5;EPHA6;SEMA6D;TACR3;COBL;KAZN;UNC5C;NRG1;UNC5D;KSR2;MYRIP;ROCK1P1;PARD3B;KIAA1217;NRG3;MIR3687;SCN8A;HECW1;ELMO1;ERG;COL6A5;ASTN2;MAPRE2;PDZRN4;CNTNAP4;SLC24A2;PON3;KHDRBS2;PON1;DNAH6;ADCY2;LRP2;TMEM163;NKAIN2;PAK1;RELN;SNTG1;ZMAT4;ADAMTS17;SPOCK3;GPC5;CTNNA3;CSMD1;TRPM3;OPCML;DTNA;WSCD1;CNTN5;GABRA5;COL22A1;PLCL1;LHFPL3;VWA3B;MERTK;HS3ST4;ARHGAP24;STXBP5L;PTPRD;FAM155A;FAM135B;DAB1;ASXL3;CPE;CNTN4;XKR4
H3K9me3 endothelial cell of umbilical vein hg19	87/2000	2.5292345672659446E-5	NCOR1P1;GABRB3;SEMA5A;DPP10;ALK;PTPRT;ROBO2;CNTNAP2;RYR2;SYCP2;THSD7B;SLC35F3;CTNND2;MIR663A;FMN2;ZNF280D;CDH4;GRM7;LIPI;ADAMTSL3;FAM110B;DLGAP1;GPR139;MACROD2;CALN1;KIRREL3;CHST9;TRPC5;SNRPN;EPHA6;DSCAM;DCC;DIO2;ARAP2;KAZN;PCDHA13;SORCS3;PCDHA12;ROCK1P1;TEKT4P2;PCDHA10;KIAA1217;MIR3648;ZNF717;BAGE2;MIR3687;DUX4L4;ASTN2;SHANK2;CNTNAP4;OCA2;KHDRBS2;ADCY2;PCDHA1;SNTG1;LARGE;PCDHA5;PCDHA4;PCDHA3;PCDHA2;CSMD2;LRRC4C;PCDHA8;CSMD1;PCDHA7;PCDHA6;ZNF585A;OPCML;LINC00273;CA10;MGAM;MUC16;CNTN5;AUTS2;GABRA5;ANKRD30BL;GABRA3;RANBP17;FRG1B;SGCZ;HS3ST4;PTPRD;FAM135B;DAB1;MIR1324;CNTN4;XKR4
H3K27me3 H7 hg19	87/2015	3.285577937242665E-5	FHOD3;LRRK2;MIR663A;FMN2;FRG2C;ARHGAP6;RYR3;ROBO1;GRM5;DACH1;CA3;ADAMTSL3;PIEZO2;HMCN1;PRKG1;WLS;EPHA5;KIRREL3;CHST9;TRPC5;PRMT8;EPHA6;DSCAM;TRPC4;DCC;SEMA6D;MAGI2;TACR3;UNC5C;NRG1;KSR2;SORCS3;FOXP2;ANO2;PARD3B;KIAA1217;MIR3648;INTS4L1;NRG3;MIR3687;HECW1;LANCL3;CDH13;ERG;COL6A5;PLCB1;ZFPM2;MAPRE2;DGKI;CNTNAP4;OCA2;RTN1;PDE1C;TSHZ2;GLIS3;CACNA1D;LRP2;CACNA1C;HDAC9;SNTG1;LARGE;ADAMTS17;SPOCK3;ANKRD36BP2;HIVEP3;CSMD3;CSMD2;CSMD1;PLXNA4;CORIN;OPCML;GABBR2;CA10;PCDH9;CNTN5;GABRA5;ANKRD30BL;LHFPL3;KY;PGM5P2;FAM155A;FAM135B;DAB1;FGF14;ASXL3;TBXAS1;CPE
H3K27me3 H1-hESC hg19	86/2000	4.303197068596782E-5	PTPRT;RYR2;ATP8A2;SLC35F3;CTNND2;LRRK2;PTPRM;FMN1;MIR663A;ARHGAP6;RYR3;RERG;HAPLN1;GRM1;GRM5;CA3;GRM7;ADAMTSL3;PIEZO2;GPR139;SH3GL2;WLS;EPHA5;TRPC5;EPHA6;DSCAM;GRID1;COL25A1;MAGI2;ARAP2;NRG1;MYRIP;SEZ6L;SYN3;SORCS3;ROCK1P1;ANO2;KIAA1217;CACNB2;INTS4L1;NRG3;CDH13;TTLL11;COL6A5;PLCB1;ZFPM2;DGKI;PDZRN4;RTN1;PDE1C;DNAH8;PON1;CACNA1D;LRP2;CACNA1C;TMEM163;HDAC9;ARHGAP22;RELN;SNTG1;ADAMTS17;SPOCK3;SYNDIG1;GPC5;SRGAP3;CORIN;OPCML;GABBR2;CNTN5;ANKRD30BL;PLCL1;SUSD1;ZNF804A;LHFPL3;VWA3B;KY;ARHGAP24;IGSF11;MSRA;PGM5P2;FAM135B;DAB1;CDK6;CPE;APBA1;CNTN4
H3K27me3 GM12878 hg19	124/3221	5.448487621559055E-5	ATP8A2;CLSTN2;CTNND2;MIR663A;DACH1;NDST3;MACROD2;HMCN1;SAMD12;SH3GL2;PKNOX2;WLS;EPHA5;ABCG8;EPHA6;CACNA2D1;SEMA6D;MAGI2;ANK3;ROCK1P1;FOXP2;KIAA1217;MIR3648;CACNB2;SCN8A;HECW1;TTLL11;ZFPM2;FREM2;KHDRBS2;RTN1;GLIS3;NPAS3;NKAIN2;ARHGAP22;ZMAT4;PLXNA4;GPM6B;LINC00273;SPECC1;DTNA;WSCD1;CNTN5;DNAH14;FRG1B;LRP1B;PTPRD;SDK1;PGM5P2;FAM135B;DAB1;FGF14;NBEA;TBXAS1;CNTN4;XKR4;FBXL7;ROBO2;CNTNAP2;RYR2;SLC35F3;PTPRM;FMN2;RYR3;HAPLN1;ROBO1;TTC28;IQCJ-

			SCHIP1;SGCD;CA3;GRM7;ADAMTSL3;ERC1;ZNF385D;PRKG1;ANKUB1;CHST9;TRPC5;GRID1;COL25A1;DCC;HUNK;TACR3;NRG1;ELOVL7;KSR2;MYRIP;LCA5L;PARD3B;DNM3;PLCB4;NRG3;MIR3687;CDH13;COL6A5;PLCB1;MAPRE2;PDZRN4;OCA2;ACSS3;LAMA2;SATB2;NOTCH2NL;LRP2;TMEM163;HDAC9;RELN;SPOCK3;ANKRD36BP2;GPC5;CTNNA3;SLIT3;CSMD3;RUNX1T1;OPCML;PCDH9;PLCL1;ZNF804A;LHFPL3;MERTK;STXBP5L;AGBL4;FAM155A;ASXL3
H3K9me3 CD14-positive monocyte hg19	85/2000	7.271295727639687E-5	*NCOR1P1;SEMA5A;ALK;RYR2;ATP8A2;CLSTN2;MIR663A;FMN2;RERG;HAPLN1;PTPRG;TTC28;ADAMTSL3;FAM110B;PIEZO2;DLGAP1;GPR139;MACROD2;HMCN1;SH3GL2;PKNOX2;ANKS1B;EPHA5;KIRREL3;CHST9;TRPC5;EPHA6;DSCAM;TRPC4;COL25A1;DCC;CACNA2D1;MAGI2;KAZN;UNC5C;NRG1;MYRIP;SYN3;SORCS3;ROCK1P1;FOXP2;ANO2;KIAA1217;CACNB2;HECW1;CDH13;PRKD1;COL6A5;DSCAML1;DOCK1;FREM2;PDZRN4;ACSS3;LAMA2;GLIS3;CACNA1D;LRP2;HDAC9;RELN;PRRG1;SYNDIG1;HIVEP3;SLIT3;LRRC4C;GPM6B;OPCML;CLVS1;GABBR2;CA10;PCDH9;WSCD1;ATRNL1;RANBP17;LHFPL3;HS3ST4;LRP1B;STXBP5L;PTPRD;SDK1;PGM5P2;FAM135B;DAB1;FRAS1;FGF14;ASXL3*
H3K27me3 heart mm9	84/2000	1.26402185849381E-4	*DPP10;ALK;PTPRT;FHOD3;CNTNAP2;ATP8A2;CSRNP3;CLSTN2;SYCP2;THSD7B;SLC35F3;DGKB;CTNND2;OTUD7A;GRIK4;SLC35F1;MIR96;RERG;GRM1;GRM5;GRM7;GPR139;SOX6;SH3GL2;ANKS1B;PCDHAC1;EPHA5;TRPC5;EPHA6;HFM1;DSCAM;TRPC4;COL25A1;DCC;CACNA2D1;HUNK;COBL;UNC5C;NRG1;UNC5D;ELOVL7;KSR2;SEZ6L;FOXP2;ANO2;TIAM2;NRG3;SCN8A;DSCAML1;DGKI;SHANK2;KHDRBS2;RTN1;SATB2;ADCY2;LRP2;LRRC16A;NKAIN2;RELN;SNTG1;SPOCK3;SYNDIG1;GPC5;SLIT3;CSMD3;ASIC2;CSMD1;TRPM3;GPM6B;OPCML;PCDH9;WSCD1;CNTN5;SUSD1;LHFPL3;SGCZ;HS3ST4;STXBP5L;SDK1;FAM135B;DAB1;FRAS1;MIR183;XKR4*
H3K9me3 osteoblast hg19	84/2000	1.2188782206904598E-4	*GABRB3;DPP10;PTPRT;CNTNAP2;RYR2;ZNF493;CLSTN2;SYCP2;THSD7B;CTNND2;MIR663A;ARHGAP6;IQCJ-SCHIP1;CDH4;DACH1;LIPI;ADAMTSL3;DLGAP1;GPR139;MACROD2;TBC1D22A;PCDHAC1;CALN1;TRPC5;PACRG;SNRPN;DSCAM;COL25A1;SEMA6D;ARAP2;PCDHA13;MYRIP;PCDHA12;ROCK1P1;PCDHA11;CNBD1;KIAA1217;MIR3648;SPATA16;NRG3;MIR3687;HECW1;ELMO1;TTLL11;ASTN2;SHANK2;CNTNAP4;KHDRBS2;TSHZ2;PON1;GLIS3;ADCY2;SEL1L2;NKAIN2;PCDHA1;SNTG1;LARGE;PCDHA4;PCDHA3;PCDHA2;FPGT-TNNI3K;PCDHA9;LRRC4C;PCDHA8;PCDHA7;TRPM3;ZNF585A;OPCML;CLVS1;CA10;MUC16;GABRA5;ANKRD30BL;DNAH14;GABRA3;RANBP17;ZNF804A;HS3ST4;PTPRD;SDK1;DAB1;MIR1324;ASXL3;XKR4*
H3K27me3 Caco-2 hg19	26/380	1.2470891577944152E-4	*ROBO2;LRRK2;MIR663A;TMEM163;HDAC9;HAPLN1;ARHGAP22;DACH1;LARGE;SPOCK3;PLXNA4;PRKG1;LINC00273;ANKRD30BL;PLCL1;ELOVL7;VWA3B;KSR2;FOXP2;LRP1B;MIR3648;DNM3;CACNB2;NBEA;MIR3687;PRKD1*
H3K27me3 MCF-7 hg19	48/989	4.007570528643969E-4	*ROBO2;RTN1;LRRK2;NTM;NRXN3;SLC35F1;LRP2;ARHGAP15;HDAC9;NPAS3;NKAIN2;RELN;SPOCK3;GPC5;SLIT3;CSMD3;PLXNA4;ADAMTS6;PRKG1;ANKUB1;OPCML;LINC00273;KIRREL3;CNTN5;AUTS2;ANKRD30BL;TRPC4;CACNA2D1;PLCL1;ZNF804A;UNC5C;LHFPL3;MYRIP;SEZ6L;ROCK1P1;FOXP2;ANO2;STXBP5L;PTPRD;HECW1;CDC42EP3;CDH13;PRKD1;CNTN4;ZFPM2;MAPRE2;FBXL7;PDZRN4*
H3K9me3 CH12.LX mm9	102/2689	7.619132858975772E-4	*FHOD3;CLSTN2;ANKRD36;CTNND2;SLC8A1;DACH1;LIPI;PCDHAC1;EPHA5;EPHA6;DSCAM;CACNA2D1;MAGI2;FNDC3B;UNC5C;PCDHA12;PCDHA11;FOXP2;PCDHA10;SCN8A;PRKD1;ASTN2;ZFPM2;FREM2;KHDRBS2;CTBP2;TSHZ2;PCDH15;TMTC1;NKAIN2;ARHGAP22;PCDHA1;ZMAT4;LARGE;ADAMTS17;PCDHA5;SYNDIG1;PCDHA4;PCDHA3;PCDHA2;PCDHA9;LRRC4C;PCDHA8;PCDHA7;PCDHA6;WSCD1;GAREM;RANBP17;PTPRD;SDK1;DAB1;NBEA;MYO5B;CNTN4;XKR4;FBXL7;GABRB3;DPP10;ALK;PTPRT;CNTNAP2;RYR2;CSRNP3;SYCP2;TENM4;LRRK2;OTUD7A;PTPRM;SLC35F1;FMN2;PTPRG;ADAMTSL1;GRM7;PRMT8;GRID1;DCC;HUNK;NRG1;MYRIP;UPK3B;TRDN;NRG3;DOCK1;PDZRN4;CNTNAP4;SLC24A2;ACSS3;SGMS1;LAMA2;SEMA3D;ADCY2;RELN;SPOCK3;SLIT3;GPC6;RUNX1T1;GABBR2;PCDH9;SUSD1;SGCZ;NFIA;ASXL3*
H3K27me3 fibroblast of dermis hg19	77/2000	0.004475074079206002	*GABRB3;ALK;ROBO2;RYR2;ATP8A2;CLSTN2;CTNND2;MIR663A;SLC35F1;IQCJ-SCHIP1;DACH1;CA3;GRM7;ADAMTSL3;NDST3;DLGAP1;NCKAP5;PCDHAC1;ANKUB1;EPHA5;PACRG;EPHA6;HFM1;ZNF283;COL25A1;ARAP2;COBL;UNC5C;NRG1;UNC5D;ELOVL7;KSR2;MYRIP;SEZ6L;SORCS3;ROCK1P1;KIAA1217;MIR3648;NRG3;MIR3687;ELMO1;ASTN2;PDZRN4;PON3;RTN1;*

			PDE1C;NTM;ATP1A4;ADCY2;LRP2;TMEM163;EHBP1;NKAIN2;ZMAT4;ADAMTS17;SYNDIG1;GPC5;CTNNA3;GPC6;SPECC1;DTNA;WSCD1;CNTN5;GABRA5;ATRNL1;ANKRD30BL;ZNF804A;LHFPL3;MERTK;SGCZ;STXBP5L;PTPRD;DAB1;FRAS1;MIR1324;CNTN4;XKR4
H3K27me3 myotube hg19	75/2000	0.011073335772249825	*GABRB3;PTPRT;ROBO2;CNTNAP2;RYR2;CTNND2;LRRK2;MIR663A;SLC35F1;FRG2C;ARHGAP6;ROBO1;TTC28;GRM5;SPTLC2;GRM7;DLGAP1;MIR4461;PCDHAC1;TRPC5;EPHA6;DSCAM;COL25A1;DCC;VPS13D;ARAP2;TACR3;COBL;UNC5D;KSR2;ROCK1P1;PCDHA10;KIAA1217;MIR3648;DNM3;INTS4L1;ANKFN1;NRG3;MIR3687;LANCL3;TTLL11;KHDRBS2;SATB2;TSHZ2;PON1;PAK1;SNTG1;SPOCK3;SPOCK1;GPC5;CTNNA3;PCDHA2;CSMD3;PCDHA9;CSMD1;CORIN;OPCML;CNTN5;GABRA5;GABRA3;ZNF804A;LHFPL3;FRG1B;SGCZ;HS3ST4;B3GALTL;PTPRD;MGAT4C;AGBL4;SDK1;FAM155A;FAM135B;MIR1324;CNTN4;XKR4*
H3K27me3 spleen mm9	73/2000	0.025840964791690763	*GABRB3;SEMA5A;FHOD3;ATP8A2;EDA;CLSTN2;TENM4;SLC35F3;CTNND2;OTUD7A;SLC35F1;BICD1;TTC28;FAM110B;MACROD2;ERC1;HMCN1;SOX6;PRKG1;ANKS1B;EPHA5;PRMT8;EPHA6;HFM1;GRID1;CACNA2D1;SEMA6D;FNDC3B;COBL;UNC5C;ELOVL7;KSR2;NAV2;MYRIP;SEZ6L;SORCS3;DNM3;CACNB2;TBC1D5;LANCL3;COL6A5;ASTN2;ZFPM2;DSCAML1;DOCK1;FREM2;DGKI;SLC24A2;RTN1;TSHZ2;GLIS3;LRP2;NPAS3;NKAIN2;RELN;LARGE;SYNDIG1;SPOCK1;N4BP2;TRPM3;GABRA5;PLCL1;MERTK;KY;STXBP5L;IGSF11;PTPRD;SDK1;DAB1;NBEA;CPE;XKR4;FBXL7*
H3K27me3 small intestine mm9	71/2000	0.056829024178286676	*ALK;PTPRT;RYR2;CLSTN2;TENM4;SLC35F3;CTNND2;SLC35F1;TTC28;PIEZO2;HMCN1;SOX6;NCKAP5;EPHB1;ADAMTS6;PRKG1;ANKS1B;EPHA5;PRMT8;DSCAM;GRID1;COL25A1;CACNA2D1;HUNK;COBL;UNC5C;UNC5D;KSR2;NAV2;CACNB2;NRG3;HECW1;CDH13;PRKD1;ASTN2;ST6GALNAC3;DSCAML1;FREM2;DGKI;RTN1;SATB2;GLIS3;ADCY2;LRP2;NPAS3;NKAIN2;RELN;SNTG1;LARGE;SYNDIG1;SPOCK1;SLIT3;CSMD3;PLXNA4;GPM6B;CORIN;RUNX1T1;GABBR2;WSCD1;PLCL1;HS3ST4;STXBP5L;IGSF11;PTPRD;FAM155A;DAB1;NSG2;ASXL3;CPE;APBA1;XKR4*
H3K27me3 kidney mm9	60/1675	0.08994934188316696	*DPP10;PTPRT;ROBO2;RYR2;THSD7B;TENM4;CTNND2;GRIK4;FMN2;RERG;HAPLN1;ROBO1;GRM7;SOX6;EPHB1;PRKG1;PKNOX2;KIRREL3;PRMT8;HFM1;DSCAM;GRID1;COL25A1;CACNA2D1;UNC5D;MYRIP;SEZ6L;FOXP2;STIM1;NRG3;SCN8A;HECW1;ELMO1;ASTN2;ZFPM2;DSCAML1;DGKI;SATB2;TMEM163;NPAS3;RELN;ZMAT4;SYNDIG1;SPOCK1;GPC5;SLIT3;CSMD3;RUNX1T1;GABBR2;AUTS2;LHFPL3;SGCZ;HS3ST4;ARHGAP24;FAM155A;FAM135B;DLG2;NFIA;CPE;FBXL7*
H3K9me3 brain mm9	23/522	0.12040134937236453	*PTPRT;INVS;RTN1;GABRA3;LHFPL3;MYRIP;PCDHA12;SGCZ;PCDHA11;PCDHA10;GRM1;DACH1;PCDHA1;MYO5B;PCDHA5;PCDHA4;PCDHA3;PCDHA2;PCDHA9;XKR4;PCDHA8;PCDHA7;PCDHA6*
H3K9me3 MCF-7 hg19	11/190	0.12573571338237605	*MIR3648;LINC00273;PCDHA1;MIR3687;ANKRD30BL;RANBP17;MIR663A;PCDHA3;PCDHA2;ROCK1P1;CALN1*
H3K9me3 fibroblast of lung hg19	77/2323	0.17833668639811315	*NCOR1P1;DPP10;PTPRT;CNTNAP2;THSD7B;CTNND2;MIR663A;FRG2C;BACH1;ROBO1;ADAMTSL1;ZNF280D;GRM7;ADAMTSL3;DLGAP1;GPR139;MACROD2;KIFAP3;WLS;PCDHAC1;CALN1;CHST9;SNRPN;EPHA6;DSCAM;KAZN;PCDHA13;PCDHA12;ROCK1P1;TEKT4P2;PCDHA11;PCDHA10;MIR3648;SPATA16;ZNF717;MIR3687;DPYD;PRKD1;ST6GALNAC3;CNTNAP4;SLC24A2;OCA2;KHDRBS2;PDE1C;ADCY2;TPTE;THSD4;NKAIN2;PCDHA1;LARGE;ADAMTS17;PCDHA5;PCDHA4;PCDHA3;PCDHA2;CSMD3;FPGT-TNNI3K;CSMD2;PCDHA8;CSMD1;PCDHA7;PCDHA6;LINC00478;LINC00273;CA10;ANKRD30BL;GABRA3;RANBP17;SUSD1;FRG1B;PTPRD;AGBL4;DAB1;NFIA;MIR1324;CNTN4;XKR4*
H3K27me3 A549 hg19	93/2891	0.19108460868267194	*PTPRT;ROBO2;CSRNP3;EDA;CLSTN2;CTNND2;PTPRM;MIR663A;FMN2;FRG2C;PTPRG;ROBO1;CDH4;GRM5;RPS6KA5;SPTLC2;GRM7;NDST3;FAM110B;MACROD2;ZNF385D;SAMD12;KIFAP3;NCKAP5;PCDHAC1;EPHA5;KIRREL3;ZSCAN5A;PRMT8;DCC;SEMA6D;MAGI2;ARAP2;HUNK;TACR3;COBL;UNC5C;ANK2;UNC5D;ELOVL7;KSR2;ANK3;ROCK1P1;RUNX1;CNBD1;KIAA1217;INTS4L2;CACNB2;INTS4L1;SPATA16;NRG3;SCN8A;LANCL3;ZFPM2;DSCAML1;MAPRE2;DGKI;KHDRBS2;ACSS3;SEL1L2;HDAC9;ZMAT4;SPOCK3;SPOCK1;FSD2;GPC5;CTNNA3;CSMD3;PCDHA9;GPC6;ZNF585A;OPCML;LINC00273;CA10;DTNA;CNTN5;AUTS2;ATRNL1;GABRA3;ZNF804A;LHFPL3;FRG1B;MERTK;PTPRD;SLC2A9;AGBL4;DLG2;MIR1324;DLC1;TBXAS1;FAT3;CNTN4;FBXL7*
H3K4me3 HEK293 hg19	68/2116	0.4296956506769359	*ROBO2;AVEN;CNTNAP2;RYR2;PLEKHB2;CTNND2;LRRK2;MAST2;BICD1;RYR3;AKAP13;HMCN1;DIP2C;NCKAP5;PRKG*

			1;SH3GL2;ANKS1B;KLF12;DSCAM;DCC;MAGI2;HUNK;COBL;PCDHA13;MYRIP;TEKT4P2;PCDHA10;PARD3B;KIAA1217;PLCB4;STIM1;ZNF717;LANCL3;WDPCP;ASTN2;PLCB1;MAPRE2;DOCK1;RTN1;TSHZ2;NOTCH2NL;CACNA1D;CACNA1C;LRRC16A;ARHGAP22;RELN;SLIT3;CSMD3;PLXNA4;RUNX1T1;WSCD1;CNTN5;AUTS2;ANKRD30BL;MGMT;LRBA;VWA3B;PTPRD;SDK1;PGM5P2;FAM155A;CDK6;NFIA;ASXL3;MYO5B;CPE;XKR4;FBXL7
H3K27me3 GM06990 hg19	9/179	0.4526379334727813	*MIR3648;LINC00273;FGF14;MIR3687;ANKRD30BL;ASXL3;FMN1;TMEM163;TTC28*
H3K27me3 testis mm9	64/2000	0.4986696530664332	*DPP10;ROBO2;CNTNAP2;ATP8A2;TENM4;DGKB;LRRK2;GRIK4;SLC35F1;FMN2;PPP3CA;CDH4;WLS;PRMT8;GRID1;TRPC4;CACNA2D1;IFNGR2;ARAP2;HUNK;UNC5C;NRG1;ELOVL7;SEZ6L;SORCS3;PCDHA11;FOXP2;PARD3B;TIAM2;NRG3;SCN8A;CDH13;TTLL11;PRKD1;ASTN2;ST6GALNAC3;DSCAML1;FREM2;DGKI;SEMA3D;ADCY2;TMEM163;NPAS3;PCDHA4;SPOCK1;GPC5;SLIT3;CSMD3;ASIC2;PLXNA4;PCDHA6;CCDC178;GABBR2;AUTS2;ATRNL1;PLCL1;GAREM;MERTK;FAM155A;DAB1;FRAS1;FGF14;MYO5B;CPE*
H3K27me3 NT2-D1 hg19	63/2000	0.6434569815211207	*ATP8A2;LRRK2;MIR663A;MIR96;RYR3;RERG;HAPLN1;GRM1;EFTUD1;GRM7;PIEZO2;GPR139;SH3GL2;CSGALNACT1;KIRREL3;CHST9;PACRG;KLF12;EPHA6;TRPC4;DCC;MAGI2;NRG1;ANK3;ANO2;PARD3B;KIAA1217;DNM3;INTS4L1;LANCL3;CDH13;COL6A5;PLCB1;MAPRE2;DGKI;PDZRN4;CNTNAP4;RTN1;PDE1C;DNAH8;GLIS3;TMTC1;PRDM11;ARHGAP22;RELN;SPOCK3;CSMD3;GPM6B;CORIN;OPCML;DYNC1H1;GABRA5;ANKRD30BL;ZNF804A;LHFPL3;VWA3B;SLC2A9;AGBL4;SDK1;FAM135B;DAB1;CPE;MIR183*
H4K20me1 A549 hg19	61/2000	1.0	*INVS;PTPRT;ROBO2;ATP8A2;EDA;CLSTN2;CTNND2;MIR663A;BACH1;RERG;HAPLN1;IQCJ-SCHIP1;DACH1;ADAMTSL3;PIEZO2;MIR4461;EPHB1;PCDHAC1;EPHA5;EPHA6;GRID1;HUNK;UNC5C;NRG1;NAV2;MYRIP;SEZ6L;ROCK1P1;FOXP2;ANO2;KIAA1217;MIR3648;DNM3;PCP4;NCOR1;STIM1;MIR3687;HECW1;PRKD1;DGKI;GLIS3;LRP2;CACNA1C;HDAC9;NKAIN2;ARHGAP22;RELN;LARGE;FSD2;SRGAP3;CSMD2;PLCL1;RANBP17;VWA3B;KY;STXBP5L;SDK1;MCM3AP;FRAS1;APBA1;XKR4*
H3K4me1 megakaryocyte mm9	74/2505	1.0	*DPP10;ALK;FHOD3;ATP8A2;SYCP2;TENM4;SLC35F3;DGKB;LRRK2;OTUD7A;GRIK4;PTPRM;FMN1;TTC28;CDH4;GRM5;SGCD;FAM110B;KIFAP3;SH3GL2;ANKS1B;WLS;PCDHAC1;KIRREL3;DSCAM;COL25A1;CACNA2D1;MAGI2;FNDC3B;DIO2;HUNK;TACR3;COBL;KAZN;UNC5D;KSR2;MYRIP;SUMF1;LCA5L;VWA8;IFT43;NRG3;SCN8A;DPYD;LANCL3;TTLL11;PRKD1;DSCAML1;ATP6V0D2;DGKI;KHDRBS2;LRP2;SEL1L2;FHIT;SYNDIG1;GPC5;CORIN;OPCML;PCDH9;ATRNL1;PLCL1;GAREM;MERTK;STXBP5L;IGSF11;PTPRD;AGBL4;SDK1;DAB1;MYO5B;CPE;XKR4;FBXL7;CCDC171*
H3K9me3 mammary epithelial cell hg19	60/2000	1.0	*GABRB3;DPP10;PTPRT;CNTNAP2;ZNF493;MIR663A;AFF3;ARHGAP6;ADAMTSL1;ZNF280D;CDH4;PIEZO2;DLGAP1;GPR139;MACROD2;SH3GL2;CALN1;PACRG;SNRPN;SEMA6D;HUNK;PCDHA13;PCDHA12;ROCK1P1;TEKT4P2;PCDHA11;PCDHA10;MIR3648;ZNF717;MIR3687;DUX4L4;ASTN2;OCA2;KHDRBS2;SATB2;ADCY2;PCDHA1;PCDHA5;PCDHA4;PCDHA3;PCDHA2;PCDHA9;LRRC4C;PCDHA8;PCDHA7;PCDHA6;OPCML;MUC16;GABRA5;ANKRD30BL;GABRA3;RANBP17;HS3ST4;PTPRD;SDK1;FAM155A;DAB1;MIR1324;CNTN4;FBXL7*
H3K27me3 brain mm9	60/2000	1.0	*SEMA5A;PTPRT;ATP8A2;SLC35F3;CTNND2;LRRK2;GRM5;ADAMTSL3;HMCN1;SH3GL2;PCDHAC1;KIRREL3;TRPC5;EPHA6;GRID1;CACNA2D1;IFNGR2;SEMA6D;FNDC3B;ARAP2;HUNK;KSR2;NAV2;PCDHA10;CACNB2;STIM1;CDC42EP3;DPYD;ELMO1;LANCL3;DOCK1;DGKI;PDZRN4;SHANK2;SKAP2;CNTNAP4;ACSS3;CTBP2;LRP2;LRRC16A;TMEM163;LARGE;SYNDIG1;CSMD3;PLXNA4;TRPM3;GPM6B;OPCML;GABBR2;LRBA;MERTK;KY;PTPRD;SDK1;FAM135B;DAB1;CDK6;MYO5B;EIF4G3;FBXL7*
H3K4me3 myotube hg19	60/2000	1.0	*SPAG16;AVEN;CNTNAP2;EDA;SLC35F3;FMN1;RERG;PPP3CA;DLGAP1;SAMD12;TBC1D22A;ANKS1B;TRPC4;MAGI2;TACR3;NRG1;FAM126B;FOXP2;ZNF717;HECW1;WDPCP;ERG;SKAP2;WDR27;ACSS3;RTN1;SGMS1;TSHZ2;DNAH6;LRP2;CACNA1C;HDAC9;MIPOL1;NPAS3;ARHGAP22;ADAMTS17;PCDHA3;FSD2;FPGT-TNNI3K;PCBD2;N4BP2;PCDHA7;PLXNA4;MDN1;PCDHA6;SPECC1;AUTS2;MGMT;LRBA;COL22A1;ZNF804A;TRAPPC8;LHFPL3;ELP4;KY;AGBL4;SDK1;PGM5P2;DIAPH3;CPE*
H3K9me3 mononuclear cell hg19	8/198	1.0	*PCDHA1;RANBP17;PCDHA3;PCDHA2;MACROD2;PCDHA12;SHANK2;PCDHA6*
H3K9me3 T-	59/2000	1.0	*NCOR1P1;SEMA5A;SPAG16;INVS;CNTNAP2;PTPRM;MIR66*

cell acute lymphoblastic leukemia hg19			*3A;SLC35F1;ZNF280D;SPTLC2;GRM7;ADAMTSL3;HMCN1;ZNF385D;MIR4461;EPHA5;TRPC5;PACRG;EPHA6;ZNF283;GRID1;COL25A1;SEMA6D;MAGI2;UNC5C;NRG1;UNC5D;MYRIP;SORCS3;ROCK1P1;TEKT4P2;FOXP2;ANKFN1;ELMO1;WDPCP;PRKD1;DGKI;SHANK2;KHDRBS2;ACSS3;ADCY2;SEL1L2;HDAC9;THSD4;ANKRD36BP2;PCDHA3;HIVEP3;GPC6;SPECC1;CA10;PCDH9;RANBP17;HS3ST4;LRP1B;SLC2A9;FAM135B;MCM3AP;DAB1;MYO5B*
H3K9me3 myotube hg19	59/2000	1.0	*RYR2;CLSTN2;MIR663A;SLC35F1;FRG2C;PTPRG;ADAMTSL1;ZNF280D;ADAMTSL3;GPR139;MACROD2;HMCN1;SH3GL2;PKNOX2;PCDHAC1;CSGALNACT1;KIRREL3;PRMT8;TRPC4;COL25A1;DCC;SEMA6D;KAZN;PCDHA13;PCDHA12;ROCK1P1;PCDHA11;ANO2;PCDHA10;MIR3648;DNM3;ANKFN1;NRG3;ZNF717;MIR3687;ZFPM2;SHANK2;PON3;KHDRBS2;DNAH8;PON1;HDAC9;THSD4;ARHGAP22;PCDHA1;SH3TC2;PCDHA5;PCDHA4;PCDHA3;PCDHA2;CSMD2;PCDHA8;PCDHA7;PCDHA6;RANBP17;SUSD1;VWA3B;FRG1B;FAM135B*
H3K4me3 astrocyte of the spinal cord hg19	58/1965	1.0	*ROBO2;CNTNAP2;PLEKHB2;CTNND2;MAST2;MIR663A;AKAP13;DACH1;SAMD12;PRKG1;SH3GL2;EPHA5;DSCAM;COL25A1;DCC;TACR3;KAZN;PARD3B;PLCB4;ZNF717;MIR3687;TTLL11;PRKD1;ASTN2;PLCB1;DOCK1;FREM2;ACSS3;CTBP2;CUL5;DNAH8;TSHZ2;NOTCH2NL;NPAS3;RELN;ZMAT4;ADAMTS17;SYNDIG1;ANKRD36BP2;PCDHA3;LRRC4C;PLXNA4;RUNX1T1;WSCD1;CNTN5;AUTS2;PLCL1;DNAH14;ZNF804A;FRG1B;PTPRD;SDK1;DAB1;FRAS1;NFIA;ASXL3;CPE;EIF4G3*
H3K4me1 embryonic fibroblast mm9	58/2000	1.0	*GABRB3;PLEKHB2;SLC35F3;DGKB;CPQ;HAPLN1;KYNU;PIEZO2;SH3GL2;PCDHAC1;TRPC5;HFM1;DSCAM;TRPC4;TACR3;KAZN;NRG1;ANK2;NAV2;SEZ6L;SYN3;SORCS3;ANO2;RUNX1;MYO3B;SCN8A;COL6A5;UTRN;ST6GALNAC3;CNTNAP4;PON3;KHDRBS2;RTN1;PDE1C;LAMA2;SEMA3D;PCDH15;LRP2;TMEM163;NPAS3;PAK1;DHX32;SH3TC2;ZMAT4;HIVEP3;ASIC2;OPCML;DTNA;WSCD1;MGMT;SUSD1;LHFPL3;LRP1B;ARHGAP24;MSRA;FRAS1;NSG2;ASXL3*
H3K4me3 WERI-Rb-1 hg19	47/1606	1.0	*INVS;ATP8A2;PLEKHB2;PDE1C;SLC35F3;TSHZ2;NOTCH2NL;BICD1;MRPL33;PTPRG;AKAP13;GRM5;GRM7;FAM110B;SPOCK3;ANKRD36BP2;N4BP2;PCDHA7;GPM6B;SH3GL2;EPHA5;CHST9;HFM1;AUTS2;PLCL1;HUNK;NRG1;PCDHA13;MYRIP;FRG1B;TEKT4P2;KY;PARD3B;KIAA1217;SDK1;PGM5P2;CDK6;MYO3B;ZNF717;CDC42EP3;TBXAS1;TTLL11;PRKD1;XKR4;ZNF675;EIF4G3;PDZRN4*
H3K4me1 mammary epithelial cell hg19	57/2000	1.0	*ATP8A2;CTNND2;SLC35F1;RYR3;CA3;DLGAP1;HMCN1;ANKS1B;ANKUB1;KIRREL3;PACRG;COL25A1;HUNK;SYN3;UPK3B;SUMF1;CACNB2;PCP4;MYO3B;SCN8A;PLCB1;ATP6V0D2;FREM2;DGKI;OCA2;RTN1;PDE1C;LAMA2;DNAH8;TSHZ2;DNAH6;GLIS3;PCDH15;NKAIN2;RELN;SNTG1;ANKRD36BP2;N4BP2;SPECC1;DYNC1H1;DTNA;CNTN5;COL22A1;DNAH14;SUSD1;EXOC6B;ZNF804A;LHFPL3;VWA3B;KY;STXBP5L;PTPRD;SDK1;FRAS1;DLG2;CPE;XKR4*
H3K27me3 thymus mm9	57/2000	1.0	*ALK;FHOD3;RYR2;SLC35F3;GRIK4;PTPRM;FMN2;CDH4;SOX6;PRKG1;SH3GL2;GRID1;SEMA6D;FNDC3B;HUNK;ELOVL7;NAV2;MYRIP;SEZ6L;FOXP2;TIAM2;NRG3;SCN8A;HECW1;ASTN2;DSCAML1;DGKI;RTN1;CTBP2;GLIS3;LRP2;LRRC16A;TMEM163;NPAS3;RELN;SNTG1;LARGE;HIVEP3;SLIT3;PCDHA2;PLXNA4;CORIN;RUNX1T1;GABBR2;GAREM;RANBP17;MERTK;HS3ST4;STXBP5L;IGSF11;FAM155A;FRAS1;NBEA;MYO5B;CPE;APBA1;XKR4*
H3K4me3 kidney epithelial cell hg19	55/1936	1.0	*CNTNAP2;ZNF493;ATP8A2;PLEKHB2;MAST2;BICD1;RYR3;AKAP13;HMCN1;DIP2C;NCKAP5;PRKG1;SH3GL2;ANKS1B;TRPC4;COL25A1;DCC;MAGI2;HUNK;MYRIP;PLCB4;STIM1;ZNF717;TTLL11;PRKD1;ASTN2;MAPRE2;DOCK1;RTN1;CTBP2;DNAH8;TSHZ2;NOTCH2NL;NRXN3;CACNA1C;NPAS3;RELN;ANKRD36BP2;WSCD1;CNTN5;AUTS2;LRBA;DNAH14;PTPRD;SDK1;PGM5P2;FAM155A;FGF14;NFIA;ASXL3;CPE;CNTN4;XKR4;EIF4G3;FBXL7*
H3K9me3 skeletal muscle myoblast hg19	56/2000	1.0	*CLSTN2;SYCP2;SLC35F3;FMN1;MIR663A;FRG2C;BACH1;ARHGAP6;GRM7;PIEZO2;DLGAP1;MACROD2;SH3GL2;PCDHAC1;EPHA5;PRMT8;ZNF283;DSCAM;SEMA6D;UNC5D;PCDHA13;SEZ6L;ROCK1P1;FOXP2;ANO2;DNM3;ANKFN1;ZNF717;TTLL11;ERG;ASTN2;UTRN;ST6GALNAC3;MAPRE2;PDZRN4;SKAP2;PON3;CUL5;PDE1C;TPTE;PCDHA1;PCDHA7;ZNF585A;GABBR2;RANBP17;SUSD1;TRAPPC8;VWA3B;FRG1B;PTPRD;AGBL4;FAM155A;MCM3AP;MIR1324;CNTN4;XKR4*
H3K4me2 mammary epithelial cell hg19	56/2000	1.0	*RYR2;SLC35F1;RERG;DLGAP1;ERC1;NCKAP5;PCDHAC1;GRID1;COL25A1;HUNK;TACR3;PCDHA13;MYRIP;UPK3B;KIAA1217;CACNB2;INTS4L1;PCP4;NRG3;ZNF717;ST6GALNAC3;FREM2;DGKI;CNTNAP4;ACSS3;RTN1;LAMA2;DNAH8;DNAH6;CACNA1D;CACNA1C;NPAS3;ADAMTS17;PCDHA3;DMD;GPC5;PLXNA4;ZNF585A;GABBR2;ATRNL1;MGMT;PLCL1*

			;ZNF804A;VWA3B;ELP4;MERTK;SGCZ;KY;PTK2;AGBL4;SDK1;PGM5P2;ASXL3;CPE;CNTN4;FBXL7
H3K4me3 skeletal muscle cell hg19	49/1754	1.0	*MAST2;SLC35F1;ARHGAP6;RYR3;GPHN;AKAP13;DACH1;FAM110B;DLGAP1;HMCN1;SAMD12;PRKG1;ANKS1B;HFM1;DSCAM;TRPC4;DCC;TACR3;UNC5D;PARD3B;ZNF717;FREM2;OCA2;NOTCH2NL;TMTC1;CACNA1D;NPAS3;PCDHA3;CSMD3;PLXNA4;PCDHA6;DTNA;AUTS2;ATRNL1;LHFPL3;FRG1B;KY;B3GALTL;PTPRD;AGBL4;PGM5P2;FAM155A;CDK6;FRAS1;NFIA;MYO5B;NF1;CPE;EIF4G3*
H3K4me3 epithelial cell of proximal tubule hg19	46/1655	1.0	*CTBP2;LRRK2;NOTCH2NL;NRXN3;ARHGAP6;BICD1;RYR3;NPAS3;PTPRG;AKAP13;SYNDIG1;PCDHA3;PCDHA2;SRGAP3;NCKAP5;PLXNA4;ANKS1B;PCDHA6;AUTS2;COL25A1;DCC;LRBA;PLCL1;HUNK;PHKB;UNC5D;PCDHA13;FRG1B;TEKT4P2;PCDHA10;PARD3B;PTPRD;AGBL4;SDK1;PGM5P2;CDK6;FRAS1;DLG2;IFT43;NFIA;ZNF717;CPE;TTLL11;XKR4;EIF4G3;PDZRN4*
H3K9me3 megakaryocyte mm9	80/2939	1.0	*GABRB3;ALK;SPAG16;SYCP2;ANKRD36;CTNND2;PTPRM;SLC35F1;ARHGAP6;GRM1;ROBO1;CDH4;DLGAP1;NCKAP5;SH3GL2;PCDHAC1;CALN1;KLF12;EPHA6;COL25A1;CACNA2D1;MAGI2;TACR3;COBL;UNC5C;ANK3;MYRIP;PCDHA12;UPK3B;PCDHA11;FOXP2;PCDHA10;DNM3;SPATA16;ANKFN1;SCN8A;HECW1;ELMO1;LANCL3;PRKD1;TLN2;FREM2;PDZRN4;SLC24A2;PON3;KHDRBS2;NTM;TSHZ2;PCDH15;ATP1A4;NRXN3;NPAS3;NKAIN2;PCDHA1;SNTG1;ZMAT4;PCDHA5;SYNDIG1;PCDHA4;PCDHA3;PCDHA2;PCDHA9;PCDHA8;GPC6;PCDHA7;GPM6B;PCDHA6;OPCML;CCDC178;COL22A1;SGCZ;LRP1B;SDK1;FAM155A;FAM135B;DAB1;FGF14;MYO5B;FAT3;XKR4*
H3K4me3 cardiac muscle cell hg19	56/2087	1.0	*CNTNAP2;PLEKHB2;MAST2;ANKRD20A1;ARHGAP6;HAPLN1;ADAMTSL1;AKAP13;ADAMTSL3;DLGAP1;HMCN1;NCKAP5;PRKG1;ANKS1B;COL25A1;DCC;MAGI2;COBL;MYRIP;PARD3B;RUNX1;INTS4L1;PLCB4;STIM1;NRG3;ZNF717;CDC42EP3;TTLL11;DOCK1;FREM2;CTBP2;DNAH8;TSHZ2;NOTCH2NL;NPAS3;NKAIN2;RELN;SYNDIG1;PCDHA3;PCDHA6;CNTN5;AUTS2;LRBA;PHKB;LHFPL3;KY;AGBL4;SDK1;PGM5P2;FAM155A;CDK6;NFIA;ASXL3;MYO5B;CPE;FBXL7*
H3K27me3 cerebellum mm9	40/1486	1.0	*ALK;ACSS3;TENM4;SATB2;GLIS3;GRIK4;PTPRM;ADCY2;LRP2;NPAS3;GRM5;SNTG1;PIEZO2;GPR139;HMCN1;SOX6;PRKG1;CORIN;EPHA5;GABRA5;ATRNL1;GRID1;COL25A1;FNDC3B;HUNK;NRG1;MERTK;SORCS3;HS3ST4;FOXP2;ANO2;PARD3B;FRAS1;MYO5B;DPYD;CDH13;DOCK1;FBXL7;PDZRN4;SKAP2*
H3K4me3 cardiac mesoderm hg19	99/3727	1.0	*FHOD3;CTNND2;MIR663A;EFTUD1;DACH1;DIP2C;SAMD12;SH3GL2;PCDHAC1;EPHA5;KIRREL3;EPHA6;DSCAM;PCDHA13;PCDHA12;PCDHA10;KIAA1217;INTS4L1;ELMO1;WDPCP;TTLL11;PRKD1;ASTN2;CUL5;PDE1C;TSHZ2;TMTC1;CACNA1D;CACNA1C;NPAS3;NKAIN2;PCDHA2;SRGAP3;LRRC4C;PCDHA7;PLXNA4;TRPM3;WSCD1;AUTS2;LRBA;DNAH14;PHKB;KY;ARHGAP24;SDK1;NBEA;MYO5B;NF1;CPE;XKR4;EIF4G3;GABRB3;ROBO2;AVEN;CNTNAP2;CSRNP3;SLC35F3;MAST2;RYR3;ROBO1;ZNF280D;AKAP13;DLGAP1;PRKG1;PACRG;COL25A1;DCC;TACR3;KSR2;MYRIP;ARID1B;PARD3B;PLCB4;IFT43;MIR3687;LANCL3;PLCB1;DOCK1;PDZRN4;OCA2;DNAH8;NOTCH2NL;ENOX2;CSMD3;CSMD1;GPC6;CORIN;CLVS1;PCDH9;GABRA5;MGMT;PLCL1;GABRA3;ZNF804A;LHFPL3;FAM155A;FRAS1;NFIA;ASXL3*
H3K4me3 retinal pigment epithelial cell hg19	54/2021	1.0	*ROBO2;CTNND2;MAST2;ARHGAP6;RYR3;ZNF280D;DACH1;DLGAP1;HMCN1;PRKG1;SH3GL2;SNRPN;HFM1;COL25A1;DCC;SEMA6D;TACR3;RUNX1;INTS4L1;PCP4;PLCB4;ZNF717;TTLL11;PRKD1;PLCB1;DOCK1;OCA2;TSHZ2;NOTCH2NL;PCDH15;CACNA1C;NPAS3;ANKRD36BP2;SRGAP3;GPC6;PLXNA4;RUNX1T1;WSCD1;AUTS2;COL22A1;ZNF804A;LHFPL3;VWA3B;KY;PTPRD;SDK1;DAB1;FRAS1;NFIA;ASXL3;NF1;CPE;XKR4;EIF4G3*
H3K4me2 myotube hg19	53/2000	1.0	*CNTNAP2;ATP8A2;CSRNP3;EDA;LRRK2;SLC35F1;RYR3;RERG;DACH1;SAMD12;NCKAP5;GRID1;DCC;MAGI2;TACR3;MYRIP;MYO3B;FREM2;ACSS3;RTN1;SGMS1;DNAH8;DNAH6;PCDH15;CACNA1C;FHIT;MIPOL1;PAK1;ADAMTS17;PCDHA3;GPC5;SLIT3;PCDHA7;PLXNA4;MDN1;ZNF585A;DTNA;CNTN5;MGMT;GABRA3;ZNF804A;TRAPPC8;LHFPL3;MERTK;HS3ST4;PTPRD;AGBL4;FAM155A;ASXL3;MYO5B;CPE;XKR4;KIAA0825*
H3K9me3 GM12878 hg19	53/2000	1.0	*ALK;LRRK2;MIR663A;SLC35F1;ARHGAP6;ZNF280D;PPP3CA;CDH4;ADAMTSL3;KYNU;DLGAP1;MACROD2;HMCN1;PCDHAC1;COL25A1;UNC5D;PCDHA13;SEZ6L;SORCS3;PCDHA12;ROCK1P1;PCDHA10;RUNX1;DNM3;NRG3;PRKD1;ERG;ZFPM2;DOCK1;SKAP2;CTBP2;GLIS3;ADAMTS17;PRRG1;PCDHA5;ANKRD36BP2;FSD2;PCDHA8;GPC6;PCDHA7;PCDHA6;CLVS1;CA10;PCDH9;GABRA5;RANBP17;ZNF804A;VWA3B;*

			FRG1B;PTK2;STXBP5L;MCM3AP;XKR4
H3K4me3 fibroblast of the aortic adventitia hg19	43/1648	1.0	*AVEN;CNTNAP2;ACSS3;ZNF493;CUL5;MAST2;TSHZ2;NOTCH2NL;MIR663A;NPAS3;AKAP13;ANKRD36BP2;PCDHA3;HMCN1;DIP2C;SAMD12;NCKAP5;PCDHA7;PRKG1;ANKS1B;RUNX1T1;EPHA5;KLF12;HFM1;AUTS2;TRPC4;LRBA;LHFPL3;VWA3B;TEKT4P2;KY;MGAT4C;SDK1;PLCB4;NFIA;NBEA;ZNF717;ASXL3;WDPCP;TTLL11;PLCB1;DOCK1;FBXL7*
H3K4me2 skeletal muscle myoblast hg19	52/2000	1.0	*FHOD3;ATP8A2;SLC35F3;LRRK2;SLC35F1;RERG;ERC1;SAMD12;NCKAP5;ANKS1B;PDE4D;MAGI2;HUNK;TACR3;UNC5D;ANK3;MYRIP;CACNB2;MYO3B;MIR3687;ERG;FREM2;RTN1;SGMS1;SATB2;TSHZ2;DNAH6;PCDH15;CACNA1C;LRRC16A;MIPOL1;NPAS3;PAK1;LARGE;ADAMTS17;PCDHA3;FSD2;GPC5;PCBD2;PCDHA7;CLVS1;CNTN5;ATRNL1;GABRA3;TRAPPC8;LHFPL3;MERTK;PTPRD;ASXL3;CPE;APBA1;KIAA0825*
H3K4me1 limb mm9	52/2000	1.0	*GABRB3;DPP10;SPAG16;DGKB;CPQ;FMN1;AFF3;RERG;KYNU;SOX6;PRKG1;ANKS1B;PCDHAC1;EPHA6;TRPC4;PDE4D;TACR3;ANK2;NAV2;SEZ6L;ANO2;DNM3;CACNB2;MYO3B;CDH13;COL6A5;PDZRN4;OCA2;PON3;KHDRBS2;LAMA2;SEMA3D;PON1;PCDH15;TMEM163;THSD4;PAK1;SH3TC2;ZMAT4;SPOCK3;FSD2;OPCML;WSCD1;CNTN5;MGMT;COL22A1;SUSD1;ARHGAP24;FAM155A;FGF14;TBXAS1;MIR183*
H3K4me1 astrocyte hg19	52/2000	1.0	*PLEKHB2;DGKB;SLC35F1;GPHN;RERG;SGCD;NDST3;PIEZO2;MACROD2;ZNF385D;SAMD12;SH3GL2;DSCAM;TRPC4;DCC;IFNGR2;MAGI2;NRG1;ANK2;SUMF1;TIAM2;DNM3;ANKFN1;IFT43;MYO3B;ZNF717;TTLL11;TLN2;ST6GALNAC3;ATP6V0D2;FREM2;OCA2;PDE1C;LAMA2;DNAH8;PCDH15;FHIT;ARHGAP22;DHX32;ZMAT4;SYNDIG1;DMD;HIVEP3;N4BP2;CLVS1;SPECC1;CNTN5;GABRA5;GABRA3;VWA3B;LRP1B;DLG2*
H3K4me3 bronchial epithelial cell hg19	48/1859	1.0	*AVEN;ZNF493;CTBP2;CLSTN2;MAST2;TSHZ2;NOTCH2NL;CACNA1C;BICD1;NPAS3;AKAP13;ADAMTS17;SLIT3;DIP2C;NCKAP5;EPHB1;N4BP2;PCDHA7;ANKS1B;RUNX1T1;PRMT8;KLF12;AUTS2;GRID1;COL25A1;LRBA;MAGI2;SUSD1;UNC5D;KY;PARD3B;PTPRD;SDK1;PGM5P2;PLCB4;NFIA;STIM1;ZNF717;MIR3687;ASXL3;CPE;PRKD1;CNTN4;ASTN2;XKR4;EIF4G3;DOCK1;FBXL7*
H3K4me3 BE2C hg19	41/1594	1.0	*CNTNAP2;ZNF493;CTBP2;SGMS1;DNAH8;NOTCH2NL;SLC35F1;CACNA1C;BICD1;MRPL33;AKAP13;ADAMTS17;PCDHA2;MACROD2;LRRC4C;PCDHA8;EPHB1;GPM6B;KIRREL3;CA10;HFM1;AUTS2;COL25A1;PLCL1;NRG1;VWA3B;TEKT4P2;KY;PCDHA10;PARD3B;B3GALTL;PTPRD;CDK6;FGF14;ZNF717;CPE;TTLL11;COL6A5;XKR4;EIF4G3;CNTNAP4*
H3K4me3 fibroblast of gingiva hg19	53/2061	1.0	*ZNF493;MAST2;ARHGAP6;HAPLN1;AKAP13;SGCD;HMCN1;SAMD12;NCKAP5;PRKG1;SH3GL2;ANKS1B;HFM1;DSCAM;COL25A1;DCC;UNC5D;MYRIP;TEKT4P2;PLCB4;ZNF717;CDC42EP3;TTLL11;PRKD1;DOCK1;RTN1;CTBP2;DNAH8;TSHZ2;NOTCH2NL;NPAS3;RELN;PCDHA3;LRRC4C;PLXNA4;RUNX1T1;LINC00478;AUTS2;LRBA;DNAH14;ZNF804A;VWA3B;MERTK;KY;AGBL4;SDK1;FAM155A;CDK6;NFIA;ASXL3;CPE;EIF4G3;FBXL7*
H3K4me1 spleen mm9	51/2000	1.0	*DPP10;ALK;PTPRT;FHOD3;AVEN;ITGAM;EDA;CLSTN2;SYCP2;CTNND2;OTUD7A;GRIK4;ADAMTSL3;FAM110B;ERC1;SH3GL2;CSGALNACT1;HFM1;GRID1;COBL;KAZN;ANK2;UNC5D;KSR2;NAV2;MYRIP;SUMF1;PARD3B;DNM3;CACNB2;IFT43;TBC1D5;ATP6V0D2;PRRG1;SLIT3;ASIC2;TRPM3;RUNX1T1;AUTS2;ATRNL1;LRBA;GAREM;LHFPL3;KY;NBEA;BRE;TBXAS1;NF1;CPE;APBA1;FBXL7*
H3K4me1 testis mm9	51/2000	1.0	*SEMA5A;ALK;FHOD3;CNTNAP2;PLEKHB2;DGKB;AFF3;HAPLN1;SGCD;RGS3;DIP2C;SAMD12;ANKS1B;PCDHAC1;GRID1;TRPC4;MAGI2;HUNK;UNC5C;NRG1;UPK3B;PCDHA11;TIAM2;SPATA16;MYO3B;DGKI;PDZRN4;CNTNAP4;OCA2;KHDRBS2;ACSS3;SGMS1;PDE1C;LAMA2;CACNA1D;RELN;HIVEP3;PCBD2;ASIC2;TRPM3;CCDC178;CNTN5;MGMT;SHFM1;PLCL1;GAREM;HS3ST4;FAM135B;NSG2;ASXL3;APBA1*
H3K4me3 fibroblast of upper leg skin hg19	50/1975	1.0	*AVEN;CNTNAP2;PLEKHB2;MAST2;FMN1;BICD1;AKAP13;HMCN1;DIP2C;NCKAP5;PRKG1;ANKS1B;EPHA5;GRID1;COL25A1;MAGI2;MYRIP;PARD3B;PLCB4;ZNF717;MIR3687;CDC42EP3;WDPCP;TTLL11;DOCK1;ACSS3;RTN1;CTBP2;SATB2;NOTCH2NL;NPAS3;RELN;PCDHA3;PCBD2;PLXNA4;LINC00478;PCDH9;AUTS2;LRBA;PHKB;LHFPL3;MERTK;KY;PTPRD;AGBL4;SDK1;CDK6;NFIA;ASXL3;FBXL7*
H3K4me3 GM12864 hg19	32/1279	1.0	*PON3;ZNF493;PLEKHB2;MAST2;NOTCH2NL;NRXN3;LRP2;PTPRG;AKAP13;FSD2;GPC5;AUTS2;COL25A1;IFNGR2;PLCL1;VWA3B;FRG1B;MERTK;TEKT4P2;NFIA;STIM1;NBEA;ZNF717;MIR3687;MYO5B;CDC42EP3;TBXAS1;NF1;CPE;TTLL11;EIF4G3;MAPRE2*
H3K4me3 astrocyte of	40/1594	1.0	*LRP2;ARHGAP6;NPAS3;AKAP13;PIEZO2;DLGAP1;CSMD3;MACROD2;NCKAP5;PRKG1;ANKS1B;DSCAM;AUTS2;MGMT;C*

the cerebellum hg19			*OL25A1;LRBA;PLCL1;HUNK;TACR3;LHFPL3;VWA3B;MYRIP;PARD3B;AGBL4;CACNB2;SDK1;PGM5P2;INTS4L1;CDK6;NFIA;NBEA;ZNF717;NF1;CPE;ERG;CNTN4;XKR4;FREM2;FBXL7;PDZRN4*
H3K27me3 K562 hg19	84/3318	1.0	*ALK;FHOD3;CNTNAP2;RYR2;ATP8A2;GRIK4;FMN1;MIR663A;AFF3;RYR3;HAPLN1;CA3;GRM7;FAM110B;PIEZO2;GPR139;HMCN1;PRKG1;ANKS1B;WLS;ANKUB1;CSGALNACT1;KIRREL3;ZSCAN5A;GRID1;HUNK;COBL;KAZN;UNC5C;ANK2;ELOVL7;KSR2;ANK3;NAV2;MYRIP;SEZ6L;ROCK1P1;MIR3648;TIAM2;CACNB2;IFT43;MIR3687;ELMO1;CDH13;PRKD1;DSCAML1;MAPRE2;FREM2;LINC00284;OCA2;PON3;RTN1;PDE1C;DNAH8;NTM;TSHZ2;PON1;DNAH6;ATP1A4;CACNA1D;LRP2;TMEM163;HDAC9;EHBP1;ZMAT4;HIVEP3;GPC5;SRGAP3;GPC6;TRPM3;CLVS1;AUTS2;ANKRD30BL;COL22A1;TRAPPC8;LHFPL3;VWA3B;FRG1B;KY;ARHGAP24;DAB1;FRAS1;MIR1324;CPE*
H3K4me2 keratinocyte hg19	50/2000	1.0	*SEMA5A;FHOD3;CNTNAP2;ANKRD20A2;RERG;DACH1;DLGAP1;ERC1;PCDHAC1;PRMT8;COL25A1;DCC;UNC5D;UPK3B;PARD3B;ST6GALNAC3;FREM2;PDZRN4;OCA2;ACSS3;RTN1;DNAH6;ARHGAP22;ADAMTS17;PCDHA4;ANKRD36BP2;SLIT3;PCBD2;N4BP2;PLXNA4;ZNF585A;GABBR2;XRCC4;CNTN5;ATRNL1;COL22A1;PLCL1;GABRA3;ZNF804A;LHFPL3;VWA3B;KY;STXBP5L;SDK1;ANKRD20A3;BRE;ASXL3;MYO5B;CPE;FBXL7*
H3K4me1 olfactory bulb mm9	50/2000	1.0	*SPAG16;ATP8A2;EDA;THSD7B;CPQ;RERG;FAM110B;ERC1;HMCN1;SAMD12;ADAMTS6;HFM1;TRPC4;MAGI2;ARAP2;TACR3;KAZN;MYRIP;PARD3B;CACNB2;LANCL3;UTRN;ATP6V0D2;FREM2;WDR27;ACSS3;LAMA2;PCDH15;LRRC16A;RELN;HIVEP3;TRPM3;GPM6B;SPECC1;GABRA5;ATRNL1;SUSD1;FBXL17;HS3ST4;ARHGAP24;PTPRD;AGBL4;MSRA;FAM135B;MYO5B;DLC1;CPE;CNTN4;XKR4;CCDC171*
H3K4me3 MCF-7 hg19	47/1890	1.0	*CTBP2;CUL5;MAST2;TSHZ2;NOTCH2NL;PCDH15;MIR663A;TMTC1;LRP2;GPHN;NPAS3;AKAP13;DACH1;ADAMTS17;SYNDIG1;PCBD2;HMCN1;LRRC4C;NCKAP5;PCDHA8;EPHB1;PCDHA7;PLXNA4;PRKG1;PCDHA6;RUNX1T1;SNRPN;CNTN5;AUTS2;CACNA2D1;LRBA;PCDHA13;PCDHA12;UPK3B;TEKT4P2;PARD3B;PTPRD;SDK1;PGM5P2;PLCB4;FRAS1;NFIA;NBEA;CDC42EP3;TTLL11;PRKD1;EIF4G3*
H3K36me3 kidney epithelial cell hg19	5/215	1.0	*MIR3648;LINC00273;ANKRD30BL;MIR3687;MIR663A*
H3K9me3 U2OS hg19	26/1078	1.0	*ACSS3;MIR663A;ZNF280D;PCDHA1;SH3TC2;ZMAT4;CCDC30;PCDHA4;PCDHA3;PCDHA2;MACROD2;MIR4461;PCDHA7;PCDHAC1;CALN1;LINC00273;ANKRD30BL;DNAH14;RANBP17;PCDHA13;PCDHA12;ROCK1P1;PCDHA11;MIR3648;MIR3687;SHANK2*
H3K27me3 splenic B cell mm9	9/391	1.0	*FHOD3;SLC35F3;CDC42EP3;TBXAS1;CPE;SORCS3;PTK2;RERG;STXBP5L*
H3K4me2 HepG2 hg19	49/2000	1.0	*ROBO2;AVEN;ATP8A2;CSRNP3;PLEKHB2;LRRK2;FMN1;ARHGAP6;RERG;DLGAP1;SAMD12;KIFAP3;NCKAP5;EPHB1;GRID1;MAGI2;MYRIP;PARD3B;CACNB2;NCOR1;ST6GALNAC3;RTN1;CTBP2;LAMA2;DNAH6;ADCY2;CACNA1C;NPAS3;ARHGAP22;SH3TC2;ADAMTS17;SPOCK3;FPGT-TNNI3K;LRRC4C;PCDHA8;N4BP2;PCDHA7;ZNF585A;GABBR2;WSCD1;AUTS2;ATRNL1;PLCL1;ZNF804A;ARHGAP24;PTPRD;DAB1;BRE;ASXL3*
H3K9ac ES-Bruce4 mm9	49/2000	1.0	*ALK;SPAG16;SLC35F3;LRRK2;GPHN;GRM1;TTC28;SGCD;CCDC91;HMCN1;EPHA5;EPHA6;GRID1;CACNA2D1;SEMA6D;FNDC3B;KSR2;FAM126B;SEZ6L;PCDHA11;PCDHA10;TIAM2;CDC42EP3;WDPCP;COL6A5;PON3;PRDM15;LRRC16A;RELN;PCDHA1;ZMAT4;PCDHA4;PCBD2;PCDHA9;PCDHA7;OPCML;GABBR2;CNTN5;ATRNL1;LHFPL3;HS3ST4;KY;PTPRD;MCM3AP;DAB1;MIR183;APBA1;CNTN4;EIF4G3*
H3K4me3 fibroblast of skin of abdomen hg19	50/2074	1.0	*AVEN;ZNF493;PLEKHB2;MAST2;FMN1;AKAP13;DACH1;SGCD;NCKAP5;EPHB1;PRKG1;SH3GL2;ANKS1B;KIRREL3;HFM1;DSCAM;COL25A1;DCC;CACNA2D1;MAGI2;HUNK;TEKT4P2;ZNF717;TTLL11;PRKD1;DOCK1;SATB2;NOTCH2NL;NPAS3;ANKRD36BP2;PCDHA3;SRGAP3;LRRC4C;PLXNA4;RUNX1T1;LINC00478;PCDH9;AUTS2;LRBA;DNAH14;ZNF804A;LHFPL3;KY;PTPRD;PGM5P2;NFIA;NBEA;ASXL3;CPE;EIF4G3*
H3K4me1 small intestine mm9	48/2000	1.0	*DPP10;ALK;FHOD3;PON3;KHDRBS2;ATP8A2;CLSTN2;TENM4;SLC35F3;CPQ;SEMA3D;LRRK2;NPAS3;NKAIN2;RELN;RGS3;SNTG1;ZMAT4;LARGE;KYNU;PRRG1;SYNDIG1;CCDC30;SRGAP3;HMCN1;ANKS1B;CSGALNACT1;EPHA5;PACRG;HFM1;MGMT;COL25A1;CACNA2D1;PLCL1;UNC5C;ANK3;NAV2;STXBP5L;CACNB2;SDK1;NSG2;SCN8A;CDH13;APBA1;*

			XKR4;ST6GALNAC3;DGKI;SHANK2
H3K4me3 SK-N-SH hg19	48/2000	1.0	ZNF493;RTN1;CTBP2;MAST2;TSHZ2;NOTCH2NL;PCDH15;LRP2;ARHGAP6;BICD1;RYR3;ADAMTSL1;AKAP13;RELN;ADAMTS17;SPOCK3;PCBD2;ZNF385D;DIP2C;SAMD12;PCDHA8;PRKG1;ANKS1B;TRPC5;HFM1;AUTS2;GRID1;DCC;LRBA;MAGI2;LHFPL3;VWA3B;FRG1B;TEKT4P2;KY;SDK1;PGM5P2;PLCB4;FRAS1;NFIA;ZNF717;ASXL3;CPE;TTLL11;PRKD1;EIF4G3;MAPRE2;DOCK1
H3K4me2 astrocyte hg19	48/2000	1.0	FHOD3;KHDRBS2;SGMS1;PDE1C;DNAH8;LRRK2;DNAH6;PCDH15;CACNA1D;HDAC9;RYR3;RERG;MIPOL1;RPS6KA5;PIEZO2;PCDHA3;DMD;HIVEP3;PCBD2;SAMD12;CSMD1;TBC1D22A;N4BP2;TRPM3;PCDHAC1;ZNF585A;SNRPN;WSCD1;ATRNL1;MGMT;GABRA3;MAGI2;HUNK;TACR3;NRG1;VWA3B;HS3ST4;PARD3B;KIAA1217;TIAM2;AGBL4;INTS4L1;ANKFN1;MYO3B;ZNF717;ATP6V0D2;KIAA0825;FREM2
H3K4me1 brain mm9	48/2000	1.0	GABRB3;SEMA5A;PTPRT;AVEN;OCA2;KHDRBS2;ACSS3;RTN1;ANKRD36;CPQ;PCDH15;CACNA1C;BICD1;RERG;ADAMTSL1;NKAIN2;SGCD;PRRG1;SLIT3;GPR139;CSMD3;DIP2C;SOX6;WLS;CALN1;CSGALNACT1;TRPC5;EPHA6;DTNA;HFM1;DSCAM;MGMT;TRPC4;COL22A1;RANBP17;KY;DNM3;CACNB2;MSRA;DLG2;NFIA;NBEA;MYO3B;MYO5B;DPYD;ELMO1;MIR183;CNTN4
H3K4me3 fibroblast of pedal digit skin hg19	49/2043	1.0	ROBO2;MAST2;MIR663A;BICD1;RYR3;HMCN1;NCKAP5;PRKG1;SH3GL2;ANKS1B;HFM1;DSCAM;CACNA2D1;PCDHA13;MYRIP;TEKT4P2;PCDHA10;PLCB4;ZNF717;TTLL11;PRKD1;ST6GALNAC3;RTN1;CTBP2;DNAH8;TSHZ2;NOTCH2NL;NPAS3;PAK1;SYNDIG1;ANKRD36BP2;PCDHA3;PLXNA4;RUNX1T1;LINC00478;PCDH9;CNTN5;AUTS2;DNAH14;LHFPL3;VWA3B;FRG1B;PTPRD;AGBL4;FAM155A;NFIA;NBEA;ASXL3;CPE
H3K4me3 epithelial cell of esophagus hg19	50/2093	1.0	ZNF493;PLEKHB2;CTNND2;MAST2;ARHGAP6;BICD1;GPHN;AKAP13;DIP2C;EPHB1;PRKG1;SH3GL2;PACRG;KLF12;SNRPN;HFM1;ZNF283;DSCAM;COL25A1;CACNA2D1;MAGI2;TEKT4P2;PLCB4;ZNF717;TTLL11;PRKD1;ST6GALNAC3;DOCK1;FREM2;ACSS3;CTBP2;DNAH8;NOTCH2NL;CACNA1C;NPAS3;PCDHA4;LRRC4C;RUNX1T1;CNTN5;AUTS2;LRBA;DNAH14;TRAPPC8;PGM5P2;FRAS1;NFIA;NBEA;ASXL3;CPE;EIF4G3
H3K4me3 foreskin fibroblast hg19	80/3309	1.0	FHOD3;ZNF493;PLEKHB2;MAST2;FMN1;GPHN;PTPRG;AKAP13;DACH1;RPS6KA5;PIEZO2;SAMD12;PRKG1;SH3GL2;ANKS1B;PACRG;HFM1;DSCAM;GRID1;TRPC4;COL25A1;DCC;IFNGR2;MAGI2;HUNK;TACR3;NRG1;TEKT4P2;SUMF1;CACNB2;TMEM135;PLCB4;ZNF717;HECW1;WDPCP;TTLL11;PRKD1;ST6GALNAC3;ZNF675;DOCK1;OCA2;ACSS3;RTN1;CTBP2;DNAH8;NOTCH2NL;CACNA1D;LRP2;NPAS3;NKAIN2;PCDHA3;SRGAP3;PCBD2;LRRC4C;PCDHA8;GPC6;PCDHA7;PLXNA4;MDN1;RUNX1T1;GABBR2;AUTS2;LRBA;ZNF804A;PHKB;VWA3B;FRG1B;KY;PTPRD;AGBL4;SDK1;PGM5P2;MCM3AP;FRAS1;NFIA;NBEA;ASXL3;CPE;EIF4G3;KIAA0825
H3K27me3 C2C12 mm9	29/1269	1.0	RYR2;RTN1;THSD7B;SLC35F3;OTUD7A;GRIK4;TMTC1;TMEM163;NPAS3;SNTG1;HIVEP3;CSMD3;EPHB1;PLXNA4;SH3GL2;WSCD1;GRID1;SUSD1;LHFPL3;KSR2;NAV2;SYN3;KY;STXBP5L;FAM135B;NSG2;SCN8A;DGKI;SHANK2
H3K4me1 brown adipose tissue mm9	47/2000	1.0	GABRB3;ALK;ATP8A2;CSRNP3;RTN1;PDE1C;SLC35F3;SEMA3D;NTM;PON1;FMN1;LRP2;MIR96;TMEM163;ROBO1;ADAMTSL1;CCDC91;GPC5;PRKG1;CORIN;SH3GL2;ANKS1B;KIRREL3;HFM1;PCDH9;GRID1;COL25A1;COL22A1;MAGI2;SUSD1;FBXL17;TACR3;UNC5D;KSR2;MYRIP;SEZ6L;ARHGAP24;STXBP5L;SLC2A9;CACNB2;PLCB4;NBEA;NSG2;LANCL3;MIR183;CDH13;DGKI
H3K4me1 placenta mm9	47/2000	1.0	DPP10;SPAG16;FHOD3;PON3;KHDRBS2;ATP8A2;RTN1;SLC35F3;DGKB;CPQ;LRRK2;PON1;GRIK4;NPAS3;ROBO1;SGCD;PCDHA1;LARGE;SYNDIG1;SPOCK1;HMCN1;SAMD12;NCKAP5;SH3GL2;WLS;CLVS1;HFM1;DSCAM;MGMT;PLCL1;MAGI2;DIO2;UNC5D;NAV2;STXBP5L;AGBL4;FAM135B;PLCB4;DAB1;NSG2;CPE;LANCL3;CDH13;COL6A5;XKR4;PLCB1;DGKI
H4K20me1 H1-hESC hg19	47/2000	1.0	CNTNAP2;RYR2;ATP8A2;CLSTN2;CHRM5;GLIS3;ADCY2;MIR663A;LRP2;THSD4;EFTUD1;RELN;GRM7;ZMAT4;ADAMTS17;PIEZO2;HIVEP3;CSMD3;MIR4461;MDN1;EPHA5;KIRREL3;CHST9;PRMT8;ANKRD30BL;GRID1;PLCL1;UNC5C;TRAPPC8;LHFPL3;VWA3B;MYRIP;SEZ6L;ROCK1P1;FOXP2;ARHGAP24;KIAA1217;MIR3648;NCOR1;MIR3687;APBA1;PRKD1;CNTN4;XKR4;ZFPM2;DSCAML1;MAPRE2
H3K4me2 fibroblast of dermis hg19	47/2000	1.0	CNTNAP2;ATP8A2;CSRNP3;RTN1;SGMS1;DNAH8;SATB2;DNAH6;ADK;PCDH15;SLC35F1;CACNA1D;FHIT;HDAC9;RERG;MIPOL1;ADAMTS17;PCDHA3;GPC5;CTNNA3;SAMD12;SH3GL2;SPECC1;WSCD1;MGMT;COL25A1;PLCL1;ZNF804A;TACR3;NRG1;UNC5D;VWA3B;MYRIP;ROCK1P1;PARD3B;RUN

			X1;PTPRD;AGBL4;CACNB2;ZNF717;ASXL3;CPE;PLCB1;ST6GALNAC3;ZFPM2;FREM2;DGKI
H3K9ac NT2-D1 hg19	47/2000	1.0	*GABRB3;SEMA5A;FHOD3;PDE1C;DNAH8;ADK;ADCY2;LRRC16A;MIR96;FHIT;MRPL33;MIPOL1;EFTUD1;ADAMTSL3;PCDHA4;PCBD2;HMCN1;SAMD12;TBC1D22A;MDN1;ADAMTS6;TRPM3;PCDHAC1;XRCC4;DSCAM;WSCD1;CNTN5;GABRA5;DCC;LRBA;ZNF804A;TACR3;COBL;ELP4;PCDHA13;FAM126B;KY;AGBL4;NCOR1;CDK6;ZNF717;WDPCP;TTLL11;PLCB1;KIAA0825;DOCK1;PDZRN4*
H3K4me2 A549 hg19	65/2720	1.0	*EDA;CTNND2;SLC35F1;RYR3;RERG;HMCN1;SAMD12;TBC1D22A;ADAMTS6;PCDHAC1;CHST9;ZNF283;DSCAM;TRPC4;ARAP2;HUNK;TACR3;UNC5D;SYN3;SORCS3;PCDHA12;UPK3B;PARD3B;KIAA1217;MIR3648;CACNB2;NCOR1;ZNF717;TTLL11;ST6GALNAC3;FREM2;ACSS3;SGMS1;LAMA2;DNAH6;CACNA1C;ARHGAP22;PCDHA1;PCDHA5;PCDHA4;ANKRD36BP2;PCDHA3;HIVEP3;GPC5;CTNNA3;PCDHA2;PCDHA8;PLXNA4;ZNF585A;AUTS2;ATRNL1;PLCL1;GABRA3;SUSD1;VWA3B;ELP4;MERTK;PTK2;AGBL4;SDK1;DIAPH3;ASXL3;CPE;CNTN4;XKR4*
H3K4me3 H7 hg19	33/1452	1.0	*AVEN;CNTNAP2;MAST2;NOTCH2NL;NPAS3;AKAP13;DACH1;HMCN1;ANKS1B;EPHA5;WSCD1;CNTN5;AUTS2;LRBA;HUNK;TACR3;PHKB;LHFPL3;PCDHA13;SDK1;FRAS1;NFIA;STIM1;MIR3687;ASXL3;NF1;ELMO1;CPE;TTLL11;ERG;XKR4;MAPRE2;PDZRN4*
H3K4me3 A549 hg19	93/3895	1.0	*RYR2;ZNF493;EDA;CLSTN2;LRRK2;MAST2;SLC35F1;BICD1;MRPL33;PTPRG;ROBO1;TTC28;MACROD2;SAMD12;NCKAP5;ADAMTS6;ANKS1B;PCDHAC1;ANKUB1;TRPC4;ARAP2;HUNK;KAZN;NRG1;UNC5D;ELOVL7;SYN3;PCDHA12;UPK3B;TEKT4P2;FOXP2;PARD3B;CACNB2;ZNF717;MIR3687;SCN8A;CDC42EP3;ELMO1;LANCL3;WDPCP;TTLL11;TLN2;ASTN2;ST6GALNAC3;ZFPM2;FREM2;KHDRBS2;ACSS3;SGMS1;LAMA2;TSHZ2;DNAH6;NOTCH2NL;CACNA1D;CACNA1C;ARHGAP22;PCDHA1;ADAMTS17;PCDHA5;SYNDIG1;ANKRD36BP2;PCDHA3;HIVEP3;GPC5;PCDHA2;PCDHA8;PCDHA7;ZNF585A;GABBR2;MGAM;DTNA;WSCD1;AUTS2;ATRNL1;MGMT;PLCL1;DNAH14;ZNF804A;PHKB;TRAPPC8;MERTK;KY;PTPRD;AGBL4;SDK1;FAM155A;CDK6;DIAPH3;ASXL3;MYO5B;CPE;CNTN4;KIAA0825*
H3K4me3 cardiac fibroblast hg19	68/2902	1.0	*AVEN;PLEKHB2;CTNND2;MAST2;ARHGAP6;BICD1;SLC8A1;PTPRG;AKAP13;DACH1;DLGAP1;MACROD2;DIP2C;NCKAP5;PRKG1;SH3GL2;ANKS1B;EPHA5;HFM1;GRID1;COL25A1;DCC;CACNA2D1;KAZN;MYRIP;TEKT4P2;PCDHA10;PARD3B;PLCB4;ZNF717;TTLL11;PRKD1;ASTN2;DOCK1;RTN1;CTBP2;CUL5;DNAH8;TSHZ2;NOTCH2NL;LRP2;NPAS3;RELN;ADAMTS17;ANKRD36BP2;SRGAP3;PCDHA7;PLXNA4;PCDHA6;RUNX1T1;DTNA;CNTN5;AUTS2;LRBA;DNAH14;PHKB;LHFPL3;PTPRD;AGBL4;SDK1;PGM5P2;CDK6;FRAS1;NFIA;ASXL3;CPE;EIF4G3;FBXL7*
H3K4me3 olfactory bulb mm9	45/2000	1.0	*SPAG16;PON3;KHDRBS2;ATP8A2;CLSTN2;DGKB;LRRK2;TSHZ2;PCDH15;PTPRM;NPAS3;ADAMTSL1;ENOX2;PPP3CA;SGCD;RPS6KA5;SPTLC2;SH3TC2;ADAMTSL3;PCDHA5;SPOCK1;ADAMTS6;TRPM3;PCDHAC1;GABBR2;DSCAM;CNTN5;PLCL1;MAGI2;KSR2;MYRIP;MERTK;PCDHA12;PCDHA11;KY;ANO2;AGBL4;CACNB2;FAM135B;MCM3AP;NSG2;MYO5B;DPYD;FREM2;SKAP2*
H3K4me3 embryonic fibroblast mm9	45/2000	1.0	*SPAG16;PON3;KHDRBS2;ACSS3;EDA;TENM4;SLC35F3;TSHZ2;PCDH15;LRRC16A;FHIT;MRPL33;NPAS3;ENOX2;SGCD;RPS6KA5;CCDC91;ZMAT4;LARGE;ADAMTS17;CCDC30;SPOCK1;CSMD3;SRGAP3;NCKAP5;EPHB1;CSGALNACT1;GABBR2;HFM1;DSCAM;GRID1;SEMA6D;MAGI2;KSR2;MERTK;AGBL4;CACNB2;FAM155A;DAB1;MYO5B;CDH13;PLCB1;MAPRE2;FREM2;DGKI*
H3K27me3 HeLa-S3 hg19	45/2000	1.0	*ALK;PTPRT;LRRK2;SATB2;NRXN3;PTPRM;ADCY2;MIR663A;DACH1;ZMAT4;LARGE;FAM110B;DMD;FSD2;CTNNA3;GPR139;EPHB1;PLXNA4;PRKG1;SLC22A10;EPHA5;KIRREL3;SPECC1;PRMT8;DTNA;DSCAM;LHFPL3;MYRIP;SYN3;ROCK1P1;KY;MIR3648;SPATA16;MIR3687;MYO5B;DLC1;SCN8A;TBXAS1;TTLL11;APBA1;TCF4;ST6GALNAC3;DSCAML1;FREM2;CNTNAP4*
H3K4me3 skeletal muscle myoblast hg19	45/2000	1.0	*SGMS1;SLC35F3;ANKRD36;DNAH8;CTNND2;TSHZ2;DNAH6;FMN1;SLC35F1;CACNA1C;BACH1;FHIT;NPAS3;DLGAP1;DMD;SLIT3;FPGT-TNNI3K;PCBD2;SAMD12;TBC1D22A;N4BP2;PCDHA7;CHST9;GABBR2;ZNF283;ATRNL1;ANKRD30BL;MGMT;CACNA2D1;LRBA;HUNK;ZNF804A;TACR3;UNC5D;VWA3B;ELP4;AGBL4;CACNB2;SDK1;PGM5P2;STIM1;MYO5B;WDPCP;ST6GALNAC3;KIAA0825*
H3K4me3 ES-Bruce4 mm9	45/2000	1.0	*ALK;SPAG16;PON3;ACSS3;ATP8A2;PLEKHB2;PDE1C;SLC35F3;LAMA2;TSHZ2;PRDM15;FHIT;MRPL33;SGCD;RPS6K*

			A5;CCDC91;SNTG1;PCDHA5;PCDHA3;PRKG1;TRPM3;PCDHA6;RUNX1T1;PCDHAC1;CLVS1;EPHA6;HFM1;WSCD1;CNTN5;MAGI2;FBXL17;UNC5C;KSR2;PCDHA12;PCDHA11;FOXP2;KY;PCDHA10;AGBL4;CACNB2;MYO3B;MYO5B;DPYD;ELMO1;CNTN4
H3K9ac H1-hESC hg19	45/2000	1.0	KHDRBS2;MAST2;DNAH6;PRDM15;ADK;PTPRM;LRP2;LRRC16A;ARHGAP6;RYR3;MRPL33;MIPOL1;AKAP13;SPOCK3;SYNDIG1;GPR139;ERC1;HMCN1;TBC1D22A;N4BP2;PCDHAC1;EPHA5;PACRG;XRCC4;DSCAM;CNTN5;TRPC4;LRBA;COL22A1;KAZN;TRAPPC8;VWA3B;ELP4;KSR2;PCDHA13;PARD3B;TMEM135;DIAPH3;NFIA;MYO5B;WDPCP;TTLL11;CNTN4;COL6A5;SKAP2
H3K4me3 brain mm9	45/2000	1.0	SPAG16;PON3;KHDRBS2;ACSS3;ATP8A2;PLEKHB2;PCDH15;FHIT;GRM1;NPAS3;SGCD;RPS6KA5;SPTLC2;SNTG1;ZMAT4;ADAMTS17;PCDHA5;PCDHA4;PCDHA3;SRGAP3;HMCN1;PCDHA9;TRPM3;PCDHAC1;SPECC1;EPHA6;HFM1;CNTN5;COL25A1;SEMA6D;MAGI2;FBXL17;KSR2;PCDHA12;PCDHA11;KY;ANO2;STXBP5L;CDK6;MYO5B;DPYD;ST6GALNAC3;DOCK1;FREM2;SKAP2
H3K27me3 myocyte mm9	25/1183	1.0	RYR2;RTN1;THSD7B;SLC35F3;GRIK4;TMTC1;TMEM163;NPAS3;HIVEP3;GPC5;ASIC2;EPHB1;PLXNA4;SH3GL2;WSCD1;GRID1;SUSD1;KSR2;MYRIP;KY;STXBP5L;SCN8A;DGKI;PDZRN4;SHANK2
H3K4me3 megakaryocyte mm9	55/2441	1.0	DPP10;SPAG16;INVS;AVEN;CLSTN2;DGKB;OTUD7A;FMN1;GPHN;ADAMTSL1;CDH4;SGCD;SAMD12;KIFAP3;ANKS1B;HFM1;DSCAM;CACNA2D1;FNDC3B;ARAP2;HUNK;COBL;UNC5C;UNC5D;KSR2;NAV2;FOXP2;SUMF1;VWA8;CACNB2;SCN8A;DPYD;DOCK1;KHDRBS2;SGMS1;WRB;TMTC1;EHBP1;RELN;ZMAT4;CCDC30;SPOCK1;GPC5;OPCML;SPECC1;PCDH9;ATRNL1;LRBA;PLCL1;PTPRD;AGBL4;SDK1;NF1;APBA1;XKR4
H3K4me1 ES-E14 mm9	54/2411	1.0	GABRB3;CNTNAP2;PLEKHB2;THSD7B;SLC35F3;LRRK2;MIR96;RERG;HAPLN1;ADAMTSL1;SGCD;KYNU;NCKAP5;PCDHAC1;PACRG;TRPC4;COL25A1;DCC;IFNGR2;ARAP2;COBL;KAZN;ANK2;ANK3;SEZ6L;SORCS3;FOXP2;SUMF1;TIAM2;VWA8;CACNB2;NRG3;MYO3B;DGKI;SHANK2;CNTNAP4;WDR27;PON3;PDE1C;PAK1;SH3TC2;SYNDIG1;PCDHA7;CLVS1;CCDC178;WSCD1;CNTN5;COL22A1;LHFPL3;ARHGAP24;MSRA;FRAS1;NSG2;MIR183
H3K4me3 NT2-D1 hg19	44/2000	1.0	ATP8A2;SGMS1;PDE1C;SLC35F3;CTNND2;LRRK2;SATB2;ADK;LRRC16A;HDAC9;MIPOL1;EFTUD1;ADAMTSL1;DACH1;ADAMTSL3;CSMD3;ERC1;PCBD2;SAMD12;TBC1D22A;PCDHA7;PCDHAC1;DSCAM;CNTN5;ATRNL1;COL25A1;LRBA;COL22A1;ARAP2;ZNF804A;TACR3;ELP4;PCDHA13;MYRIP;FAM126B;SEZ6L;AGBL4;NFIA;BRE;ZNF717;DPYD;PLCB1;ZFPM2;SKAP2
H3K4me3 testis mm9	44/2000	1.0	SPAG16;OCA2;PON3;ATP8A2;CLSTN2;PDE1C;SLC35F3;PRDM11;SEL1L2;FHIT;EHBP1;NPAS3;SGCD;RPS6KA5;SPTLC2;SNTG1;ZMAT4;HMCN1;SAMD12;SOX6;NCKAP5;ADAMTS6;PCDHA6;RUNX1T1;PCDHAC1;DSCAM;MGMT;GRID1;COL25A1;SEMA6D;PLCL1;MAGI2;GAREM;KSR2;MERTK;PCDHA12;KY;B3GALTL;FAM135B;MYO5B;DPYD;CNTN4;ST6GALNAC3;DOCK1
H3K4me3 fibroblast of pulmonary artery hg19	46/2093	1.0	ZNF493;RTN1;CTBP2;MAST2;NOTCH2NL;CACNA1D;ANKRD20A1;LRRC16A;NPAS3;ADAMTSL1;AKAP13;DACH1;ADAMTS17;ANKRD36BP2;PCDHA3;SRGAP3;PCBD2;DIP2C;SAMD12;NCKAP5;EPHB1;PCDHA7;PLXNA4;PRKG1;SH3GL2;RUNX1T1;EPHA5;HFM1;AUTS2;COL25A1;DCC;MYRIP;TEKT4P2;KY;PTPRD;SDK1;PLCB4;NFIA;ZNF717;ASXL3;CPE;TTLL11;PRKD1;EIF4G3;DOCK1;FREM2
H3K9me3 ES-E14 mm9	2/155	1.0	B3GALTL;PCDHA8
H3K4me3 fibroblast of villous mesenchyme hg19	43/1982	1.0	AVEN;ZNF493;RTN1;DNAH8;MAST2;NOTCH2NL;FHIT;BICD1;NPAS3;ADAMTSL1;AKAP13;SPOCK3;PCDHA3;PCDHA2;SRGAP3;DIP2C;SAMD12;LRRC4C;PCDHA7;PRKG1;ANKS1B;RUNX1T1;PCDH9;AUTS2;PLCL1;TEKT4P2;PARD3B;PTPRD;AGBL4;SDK1;PGM5P2;PLCB4;NFIA;ZNF717;ASXL3;CPE;TTLL11;PRKD1;PLCB1;ST6GALNAC3;EIF4G3;DOCK1;FREM2
H3K4me3 choroid plexus epithelial cell hg19	45/2069	1.0	CTBP2;PLEKHB2;DNAH8;MAST2;TSHZ2;NOTCH2NL;NRXN3;FMN1;BICD1;NPAS3;AKAP13;MACROD2;HMCN1;NCKAP5;PRKG1;GPM6B;RUNX1T1;CNTN5;AUTS2;DCC;LRBA;MAGI2;PHKB;KY;PARD3B;RUNX1;PTPRD;AGBL4;SDK1;FAM155A;PLCB4;CDK6;NFIA;ZNF717;ASXL3;MYO5B;CDC42EP3;CPE;TTLL11;PRKD1;ASTN2;PLCB1;DOCK1;FREM2;FBXL7
H3K4me3 keratinocyte hg19	76/3344	1.0	SEMA5A;CNTNAP2;ZNF493;MAST2;BICD1;RYR3;RERG;ADAMTSL1;ZNF280D;PPP3CA;AKAP13;DACH1;RPS6KA5;DLGAP1;ERC1;TBC1D22A;EPHB1;SH3GL2;ANKS1B;PCDHAC1;PRMT8;KLF12;DSCAM;COL25A1;KAZN;NRG1;TEKT4P2;PA

			RD3B;PLCB4;ZNF717;MIR3687;LANCL3;WDPCP;ST6GALNAC3;DOCK1;FREM2;RTN1;CTBP2;SGMS1;DNAH8;DNAH6;NOTCH2NL;CACNA1C;ARHGAP22;ADAMTS17;SLIT3;FPGT-TNNI3K;PCBD2;LRRC4C;N4BP2;ZNF585A;AUTS2;ATRNL1;MGMT;LRBA;COL22A1;PLCL1;ZNF804A;TRAPPC8;VWA3B;ELP4;FRG1B;MERTK;KY;PTPRD;AGBL4;SDK1;PGM5P2;FAM155A;FRAS1;NBEA;BRE;ASXL3;CPE;XKR4;KIAA0825
H3K9ac astrocyte hg19	43/2000	1.0	*FHOD3;RTN1;SGMS1;LAMA2;DNAH8;DNAH6;LRRC16A;FHIT;HDAC9;MIPOL1;RPS6KA5;CTNNA3;SLIT3;PCBD2;SAMD12;TBC1D22A;N4BP2;CNTN5;AUTS2;ANKRD30BL;COL25A1;LRBA;COL22A1;SEMA6D;GABRA3;MAGI2;TRAPPC8;NRG1;VWA3B;PARD3B;PTPRD;TIAM2;AGBL4;DIAPH3;STIM1;ZNF717;ASXL3;WDPCP;ERG;ST6GALNAC3;KIAA0825;DGKI;SKAP2*
H3K4me3 SK-N-MC hg19	29/1414	1.0	*WDR27;CNTNAP2;RTN1;CTNND2;MAST2;NOTCH2NL;HDAC9;MRPL33;GRM7;PCDHA5;LINC00478;AUTS2;SEMA6D;PLCL1;DNAH14;FBXL17;ZNF804A;NRG1;TEKT4P2;PARD3B;DNM3;AGBL4;FRAS1;TBXAS1;NF1;CPE;XKR4;ST6GALNAC3;EIF4G3*
H3K4me3 BJ hg19	40/1883	1.0	*CNTNAP2;ZNF493;ATP8A2;RTN1;CTBP2;DNAH8;MAST2;NOTCH2NL;LRRC16A;ARHGAP6;BICD1;NPAS3;AKAP13;ADAMTS17;PCBD2;HMCN1;DIP2C;N4BP2;PCDHA7;PRKG1;RUNX1T1;WSCD1;TRPC4;DCC;LRBA;PLCL1;LHFPL3;VWA3B;MYRIP;FAM155A;PLCB4;FRAS1;NFIA;ZNF717;MIR3687;TBXAS1;CPE;TTLL11;EIF4G3;DOCK1*
H3K4me3 Caco-2 hg19	43/2029	1.0	*FHOD3;AVEN;CNTNAP2;PON3;RTN1;SYCP2;CTNND2;MAST2;TSHZ2;NOTCH2NL;CACNA1C;BICD1;AKAP13;ADAMTS17;SPOCK3;PCBD2;DIP2C;NCKAP5;ANKS1B;RUNX1T1;KLF12;PCDH9;AUTS2;CACNA2D1;LRBA;HUNK;VWA3B;MYRIP;PTPRD;SDK1;PGM5P2;PLCB4;NFIA;BRE;ZNF717;ASXL3;CPE;TTLL11;PRKD1;ASTN2;PLCB1;EIF4G3;MAPRE2*
H3K4me3 GM12866 hg19	42/2000	1.0	*FHOD3;AVEN;ATP8A2;RTN1;CLSTN2;PLEKHB2;SLC35F3;DNAH8;MAST2;TSHZ2;PON1;NOTCH2NL;NRXN3;MIR663A;SLC35F1;ARHGAP6;BICD1;GPHN;ARHGAP22;AKAP13;PAK1;DACH1;ATRNL1;COL25A1;LRBA;PLCL1;SUSD1;KAZN;MYRIP;MERTK;TEKT4P2;FOXP2;RUNX1;SDK1;PGM5P2;MCM3AP;CDK6;NFIA;ZNF717;SCN8A;TBXAS1;MAPRE2*
H3K4me1 endothelial cell of umbilical vein hg19	42/2000	1.0	*ATP8A2;RTN1;CUL5;PLEKHB2;LAMA2;SATB2;PCDH15;SLC35F1;CACNA1C;SAMSN1;RERG;NDST3;ADAMTS17;SRGAP3;LRRC4C;SH3GL2;SPECC1;PACRG;HFM1;PCDH9;AUTS2;MGMT;TRPC4;COL25A1;MAGI2;ARAP2;HUNK;ZNF804A;TACR3;ANK2;VWA3B;PTK2;ARHGAP24;SUMF1;PTPRD;BRE;ELMO1;CPE;TTLL11;DGKI;FBXL7;PDZRN4*
H3K4me3 cerebellum mm9	42/2000	1.0	*ATP8A2;RTN1;EDA;DGKB;LRP2;MRPL33;EFTUD1;ADAMTSL1;ENOX2;RPS6KA5;ADAMTS17;PCDHA5;PCDHA4;PCDHA3;PCDHA9;EPHB1;GPC6;PRKG1;TRPM3;PCDHA6;RUNX1T1;PCDHAC1;CALN1;HFM1;MAGI2;FBXL17;NRG1;PCDHA12;FOXP2;KY;ANO2;PCDHA10;SUMF1;B3GALTL;TIAM2;AGBL4;FAM155A;IFT43;BRE;MYO5B;DPYD;ELMO1*
H3K4me3 cortical plate mm9	42/2000	1.0	*PON3;RTN1;PDE1C;WRB;PCDH15;FHIT;SLC8A1;GRM1;MRPL33;NPAS3;EFTUD1;ADAMTSL1;ENOX2;SGCD;RPS6KA5;SPTLC2;ZMAT4;ADAMTS17;PCDHA5;PCDHA3;SOX6;EPHB1;PRKG1;TRPM3;PCDHAC1;SPECC1;TRPC5;HFM1;WSCD1;SEMA6D;MAGI2;PCDHA12;FOXP2;KY;CDK6;MYO5B;DPYD;ELMO1;ST6GALNAC3;FREM2;SHANK2;SKAP2*
H3K4me3 brown adipose tissue mm9	42/2000	1.0	*PON3;CLSTN2;CTNND2;TSHZ2;PRDM15;FHIT;MRPL33;EFTUD1;SGCD;RPS6KA5;SPTLC2;ADAMTS17;CCDC30;SRGAP3;SAMD12;NCKAP5;GPC6;RUNX1T1;PCDHAC1;SPECC1;GABBR2;HFM1;MGMT;SEMA6D;PLCL1;MAGI2;GAREM;KSR2;MYRIP;SEZ6L;MERTK;B3GALTL;AGBL4;CACNB2;DLG2;NBEA;BRE;MYO5B;XKR4;ST6GALNAC3;DOCK1;FREM2*
H3K9me3 astrocyte hg19	42/2000	1.0	*DPP10;PTPRT;RYR2;PON3;ATP8A2;CLSTN2;DNAH8;SATB2;FMN1;MIR663A;FRG2C;NPAS3;PTPRG;ADAMTSL1;RPS6KA5;ADAMTSL3;ZMAT4;LARGE;PCDHA5;PIEZO2;PCDHA2;MACROD2;PCDHA9;PCDHA8;PCDHA7;PKNOX2;KIRREL3;PRMT8;COL25A1;RANBP17;TACR3;KAZN;LHFPL3;VWA3B;MYRIP;FRG1B;PCDHA12;ROCK1P1;SLC2A9;DAB1;ZNF717;PDZRN4*
H3K4me1 GM12878 hg19	42/2000	1.0	*LINC00284;OCA2;KHDRBS2;ITGAM;RTN1;PON1;FMN1;PRDM11;FHIT;RERG;TTC28;ARHGAP22;KYNU;ADAMTS17;SPOCK3;FSD2;CTNNA3;SRGAP3;EPHB1;GPM6B;CLVS1;CSGALNACT1;HFM1;PCDH9;COL25A1;EXOC6B;ANK2;ROCK1P1;SUMF1;STXBP5L;DNM3;FRAS1;IFT43;NFIA;BRE;MIR1324;HECW1;ELMO1;WDPCP;TTLL11;ERG;TLN2*
H3K4me2 fibroblast of lung hg19	42/2000	1.0	*ATP8A2;RTN1;EDA;SGMS1;DNAH8;SATB2;PCDH15;SLC35F1;CACNA1D;HDAC9;RERG;ARHGAP22;RPS6KA5;RELN;CA3;ZMAT4;PCDHA4;PCDHA3;DMD;GPC5;ERC1;PCBD2;SAMD12;GPM6B;LINC00478;SPECC1;GABBR2;PCDH9;AUTS2;COL25A1;NRG1;VWA3B;MYRIP;MERTK;RUNX1;DIAPH3;MYO*

			3B;ASXL3;ERG;ST6GALNAC3;KIAA0825;FREM2
H3K4me3 HL-60 hg19	39/1881	1.0	FHOD3;CNTNAP2;PON3;RTN1;PLEKHB2;MAST2;NOTCH2NL;CACNA1C;BICD1;SLC8A1;PTPRG;ARHGAP22;AKAP13;ADAMTS17;SRGAP3;PCBD2;EPHB1;ADAMTS6;SH3GL2;ANKS1B;CACNA2D1;LRBA;PLCL1;HUNK;MYRIP;FRG1B;MERTK;TEKT4P2;FRAS1;STIM1;ZNF717;MIR3687;MYO5B;TBXAS1;TTLL11;PRKD1;CNTN4;EIF4G3;MAPRE2
H3K4me1 A549 hg19	64/2951	1.0	RYR2;EDA;MIR663A;ARHGAP6;SLC8A1;GPHN;RERG;DACH1;NDST3;HMCN1;NCKAP5;EYS;SLC22A10;SH3GL2;PACRG;EPHA6;ZNF283;TRPC4;COL25A1;IFNGR2;TACR3;ANK2;KSR2;MYRIP;UPK3B;FOXP2;SUMF1;PARD3B;RUNX1;MIR3648;TIAM2;TMEM135;MYO3B;MIR3687;TTLL11;ERG;TLN2;LINC00486;ATP6V0D2;FREM2;LINC00284;OCA2;CUL5;DNAH6;EHBP1;SH3TC2;SPOCK3;PCDHA4;HIVEP3;GPC5;CSMD3;GPM6B;CORIN;ZNF585A;MGAM;ANKRD30BL;COL22A1;EXOC6B;SGCZ;LRP1B;STXBP5L;PTPRD;FAM155A;DLG2
H3K4me2 endothelial cell of umbilical vein hg19	41/2000	1.0	CSRNP3;RTN1;LAMA2;LRRK2;DNAH6;ADK;PCDH15;FMN1;SLC35F1;RERG;PIEZO2;PCDHA4;PCDHA3;GPC5;SLIT3;SRGAP3;PCBD2;N4BP2;PCDHA7;SH3GL2;ZNF585A;MGMT;TRPC4;COL25A1;MAGI2;ARAP2;ZNF804A;TACR3;VWA3B;ELP4;AGBL4;CACNB2;BRE;MYO5B;WDPCP;ASTN2;LINC00486;ZNF675;KIAA0825;FREM2;PDZRN4
H3K4me2 H1-hESC hg19	41/2000	1.0	SEMA5A;PDE1C;LAMA2;DNAH8;PON1;DNAH6;NRXN3;HDAC9;RERG;MIPOL1;RPS6KA5;ADAMTSL3;FPGT-TNNI3K;ERC1;ZNF585A;PCDH9;WSCD1;CNTN5;COL25A1;COL22A1;SUSD1;ARAP2;ZNF804A;TACR3;VWA3B;MYRIP;PCDHA12;UPK3B;PCDHA10;AGBL4;INTS4L1;MCM3AP;PCP4;DIAPH3;BRE;ZNF717;ASXL3;DPYD;XKR4;DGKI;CNTNAP4
H3K4me3 small intestine mm9	41/2000	1.0	PON3;KHDRBS2;ACSS3;ATP8A2;CLSTN2;TENM4;SLC35F3;TSHZ2;PRDM15;OTUD7A;FHIT;EHBP1;EFTUD1;SGCD;RPS6KA5;SPTLC2;SNTG1;CCDC30;SAMD12;SPECC1;GABBR2;HFM1;MGMT;PLCL1;LHFPL3;KSR2;NAV2;MYRIP;MERTK;KY;B3GALTL;AGBL4;CACNB2;NBEA;BRE;DPYD;COL6A5;ASTN2;ST6GALNAC3;DOCK1;FREM2
H4K20me1 keratinocyte hg19	41/2000	1.0	WDR27;SLC24A2;OCA2;ATP8A2;PLEKHB2;ADK;MIR663A;CACNA1D;LRP2;CACNA1C;MIR96;ARHGAP22;PPP3CA;DACH1;LARGE;FSD2;NCKAP5;EPHB1;PLXNA4;CORIN;SH3GL2;EPHA5;DYNC1H1;TRPC5;WSCD1;ANKRD30BL;GRID1;TRPC4;COL25A1;SEMA6D;HUNK;MYRIP;SEZ6L;ROCK1P1;MIR3648;DAB1;BAGE2;MIR3687;CDC42EP3;MIR183;WDPCP
H3K4me3 fibroblast of mammary gland hg19	41/2005	1.0	ACSS3;ZNF493;RTN1;PLEKHB2;DNAH8;MAST2;NOTCH2NL;FMN1;NPAS3;AKAP13;DACH1;PCDHA4;SRGAP3;DIP2C;LRRC4C;PRKG1;GPM6B;SH3GL2;PCDHA6;RUNX1T1;EPHA5;DTNA;HFM1;AUTS2;COL25A1;DCC;IFNGR2;MERTK;TEKT4P2;PARD3B;PTPRD;FRAS1;NFIA;ZNF717;ASXL3;TTLL11;PRKD1;PLCB1;EIF4G3;DOCK1;PDZRN4
H3K36me3 Caco-2 hg19	5/382	1.0	MIR3648;LINC00273;ANKRD30BL;MIR3687;MIR663A
H3K4me3 fibroblast of lung hg19	117/5182	1.0	PLEKHB2;DACH1;RPS6KA5;PIEZO2;HMCN1;DIP2C;SAMD12;NCKAP5;TBC1D22A;EPHB1;SH3GL2;ANKS1B;HFM1;ZNF283;DSCAM;CACNA2D1;MAGI2;KAZN;PCDHA10;CACNB2;ZNF717;WDPCP;TTLL11;PRKD1;ASTN2;FREM2;RTN1;CUL5;TSHZ2;PCDH15;TMTC1;CACNA1D;FHIT;NPAS3;ARHGAP22;ADAMTS17;PCDHA4;PCDHA3;HIVEP3;SRGAP3;LRRC4C;PCDHA7;PLXNA4;PCDHA6;SPECC1;AUTS2;LRBA;DNAH14;PHKB;FRG1B;KY;SDK1;CDK6;FGF14;DIAPH3;NBEA;CPE;EIF4G3;KIAA0825;SPAG16;ZNF493;CSRNP3;MAST2;ADK;SLC35F1;ARHGAP6;BICD1;GPHN;MRPL33;PTPRG;ADAMTSL1;AKAP13;PRKG1;PACRG;TRPC4;COL25A1;DCC;IFNGR2;HUNK;NRG1;TEKT4P2;SUMF1;PARD3B;PLCB4;IFT43;CDC42EP3;PLCB1;ST6GALNAC3;DOCK1;PON3;ACSS3;SGMS1;DNAH8;DNAH6;NOTCH2NL;HDAC9;RELN;ANKRD36BP2;DMD;CTNNA3;CSMD3;PCBD2;GPC6;CORIN;RUNX1T1;GABBR2;PCDH9;ATRNL1;PLCL1;VWA3B;ELP4;MERTK;AGBL4;FRAS1;NFIA;ASXL3;APBA1
H3K4me2 GM12878 hg19	40/2000	1.0	ITGAM;RTN1;EDA;CTBP2;DNAH8;LRRK2;NOTCH2NL;PTPRM;SLC35F1;CACNA1C;RERG;ARHGAP22;ADAMTS17;HIVEP3;CTNNA3;FPGT-TNNI3K;ZNF585A;ATRNL1;ANKRD30BL;COL25A1;PLCL1;HUNK;UNC5C;LHFPL3;VWA3B;MYRIP;MERTK;ROCK1P1;PTK2;PARD3B;PTPRD;PGM5P2;INTS4L1;ASXL3;MYO5B;SCN8A;CPE;CNTN4;XKR4;FREM2
H3K4me3 kidney mm9	40/2000	1.0	SPAG16;PON3;KHDRBS2;ACSS3;EDA;TENM4;PRDM15;OTUD7A;PTPRM;MRPL33;ADAMTSL1;SGCD;RPS6KA5;SPTLC2;ADAMTS17;HMCN1;SAMD12;NCKAP5;RUNX1T1;EPHA5;GABBR2;MGMT;GRID1;SEMA6D;PLCL1;MAGI2;UNC5D;KSR2;SEZ6L;MERTK;FOXP2;B3GALTL;TIAM2;AGBL4;CACNB2;NB

			EA;DPYD;ST6GALNAC3;MAPRE2;FREM2
H3K4me2 osteoblast hg19	40/2000	1.0	*RTN1;EDA;SGMS1;LAMA2;DNAH8;TSHZ2;DNAH6;HDAC9;RERG;HAPLN1;MIPOL1;PAK1;DACH1;RPS6KA5;PCDHA4;PCDHA3;GPC5;SAMD12;SH3GL2;ZNF585A;CNTN5;AUTS2;MGMT;GRID1;COL22A1;PHKB;TRAPPC8;VWA3B;MYRIP;PARD3B;PTPRD;TIAM2;AGBL4;CACNB2;ZNF717;ASXL3;CPE;ATP6V0D2;KIAA0825;CNTNAP4*
H3K4me1 mononuclear cell hg19	40/2000	1.0	*ALK;FHOD3;PON3;RTN1;CLSTN2;CUL5;LAMA2;DNAH6;MIR663A;LRP2;CACNA1C;SLC8A1;GPHN;RERG;CA3;NDST3;CSMD3;MIR4461;EPHB1;PLXNA4;ADAMTS6;CSGALNACT1;PACRG;HFM1;ZNF283;DNAH14;ANK3;KY;SUMF1;STXBP5L;MIR3648;DLG2;IFT43;ELMO1;CPE;TTLL11;ERG;ZFPM2;MAPRE2;PDZRN4*
H3K4me3 fibroblast of dermis hg19	40/2000	1.0	*FHOD3;CSRNP3;RTN1;SGMS1;DNAH6;GLIS3;FHIT;HDAC9;RERG;DACH1;ADAMTS17;PCDHA4;ANKRD36BP2;PCDHA3;PCBD2;SAMD12;SH3GL2;ZNF585A;KIRREL3;SPECC1;COL25A1;LRBA;VWA3B;ELP4;ANK3;MYRIP;MERTK;KY;PTK2;PTPRD;AGBL4;MYO3B;BAGE2;ASXL3;ELMO1;CPE;WDPCP;PLCB1;ZFPM2;FREM2*
H3K4me3 H1-hESC hg19	40/2000	1.0	*SPAG16;SGMS1;PDE1C;ANKRD36;CTNND2;DNAH6;HDAC9;MIPOL1;NPAS3;RPS6KA5;ADAMTSL3;SLIT3;GPR139;FPGT-TNNI3K;ERC1;PCBD2;SAMD12;PCDHA8;TBC1D22A;ANKS1B;DSCAM;AUTS2;ATRNL1;COL25A1;COL22A1;SUSD1;ARAP2;ZNF804A;ELP4;MYRIP;UPK3B;FAM155A;DIAPH3;BRE;ZNF717;ASXL3;DPYD;WDPCP;APBA1;CNTNAP4*
H3K27ac brain mm9	40/2000	1.0	*INVS;FHOD3;AVEN;KHDRBS2;CLSTN2;TENM4;DGKB;WRB;LRRK2;PRDM11;ROBO1;ENOX2;SGCD;RPS6KA5;HIVEP3;SRGAP3;NCKAP5;GPC6;SCAI;SH3GL2;SPECC1;GABBR2;WSCD1;PLCL1;EXOC6B;KSR2;MYRIP;PCDHA11;ARHGAP24;CACNB2;MSRA;MCM3AP;CDK6;FRAS1;NBEA;NSG2;BRE;DLC1;ST6GALNAC3;FBXL7*
H3K4me3 brain microvascular endothelial cell hg19	42/2101	1.0	*ROBO2;ZNF493;MAST2;TSHZ2;NOTCH2NL;TMTC1;LRRC16A;RYR3;NPAS3;AKAP13;SYNDIG1;CSMD3;HMCN1;NCKAP5;PLXNA4;PRKG1;SH3GL2;ANKS1B;RUNX1T1;CNTN5;AUTS2;DCC;LRBA;DNAH14;MAGI2;HUNK;KY;PARD3B;PTPRD;SDK1;PLCB4;FRAS1;NFIA;ZNF717;ASXL3;CPE;TTLL11;PRKD1;XKR4;EIF4G3;MAPRE2;DOCK1*
H3K4me3 fibroblast of foreskin hg19	28/1505	1.0	*ZNF493;NOTCH2NL;LRRC16A;FHIT;BICD1;NPAS3;PTPRG;AKAP13;HMCN1;N4BP2;HFM1;AUTS2;COL25A1;PLCL1;FNDC3B;VWA3B;TEKT4P2;KY;PARD3B;PGM5P2;INTS4L1;CDK6;NFIA;NBEA;ZNF717;TTLL11;ZNF675;EIF4G3*
H3K4me1 CD14-positive monocyte hg19	39/2000	1.0	*ALK;KHDRBS2;ACSS3;CLSTN2;CUL5;PDE1C;MAST2;MIR663A;TMTC1;CACNA1C;PRDM11;SAMSN1;DACH1;CA3;ADAMTS17;SPOCK3;ANKRD36BP2;SPOCK1;HMCN1;LRRC4C;KIFAP3;GPM6B;PACRG;DTNA;ZNF283;ATRNL1;MGMT;COL25A1;RANBP17;PHKB;SEZ6L;MERTK;ROCK1P1;KIAA1217;CACNB2;TMEM135;MYO5B;ATP6V0D2;PDZRN4*
H3K4me1 skeletal muscle myoblast hg19	39/2000	1.0	*RTN1;DGKB;LRRK2;SATB2;GLIS3;PCDH15;LRP2;SLC8A1;GPHN;RERG;HAPLN1;ARHGAP22;ADAMTS17;SPOCK3;DMD;FSD2;HIVEP3;GPC5;SRGAP3;SH3GL2;DTNA;ZNF283;CNTN5;COL22A1;MAGI2;TACR3;AKR1C2;UPK3B;SUMF1;MIR3648;FGF14;IFT43;MYO3B;MIR3687;DLC1;TTLL11;ERG;TLN2;UTRN*
H3K27me3 HepG2 hg19	39/2000	1.0	*SPAG16;PON3;KHDRBS2;ZNF493;RTN1;CTNND2;LRRK2;DNAH6;LRP2;FHIT;GPHN;RERG;MIPOL1;DACH1;NDST3;CTNNA3;PCDHA7;ZNF585A;CLVS1;ABCG8;TRPC5;COL25A1;PLCL1;PDE4D;MAGI2;PHKB;ANK2;ELP4;FAM126B;STXBP5L;DIAPH3;BRE;BAGE2;MIR1324;ASXL3;DLC1;CDH13;WDPCP;KIAA0825*
H3K4me3 limb mm9	39/2000	1.0	*SPAG16;PON3;KHDRBS2;ACSS3;ATP8A2;PLEKHB2;NPAS3;SGCD;RPS6KA5;SPTLC2;ZMAT4;CSMD3;SRGAP3;NCKAP5;RUNX1T1;PCDHAC1;SPECC1;GABBR2;PRMT8;EPHA6;HFM1;CNTN5;DCC;SEMA6D;PLCL1;MAGI2;FBXL17;KSR2;MERTK;KY;AGBL4;MCM3AP;NFIA;NBEA;NSG2;MYO5B;XKR4;DOCK1;FREM2*
H3K4me3 placenta mm9	39/2000	1.0	*SEMA5A;SPAG16;KHDRBS2;CLSTN2;LRRK2;TSHZ2;GLIS3;PTPRM;FMN1;EHBP1;EFTUD1;CDH4;SPTLC2;ZMAT4;CCDC30;SPOCK1;GPC5;HMCN1;SH3GL2;GABBR2;HFM1;DSCAM;COL25A1;SEMA6D;PLCL1;TRAPPC8;LHFPL3;KSR2;MYRIP;MERTK;KY;AGBL4;CACNB2;MCM3AP;CDK6;MYO5B;NF1;XKR4;ST6GALNAC3*
H3K4me3 osteoblast hg19	38/2000	1.0	*ATP8A2;RTN1;SGMS1;DNAH8;DNAH6;FHIT;HDAC9;MIPOL1;NPAS3;RPS6KA5;ADAMTS17;PCDHA4;GPC5;FPGT-TNNI3K;SAMD12;PCDHA7;ANKS1B;PCDHA6;ZNF585A;PCDH9;AUTS2;MGMT;COL25A1;DCC;PDE4D;TRAPPC8;VWA3B;KY;PARD3B;PTPRD;AGBL4;CACNB2;DAB1;ZNF717;APBA1;ASTN2;PLCB1;ST6GALNAC3*
H2AFZ H1-	38/2000	1.0	*ITGAM;ADK;SLC35F1;LRRC16A;EFTUD1;ENOX2;IQCJ-*

hESC hg19			*SCHIP1;PCDHA1;FSD2;HIVEP3;PCDHA2;CSMD3;FPGT-TNNI3K;SAMD12;KIFAP3;TBC1D22A;ADAMTS6;PRKG1;EYS;SH3GL2;PCDHAC1;PACRG;ZNF283;LRBA;PDE4D;TACR3;ELP4;PCDHA13;ARHGAP24;MGAT4C;BRE;ZNF717;TCF4;PRKD1;ERG;COL6A5;XKR4;SKAP2*
H3K4me1 thymus mm9	38/2000	1.0	*ATP8A2;CLSTN2;PDE1C;CPQ;MAST2;OTUD7A;GRIK4;BACH1;TMEM163;BICD1;SGCD;SRGAP3;ERC1;NCKAP5;TBC1D22A;HFM1;ATRNL1;IFNGR2;DIO2;ARAP2;TACR3;LHFPL3;ELOVL7;MERTK;SUMF1;STXBP5L;PARD3B;RUNX1;TIAM2;AGBL4;IFT43;NBEA;LANCL3;TTLL11;APBA1;ST6GALNAC3;MAPRE2;DOCK1*
H3K9me3 fibroblast of dermis hg19	38/2000	1.0	*DPP10;INVS;PTPRT;CNTNAP2;KHDRBS2;THSD7B;CTNND2;ADCY2;MIR663A;BACH1;ADAMTSL1;NKAIN2;DACH1;ADAMTSL3;ZMAT4;MACROD2;KIFAP3;ADAMTS6;CA10;DSCAM;GABRA3;RANBP17;DIO2;COBL;KAZN;FRG1B;SEZ6L;ROCK1P1;TEKT4P2;PTPRD;SPATA16;MIR1324;SCN8A;DPYD;TTLL11;ERG;XKR4;CNTNAP4*
H3K4me3 astrocyte hg19	38/2000	1.0	*OCA2;SGMS1;DNAH8;SATB2;DNAH6;ADK;FHIT;HDAC9;RERG;RPS6KA5;ADAMTS17;PCBD2;SAMD12;TBC1D22A;N4BP2;ANKS1B;TRPC5;AUTS2;ATRNL1;MGMT;GABRA3;TACR3;TRAPPC8;LHFPL3;VWA3B;ELP4;MYRIP;KY;AGBL4;MCM3AP;ANKFN1;DAB1;MYO3B;BRE;BAGE2;ST6GALNAC3;KIAA0825;FREM2*
H3K4me3 WI38 hg19	49/2521	1.0	*ATP8A2;PLEKHB2;LRRK2;MAST2;FMN1;BICD1;RERG;MRPL33;PTPRG;AKAP13;SAMD12;ANKS1B;TRPC4;HUNK;NRG1;TEKT4P2;ARID1B;PARD3B;TMEM135;ZNF717;CDC42EP3;TTLL11;PLCB1;ST6GALNAC3;DGKI;RTN1;CTBP2;DNAH8;SATB2;DNAH6;NOTCH2NL;TMTC1;CACNA1C;HDAC9;PAK1;RELN;PLXNA4;AUTS2;LRBA;PLCL1;SUSD1;LHFPL3;FRG1B;PGM5P2;DAB1;CDK6;NFIA;TBXAS1;CPE*
H3K27me3 G1E mm9	50/2569	1.0	*SPAG16;FHOD3;CNTNAP2;RYR2;THSD7B;BICD1;ADAMTSL1;AKAP13;HMCN1;WLS;CSGALNACT1;ABCG8;PACRG;EPHA6;DIO2;COBL;UNC5C;SYN3;UPK3B;FOXP2;TIAM2;DNM3;CACNB2;MYO3B;LANCL3;PRKD1;ZFPM2;OCA2;KHDRBS2;RTN1;PON1;TMTC1;LRP2;LRRC16A;ENOX2;PAK1;SH3TC2;SNTG1;PLXNA4;RUNX1T1;MGAM;PLCL1;LHFPL3;MERTK;LRP1B;STXBP5L;MSRA;DLC1;CPE;XKR4*
H4K20me1 GM12878 hg19	37/2000	1.0	*WDR27;ALK;ATP8A2;RTN1;EDA;PLEKHB2;NTM;ADK;FMN1;MIR663A;SLC35F1;LRP2;CACNA1C;RYR3;GPHN;LARGE;PIEZO2;CSMD3;ERC1;CLVS1;GABBR2;HUNK;KAZN;MYRIP;SYN3;ROCK1P1;FOXP2;KY;MIR3648;NCOR1;FRAS1;MIR3687;MIR1324;MYO5B;SCN8A;TTLL11;MAPRE2*
H3K4me1 keratinocyte hg19	37/2000	1.0	*ALK;ACSS3;LAMA2;DNAH6;FMN1;MIR663A;GPHN;RERG;NDST3;SPOCK3;ANKRD36BP2;SLIT3;CSMD2;EPHB1;GPC6;PCDHAC1;CSGALNACT1;EPHA5;KIRREL3;PACRG;PCDH9;ANKRD30BL;COL25A1;COL22A1;MYRIP;UPK3B;ROCK1P1;KY;SUMF1;MIR3648;DLG2;IFT43;MYO3B;TTLL11;ERG;ST6GALNAC3;FREM2*
H2AFZ keratinocyte hg19	37/2000	1.0	*WDR27;LAMA2;DNAH8;MAST2;ADK;LRRC16A;RERG;MRPL33;SYNDIG1;FSD2;SLIT3;KIFAP3;PLXNA4;SH3GL2;PCDHAC1;SPECC1;PCDH9;COL22A1;ARAP2;KAZN;PHKB;NRG1;LHFPL3;VWA3B;PCDHA13;FRG1B;FAM126B;ARID1B;PCDHA10;PARD3B;DAB1;DIAPH3;ASXL3;MYO5B;ST6GALNAC3;FREM2;SKAP2*
H3K4me1 Panc1 hg19	37/2000	1.0	*ROBO2;OCA2;CUL5;PLEKHB2;LRRK2;CHRM5;PCDH15;FMN1;HAPLN1;ROBO1;SH3TC2;NDST3;FAM110B;CSMD3;SRGAP3;HMCN1;CORIN;CSGALNACT1;EPHA5;PACRG;HFM1;TRPC4;PLCL1;DNAH14;EXOC6B;ANK2;VWA3B;KSR2;ANK3;UPK3B;SGCZ;LRP1B;FGF14;IFT43;UTRN;ST6GALNAC3;MAPRE2*
H3K4me1 HepG2 hg19	37/2000	1.0	*LINC00284;KHDRBS2;CSRNP3;CUL5;LRRK2;DNAH6;GLIS3;FMN1;CACNA1C;LRRC16A;DACH1;RGS3;NDST3;ADAMTS17;SPOCK3;MACROD2;HMCN1;LRRC4C;NCKAP5;EPHB1;SLC22A10;PACRG;ZNF283;PCDH9;CNTN5;IFNGR2;ZNF804A;ANK2;UPK3B;SUMF1;STXBP5L;MIR3648;PTPRD;DLG2;TTLL11;TLN2;ST6GALNAC3*
H2AFZ fibroblast of dermis hg19	37/2000	1.0	*SPAG16;OCA2;RTN1;SGMS1;SYCP2;SATB2;ADK;FMN1;FHIT;HDAC9;RERG;MRPL33;PPP3CA;AKAP13;PAK1;PCDHA3;KIFAP3;NCKAP5;TBC1D22A;PCDHA7;SPECC1;LRBA;HUNK;TACR3;VWA3B;ELP4;FRG1B;MERTK;KY;ARHGAP24;PARD3B;PGM5P2;BAGE2;CPE;APBA1;PRKD1;ST6GALNAC3*
H3K4me3 thymus mm9	37/2000	1.0	*SPAG16;PON3;SGMS1;PDE1C;LRRK2;TSHZ2;FHIT;EHBP1;ENOX2;RPS6KA5;SPTLC2;ADAMTS17;SRGAP3;HMCN1;SAMD12;PLXNA4;SH3GL2;RUNX1T1;SPECC1;HFM1;PLCL1;GAREM;UNC5C;KSR2;MERTK;LCA5L;B3GALTL;TIAM2;DNM3;CACNB2;MCM3AP;MYO5B;DPYD;ST6GALNAC3;DOCK1;FREM2;SKAP2*
H3K9me3 ES-Bruce4 mm9	7/570	1.0	*PCP4;TENM4;WSCD1;CPQ;MYO5B;PCDHA8;PTPRG*

H3K4me3 mammary epithelial cell hg19	73/3567	1.0	*ALK;PLEKHB2;CTNND2;LRRK2;MAST2;RYR3;TTC28;ADAMTSL1;AKAP13;ADAMTSL3;DLGAP1;ERC1;SAMD12;NCKAP5;TBC1D22A;ANKS1B;PCDHAC1;CHST9;KLF12;DCC;CACNA2D1;COBL;KAZN;UNC5D;PCDHA13;MYRIP;TEKT4P2;PLCB4;ZNF717;CDC42EP3;WDPCP;TTLL11;ASTN2;PLCB1;ST6GALNAC3;DOCK1;FREM2;DGKI;RTN1;CTBP2;SGMS1;DNAH8;DNAH6;NOTCH2NL;CACNA1C;MIPOL1;NPAS3;ZMAT4;SPOCK3;SYNDIG1;PCDHA3;DMD;PCBD2;RUNX1T1;PCDH9;CNTN5;AUTS2;ATRNL1;LRBA;PLCL1;PHKB;VWA3B;ELP4;KY;PTPRD;AGBL4;SDK1;PGM5P2;CDK6;ASXL3;CPE;XKR4;FBXL7*
H3K4me3 erythroblast mm9	52/2674	1.0	*SEMA5A;INVS;FHOD3;CLSTN2;LRRK2;OTUD7A;GRIK4;FMN1;GPHN;TTC28;CCDC91;MACROD2;ERC1;SAMD12;KIFAP3;CSGALNACT1;KIRREL3;HFM1;ARAP2;HUNK;UNC5C;KSR2;FAM126B;TIAM2;VWA8;TBC1D5;FREM2;ACSS3;SGMS1;PRDM15;LRP2;LRRC16A;EHBP1;HIVEP3;GPC5;N4BP2;SPECC1;ATRNL1;MGMT;PHKB;LHFPL3;ELP4;PTK2;STXBP5L;IGSF11;AGBL4;SDK1;FAM155A;MYO5B;NF1;APBA1;XKR4*
H3K4me3 Jurkat hg19	38/2071	1.0	*GABRB3;CNTNAP2;ZNF493;SYCP2;PLEKHB2;MAST2;NOTCH2NL;FHIT;GPHN;AKAP13;DACH1;RPS6KA5;ADAMTS17;HIVEP3;SRGAP3;PCBD2;DIP2C;SAMD12;PCDHA8;EPHB1;SH3GL2;HFM1;ZNF283;AUTS2;LRBA;PLCL1;DNAH14;HUNK;TRAPPC8;MYRIP;FRG1B;MERTK;PTPRD;MSRA;NBEA;TTLL11;PLCB1;EIF4G3*
H4K20me1 mammary epithelial cell hg19	36/2000	1.0	*ALK;RYR2;CLSTN2;PLEKHB2;LAMA2;PTPRM;MIR663A;HDAC9;PTPRG;ADAMTSL3;LARGE;CSMD2;PLXNA4;MDN1;CLVS1;CSGALNACT1;CHST9;PRMT8;CA10;ANKRD30BL;GRID1;COL25A1;HUNK;ZNF804A;LHFPL3;UNC5D;ROCK1P1;ARHGAP24;MIR3648;PTPRD;SPATA16;MIR3687;ASXL3;SCN8A;MAPRE2;PDZRN4*
H4K20me1 astrocyte hg19	36/2000	1.0	*ALK;OCA2;ATP8A2;CLSTN2;WRB;CTNND2;MIR663A;LRP2;TMEM163;BICD1;ADAMTSL1;NKAIN2;DACH1;N4BP2;SCAI;CORIN;ANKS1B;CSGALNACT1;KIRREL3;PRMT8;WSCD1;TRPC4;DCC;PLCL1;TRAPPC8;ELOVL7;FRG1B;SEZ6L;ROCK1P1;DNM3;MIR1324;HECW1;DPYD;CPE;ST6GALNAC3;MAPRE2*
H2AFZ osteoblast hg19	36/2000	1.0	*ZNF493;EDA;SGMS1;WRB;PON1;DNAH6;ADK;LRRC16A;HDAC9;RERG;MRPL33;MIPOL1;ARHGAP22;DACH1;SYNDIG1;PCDHA3;CTNNA3;SLIT3;KIFAP3;SH3GL2;ZNF283;SHFM1;ZNF804A;FRG1B;FAM126B;MERTK;ARHGAP24;PTPRD;DIAPH3;BAGE2;WDPCP;APBA1;TCF4;ST6GALNAC3;FREM2;CNTNAP4*
H3K4me1 cortical plate mm9	36/2000	1.0	*GABRB3;ALK;ATP8A2;CSRNP3;RTN1;CUL5;PDE1C;DGKB;MAST2;LRP2;TMEM163;TTC28;ARHGAP22;SGCD;ADAMTSL3;ASIC2;NCKAP5;ADAMTS6;CORIN;PACRG;WSCD1;MGMT;SUSD1;KAZN;PHKB;ANK2;ELP4;TNNI3K;TRDN;SLC2A9;ANKFN1;MYO5B;HECW1;DPYD;DOCK1;FBXL7*
H3K4me3 LNCaP clone FGC hg19	27/1591	1.0	*PLEKHB2;MAST2;NOTCH2NL;CACNA1D;CACNA1C;BICD1;HDAC9;GPHN;MRPL33;PTPRG;DACH1;PCDHA2;NCKAP5;N4BP2;EPHA6;DTNA;HFM1;AUTS2;PLCL1;FRG1B;TEKT4P2;PARD3B;NFIA;NBEA;ZNF717;CPE;EIF4G3*
H3K4me3 GM12865 hg19	24/1451	1.0	*DTNA;RTN1;PLEKHB2;AUTS2;COL25A1;DCC;LRBA;MAST2;TSHZ2;MYRIP;ARHGAP6;GPHN;B3GALTL;ARHGAP22;RPS6KA5;ZNF717;SCN8A;CDC42EP3;TTLL11;SRGAP3;PCBD2;EIF4G3;N4BP2;ADAMTS6*
H2AFZ A549 hg19	61/3109	1.0	*GABRB3;INVS;CNTNAP2;CSRNP3;ADK;SLC35F1;PTPRG;PPP3CA;DACH1;SAMD12;KIFAP3;NCKAP5;TBC1D22A;EPHB1;ADAMTS6;ZNF283;ARAP2;TACR3;UNC5D;FAM126B;UPK3B;MIR3648;WDPCP;ST6GALNAC3;ZNF675;ATP6V0D2;DOCK1;SKAP2;WDR27;ACSS3;RTN1;TSHZ2;DNAH6;CACNA1D;LRRC16A;MIPOL1;ARHGAP22;PCDHA1;ZMAT4;PCDHA5;PCDHA4;ANKRD36BP2;CSMD3;PCBD2;PCDHA8;PCDHA7;PLXNA4;ATRNL1;LRBA;ZNF804A;MERTK;SGCZ;ARHGAP24;MGAT4C;PGM5P2;DIAPH3;BRE;ZNF618;CPE;CNTN4;EIF4G3*
H3K4me1 erythroblast mm9	50/2632	1.0	*SEMA5A;ALK;FHOD3;ATP8A2;EDA;CLSTN2;CPQ;LRRK2;OTUD7A;GRIK4;FMN1;GPHN;TTC28;AKAP13;FAM110B;MACROD2;ERC1;SOX6;KIFAP3;CSGALNACT1;PACRG;FNDC3B;KSR2;FOXP2;LCA5L;PARD3B;TIAM2;VWA8;DNM3;IFT43;FREM2;SKAP2;CACNA1C;LRRC16A;TMEM163;EHBP1;GPC5;CORIN;ATRNL1;MGMT;EXOC6B;ELP4;STXBP5L;IGSF11;AGBL4;SDK1;FRAS1;NBEA;NF1;CCDC171*
H3K4me1 cerebellum mm9	35/2000	1.0	*ROBO2;PON3;ACSS3;CSRNP3;MIR96;GRM5;SGCD;SNTG1;ADAMTSL3;PIEZO2;HMCN1;SOX6;NCKAP5;OPCML;CSGALNACT1;SPECC1;DTNA;WSCD1;MGMT;SUSD1;TACR3;COBL;NRG1;MYRIP;MERTK;SORCS3;KY;ANO2;ARHGAP24;SUMF1;LCA5L;FRAS1;DPYD;CNTN4;SHANK2*
H3K4me2 K562 hg19	35/2000	1.0	*SPAG16;OCA2;CNTNAP2;PON3;SLC35F3;ANKRD36;TSHZ2;DNAH6;LRP2;ARHGAP15;ARHGAP22;SRGAP3;SAMD12;TB*

			C1D22A;ANKS1B;ZNF585A;GABBR2;TRPC4;GABRA3;ARAP2;ANK2;MYRIP;MERTK;ROCK1P1;ARHGAP24;PARD3B;RUNX1;AGBL4;NBEA;ZNF717;MYO5B;DPYD;TTLL11;COL6A5;ST6GALNAC3
H3K4me3 GM12875 hg19	35/2000	1.0	*SPAG16;PON3;MAST2;NOTCH2NL;NRXN3;MIR663A;SLC35F1;PTPRG;AKAP13;PAK1;DACH1;PIEZO2;DLGAP1;SCAI;ZNF283;PCDH9;AUTS2;LRBA;PLCL1;DNAH14;TEKT4P2;ARID1B;NFIA;ZNF717;MIR3687;MYO5B;SCN8A;NF1;ELMO1;CPE;TTLL11;ASTN2;XKR4;ST6GALNAC3;EIF4G3*
H3K4me1 osteoblast hg19	35/2000	1.0	*LINC00284;PLEKHB2;DNAH6;LRRC16A;GPHN;RERG;DACH1;KYNU;GPC5;CTNNA3;NCKAP5;TBC1D22A;EPHB1;WLS;PACRG;DTNA;HFM1;COL25A1;IFNGR2;DNAH14;MAGI2;EXOC6B;TRAPPC8;MERTK;SUMF1;MIR3648;TIAM2;FGF14;DLG2;MIR3687;CPE;WDPCP;TTLL11;ERG;ATP6V0D2*
H3K4me2 CD14-positive monocyte hg19	35/2000	1.0	*SEMA5A;ALK;SPAG16;ACSS3;CLSTN2;DNAH6;CACNA1C;AFF3;BICD1;RERG;MIPOL1;NPAS3;RPS6KA5;SYNDIG1;LRRC4C;PLXNA4;MDN1;SH3GL2;ZNF585A;ZNF283;AUTS2;MGMT;NRG1;ELP4;SEZ6L;KY;KIAA1217;CACNB2;PGM5P2;DIAPH3;ERG;COL6A5;ZFPM2;DOCK1;PDZRN4*
H3K9me3 keratinocyte hg19	35/2000	1.0	*NCOR1P1;PTPRT;CNTNAP2;KHDRBS2;CTNND2;SATB2;MIR663A;ARHGAP6;GRM1;TTC28;ADAMTSL1;ZNF280D;ARHGAP22;IQCJ-SCHIP1;SH3TC2;ADAMTSL3;ZMAT4;PIEZO2;ANKRD36BP2;GPR139;MACROD2;PCDHA6;DNAH14;GABRA3;RANBP17;KAZN;NAV2;SYN3;ROCK1P1;CNBD1;KIAA1217;SPATA16;FAT3;XKR4;CNTNAP4*
H3K4me3 HepG2 hg19	70/3586	1.0	*FHOD3;ZNF493;ATP8A2;PLEKHB2;CTNND2;MAST2;FMN1;MIR663A;SLC35F1;ARHGAP6;BICD1;AKAP13;RPS6KA5;KYNU;DLGAP1;SAMD12;TBC1D22A;EPHB1;ABCG8;KLF12;MAGI2;ARAP2;COBL;MYRIP;PLCB4;BAGE2;MIR3687;SCN8A;TTLL11;PRKD1;PLCB1;MAPRE2;DOCK1;DGKI;OCA2;PON3;KHDRBS2;RTN1;SGMS1;SATB2;TSHZ2;DNAH6;NOTCH2NL;CACNA1C;ARHGAP22;PAK1;LARGE;ADAMTS17;SPOCK3;PCBD2;N4BP2;PCDHA7;SCAI;ZNF585A;GABBR2;AUTS2;LRBA;PLCL1;TRAPPC8;ELP4;FRG1B;ARHGAP24;PTPRD;AGBL4;SDK1;NFIA;BRE;ASXL3;KIAA0825;FBXL7*
H2AFZ skeletal muscle myoblast hg19	34/2000	1.0	*CNTNAP2;CTNND2;ADK;ADCY2;FHIT;MRPL33;ARHGAP22;RELN;SH3TC2;PCDHA3;DLGAP1;SLIT3;FPGT-TNNI3K;SRGAP3;SAMD12;MDN1;ADAMTS6;GABBR2;WSCD1;DCC;SHFM1;HUNK;UNC5D;FRG1B;MERTK;ARHGAP24;PARD3B;AGBL4;SDK1;INTS4L1;BRE;WDPCP;DGKI;SKAP2*
H3K9me3 HeLa-S3 hg19	34/2000	1.0	*SLC24A2;ZNF493;CLSTN2;CTNND2;DNAH6;MIR663A;FRG2C;HDAC9;PCDHA1;SPTLC2;SH3TC2;PCDHA3;FSD2;CSMD3;FPGT-TNNI3K;GPC6;CALN1;ZNF585A;SNRPN;DSCAM;COL25A1;COL22A1;UNC5C;FRG1B;PCDHA12;ROCK1P1;FAM135B;SPATA16;ASXL3;SCN8A;TTLL11;TLN2;CNTN4;SHANK2*
H3K4me1 myotube hg19	34/2000	1.0	*SLC24A2;ACSS3;PLEKHB2;LRRK2;NRXN3;MIR663A;LRP2;ARHGAP6;EHBP1;GPHN;MRPL33;NDST3;DMD;HIVEP3;CTNNA3;SRGAP3;SCAI;ZNF585A;EPHA5;ANKRD30BL;TRPC4;EXOC6B;TACR3;AKR1C2;UPK3B;SUMF1;TRDN;TIAM2;MYO3B;DLC1;CPE;TLN2;LINC00486;FBXL7*
H3K9ac ES-E14 mm9	34/2000	1.0	*SEMA5A;ALK;KHDRBS2;CTBP2;LRRK2;MAST2;ADK;ADAMTSL1;SGCD;RELN;LARGE;ERC1;HMCN1;OPCML;EPHA5;CNTN5;ATRNL1;IFNGR2;HUNK;KAZN;ELP4;SEZ6L;PCDHA11;HS3ST4;KY;ANO2;PTPRD;AGBL4;MCM3AP;DAB1;CDC42EP3;CREBRF;TCF4;XKR4*
H3K9me3 NT2-D1 hg19	25/1583	1.0	*PTPRM;MIR663A;ZNF280D;RGS3;PCDHA1;CCDC30;PCDHA3;PCDHA2;MACROD2;PCDHA7;PCDHAC1;LINC00273;ANKRD30BL;DNAH14;RANBP17;PCDHA13;SYN3;ROCK1P1;PCDHA11;ANO2;MIR3648;IGSF11;B3GALTL;SLC2A9;MIR3687*
H4K20me1 myotube hg19	33/2000	1.0	*OCA2;CSRNP3;CHRM5;GLIS3;MIR663A;PRDM11;EHBP1;RERG;SGCD;DHX32;PRRG1;PCDHA3;DMD;FSD2;HIVEP3;KIFAP3;ANKS1B;WLS;PCDH9;ANKRD30BL;SHFM1;ZNF804A;ANK2;ROCK1P1;DNM3;MCM3AP;NCOR1;DLG2;MYO3B;DPYD;PRKD1;UTRN;KIAA0825*
H3K4me3 lung mm9	33/2000	1.0	*PON3;ACSS3;EDA;CLSTN2;LRRC16A;FHIT;MRPL33;EFTUD1;ADAMTSL1;ENOX2;RPS6KA5;ZMAT4;ADAMTS17;SRGAP3;SAMD12;NCKAP5;EPHB1;RUNX1T1;SPECC1;HFM1;SEMA6D;MAGI2;SUSD1;KSR2;SUMF1;B3GALTL;AGBL4;CACNB2;IFT43;MYO5B;DPYD;ST6GALNAC3;FREM2*
H2AFZ astrocyte hg19	33/2000	1.0	*KHDRBS2;RTN1;SGMS1;DNAH8;SATB2;RELN;KYNU;PIEZO2;PCBD2;KIFAP3;MDN1;DSCAM;COL25A1;CACNA2D1;LRBA;SHFM1;ZNF804A;TACR3;TRAPPC8;ELP4;MYRIP;FAM126B;ARHGAP24;AGBL4;ANKFN1;TMEM135;MYO3B;WDPCP;APBA1;PRKD1;XKR4;ST6GALNAC3;SKAP2*
H3K4me1 lung mm9	33/2000	1.0	*PTPRT;ACSS3;RTN1;CLSTN2;SLC35F3;TSHZ2;CACNA1C;FHIT;NPAS3;RELN;ZMAT4;GPC5;MDN1;SH3GL2;PACRG;PCDH9;MGMT;GRID1;COL25A1;KAZN;ELOVL7;KSR2;SEZ6L*

			;CACNB2;FAM155A;DAB1;MYO5B;NF1;CPE;APBA1;XKR4;DGKI;CCDC171
H3K4me3 bone marrow macrophage mm9	33/2000	1.0	DPP10;EDA;CLSTN2;PRDM15;ARHGAP15;FHIT;MRPL33;RPS6KA5;CCDC30;MACROD2;SRGAP3;HMCN1;SAMD12;RUNX1T1;PCDHAC1;SPECC1;GABBR2;HFM1;DSCAM;SEMA6D;PLCL1;GAREM;SUSD1;KSR2;FOXP2;TIAM2;AGBL4;CACNB2;NBEA;BRE;DPYD;ST6GALNAC3;DOCK1
H3K9ac heart mm9	33/2000	1.0	SEMA5A;ACSS3;SGMS1;ADK;GRIK4;LRRC16A;ADAMTSL1;SGCD;RELN;ERC1;TBC1D22A;MDN1;EPHA5;ATRNL1;COL25A1;LRBA;MAGI2;DIO2;ARAP2;UNC5C;ANK2;TNNI3K;MYRIP;TIAM2;AGBL4;MCM3AP;STIM1;MYO5B;DLC1;APBA1;XKR4;DGKI;SKAP2
H3K4me3 endothelial cell of umbilical vein hg19	66/3497	1.0	MAST2;ARHGAP6;RERG;AKAP13;PIEZO2;DLGAP1;MACROD2;HMCN1;DIP2C;SAMD12;TBC1D22A;PRKG1;ANKS1B;COL25A1;MAGI2;TACR3;CACNB2;PLCB4;ZNF717;MIR3687;WDPCP;ASTN2;PLCB1;MAPRE2;FREM2;PDZRN4;RTN1;SGMS1;DNAH8;DNAH6;NOTCH2NL;CACNA1C;LRRC16A;TMEM163;NPAS3;RELN;ADAMTS17;PCDHA4;PCDHA3;SLIT3;SRGAP3;PCBD2;N4BP2;SPECC1;PCDH9;WSCD1;LRBA;DNAH14;SUSD1;ZNF804A;VWA3B;AGBL4;SDK1;PGM5P2;FAM155A;MCM3AP;FRAS1;NFIA;ASXL3;MYO5B;TBXAS1;CPE;CNTN4;EIF4G3;KIAA0825;FBXL7
H3K4me3 GM12878 hg19	69/3640	1.0	SEMA5A;ITGAM;PLEKHB2;MAST2;ARHGAP6;BICD1;GPHN;RERG;PTPRG;AKAP13;RPS6KA5;KYNU;DLGAP1;CHST9;KLF12;COL25A1;CACNA2D1;ROCK1P1;MIR3648;DNM3;INTS4L1;IFT43;STIM1;ZNF717;MIR3687;SCN8A;HECW1;PLCB1;ST6GALNAC3;OCA2;PON3;KHDRBS2;RTN1;DNAH8;TSHZ2;NRXN3;FHIT;ARHGAP22;ADAMTS17;SPOCK3;ANKRD36BP2;HIVEP3;FPGT-TNNI3K;PCBD2;LRRC4C;SCAI;ZNF585A;SPECC1;DTNA;PCDH9;AUTS2;ATRNL1;LRBA;PLCL1;DNAH14;SUSD1;VWA3B;MERTK;PTK2;B3GALTL;PGM5P2;MCM3AP;FRAS1;NFIA;ZNF618;MYO5B;CPE;XKR4;EIF4G3
H3K4me2 CH12.LX mm9	32/2000	1.0	FHOD3;AVEN;ITGAM;ADK;GRIK4;FMN2;LRRC16A;TTC28;PAK1;SGCD;ADAMTS17;HIVEP3;CTNNA3;ERC1;PLXNA4;CSGALNACT1;DTNA;ATRNL1;LRBA;ANK2;FAM126B;PTK2;IGSF11;TIAM2;VWA8;CACNB2;FGF14;STIM1;NSG2;NF1;APBA1;DSCAML1
H3K4me2 B cell hg19	32/2000	1.0	SPAG16;AVEN;CNTNAP2;PON3;CACNA1D;PRDM11;LRRC16A;RERG;MIPOL1;NPAS3;ARHGAP22;FSD2;MACROD2;N4BP2;ADAMTS6;PCDHAC1;GABBR2;TRPC4;PLCL1;SUSD1;HUNK;COBL;HS3ST4;KIAA1217;AGBL4;PGM5P2;INTS4L1;WDPCP;PLCB1;KIAA0825;DGKI;PDZRN4
H3K27ac cortical plate mm9	32/2000	1.0	INVS;AVEN;CLSTN2;TENM4;CPQ;LRRK2;PTPRM;FHIT;PTPRG;ROBO1;ENOX2;RPS6KA5;GRM7;SRGAP3;DIP2C;GPC6;RUNX1T1;DSCAM;WSCD1;IFNGR2;SEMA6D;SHFM1;MAGI2;EXOC6B;KSR2;MYRIP;FAM126B;B3GALTL;MSRA;NFIA;DLC1;ST6GALNAC3
H2AFZ GM12878 hg19	32/2000	1.0	WDR27;INVS;AVEN;RTN1;SGMS1;PLEKHB2;DNAH8;TSHZ2;DNAH6;NRXN3;MRPL33;FSD2;FPGT-TNNI3K;PCBD2;LRRC4C;TBC1D22A;MDN1;LRBA;PLCL1;DNAH14;ZNF804A;VWA3B;FAM126B;TEKT4P2;DAB1;BRE;ZNF717;MIR1324;CDC42EP3;CPE;CNTN4;ST6GALNAC3
H3K9ac HeLa-S3 hg19	32/2000	1.0	SPAG16;INVS;SGMS1;SLC35F3;BICD1;MIPOL1;PPP3CA;RPS6KA5;FPGT-TNNI3K;SAMD12;TBC1D22A;N4BP2;ADAMTS6;ZNF585A;XRCC4;MGMT;CACNA2D1;LRBA;COBL;PHKB;TRAPPC8;ELOVL7;ELP4;FRG1B;FAM126B;FOXP2;TMEM135;DIAPH3;STIM1;NBEA;BAGE2;TLN2
H3K9ac CD14-positive monocyte hg19	32/2000	1.0	WDR27;SPAG16;FHOD3;PON3;ACSS3;SGMS1;MAST2;DNAH6;LRRC16A;FHIT;AKAP13;CA3;PCBD2;SAMD12;MDN1;ZNF585A;KLF12;MGMT;COL25A1;DNAH14;ANK2;ELP4;FRG1B;ROCK1P1;PTK2;ARHGAP24;DIAPH3;WDPCP;TTLL11;ST6GALNAC3;DSCAML1;KIAA0825
H3K4me3 Panc1 hg19	60/3295	1.0	CNTNAP2;ATP8A2;CLSTN2;PLEKHB2;MAST2;ADK;SLC35F1;GPHN;DACH1;RPS6KA5;ERC1;EPHB1;DSCAM;GRID1;TRPC4;HUNK;TACR3;PCDHA13;FAM126B;TEKT4P2;PARD3B;MIR3648;DNM3;CACNB2;ZNF717;CDC42EP3;WDPCP;FREM2;DGKI;ACSS3;RTN1;SGMS1;LAMA2;TSHZ2;DNAH6;NOTCH2NL;FHIT;NPAS3;ARHGAP22;ADAMTS17;SPOCK3;PCDHA4;HIVEP3;GPC5;CSMD3;SRGAP3;LRRC4C;PCDHA8;N4BP2;GPC6;LINC00478;GABBR2;AUTS2;MGMT;VWA3B;KY;AGBL4;BRE;EIF4G3;KIAA0825
H3K4me3 HCT116 hg19	32/2040	1.0	PON3;ZNF493;CTBP2;PLEKHB2;MAST2;NOTCH2NL;CACNA1D;GPHN;PTPRG;AKAP13;RPS6KA5;SRGAP3;PCBD2;PRKG1;ANKS1B;PCDHAC1;KLF12;HFM1;LRBA;VWA3B;ANK3;TEKT4P2;ARHGAP24;PARD3B;PLCB4;IFT43;NFIA;STIM1;MIR3687;TTLL11;EIF4G3;DOCK1
H4K20me1	31/2000	1.0	INVS;OCA2;CNTNAP2;ATP8A2;MIR663A;PRDM11;LRRC16

K562 hg19			A;BICD1;EHBP1;GRM1;CDH4;GPR139;MIR4461;GPM6B;ABCG8;LRBA;TRAPPC8;ELP4;FRG1B;FAM126B;ROCK1P1;TEKT4P2;MIR3648;NCOR1;STIM1;MIR3687;MIR1324;APBA1;PRKD1;ZFPM2;KIAA0825
H3K4me1 ES-Bruce4 mm9	31/2000	1.0	GABRB3;PON3;CSRNP3;SYCP2;LAMA2;DGKB;ADCY2;THSD4;RERG;HAPLN1;SH3TC2;KYNU;HIVEP3;PCDHA2;SOX6;OPCML;CCDC178;DCC;COL22A1;SUSD1;NRG1;ANK2;NAV2;AGBL4;FRAS1;FGF14;DLC1;HECW1;TBXAS1;TTLL11;ST6GALNAC3
H3K4me1 H1-hESC hg19	31/2000	1.0	PLEKHB2;TSHZ2;PON1;NRXN3;GRIK4;LRP2;SEL1L2;GRM1;PIEZO2;ANKRD36BP2;GPR139;FPGT-TNNI3K;CA10;DSCAM;GABRA5;MGMT;COL25A1;SEMA6D;DNAH14;MAGI2;UPK3B;FAM135B;PCP4;DAB1;FGF14;ELMO1;MIR183;CDH13;TLN2;COL6A5;CNTNAP4
H2AFZ mammary epithelial cell hg19	31/2000	1.0	CNTNAP2;LRRC16A;ARHGAP6;RYR3;RERG;NPAS3;RELN;ADAMTS17;DLGAP1;SLIT3;PCBD2;NCKAP5;SPECC1;ATRNL1;CACNA2D1;LRBA;SHFM1;NRG1;LHFPL3;VWA3B;PCDHA13;FRG1B;FAM126B;KY;PCDHA10;PARD3B;AGBL4;PGM5P2;CDC42EP3;ATP6V0D2;FBXL7
H3K79me2 liver mm9	31/2000	1.0	INVS;MAST2;PON1;CACNA1D;LRRC16A;BACH1;GPHN;ENOX2;SPTLC2;HIVEP3;DYNC1H1;KLF12;XRCC4;ATRNL1;IFNGR2;SHFM1;VPS13D;TRAPPC8;ELP4;FAM126B;SUMF1;PARD3B;TMEM135;NCOR1;STIM1;BRE;DPYD;NF1;UTRN;DOCK1;SKAP2
H4K20me1 skeletal muscle myoblast hg19	31/2000	1.0	INVS;SGMS1;MIR663A;BACH1;TMEM163;RERG;GRM1;EFTUD1;NKAIN2;RELN;PLXNA4;SH3GL2;EPHA5;GABBR2;PCDH9;WSCD1;ANKRD30BL;TRPC4;SEMA6D;PLCL1;EXOC6B;TRAPPC8;ELOVL7;SEZ6L;ROCK1P1;MIR3648;B3GALTL;NCOR1;MIR3687;MIR1324;DGKI
H3K9ac GM12878 hg19	31/2000	1.0	SPAG16;FHOD3;AVEN;SGMS1;LRRK2;ADK;LRRC16A;ARHGAP22;RPS6KA5;HIVEP3;FPGT-TNNI3K;N4BP2;MDN1;ADAMTS6;CORIN;SPECC1;XRCC4;ATRNL1;PLCL1;TRAPPC8;ELP4;FRG1B;ROCK1P1;TEKT4P2;ARHGAP24;DNM3;NCOR1;DIAPH3;NFIA;ZNF717;EIF4G3
H3K4me1 bone marrow macrophage mm9	31/2000	1.0	DPP10;FHOD3;PON3;ITGAM;CPQ;CACNA1C;TMEM163;FHIT;CCDC91;KYNU;PRRG1;HIVEP3;CTNNA3;MACROD2;CORIN;WLS;PCDHAC1;PACRG;AUTS2;PLCL1;GAREM;EXOC6B;FBXL17;ELOVL7;STXBP5L;NBEA;MYO5B;SCN8A;CPE;XKR4;ATP6V0D2
H3K9ac A549 hg19	31/2000	1.0	DNAH6;FHIT;SPOCK3;SYNDIG1;PCDHA4;SRGAP3;PCBD2;SAMD12;TBC1D22A;ADAMTS6;PCDHAC1;GABRA5;MGMT;DCC;HUNK;TRAPPC8;ELOVL7;ELP4;MYRIP;MERTK;PTPRD;AGBL4;PGM5P2;FGF14;DIAPH3;STIM1;MIR3687;SCN8A;ST6GALNAC3;DSCAML1;KIAA0825
H3K4me1 fibroblast of lung hg19	31/2000	1.0	OCA2;RTN1;PLEKHB2;LAMA2;MIR663A;SLC8A1;GPHN;ARHGAP22;SGCD;DHX32;SH3TC2;NDST3;PRRG1;HIVEP3;SRGAP3;ZNF385D;CSMD2;GPM6B;CORIN;LINC00478;CSGALNACT1;DTNA;COL25A1;SEMA6D;VWA3B;FGF14;MYO3B;DLC1;CPE;TTLL11;ERG
H3K4me1 liver mm9	68/3706	1.0	ALK;FHOD3;ATP8A2;CSRNP3;CLSTN2;SLC35F3;DGKB;CPQ;CTNND2;MAST2;PTPRM;FMN1;AFF3;BICD1;TTC28;ADAMTSL1;RPS6KA5;CCDC91;KYNU;SAMD12;SOX6;NCKAP5;EPHB1;CSGALNACT1;EPHA5;KIRREL3;ABCG8;COL25A1;VPS13D;ANK2;KSR2;ANK3;SUMF1;PARD3B;DNM3;NCOR1;CDC42EP3;LANCL3;ATP6V0D2;SKAP2;PON3;PDE1C;CACNA1D;CACNA1C;SAMSN1;ZMAT4;LARGE;CCDC30;HIVEP3;CORIN;SPECC1;DTNA;MGMT;COL22A1;SGCZ;KY;PTK2;STXBP5L;AGBL4;FRAS1;NSG2;MYO5B;DLC1;TBXAS1;APBA1;XKR4;FBXL7;CCDC171
H3K4me3 B cell hg19	48/2843	1.0	INVS;RTN1;EDA;MAST2;PRDM15;NOTCH2NL;MIR663A;LRP2;CACNA1C;BICD1;RERG;MRPL33;PTPRG;AKAP13;ADAMTS17;ANKRD36BP2;FSD2;MACROD2;LRRC4C;EPHB1;N4BP2;GPM6B;ZNF283;AUTS2;GRID1;COL25A1;PLCL1;SUSD1;HUNK;ELOVL7;MYRIP;TEKT4P2;FOXP2;ARHGAP24;AGBL4;SDK1;PGM5P2;TMEM135;CDK6;FRAS1;ZNF717;MIR3687;NF1;CPE;TTLL11;PRKD1;EIF4G3;KIAA0825
H3K4me3 GM06990 hg19	27/1852	1.0	RTN1;EDA;PLEKHB2;DNAH8;MAST2;BICD1;PTPRG;KYNU;DLGAP1;SRGAP3;PCBD2;DIP2C;EPHB1;ANKS1B;AUTS2;LRBA;MYRIP;FRG1B;TEKT4P2;PGM5P2;FRAS1;NFIA;STIM1;ZNF717;ASXL3;EIF4G3;MAPRE2
H3K9ac myotube hg19	30/2000	1.0	INVS;MAST2;DNAH6;ADK;LRRC16A;BACH1;MIPOL1;PRRG1;FPGT-TNNI3K;PCBD2;N4BP2;PCDH9;XRCC4;ATRNL1;MGMT;COL25A1;LRBA;IFNGR2;TRAPPC8;ELP4;FAM126B;B3GALTL;PGM5P2;DIAPH3;STIM1;MYO3B;ZNF717;CPE;KIAA0825;SKAP2
H2AFZ myotube hg19	30/2000	1.0	ATP8A2;CSRNP3;SGMS1;RYR3;MRPL33;ARHGAP22;DACH1;RPS6KA5;ADAMTS17;PCDHA3;FPGT-TNNI3K;SRGAP3;PCDHA7;MDN1;SCAI;COL25A1;LRBA;AR

			AP2;HUNK;TACR3;ELP4;SEZ6L;PARD3B;AGBL4;TMEM135;BRE;CDC42EP3;CPE;PLCB1;SKAP2
H3K27ac cerebellum mm9	30/2000	1.0	*INVS;ATP8A2;SGMS1;PDE1C;DGKB;LRRK2;TMEM163;FHIT;EHBP1;MRPL33;ROBO1;ENOX2;RPS6KA5;SPTLC2;SH3TC2;CSMD3;CALN1;GABBR2;HFM1;DSCAM;SEMA6D;MAGI2;PHKB;KSR2;KY;MSRA;BRE;LANCL3;XKR4;MAPRE2*
H4K20me1 HepG2 hg19	30/2000	1.0	*INVS;OCA2;EDA;CTNND2;MIR663A;LRP2;TMEM163;NKAIN2;SPTLC2;ADAMTS17;SLIT3;CORIN;CLVS1;LINC00273;CHST9;GABBR2;ANKRD30BL;PLCL1;KAZN;PHKB;TRAPPC8;ELP4;SEZ6L;ROCK1P1;MIR3648;MCM3AP;NCOR1;MIR3687;WDPCP;DGKI*
H3K9me3 H1-hESC hg19	30/2000	1.0	*NCOR1P1;SLC24A2;OCA2;DNAH8;FMN1;ADCY2;MIR663A;THSD4;PTPRG;ZNF280D;RPS6KA5;ADAMTSL3;LARGE;ANKRD36BP2;GPR139;MACROD2;CALN1;ZNF283;COL25A1;RANBP17;DIO2;KSR2;SYN3;ROCK1P1;STXBP5L;IGSF11;SPATA16;STIM1;TTLL11;FREM2*
H3K9ac liver mm9	30/2000	1.0	*FHOD3;SGMS1;LRRK2;MAST2;ADK;GRIK4;ADAMTSL1;CTNNA3;ERC1;HMCN1;MDN1;SPECC1;HFM1;XRCC4;ATRNL1;GRID1;COL25A1;CACNA2D1;ARAP2;HUNK;PHKB;ELP4;KSR2;FAM126B;MERTK;PTK2;MCM3AP;DAB1;STIM1;CPE*
H3K4me3 liver mm9	48/2880	1.0	*SPAG16;PON3;ACSS3;CLSTN2;TENM4;CTNND2;PRDM15;FHIT;MRPL33;EFTUD1;ADAMTSL1;SGCD;RPS6KA5;DHX32;FAM110B;ADAMTS17;CCDC30;SRGAP3;SAMD12;SCAI;RUNX1T1;CALN1;SPECC1;TRPC5;HFM1;MGMT;SEMA6D;PLCL1;MAGI2;SUSD1;FBXL17;PHKB;KSR2;MERTK;RUNX1;B3GALTL;TIAM2;VWA8;AGBL4;CACNB2;FAM155A;NBEA;MYO5B;DPYD;LANCL3;ST6GALNAC3;DOCK1;FREM2*
H3K27ac A549 hg19	50/2991	1.0	*INVS;MAST2;PTPRG;PPP3CA;RPS6KA5;SAMD12;NCKAP5;ADAMTS6;PCDHAC1;ARAP2;HUNK;ELOVL7;PCDHA12;UPK3B;TEKT4P2;DNM3;TMEM135;STIM1;SCN8A;WDPCP;ST6GALNAC3;RTN1;SGMS1;DNAH6;FHIT;MIPOL1;ZMAT4;PCDHA5;PCDHA4;ANKRD36BP2;PCDHA3;FPGT-TNNI3K;SRGAP3;MDN1;MGAM;XRCC4;WSCD1;GABRA5;ATRNL1;MGMT;LRBA;TRAPPC8;ELP4;MERTK;PTK2;PTPRD;SDK1;PGM5P2;DIAPH3;KIAA0825*
H3K9me3 A549 hg19	29/2000	1.0	*THSD7B;TPTE;MIR663A;THSD4;MIPOL1;ADAMTSL1;ADAMTSL3;LARGE;ANKRD36BP2;MIR4461;KIFAP3;CALN1;CSGALNACT1;ANKRD30BL;COL25A1;RANBP17;KSR2;MYRIP;FRG1B;ROCK1P1;TEKT4P2;ANO2;STXBP5L;KIAA1217;SLC2A9;DNM3;SDK1;MAPRE2;SHANK2*
H3K27me3 T-cell acute lymphoblastic leukemia hg19	29/2000	1.0	*DPP10;ITGAM;RTN1;NOTCH2NL;MIR663A;BICD1;MRPL33;IQCJ-SCHIP1;DACH1;DHX32;PCDHA1;SPTLC2;CTNNA3;MIR4461;MDN1;ZNF585A;SHFM1;DNAH14;ARAP2;LCA5L;CNBD1;MIR3648;INTS4L1;PLCB4;IFT43;MIR3687;MIR1324;DPYD;WDPCP*
H2AFZ endothelial cell of umbilical vein hg19	29/2000	1.0	*RTN1;LAMA2;TSHZ2;ADK;CACNA1C;MRPL33;ARHGAP22;DHX32;ADAMTS17;PIEZO2;SRGAP3;KIFAP3;SPECC1;WSCD1;CNTN5;ANKRD30BL;LRBA;HUNK;ZNF804A;VWA3B;ELP4;PCDHA10;AGBL4;PGM5P2;BRE;ASXL3;MYO5B;XKR4;KIAA0825*
H3ac C2C12 mm9	29/2000	1.0	*FHOD3;SGMS1;LAMA2;MAST2;ADK;FMN1;LRRC16A;BACH1;GPHN;MIPOL1;TTC28;PPP3CA;SGCD;CCDC30;ADAMTS6;ANKS1B;PLCL1;DIO2;ARAP2;UNC5C;PHKB;ELP4;FAM126B;VWA8;MCM3AP;NF1;CPE;TTLL11;APBA1*
H2AFZ fibroblast of lung hg19	29/2000	1.0	*EDA;SGMS1;DNAH8;DNAH6;GLIS3;BICD1;HDAC9;MRPL33;ZMAT4;SPOCK3;SAMD12;PCDHA8;PCDHA7;MDN1;SH3GL2;PCDHA6;PCDHAC1;SPECC1;ZNF283;GABRA5;PCDHA13;PCDHA10;PARD3B;DLG2;MYO3B;ASXL3;SCN8A;CPE;FREM2*
H3K4me1 fibroblast of dermis hg19	29/2000	1.0	*DGKB;DNAH6;LRP2;GPHN;NDST3;HIVEP3;GPC5;SRGAP3;CSMD2;SPECC1;HFM1;ZNF283;SEMA6D;PDE4D;DNAH14;VPS13D;EXOC6B;ANK2;VWA3B;ROCK1P1;IGSF11;B3GALTL;FGF14;TTLL11;ERG;LINC00486;ST6GALNAC3;FREM2;DGKI*
H3K4me1 K562 hg19	65/3684	1.0	*SPAG16;CNTNAP2;ITGAM;GRIK4;CA3;GPR139;HMCN1;SAMD12;ADAMTS6;PCDHAC1;PACRG;TRPC4;COL25A1;VPS13D;ARAP2;HUNK;MYRIP;UPK3B;ROCK1P1;TEKT4P2;KIAA1217;MIR3648;DNM3;CACNB2;SPATA16;TMEM135;IFT43;MIR3687;DPYD;TLN2;PLCB1;LINC00486;DSCAML1;PON3;CUL5;DNAH8;TSHZ2;PON1;LRRC16A;HDAC9;MIPOL1;ARHGAP22;RELN;PCDHA1;SH3TC2;ZMAT4;SRGAP3;GPC6;PLXNA4;SPECC1;ATRNL1;DNAH14;GABRA3;SUSD1;EXOC6B;VWA3B;MERTK;KY;ARHGAP24;STXBP5L;IGSF11;AGBL4;FRAS1;MIR183;KIAA0825*
H3K4me3 heart mm9	53/3158	1.0	*SPAG16;ATP8A2;EDA;CLSTN2;SLC35F3;DGKB;PTPRM;MRPL33;EFTUD1;CDH4;SGCD;RPS6KA5;SPTLC2;GRM7;SAMD12;NCKAP5;HFM1;DCC;SEMA6D;MAGI2;KSR2;MYRIP;FOXP2;ANO2;CACNB2;DPYD;LANCL3;ST6GALNAC3;DOCK1;FREM2;PON3;KHDRBS2;WRB;TSHZ2;PRDM11;FHIT;EHBP1;Z*

			MAT4;HIVEP3;SRGAP3;TRPM3;GABBR2;MGMT;PLCL1;GAREM;MERTK;KY;B3GALTL;AGBL4;FAM155A;NBEA;NSG2;MYO5B
H3K36me3 SK-N-SH hg19	5/707	1.0	*MIR3648;LINC00273;ANKRD30BL;MIR3687;MIR663A*
H3K9ac HepG2 hg19	28/2000	1.0	*SGMS1;ANKRD36;LRRC16A;BICD1;MIPOL1;PPP3CA;RPS6KA5;FPGT-TNNI3K;SRGAP3;N4BP2;PCDHA7;MDN1;ZNF585A;ABCG8;KLF12;HFM1;SUSD1;ARAP2;PHKB;TRAPPC8;PTK2;PARD3B;STIM1;BRE;MIR3687;SCN8A;TTLL11;KIAA0825*
H3K4me2 T-cell acute lymphoblastic leukemia hg19	28/2000	1.0	*SPAG16;AVEN;RTN1;ANKRD36;CACNA1C;PRDM11;LRRC16A;FHIT;ARHGAP22;FPGT-TNNI3K;SRGAP3;PCBD2;N4BP2;ADAMTS6;ZNF585A;SPECC1;HFM1;ZNF283;ATRNL1;DNAH14;ELP4;MIR3648;INTS4L1;NCOR1;PLCB4;MIR3687;SCN8A;CDC42EP3*
H3K27ac limb mm9	28/2000	1.0	*LAMA2;LRRK2;PRDM15;HAPLN1;MRPL33;PTPRG;ROBO1;ENOX2;SGCD;RPS6KA5;PCBD2;DIP2C;NCKAP5;SCAI;RUNX1T1;SPECC1;SHFM1;FAM126B;ARID1B;CACNB2;MCM3AP;CDK6;FRAS1;BRE;ST6GALNAC3;EIF4G3;DOCK1;FBXL7*
H3K27ac fibroblast of dermis hg19	28/2000	1.0	*SPAG16;SGMS1;ADK;NOTCH2NL;MIR663A;NPAS3;TTC28;PPP3CA;RPS6KA5;FPGT-TNNI3K;PCBD2;DIP2C;SAMD12;TBC1D22A;MDN1;DTNA;HFM1;LRBA;HUNK;FRG1B;ROCK1P1;RUNX1;MIR3648;ZNF717;MIR3687;DLC1;KIAA0825;SKAP2*
H3K79me2 keratinocyte hg19	28/2000	1.0	*ZNF493;WRB;MIR663A;MIR96;EFTUD1;RPS6KA5;FPGT-TNNI3K;SAMD12;N4BP2;SCAI;ZNF585A;ZNF283;XRCC4;DSCAM;MGMT;FRG1B;ROCK1P1;TEKT4P2;SUMF1;MIR3648;MSRA;PGM5P2;FRAS1;ZNF717;MIR3687;MIR183;WDPCP;UTRN*
H3K9ac fibroblast of lung hg19	28/2000	1.0	*RTN1;HDAC9;ADAMTSL1;PPP3CA;AKAP13;MACROD2;SRGAP3;PCBD2;SAMD12;TBC1D22A;PCDHA7;MDN1;GPM6B;XRCC4;MAGI2;FRG1B;FAM126B;SGCZ;FOXP2;PTK2;AGBL4;SDK1;FAM155A;FGF14;NFIA;STIM1;BRE;WDPCP*
H3K79me2 skeletal muscle myoblast hg19	28/2000	1.0	*SPAG16;AVEN;WRB;MIR663A;EFTUD1;ENOX2;DHX32;SPOCK1;FPGT-TNNI3K;SCAI;ZNF283;XRCC4;ANKRD30BL;COL25A1;CACNA2D1;MAGI2;VPS13D;ROCK1P1;TEKT4P2;SUMF1;MIR3648;PGM5P2;MCM3AP;BRE;ZNF717;MIR3687;SHANK2;SKAP2*
H3K4me3 ES-E14 mm9	52/3190	1.0	*DPP10;SPAG16;ATP8A2;PLEKHB2;LRRK2;ADK;ADAMTSL1;PPP3CA;SGCD;RPS6KA5;ERC1;HMCN1;CSGALNACT1;TRPC5;EPHA6;GRID1;SEMA6D;ARAP2;HUNK;UNC5C;NAV2;MYRIP;FAM126B;SEZ6L;ANO2;STIM1;CDC42EP3;FREM2;DGKI;SKAP2;KHDRBS2;LRRC16A;PCDHA1;CSMD1;PCDHA7;OPCML;CNTN5;ATRNL1;LRBA;GAREM;ELP4;MERTK;HS3ST4;KY;STXBP5L;PTPRD;SDK1;MCM3AP;NF1;APBA1;CNTN4;XKR4*
H3K4me3 C2C12 mm9	27/2000	1.0	*WDR27;INVS;FHOD3;AVEN;SGMS1;PLEKHB2;CPQ;PRDM11;LRRC16A;EHBP1;EFTUD1;TTC28;CCDC91;LARGE;CCDC30;CTNNA3;ATRNL1;LRBA;SEMA6D;PHKB;TRAPPC8;FAM126B;MERTK;VWA8;NBEA;CPE;MAPRE2*
H3K9ac mammary epithelial cell hg19	27/2000	1.0	*PDE1C;MAST2;DNAH6;LRRC16A;BACH1;PTPRG;ADAMTSL1;ARHGAP22;RPS6KA5;SPOCK3;FPGT-TNNI3K;ERC1;PCBD2;LRRC4C;CSGALNACT1;PRMT8;DTNA;MAGI2;PHKB;VWA3B;ELP4;MIR3648;DIAPH3;ZNF717;MIR3687;WDPCP;ATP6V0D2*
H3K4me2 HeLa-S3 hg19	27/2000	1.0	*CTBP2;SGMS1;LRRK2;PCDH15;MIR663A;DMD;ERC1;HMCN1;TBC1D22A;ADAMTS6;ANKS1B;ZNF585A;HFM1;XRCC4;EXOC6B;ELOVL7;ELP4;UPK3B;ROCK1P1;ARHGAP24;PARD3B;RUNX1;DNM3;NCOR1;BAGE2;CPE;DOCK1*
H3K4me3 spleen mm9	27/2000	1.0	*PON3;EDA;SAMSN1;FHIT;RPS6KA5;SPTLC2;SH3TC2;FAM110B;ADAMTS17;CCDC30;SRGAP3;SPECC1;HFM1;SEMA6D;PLCL1;SUSD1;MERTK;LCA5L;RUNX1;B3GALTL;TIAM2;MCM3AP;NBEA;MYO5B;ST6GALNAC3;DOCK1;FREM2*
H3K4me2 myocyte mm9	27/2000	1.0	*ROBO2;AVEN;PLEKHB2;DGKB;GRIK4;MIPOL1;DHX32;ADAMTSL3;CCDC30;GPC5;CTNNA3;SAMD12;LRRC4C;NCKAP5;TBC1D22A;PLCL1;DIO2;FBXL17;UNC5C;ELOVL7;MERTK;PARD3B;CPE;APBA1;TCF4;ZFPM2;CNTNAP4*
H3K79me2 fibroblast of dermis hg19	27/2000	1.0	*SEMA5A;AVEN;ZNF493;MIR663A;MIPOL1;EFTUD1;TTC28;ENOX2;LARGE;FPGT-TNNI3K;GPC6;LINC00478;ZNF283;ANKRD30BL;MGMT;FRG1B;ROCK1P1;TEKT4P2;SUMF1;RUNX1;FRAS1;BRE;ZNF717;MIR3687;WDPCP;APBA1;PLCB1*
H3K4me1 kidney mm9	27/2000	1.0	*GABRB3;DPP10;AVEN;PON3;DGKB;TMEM163;ADAMTSL1;GPC5;NCKAP5;DTNA;HFM1;WSCD1;MGMT;COL25A1;PHKB;LHFPL3;SEZ6L;SGCZ;FOXP2;ARHGAP24;LCA5L;NSG2;MYO3B;TBXAS1;NF1;COL6A5;DGKI*
H3K27ac olfactory	27/2000	1.0	*INVS;TENM4;TSHZ2;PTPRM;MRPL33;ENOX2;PPP3CA;RPS6KA5;SH3TC2;GABBR2;HFM1;WSCD1;SEMA6D;SHFM1;MAG*

bulb mm9			*I2;KSR2;FAM126B;MERTK;B3GALTL;STIM1;NBEA;BRE;DLC1;ELMO1;XKR4;ST6GALNAC3;MAPRE2*
H2AFZ HepG2 hg19	27/2000	1.0	*WDR27;SPAG16;OCA2;PLEKHB2;WRB;ADK;NOTCH2NL;FHIT;MRPL33;RPS6KA5;ADAMTS17;FPGT-TNNI3K;HMCN1;SAMD12;KIFAP3;NCKAP5;PCDHA7;SPECC1;ZNF283;DNAH14;ARAP2;COBL;FAM126B;ARHGAP24;MYO3B;BRE;PLCB1*
H3K27ac fibroblast of lung hg19	60/3617	1.0	*SPAG16;INVS;ZNF493;ADK;MIR663A;ARHGAP6;BICD1;MRPL33;AKAP13;HMCN1;SAMD12;NCKAP5;TBC1D22A;PRKG1;KLF12;TRPC4;CACNA2D1;IFNGR2;MAGI2;FAM126B;TEKT4P2;ARID1B;PARD3B;RUNX1;TMEM135;MIR3687;CDC42EP3;WDPCP;SKAP2;WDR27;SGMS1;LAMA2;DNAH6;PRDM15;NOTCH2NL;LRRC16A;MIPOL1;SH3TC2;PRRG1;FPGT-TNNI3K;PCBD2;LRRC4C;N4BP2;GPC6;MDN1;RUNX1T1;PCDH9;XRCC4;LRBA;SHFM1;PHKB;TRAPPC8;ELP4;FRG1B;ARHGAP24;B3GALTL;DIAPH3;BRE;EIF4G3;KIAA0825*
H3K9me3 erythroblast mm9	35/2445	1.0	*EDA;SYCP2;PCDH15;NRXN3;TMTC1;ARHGAP6;TMEM163;RYR3;RELN;PCDHA1;PCDHA5;SYNDIG1;HIVEP3;PCDHA9;PCDHA8;PCDHA7;PLXNA4;GPM6B;PCDHA6;RUNX1T1;PCDHAC1;CALN1;CCDC178;TACR3;MYRIP;PCDHA12;PCDHA10;FAM155A;FAM135B;FRAS1;MYO5B;LANCL3;MIR183;COL6A5;PDZRN4*
H3ac myocyte mm9	26/2000	1.0	*AVEN;MAST2;LRRC16A;GPHN;MRPL33;MIPOL1;TTC28;ADAMTSL3;SRGAP3;ERC1;TBC1D22A;CSGALNACT1;ATRNL1;SEMA6D;HUNK;UNC5C;FAM126B;PTK2;VWA8;MCM3AP;TMEM135;CPE;APBA1;TCF4;EIF4G3;SKAP2*
H3K9ac fibroblast of dermis hg19	26/2000	1.0	*SGMS1;DNAH6;MIR663A;LRRC16A;MIPOL1;NPAS3;PPP3CA;RPS6KA5;FPGT-TNNI3K;SAMD12;LRRC4C;N4BP2;SCAI;EPHA5;PCDH9;LRBA;PDE4D;MAGI2;PHKB;PTK2;LRP1B;PLCB4;FGF14;ZNF717;CPE;KIAA0825*
H3K27ac endothelial cell of umbilical vein hg19	26/2000	1.0	*SPAG16;INVS;SGMS1;DNAH8;DNAH6;PTPRM;MIR663A;DACH1;FPGT-TNNI3K;SAMD12;TBC1D22A;N4BP2;PLXNA4;MDN1;EPHA5;XRCC4;LRBA;PHKB;ELP4;FRG1B;FAM126B;SUMF1;FRAS1;ZNF717;KIAA0825;SKAP2*
H3K79me2 MEL cell line mm9	26/2000	1.0	*INVS;AVEN;CPQ;SAMSN1;EHBP1;TTC28;ENOX2;PPP3CA;CTNNA3;ERC1;PCBD2;XRCC4;LRBA;IFNGR2;PHKB;TRAPPC8;ELP4;SUMF1;NCOR1;STIM1;BRE;TBC1D5;CREBRF;TCF4;SKAP2;CCDC171*
H3K27ac osteoblast hg19	26/2000	1.0	*SPAG16;FHOD3;CSRNP3;SGMS1;LAMA2;LRRK2;DNAH6;CACNA1C;LRRC16A;MIPOL1;AKAP13;FPGT-TNNI3K;PCBD2;N4BP2;MDN1;CSGALNACT1;LRBA;ELP4;TEKT4P2;SUMF1;MIR3648;TIAM2;ZNF717;MIR3687;KIAA0825;SKAP2*
H3K9ac endothelial cell of umbilical vein hg19	26/2000	1.0	*SGMS1;DNAH8;DNAH6;SPOCK3;FPGT-TNNI3K;PCBD2;SAMD12;TBC1D22A;N4BP2;SPECC1;PCDH9;AUTS2;ATRNL1;MAGI2;PHKB;ELP4;FRG1B;PARD3B;STIM1;BRE;ASXL3;CPE;WDPCP;PLCB1;LINC00486;KIAA0825*
H2AFZ B cell hg19	26/2000	1.0	*WDR27;SPAG16;INVS;AVEN;RTN1;SGMS1;PDE1C;MRPL33;MIPOL1;HIVEP3;LRRC4C;TBC1D22A;WLS;SPECC1;ZNF283;TRPC4;IFNGR2;ZNF804A;VWA3B;FAM126B;SEZ6L;MERTK;SDK1;NFIA;WDPCP;PLCB1*
H3K36me3 heart mm9	26/2000	1.0	*INVS;AVEN;PON3;CSRNP3;MAST2;CACNA1D;SLC8A1;EHBP1;MIPOL1;ENOX2;GPC5;ERC1;ADAMTS6;KLF12;DTNA;XRCC4;PDE4D;ELP4;FAM126B;SGCZ;SUMF1;VWA8;NCOR1;NF1;UTRN;ZFPM2*
H4K20me1 fibroblast of lung hg19	26/2000	1.0	*WDR27;INVS;AVEN;CLSTN2;NOTCH2NL;MIR663A;HDAC9;PTPRG;DACH1;LARGE;FAM110B;FSD2;CSGALNACT1;LINC00273;KIRREL3;PRMT8;ANKRD30BL;COL25A1;VWA3B;ROCK1P1;MIR3648;NCOR1;MIR3687;SCN8A;ERG;MAPRE2*
H4K20me1 HeLa-S3 hg19	26/2000	1.0	*SLC24A2;INVS;OCA2;CHRM5;MIR663A;PRDM11;MIR96;BACH1;EHBP1;IQCJ-SCHIP1;DHX32;CA3;NDST3;GPR139;SRGAP3;HMCN1;MIR4461;ZNF283;PCDH9;ANK2;VWA3B;ROCK1P1;TMEM135;DAB1;BRE;ZNF717*
H3K27ac heart mm9	44/2941	1.0	*INVS;PON3;CUL5;PLEKHB2;DGKB;LRRK2;MAST2;PRDM15;PRDM11;FHIT;EHBP1;THSD4;HAPLN1;MRPL33;ROBO1;ENOX2;SGCD;RPS6KA5;SRGAP3;NCKAP5;TBC1D22A;SCAI;SPECC1;WSCD1;SEMA6D;SHFM1;PLCL1;EXOC6B;PHKB;TRAPPC8;ANK2;SUMF1;B3GALTL;CACNB2;MCM3AP;CDK6;FRAS1;IFT43;NBEA;BRE;ST6GALNAC3;MAPRE2;DOCK1;FREM2*
H3K4me1 heart mm9	58/3628	1.0	*DPP10;ALK;SPAG16;AVEN;ATP8A2;PLEKHB2;DGKB;CPQ;BICD1;SLC8A1;RERG;NCKAP5;PCDHAC1;KIRREL3;ABCG8;DSCAM;TRPC4;DIO2;HUNK;KAZN;NRG1;ANK2;ANK3;NAV2;SUMF1;DSCAML1;PDZRN4;SHANK2;SKAP2;KHDRBS2;RTN1;PDE1C;LAMA2;SATB2;NTM;CACNA1D;TMEM163;THSD4*

			;NPAS3;SH3TC2;CCDC30;SPOCK1;CSMD3;TRPM3;CORIN;SPECC1;XRCC4;CNTN5;MGMT;SUSD1;ELP4;LRP1B;STXBP5L;MSRA;NFIA;NSG2;ASXL3;CNTN4
H3K4me1 G1E-ER4 mm9	33/2415	1.0	PON3;PON1;LRP2;AFF3;BICD1;EHBP1;THSD4;TTC28;ADAMTSL1;DHX32;RGS3;ADAMTS17;CCDC30;LRRC4C;SOX6;KIFAP3;SCAI;ANKS1B;ABCG8;PACRG;ATRNL1;MGMT;RANBP17;SUSD1;EXOC6B;DIO2;MERTK;FOXP2;SUMF1;LCA5L;TMEM135;IFT43;LANCL3
H3K27me3 CH12.LX mm9	37/2618	1.0	SEMA5A;FHOD3;ITGAM;SYCP2;ANKRD36;TMEM163;ARHGAP22;PPP3CA;SGCD;GPC5;ERC1;SOX6;NCKAP5;PLXNA4;CALN1;OPCML;CSGALNACT1;KIRREL3;DYNC1H1;ABCG8;PRMT8;DSCAM;PLCL1;EXOC6B;MERTK;PTK2;STXBP5L;TIAM2;ANKFN1;PCP4;FGF14;STIM1;NSG2;SCN8A;ERG;ST6GALNAC3;DGKI
H3K27ac GM12878 hg19	25/2000	1.0	SEMA5A;SPAG16;PTPRT;FHOD3;SGMS1;SLC35F1;LRRC16A;AFF3;ARHGAP22;FPGT-TNNI3K;PCBD2;N4BP2;MDN1;ADAMTS6;CHST9;XRCC4;SUSD1;COBL;TRAPPC8;FRG1B;ROCK1P1;TEKT4P2;DIAPH3;ZNF618;SCN8A
H3K9me1 endothelial cell of umbilical vein hg19	25/2000	1.0	AVEN;OCA2;SGMS1;PLEKHB2;PTPRM;MIR663A;BACH1;SAMSN1;HDAC9;PTPRG;SH3TC2;PIEZO2;MDN1;LINC00273;IFNGR2;ROCK1P1;PARD3B;RUNX1;MIR3648;TMEM135;NFIA;BAGE2;MIR3687;SCN8A;DSCAML1
H4K20me1 T-cell acute lymphoblastic leukemia hg19	25/2000	1.0	MAST2;ADK;MIR663A;PRDM11;TTC28;ARHGAP22;SPTLC2;GPC5;EPHB1;CORIN;LINC00273;PCDH9;ANKRD30BL;LRBA;TRAPPC8;FAM126B;SEZ6L;UPK3B;ROCK1P1;B3GALTL;PCP4;NCOR1;MIR1324;EIF4G3;KIAA0825
H3K79me2 osteoblast hg19	25/2000	1.0	ZNF493;ANKRD36;MIR663A;LRRC16A;MIPOL1;TTC28;LARGE;FPGT-TNNI3K;HMCN1;N4BP2;ZNF585A;LINC00273;ZNF283;PCDH9;XRCC4;ANKRD30BL;DNAH14;FRG1B;ROCK1P1;TEKT4P2;SUMF1;LCA5L;MIR3648;ZNF717;MIR3687
H3K4me3 CD14-positive monocyte hg19	57/3639	1.0	ALK;SPAG16;CNTNAP2;MAST2;MIR663A;AFF3;MACROD2;HMCN1;EPHB1;SH3GL2;PCDHAC1;KLF12;GRID1;COL25A1;CACNA2D1;HUNK;SEZ6L;TEKT4P2;PARD3B;PLCB4;ZNF717;CDC42EP3;TTLL11;ZNF675;DOCK1;PDZRN4;PON3;KHDRBS2;ACSS3;DNAH8;NOTCH2NL;CACNA1C;LRRC16A;MIPOL1;SH3TC2;PCDHA3;N4BP2;MDN1;GPM6B;ZNF585A;AUTS2;ATRNL1;MGMT;LRBA;SUSD1;PHKB;ELP4;FRG1B;HS3ST4;KY;ARHGAP24;AGBL4;PGM5P2;CDK6;DIAPH3;NF1;APBA1
H3K4me3 HeLa-S3 hg19	55/3589	1.0	PLEKHB2;SLC35F3;ANKRD36;MAST2;ADK;BICD1;PPP3CA;AKAP13;SAMD12;KIFAP3;TBC1D22A;ADAMTS6;SH3GL2;ANKS1B;HFM1;GRID1;COBL;ELOVL7;UPK3B;ROCK1P1;TEKT4P2;FOXP2;PARD3B;DNM3;IFT43;WDPCP;TTLL11;MAPRE2;DOCK1;CTBP2;SGMS1;TSHZ2;NOTCH2NL;LRRC16A;PAK1;FPGT-TNNI3K;PCBD2;N4BP2;ZNF585A;XRCC4;LRBA;PLCL1;PHKB;TRAPPC8;ELP4;FRG1B;PTK2;ARHGAP24;PGM5P2;NFIA;NBEA;BRE;MYO5B;CPE;EIF4G3
H3K9me3 K562 hg19	24/2000	1.0	INVS;IFNGR2;DNAH14;PRDM15;ADK;PCDH15;MIR663A;FRG1B;ROCK1P1;TEKT4P2;MRPL33;ZNF280D;AKAP13;MCM3AP;RPS6KA5;NFIA;STIM1;ASXL3;SRGAP3;KIFAP3;ZNF675;EIF4G3;CALN1;ZNF585A
H3K79me2 GM12878 hg19	24/2000	1.0	LINC00273;SPAG16;ZNF493;ZNF283;XRCC4;ANKRD30BL;MGMT;WRB;LRRK2;ANK2;ROCK1P1;FHIT;TEKT4P2;SUMF1;MIR3648;ENOX2;MCM3AP;IFT43;MYO3B;ZNF717;MIR3687;GPC5;PCBD2;ZNF585A
H3K4me1 T-cell acute lymphoblastic leukemia hg19	24/2000	1.0	SPECC1;HFM1;ZNF283;CUL5;ATRNL1;IFNGR2;DNAH14;EXOC6B;ELP4;CACNA1C;PRDM11;FAM126B;LRRC16A;MERTK;ARHGAP22;DACH1;SCN8A;CDC42EP3;TTLL11;FPGT-TNNI3K;SRGAP3;MIR4461;EPHB1;ADAMTS6
H3K27ac bone marrow mm9	24/2000	1.0	INVS;AVEN;PLEKHB2;SEMA6D;PRDM11;FAM126B;MERTK;SAMSN1;ARID1B;PTK2;MRPL33;EFTUD1;B3GALTL;SLC2A9;CACNB2;MCM3AP;CDK6;RPS6KA5;BRE;SPTLC2;SH3TC2;DLC1;SRGAP3;SCAI
H3K4me3 mononuclear cell hg19	24/2000	1.0	PON3;ZNF283;MGMT;COL25A1;DNAH8;LRBA;DNAH6;ADK;PHKB;VWA3B;ELP4;FAM126B;BICD1;PTK2;MIR3648;FRAS1;DIAPH3;BRE;MYO5B;WDPCP;PLCB1;MIR4461;KIAA0825;MDN1
H3K9ac keratinocyte hg19	24/2000	1.0	SGMS1;XRCC4;AUTS2;MGMT;DNAH8;COL22A1;PHKB;VWA3B;ELP4;UPK3B;ROCK1P1;ADAMTSL1;PTPRD;RPS6KA5;DIAPH3;STIM1;ZNF717;CPE;WDPCP;FPGT-TNNI3K;ERC1;PCBD2;TBC1D22A;FREM2
H4K20me1 osteoblast	24/2000	1.0	SEMA5A;LINC00273;ALK;GABBR2;PCDH9;WRB;PLCL1;VPS13D;UNC5C;TRAPPC8;MIR663A;MERTK;ROCK1P1;TMEM1

hg19			*63;TEKT4P2;MIR3648;B3GALTL;MIR3687;MIR1324;WDPCP;ERC1;TBC1D22A;MDN1;LINC00478*
H3K4me3 K562 hg19	68/4606	1.0	*SPAG16;FHOD3;ZNF493;ATP8A2;PLEKHB2;SLC35F3;MAST2;BICD1;MRPL33;EFTUD1;PPP3CA;AKAP13;RPS6KA5;ERC1;SAMD12;ANKS1B;KLF12;TRPC4;MYRIP;FAM126B;ROCK1P1;TEKT4P2;PARD3B;DNM3;TMEM135;PLCB4;IFT43;ZNF717;BAGE2;MIR3687;CDC42EP3;DPYD;WDPCP;TTLL11;PRKD1;ST6GALNAC3;MAPRE2;OCA2;PON3;PDE1C;TSHZ2;DNAH6;NOTCH2NL;HDAC9;NPAS3;ARHGAP22;GPC5;SRGAP3;N4BP2;ZNF585A;GABBR2;PCDH9;LRBA;PLCL1;TRAPPC8;FRG1B;MERTK;ARHGAP24;AGBL4;SDK1;FAM155A;DIAPH3;NFIA;NBEA;BRE;MYO5B;EIF4G3;KIAA0825*
H3K4me1 MEL cell line mm9	40/4170	1.0	*FHOD3;PON3;CUL5;ANKRD36;CPQ;PRDM11;AFF3;SAMSN1;BICD1;GPHN;TTC28;PAK1;DHX32;SH3TC2;PRRG1;CTNNA3;SRGAP3;N4BP2;ADAMTS6;DYNC1H1;PACRG;IFNGR2;SUSD1;EXOC6B;DIO2;FBXL17;PHKB;ELOVL7;FAM126B;ARID1B;RUNX1;SLC2A9;TMEM135;NCOR1;IFT43;TBC1D5;HECW1;TBXAS1;CREBRF;ST6GALNAC3*
H3K4me3 CH12.LX mm9	54/4143	1.0	*FHOD3;AVEN;MAST2;ADK;GRIK4;GPHN;TTC28;PPP3CA;SGCD;SPTLC2;ERC1;CSGALNACT1;PACRG;DIO2;UNC5C;ELOVL7;FAM126B;UPK3B;PARD3B;TIAM2;VWA8;CACNB2;STIM1;CDC42EP3;ST6GALNAC3;MAPRE2;SKAP2;LRRC16A;ARHGAP15;TMEM163;FHIT;HIVEP3;GPC5;PCBD2;DTNA;XRCC4;ATRNL1;MGMT;LRBA;PLCL1;PHKB;TRAPPC8;ELP4;PTK2;STXBP5L;IGSF11;B3GALTL;MCM3AP;FRAS1;NSG2;BRE;NF1;APBA1;CCDC171*
H3K4me3 MEL cell line mm9	36/4088	1.0	*FHOD3;AVEN;PON3;PLEKHB2;LRRC16A;ARHGAP15;AFF3;MRPL33;EFTUD1;ENOX2;PPP3CA;RPS6KA5;DHX32;SPTLC2;ADAMTS17;SRGAP3;KIFAP3;PCDHA8;SPECC1;ATRNL1;LRBA;IFNGR2;SUSD1;TRAPPC8;FAM126B;SUMF1;LCA5L;B3GALTL;VWA8;IFT43;BRE;CDC42EP3;NF1;MAPRE2;SKAP2;CCDC171*
H3K36me3 CH12.LX mm9	19/4031	1.0	*WDR27;LRRC69;PDE1C;IFNGR2;WRB;SATB2;VPS13D;KAZN;CACNA1D;MIR96;EHBP1;SUMF1;TTC28;NCOR1;ELMO1;MIR183;PCBD2;EIF4G3;MDN1*
H3K4me1 CH12.LX mm9	45/3885	1.0	*SEMA5A;ITGAM;EDA;THSD7B;CPQ;MAST2;ATP1A4;TMTC1;FMN2;ARHGAP15;AFF3;TMEM163;EHBP1;RYR3;ARHGAP22;PAK1;SGCD;CCDC91;DHX32;ADAMTS17;CCDC30;GPC5;SRGAP3;CSGALNACT1;DYNC1H1;SUSD1;EXOC6B;DIO2;ANK2;SUMF1;STXBP5L;LCA5L;PARD3B;IGSF11;B3GALTL;TIAM2;DNM3;CACNB2;PCP4;FRAS1;DLG2;IFT43;TBXAS1;ST6GALNAC3;EIF4G3*
H3K9ac K562 hg19	43/3409	1.0	*SPAG16;OCA2;PON3;ATP8A2;SGMS1;ANKRD36;SATB2;MAST2;ADK;MIR663A;BICD1;HDAC9;MRPL33;EFTUD1;ARHGAP22;PPP3CA;FPGT-TNNI3K;SRGAP3;SCAI;ZNF585A;TRAPPC8;MYRIP;MERTK;UPK3B;ROCK1P1;PTK2;ARHGAP24;SUMF1;PARD3B;DNM3;AGBL4;NCOR1;PLCB4;NFIA;BRE;ZNF717;BAGE2;CDC42EP3;DPYD;TTLL11;PRKD1;ZFPM2;KIAA0825*
H3K36me3 MEL cell line mm9	13/3352	1.0	*WDR27;PON3;PACRG;CPQ;VPS13D;PRDM11;ARHGAP15;SUMF1;MIPOL1;EFTUD1;NCOR1;PRRG1;KIFAP3*
H3K36me3 A549 hg19	28/3112	1.0	*PLEKHB2;LRRK2;PTPRM;MIR663A;BACH1;EHBP1;ARHGAP22;LARGE;FSD2;FPGT-TNNI3K;DIP2C;MIR4461;TBC1D22A;MDN1;ZNF585A;CSGALNACT1;KIRREL3;CACNA2D1;PDE4D;RANBP17;FRG1B;ROCK1P1;PCDHA11;LCA5L;TIAM2;MCM3AP;TMEM135;DIAPH3*
H3K27ac liver mm9	36/3076	1.0	*PON3;SGMS1;CUL5;PLEKHB2;MAST2;TSHZ2;PRDM11;FHIT;EHBP1;MRPL33;ENOX2;RPS6KA5;SPTLC2;SH3TC2;TBC1D22A;CALN1;SPECC1;MGMT;SEMA6D;SHFM1;MAGI2;GAREM;SUSD1;PHKB;TRAPPC8;ELP4;FAM126B;MERTK;B3GALTL;MCM3AP;CDK6;BRE;MYO5B;MAPRE2;DOCK1;SKAP2*
H3K36me3 erythroblast mm9	10/3059	1.0	*SPECC1;SLC2A9;RELN;WRB;CTNNA3;FAM126B;UTRN;ST6GALNAC3;MDN1;TTC28*
H3K36me3 megakaryocyte mm9	6/3028	1.0	*SPECC1;PLCB4;SGMS1;BRE;PLEKHB2;ANKRD36*
H3K36me3 G1E mm9	13/3013	1.0	*SGMS1;IFNGR2;WRB;RANBP17;FBXL17;ANK2;ELP4;FAM126B;SUMF1;TTC28;ERC1;ST6GALNAC3;MDN1*
H3K27ac H1-hESC hg19	23/2000	1.0	*SPAG16;ACSS3;LRBA;MAST2;PRDM15;FBXL17;TRAPPC8;ELP4;MIPOL1;AKAP13;TMEM135;IFT43;NFIA;ZNF717;WDPCP;ERC1;PCBD2;EIF4G3;TBC1D22A;N4BP2;MDN1;SKAP2;OPCML*
H2AFZ HeLa-S3 hg19	23/2000	1.0	*SPAG16;SGMS1;SLC35F3;SHFM1;DNAH6;ADK;PHKB;ELP4;FAM126B;LRRC16A;BICD1;ARHGAP24;MRPL33;MIPOL1;EFTUD1;TMEM135;CPE;FPGT-TNNI3K;PCBD2;KIFAP3;ZNF675;ADAMTS6;SH3GL2*

H3K27ac HepG2 hg19	23/2000	1.0	*WDR27;ABCG8;ZNF493;DTNA;SGMS1;XRCC4;LRBA;FMN1;TRAPPC8;ELP4;SYN3;BICD1;TEKT4P2;MIPOL1;AKAP13;BRE;FPGT-TNNI3K;PCBD2;PLCB1;N4BP2;MDN1;ADAMTS6;SKAP2*
H3K4me3 T-cell acute lymphoblastic leukemia hg19	23/2000	1.0	*SPAG16;XRCC4;ATRNL1;MGMT;ANKRD36;DNAH14;ADK;PHKB;ELP4;CACNA1C;LRRC16A;FHIT;SUMF1;INTS4L1;PAK1;PLCB4;SCN8A;WDPCP;FPGT-TNNI3K;SRGAP3;KIAA0825;ADAMTS6;ZNF585A*
H3K27ac small intestine mm9	23/2000	1.0	*SPECC1;AVEN;ACSS3;DGKB;SEMA6D;SHFM1;LRRK2;MAGI2;TSHZ2;SUSD1;EXOC6B;PHKB;MRPL33;B3GALTL;ENOX2;CACNB2;MCM3AP;CDK6;SGCD;RPS6KA5;BRE;MAPRE2;SKAP2*
H2AFZ K562 hg19	23/2000	1.0	*WDR27;SEMA5A;PON3;KLF12;ZNF493;EDA;SLC35F3;AUTS2;LRBA;ANK2;ELP4;ARHGAP24;MRPL33;STXBP5L;IGSF11;AGBL4;NBEA;DPYD;TCF4;SRGAP3;SAMD12;KIFAP3;SKAP2*
H3K27ac astrocyte hg19	23/2000	1.0	*INVS;FHOD3;SGMS1;XRCC4;AUTS2;LAMA2;LRBA;ZNF804A;FMN1;PHKB;ELP4;FRG1B;FAM126B;MIPOL1;NPAS3;TMEM135;NFIA;CPE;WDPCP;FPGT-TNNI3K;PCBD2;TBC1D22A;KIAA0825*
H3K9ac skeletal muscle myoblast hg19	23/2000	1.0	*SPAG16;AUTS2;ATRNL1;MGMT;MAST2;DNAH6;GLIS3;PHKB;TRAPPC8;TEKT4P2;RERG;MIPOL1;LCA5L;ARHGAP22;ZNF717;CPE;WDPCP;PCBD2;SAMD12;TBC1D22A;N4BP2;KIAA0825;MDN1*
H3K9me1 keratinocyte hg19	23/2000	1.0	*WDR27;LINC00284;LRRC69;AVEN;PLEKHB2;TSHZ2;FMN1;PHKB;MIR663A;ELOVL7;FRG1B;BACH1;ROCK1P1;KY;THSD4;RUNX1;MIR3648;TMEM135;MIR3687;ADAMTS17;FSD2;APBA1;MIR4461*
H3K27ac myotube hg19	23/2000	1.0	*SGMS1;XRCC4;LAMA2;MAGI2;PHKB;TRAPPC8;FRG1B;FAM126B;TEKT4P2;PGM5P2;TMEM135;RPS6KA5;DIAPH3;MIR3687;CPE;SPOCK1;WDPCP;FPGT-TNNI3K;PCBD2;SAMD12;TBC1D22A;N4BP2;ZNF585A*
H3K36me3 endothelial cell of umbilical vein hg19	16/2929	1.0	*CSGALNACT1;LINC00273;PLEKHB2;ANKRD30BL;FNDC3B;MIR663A;BACH1;ROCK1P1;ANO2;MIR3648;TIAM2;MIR3687;FSD2;FPGT-TNNI3K;DOCK1;ADAMTS6*
H3K36me3 G1E-ER4 mm9	12/2903	1.0	*SGMS1;ANKRD36;WRB;TBXAS1;ERC1;FAM126B;ST6GALNAC3;MDN1;EHBP1;THSD4;SUMF1;TTC28*
H3K79me2 A549 hg19	34/2787	1.0	*WDR27;AVEN;WRB;MIR663A;THSD4;GPHN;MIPOL1;EFTUD1;TTC28;ENOX2;RPS6KA5;FPGT-TNNI3K;KIFAP3;N4BP2;SCAI;LINC00273;ZNF283;XRCC4;ELOVL7;UPK3B;ROCK1P1;TEKT4P2;SUMF1;LCA5L;PARD3B;MIR3648;IGSF11;FRAS1;IFT43;ZNF717;MIR3687;MIR183;WDPCP;UTRN*
H3K27me3 MEL cell line mm9	33/2672	1.0	*FHOD3;RYR2;ITGAM;CLSTN2;PON1;ATP1A4;TPTE;PRDM11;DHX32;RGS3;LIPI;NDST3;CCDC30;SOX6;ADAMTS6;PKNOX2;ABCG8;PACRG;MGAM;EXOC6B;ELOVL7;NAV2;MERTK;FAM227B;MSRA;NFIA;NBEA;NSG2;HECW1;LANCL3;APBA1;ERG;MAPRE2*
H3K27me3 G1E-ER4 mm9	26/2653	1.0	*SPAG16;RYR2;PLEKHB2;CHRM5;PON1;ATP1A4;TPTE;PAK1;SH3TC2;FSD2;CSMD3;SRGAP3;PRKG1;WLS;CSGALNACT1;ATRNL1;DIO2;LHFPL3;UPK3B;FOXP2;STXBP5L;DLG2;DLC1;CPE;ATP6V0D2;DGKI*
H3K36me3 keratinocyte hg19	16/2651	1.0	*LINC00273;LRRC69;ZNF283;ANKRD30BL;SHFM1;NOTCH2NL;MIR663A;BACH1;ROCK1P1;MRPL33;RUNX1;MIR3648;SH3TC2;MIR3687;TBC1D22A;DOCK1*
H3K36me3 K562 hg19	19/2634	1.0	*LINC00273;XRCC4;ANKRD30BL;PRDM15;FBXL17;MIR663A;FRG1B;ROCK1P1;RUNX1;EFTUD1;ZNF280D;MIR3648;SPTLC2;MIR3687;TBC1D5;NF1;GPC5;MIR4461;SCAI*
H3K4me3 G1E-ER4 mm9	29/2605	1.0	*KHDRBS2;THSD7B;MAST2;PON1;EHBP1;GPHN;TTC28;ADAMTSL3;ERC1;PCBD2;KIFAP3;XRCC4;GABRA5;ATRNL1;LRBA;TRAPPC8;ELP4;MYRIP;FAM126B;SEZ6L;FOXP2;MCM3AP;TMEM135;BRE;NF1;CREBRF;XKR4;SKAP2;CCDC171*
H3K4me3 G1E mm9	29/2531	1.0	*SGMS1;CLSTN2;PRDM15;ADK;MIR96;GPHN;EFTUD1;TTC28;PPP3CA;RELN;ERC1;PCBD2;HMCN1;KIFAP3;SH3GL2;XRCC4;ATRNL1;MGMT;LRBA;MYRIP;FOXP2;STXBP5L;MCM3AP;STIM1;BRE;NF1;APBA1;XKR4;CCDC171*
H3K36me3 GM12878 hg19	20/2531	1.0	*LINC00273;LRRC69;PLEKHB2;ANKRD30BL;PDE4D;MIR663A;FRG1B;FAM126B;BACH1;ROCK1P1;MRPL33;EFTUD1;MIR3648;MIR3687;LARGE;FAM110B;PIEZO2;MIR4461;MDN1;SHANK2*
H3K4me1 G1E mm9	27/2521	1.0	*FHOD3;ANKRD36;CPQ;BICD1;EHBP1;MIPOL1;AKAP13;CCDC91;SNTG1;SOX6;KIFAP3;ADAMTS6;SPECC1;PACRG;ATRNL1;MGMT;SHFM1;EXOC6B;MERTK;FOXP2;STXBP5L;LCA5L;VWA8;DNM3;LANCL3;APBA1;CCDC171*
H3K36me3	17/2514	1.0	*LINC00273;ANKRD30BL;SHFM1;MIR663A;PCDHA11;PCDH*

cardiac mesoderm hg19			*A10;MIR3648;TIAM2;MCM3AP;MIR3687;PCDHA3;FAT3;UTRN;MIR4461;TBC1D22A;DOCK1;FREM2*
H3K36me3 HepG2 hg19	13/2389	1.0	*LINC00273;PLEKHB2;ANKRD30BL;IFNGR2;WRB;MIR663A;FAM126B;BACH1;ROCK1P1;MIR3648;MIR3687;MIR4461;MDN1*
H3K27ac testis mm9	22/2000	1.0	*LRRC69;CCDC178;PLEKHB2;LAMA2;SEMA6D;MAGI2;PRDM15;ATP1A4;FHIT;SUMF1;ENOX2;AGBL4;PPP3CA;CDK6;RPS6KA5;SPTLC2;CCDC30;CREBRF;TTLL11;TBC1D22A;DOCK1;SKAP2*
H3K27ac kidney mm9	22/2000	1.0	*PON3;PDE1C;SEMA6D;LRRK2;MAST2;TSHZ2;SUSD1;EXOC6B;FAM126B;SUMF1;B3GALTL;ENOX2;CACNB2;RPS6KA5;IFT43;NBEA;ASXL3;SRGAP3;NCKAP5;DOCK1;FREM2;SKAP2*
H3K4me3 bone marrow mm9	22/2000	1.0	*PON3;HFM1;EDA;PLEKHB2;WRB;SEMA6D;SUSD1;PRDM15;FBXL17;MERTK;FHIT;MRPL33;SUMF1;RUNX1;EFTUD1;DHX32;IFT43;NSG2;ADAMTS17;CCDC30;SAMD12;ST6GALNAC3*
H3K4me1 HeLa-S3 hg19	22/2000	1.0	*DGKB;EXOC6B;FMN1;PHKB;MIR663A;UPK3B;ROCK1P1;SAMSN1;ARHGAP24;GPHN;SUMF1;RUNX1;ADAMTSL1;NCOR1;DLG2;IFT43;WDPCP;TTLL11;HIVEP3;CTNNA3;TLN2;SKAP2*
H3K27ac Panc1 hg19	22/2000	1.0	*EPHA5;ZNF493;HFM1;CTBP2;SGMS1;XRCC4;ADK;ANK2;CACNA1D;FAM126B;PCDHA12;SUMF1;MIPOL1;AKAP13;DIAPH3;ZNF717;MIR3687;CDC42EP3;SAMD12;KIAA0825;SKAP2;ZNF585A*
H3K9ac CH12.LX mm9	22/2000	1.0	*CSGALNACT1;INVS;FHOD3;AVEN;DTNA;ATRNL1;MGMT;FAM126B;PARD3B;TIAM2;VWA8;MCM3AP;NSG2;BRE;CDC42EP3;NF1;HIVEP3;APBA1;SRGAP3;ERC1;TBC1D22A;CCDC171*
H3K27ac embryonic fibroblast mm9	22/2000	1.0	*SPECC1;PON3;SEMA6D;SHFM1;LRRK2;PRDM15;MRPL33;PTPRG;B3GALTL;VWA8;MCM3AP;SGCD;RPS6KA5;NBEA;SPTLC2;SRGAP3;UTRN;NCKAP5;MAPRE2;GPC6;DOCK1;SCAI*
H3K4me1 bone marrow mm9	22/2000	1.0	*CSGALNACT1;EPHA5;ATP8A2;SYCP2;MGMT;GAREM;RANBP17;KAZN;TRAPPC8;ANK2;CACNA1C;FOXP2;EHBP1;SLC2A9;AKAP13;RPS6KA5;DHX32;KYNU;GPC5;ERC1;SAMD12;ANKS1B*
H3K36me3 HeLa-S3 hg19	15/2234	1.0	*CSGALNACT1;LINC00273;PLEKHB2;ANKRD30BL;COL25A1;PTPRM;MIR663A;ROCK1P1;THSD4;MIR3648;MIR3687;CDC42EP3;FSD2;FPGT-TNNI3K;MDN1*
H2AFZ CD14-positive monocyte hg19	21/2000	1.0	*AVEN;OCA2;KHDRBS2;CSRNP3;SGMS1;COL25A1;ATP1A4;FRG1B;LRRC16A;MERTK;ARHGAP24;MIPOL1;ARHGAP22;DNM3;AGBL4;ADAMTS17;ELMO1;PCDHA3;TCF4;CSMD3;PCBD2*
H3K27ac mammary epithelial cell hg19	21/2000	1.0	*SPAG16;XRCC4;DNAH6;PHKB;ELOVL7;VWA3B;ELP4;FHIT;TEKT4P2;MIPOL1;PTPRD;ENOX2;TMEM135;RPS6KA5;ZNF717;WDPCP;SLIT3;FPGT-TNNI3K;PCBD2;TBC1D22A;MDN1*
H3K4me3 myocyte mm9	21/2000	1.0	*ROBO2;TENM4;SEMA3D;SHFM1;LRRK2;FMN1;PHKB;ELP4;FAM126B;MERTK;ARID1B;EFTUD1;SPTLC2;TBC1D5;CCDC30;SAMD12;TBC1D22A;N4BP2;ADAMTS6;PKNOX2;SKAP2*
H3K36me3 T-cell acute lymphoblastic leukemia hg19	21/2000	1.0	*PCDH9;MGMT;NOTCH2NL;FBXL17;MIR663A;ELP4;FRG1B;FAM126B;BACH1;ROCK1P1;GPHN;MIR3648;B3GALTL;NCOR1;SPTLC2;BAGE2;MIR3687;KIFAP3;ZNF675;TBC1D22A;SKAP2*
H3K79me2 endothelial cell of umbilical vein hg19	21/2000	1.0	*AVEN;ZNF283;PCDH9;ANKRD30BL;ANKRD36;MIR663A;FRG1B;ROCK1P1;SUMF1;EFTUD1;TTC28;B3GALTL;PGM5P2;IFT43;NRG3;BRE;MIR3687;WDPCP;KIFAP3;N4BP2;SCAI*
H3K27ac HeLa-S3 hg19	21/2000	1.0	*SPAG16;XRCC4;COL25A1;LRBA;ADK;ARAP2;TRAPPC8;ELP4;FAM126B;MIPOL1;TMEM135;RPS6KA5;DIAPH3;WDPCP;FPGT-TNNI3K;SAMD12;N4BP2;DOCK1;WLS;SKAP2;ZNF585A*
H3K36me3 fibroblast of lung hg19	21/2000	1.0	*WDR27;SEMA5A;LINC00273;ZNF283;ANKRD30BL;SUSD1;FMN1;PHKB;MIR663A;ROCK1P1;THSD4;RUNX1;MIR3648;TIAM2;SH3TC2;MIR3687;SCN8A;FAM110B;TTLL11;DOCK1;SHANK2*
H3K27ac spleen mm9	21/2000	1.0	*INVS;SGMS1;SEMA6D;MAST2;SUSD1;PTPRM;MERTK;FHIT;ARHGAP24;RUNX1;EFTUD1;B3GALTL;ENOX2;MCM3AP;RPS6KA5;BRE;SPTLC2;DLC1;ST6GALNAC3;MAPRE2;SKAP2*
H3K79me2 HeLa-S3 hg19	20/2000	1.0	*WDR27;ZNF283;MGMT;VPS13D;PHKB;FAM126B;ROCK1P1;TEKT4P2;SUMF1;MIPOL1;STXBP5L;EFTUD1;MSRA;CCDC91;DHX32;BRE;MIR3687;MIR183;KIFAP3;WLS*
H3K79me2 mammary epithelial	20/2000	1.0	*LINC00273;ZNF283;WRB;MIR663A;FRG1B;MIR96;ROCK1P1;TEKT4P2;THSD4;MIR3648;ARHGAP22;DNM3;PGM5P2;MCM3AP;IFT43;BRE;MIR3687;MIR183;WDPCP;SCAI*

cell hg19			
H4K20me1 B cell hg19	20/2000	1.0	*WDR27;LINC00273;INVS;AVEN;OCA2;CTBP2;XRCC4;ANKRD30BL;SUSD1;ADK;NOTCH2NL;MIR663A;VWA3B;ROCK1P1;MIR3648;MCM3AP;RPS6KA5;MIR3687;WDPCP;CORIN*
H3K4me1 HCT116 hg19	20/2000	1.0	*PLEKHB2;IFNGR2;MAST2;FBXL17;ARAP2;COBL;FMN1;MYRIP;UPK3B;ROCK1P1;EHBP1;MIPOL1;MCM3AP;TMEM135;SH3TC2;CPE;SRGAP3;TLN2;SAMD12;WLS*
H4K20me1 fibroblast of dermis hg19	20/2000	1.0	*LINC00284;LINC00273;DYNC1H1;GABBR2;PRMT8;SHFM1;RANBP17;MIR663A;NAV2;ROCK1P1;RERG;PARD3B;MIR3648;AKAP13;FAM155A;MIR3687;HECW1;SRGAP3;PLXNA4;PRKG1*
H3K79me2 heart mm9	20/2000	1.0	*WDR27;INVS;SGMS1;CUL5;SHFM1;VPS13D;FNDC3B;EXOC6B;ARAP2;PTPRM;TRAPPC8;CACNA1D;PARD3B;PPP3CA;PLCB4;NBEA;CDC42EP3;MACROD2;UTRN;CORIN*
H3K79me2 myotube hg19	20/2000	1.0	*ZNF283;ANKRD30BL;VPS13D;MIR663A;FRG1B;FAM126B;LRRC16A;ROCK1P1;TEKT4P2;SUMF1;TTC28;ENOX2;FRAS1;DHX32;BRE;MIR3687;FPGT-TNNI3K;MDN1;SCAI;ZNF585A*
H3K79me2 CH12.LX mm9	19/2000	1.0	*WDR27;INVS;XRCC4;LRBA;MAST2;PHKB;FAM126B;SAMSN1;GPHN;SUMF1;MIPOL1;PPP3CA;MCM3AP;TMEM135;NCOR1;CDK6;RPS6KA5;NF1;TBC1D22A*
H3K79me2 CD14-positive monocyte hg19	19/2000	1.0	*AVEN;ZNF493;RTN1;ZNF283;ANKRD30BL;MAST2;MIR663A;FRG1B;ROCK1P1;FHIT;BICD1;TEKT4P2;MIR3648;MCM3AP;MIR3687;FPGT-TNNI3K;KIFAP3;N4BP2;MDN1*
H3K27ac B cell hg19	19/2000	1.0	*AVEN;XRCC4;MAST2;TRAPPC8;VWA3B;ELP4;FRG1B;ROCK1P1;MRPL33;MCM3AP;PAK1;TMEM135;FPGT-TNNI3K;ERC1;KIFAP3;ZNF675;MDN1;ADAMTS6;ZNF585A*
H3K79me2 astrocyte hg19	19/2000	1.0	*WDR27;ACSS3;ZNF283;ANKRD30BL;WRB;MAGI2;MIR663A;FAM126B;ROCK1P1;SUMF1;PARD3B;PGM5P2;MCM3AP;IFT43;ZNF717;MIR3687;WDPCP;SCAI;ZNF585A*
H4K20me1 endothelial cell of umbilical vein hg19	19/2000	1.0	*CSGALNACT1;LINC00273;SLC24A2;SPECC1;GABBR2;RTN1;RANBP17;NOTCH2NL;MIR663A;FRG1B;ROCK1P1;FOXP2;ZNF280D;MIR3648;SPTLC2;MIR3687;SCN8A;ERG;EIF4G3*
H3K4me3 NB4 hg19	19/2000	1.0	*PACRG;HFM1;ZNF283;CUL5;PLEKHB2;AUTS2;LRBA;MAST2;NOTCH2NL;ZNF804A;PRDM11;KY;GPHN;DACH1;IFT43;ZNF717;ADAMTS17;SRGAP3;EIF4G3*
H3K27ac K562 hg19	19/2000	1.0	*WDR27;XRCC4;ADK;FBXL17;MIR663A;FRG1B;ROCK1P1;MRPL33;RUNX1;EFTUD1;RPS6KA5;RELN;ZNF717;BAGE2;TBXAS1;MIR183;FPGT-TNNI3K;KIAA0825;GPC6*
H3K27ac CH12.LX mm9	18/2000	1.0	*INVS;AVEN;WRB;ADK;PHKB;TRAPPC8;ANK2;ARID1B;GPHN;SUMF1;EFTUD1;PPP3CA;MCM3AP;TMEM135;CTNNA3;TBC1D22A;MDN1;SKAP2*
H3K36me3 testis mm9	18/2000	1.0	*CCDC178;PACRG;SYCP2;PDE1C;ANKRD36;MAGI2;VPS13D;CACNA1D;FAM227B;MIPOL1;TTC28;SPATA16;NCOR1;CDC42EP3;TTLL11;MACROD2;SOX6;EIF4G3*
H4K20me1 CD14-positive monocyte hg19	18/2000	1.0	*LINC00273;EDA;ANKRD30BL;MAST2;EXOC6B;TACR3;FMN1;MIR663A;VWA3B;FAM126B;SEZ6L;ROCK1P1;SAMSN1;MIR3648;RPS6KA5;MIR3687;GPM6B;CORIN*
H3K9me1 K562 hg19	18/2000	1.0	*ABCG8;CHRM5;EXOC6B;AKR1C2;TRAPPC8;MIR663A;FRG1B;LRRC16A;STXBP5L;CA3;BAGE2;MIR1324;FSD2;MIR4461;N4BP2;GPC6;ADAMTS6;SHANK2*
H3K27ac skeletal muscle myoblast hg19	18/2000	1.0	*SPAG16;COL25A1;ADK;GLIS3;PHKB;TRAPPC8;FRG1B;FAM126B;TEKT4P2;PTK2;MIPOL1;PARD3B;PPP3CA;WDPCP;FPGT-TNNI3K;PCBD2;TBC1D22A;KIAA0825*
H3K27ac HCT116 hg19	18/2000	1.0	*XRCC4;SLC35F3;SHFM1;DNAH14;ADK;ARAP2;LRRC16A;UPK3B;ROCK1P1;MRPL33;ARHGAP22;NCOR1;DIAPH3;TBXAS1;WDPCP;TBC1D22A;N4BP2;MDN1*
H3K27ac brown adipose tissue mm9	18/2000	1.0	*PON3;PLEKHB2;TENM4;MGMT;SEMA6D;MAGI2;EXOC6B;MRPL33;B3GALTL;MCM3AP;SGCD;DLG2;IFT43;BRE;MAPRE2;DOCK1;SKAP2;RUNX1T1*
H3K79me2 fibroblast of lung hg19	18/2000	1.0	*ACSS3;ZNF493;ZNF283;XRCC4;ANKRD30BL;LRRK2;NAV2;ROCK1P1;TEKT4P2;SUMF1;RUNX1;MIR3648;PGM5P2;MCM3AP;ZNF717;MIR3687;WDPCP;FPGT-TNNI3K*
H3K79me2 K562 hg19	17/2000	1.0	*ZNF283;ANKRD30BL;ANKRD36;WRB;ROCK1P1;TEKT4P2;SUMF1;TTC28;MCM3AP;BRE;ZNF717;MIR3687;WDPCP;EIF4G3;TBC1D22A;N4BP2;GPC6*
H3K36me3 mammary epithelial cell hg19	17/2000	1.0	*LINC00273;ZNF283;PLEKHB2;DSCAM;ANKRD30BL;SEMA3D;MIR663A;FAM126B;ROCK1P1;THSD4;EFTUD1;MIR3648;MIR3687;FSD2;HIVEP3;FPGT-TNNI3K;ADAMTS6*

H3K9ac T-cell acute lymphoblastic leukemia hg19	17/2000	1.0	*SPAG16;AVEN;MGMT;DNAH14;MIR663A;CACNA1C;PTPRG;MCM3AP;PAK1;PLCB4;DIAPH3;WDPCP;SRGAP3;N4BP2;KIAA0825;ADAMTS6;ZNF585A*
H3K79me2 H1-hESC hg19	17/2000	1.0	*ANKRD30BL;DNAH14;VPS13D;SUSD1;MIR663A;MIR96;ROCK1P1;SUMF1;IFT43;ZNF717;BAGE2;MIR3687;MIR183;GPR139;SRGAP3;MIR4461;ZNF585A*
H3K9ac MEL cell line mm9	16/2000	1.0	*FHOD3;AVEN;PON3;SGMS1;LRBA;PRDM15;ADK;ELP4;FAM126B;GPHN;LCA5L;CDC42EP3;APBA1;ERC1;PCDHA8;N4BP2*
H3K36me3 astrocyte hg19	16/2000	1.0	*LINC00273;LRRC69;PLEKHB2;IFNGR2;WRB;MIR663A;ROCK1P1;THSD4;MIR3648;DHX32;MIR3687;LARGE;FSD2;FPGT-TNNI3K;ADAMTS6;WLS*
H3K36me3 myotube hg19	16/2000	1.0	*LINC00273;LRRC69;SLC24A2;KIRREL3;CLSTN2;ANKRD30BL;SHFM1;PTPRM;MIR663A;ROCK1P1;MIR3648;TIAM2;MIR3687;SCN8A;FAM110B;FPGT-TNNI3K*
H3K27ac ES-E14 mm9	16/2000	1.0	*SGMS1;XRCC4;ATRNL1;ADK;HUNK;TRAPPC8;FAM126B;ARID1B;PTK2;GPHN;MIPOL1;AKAP13;NF1;SRGAP3;ERC1;TBC1D22A*
H3K36me3 CD14-positive monocyte hg19	16/2000	1.0	*PLEKHB2;ANKRD30BL;IFNGR2;LRRK2;ZNF804A;MIR663A;FRG1B;FAM126B;ROCK1P1;MIR3648;BAGE2;MIR3687;FSD2;FPGT-TNNI3K;MIR4461;WLS*
H3K27ac bone marrow macrophage mm9	16/2000	1.0	*AVEN;PON3;SGMS1;CUL5;PLEKHB2;MGMT;PHKB;TRAPPC8;SAMSN1;MRPL33;SUMF1;B3GALTL;PPP3CA;MCM3AP;CDK6;RPS6KA5*
H3K27ac thymus mm9	16/2000	1.0	*AVEN;DYNC1H1;PRDM15;ADK;AFF3;GPHN;MRPL33;MIPOL1;RUNX1;ENOX2;AKAP13;NSG2;BRE;CDC42EP3;PRRG1;PCBD2*
H3K36me3 skeletal muscle myoblast hg19	15/2000	1.0	*LINC00273;ZNF493;PLEKHB2;SUSD1;MIR663A;ROCK1P1;BICD1;LCA5L;MIR3648;BRE;MIR3687;SCN8A;UTRN;GPC6;SHANK2*
H3K27ac T-cell acute lymphoblastic leukemia hg19	15/2000	1.0	*WDR27;AVEN;ZNF283;XRCC4;PLCL1;TRAPPC8;ELP4;MCM3AP;SRGAP3;EIF4G3;N4BP2;KIAA0825;ADAMTS6;SKAP2;ZNF585A*
H3K27ac CD14-positive monocyte hg19	15/2000	1.0	*ZNF283;SGMS1;LRBA;WRB;EXOC6B;FBXL17;ELP4;MERTK;ROCK1P1;SAMSN1;FHIT;ARHGAP24;PAK1;DACH1;MDN1*
H3K27ac MCF-7 hg19	15/2000	1.0	*ACSS3;LRBA;SHFM1;DNAH14;ARAP2;ELP4;FAM126B;TEKT4P2;MRPL33;SUMF1;NFIA;BAGE2;HMCN1;FREM2;PCDHA6*
H3K27ac keratinocyte hg19	15/2000	1.0	*SGMS1;XRCC4;DNAH6;ELOVL7;VWA3B;ROCK1P1;TEKT4P2;MIPOL1;TMEM135;BRE;ZNF717;TBXAS1;FPGT-TNNI3K;HMCN1;SAMD12*
H3K36me3 brain mm9	14/2000	1.0	*WDR27;VPS13D;NRXN3;ANK2;CACNA1D;CACNA1C;PCDHA12;PCDHA11;MRPL33;NCOR1;NDST3;FAT3;PCBD2;PCDHA7*
H3K27ac MEL cell line mm9	14/2000	1.0	*WRB;SHFM1;MAST2;SUSD1;FAM126B;ARHGAP15;AFF3;MRPL33;B3GALTL;ENOX2;VWA8;MCM3AP;TMEM135;CTNNA3*
H3K36me3 H1-hESC hg19	14/2000	1.0	*SGMS1;IFNGR2;ADCY2;MIR663A;ROCK1P1;PTPRG;SPTLC2;BAGE2;LARGE;ST6GALNAC3;MIR4461;DOCK1;FREM2;SHANK2*
H3K79me2 HepG2 hg19	13/2000	1.0	*ZNF493;ZNF283;ANKRD30BL;MAGI2;MIR663A;ROCK1P1;TEKT4P2;SUMF1;ENOX2;MIR3687;FPGT-TNNI3K;PCBD2;ZNF585A*
H3K4me1 NT2-D1 hg19	13/2000	1.0	*PRMT8;SATB2;VWA3B;CACNA1C;MIR96;UPK3B;BACH1;HDAC9;LCA5L;ARHGAP22;MIR183;GPR139;ST6GALNAC3*
H3K36me3 fibroblast of dermis hg19	13/2000	1.0	*SHFM1;MIR663A;FRG1B;ROCK1P1;RUNX1;MIR3648;MIR3687;SCN8A;FAM110B;FSD2;FPGT-TNNI3K;DIP2C;DOCK1*
H3K79me2 myocyte mm9	13/2000	1.0	*CSGALNACT1;INVS;SPECC1;IFNGR2;WRB;EXOC6B;PTK2;TIAM2;IFT43;UTRN;TBC1D22A;MDN1;ADAMTS6*
H3K79me2 T-cell acute lymphoblastic leukemia hg19	13/2000	1.0	*PCDH9;ANKRD30BL;ANKRD36;MIR663A;ROCK1P1;FHIT;SUMF1;ENOX2;IFT43;MIR3687;FPGT-TNNI3K;PCBD2;ZNF585A*
H3K79me2 C2C12 mm9	12/2000	1.0	*SPECC1;PAK1;CDK6;BRE;PLEKHB2;CDH13;TTLL11;FAM126B;UTRN;LRRC4C;TBC1D22A;MIPOL1*

H3K27ac ES-Bruce4 mm9	12/2000	1.0	*VWA8;CSRNP3;CUL5;BRE;PRDM15;ADK;PHKB;ERC1;PCBD2;MDN1;MRPL33;CCDC171*
H3K36me3 osteoblast hg19	12/2000	1.0	*TIAM2;PLEKHB2;FAM110B;FSD2;MIR663A;FPGT-TNNI3K;ROCK1P1;BICD1;TBC1D22A;ADAMTS6;THSD4;SCAI*
H3K27ac placenta mm9	12/2000	1.0	*INVS;VWA8;SPECC1;PON3;MCM3AP;SEMA6D;SHFM1;SUSD1;PHKB;KIFAP3;SKAP2;MRPL33*
H2AFZ T-cell acute lymphoblastic leukemia hg19	11/2000	1.0	*ZNF493;DACH1;ZNF283;BRE;CDC42EP3;DPYD;PHKB;FPGT-TNNI3K;FAM126B;MDN1;MRPL33*
H3K79me3 C2C12 mm9	11/2000	1.0	*WDR27;MCM3AP;PAK1;CDK6;WRB;LARGE;WDPCP;FAM126B;LRRC4C;TBC1D22A;PTK2*
H3K36me3 ES-E14 mm9	10/2000	1.0	*NCOR1;CTBP2;IFNGR2;VPS13D;ARAP2;KAZN;MIR183;CACNA1D;MIR96;SCAI*
H3K36me3 small intestine mm9	10/2000	1.0	*MGAM;NCOR1;VPS13D;KAZN;CACNA1D;PCBD2;CACNA1C;UTRN;MERTK;SHANK2*
H3K36me3 ES-Bruce4 mm9	9/2000	1.0	*WDR27;NCOR1;CTBP2;KAZN;MIR183;CACNA1D;UTRN;MIR96;MIPOL1*
H3K36me3 liver mm9	9/2000	1.0	*LRRC69;ENOX2;PON3;KLF12;NCOR1;CACNA1D;PCBD2;TLN2;UTRN*
H3K36me3 U2OS hg19	9/2000	1.0	*WDR27;LRRC69;TIAM2;MCM3AP;ZNF283;CUL5;UTRN;DOCK1;SHANK2*
H3K36me3 spleen mm9	8/2000	1.0	*MCM3AP;NCOR1;VPS13D;CACNA1D;CACNA1C;EIF4G3;FAM227B;EFTUD1*
H3K36me3 splenic B cell mm9	3/2000	1.0	*VPS13D;CACNA1D;EIF4G3*
H3K36me3 NT2-D1 hg19	5/2000	1.0	*TIAM2;MCM3AP;ZNF283;UTRN;BACH1*
H3K36me3 H7 hg19	11/1851	1.0	*MIR3648;LINC00273;SLC24A2;TIAM2;ANKRD30BL;MIR3687;MIR663A;MIR4461;TBC1D22A;DOCK1;SHANK2*
H3K36me3 bronchial epithelial cell hg19	6/1681	1.0	*MIR3648;LINC00273;ANKRD30BL;MIR3687;MIR663A;BACH1*
H3K36me3 MCF-7 hg19	5/1395	1.0	*ANKRD30BL;MIR4461;PCDHA12;ROCK1P1;FREM2*
H3K36me3 BJ hg19	7/1302	1.0	*MIR3648;LINC00273;TIAM2;ANKRD30BL;MIR3687;MIR663A;BACH1*
H3K36me3 GM06990 hg19	6/1255	1.0	*MIR3648;LINC00273;ANKRD30BL;MIR3687;MIR663A;BACH1*
H3K36me3 thymus mm9	5/1235	1.0	*MCM3AP;NCOR1;VPS13D;CACNA1D;CACNA1C*
H3K36me3 kidney mm9	5/1187	1.0	*MCM3AP;NCOR1;LRRK2;KAZN;CACNA1D*
